# Application of a Screening‐Level Pollinator Risk Assessment Framework to Trisiloxane Polyether Surfactants

**DOI:** 10.1002/etc.5479

**Published:** 2022-10-20

**Authors:** Jennifer K. Collins, Jennifer M. Jackson

**Affiliations:** ^1^ Waterborne Environmental Leesburg Virginia USA

**Keywords:** Ecological risk assessment, ecotoxicology, pollinator risk assessment, terrestrial invertebrate toxicology, trisiloxane polyether surfactants

## Abstract

Regulatory requirements exist to assess the potential impacts of pesticides on insect pollinators, but “inert,” coformulants to pesticide formulations are not included in standard regulatory risk assessments. Some publications in the open literature have suggested that the agricultural uses of “inert” ingredients, including trisiloxane polyether surfactants, may result in adverse effects on pollinators. We conducted a screening‐level risk assessment to evaluate the potential risk to insect pollinators, using honey bees (*Apis mellifera*) as a surrogate, from exposure to three trisiloxane polyether surfactants based on agricultural application scenarios following the current US Environmental Protection Agency (USEPA) guidance. The exposure assessment included data from two sources: (1) use data reported in California's (USA) Pesticide Use Registry (PUR) database for all crops, and (2) an almond orchard residue study conducted using the three trisiloxane polyether surfactants. Honey bee laboratory studies with each of the trisiloxane polyether surfactants reported 50% lethal doses (LD50s) or no adverse effect levels, which were used as the effects inputs to BeeREX. The exposure and toxicity data were combined to estimate potential honey bee risk based on the determination of acute and chronic risk quotients (RQs) for larval and adult life stages. The RQs calculated using both the PUR use rates as well as the application rates and peak measured residues from the almond orchard residue study were below the USEPA acute and chronic levels of concern (acute, 0.4; chronic, 1.0). Based on these results, the use of these three trisiloxane polyether surfactants in agricultural use settings can be considered minimal risk to insect pollinators, and higher tier assessment is unnecessary for the characterization of risk. *Environ Toxicol Chem* 2022;41:3084–3094. © 2022 The Authors. *Environmental Toxicology and Chemistry* published by Wiley Periodicals LLC on behalf of SETAC.

## INTRODUCTION

The health and resilience of ecological habitats and agricultural sustainability rely on pollinating insects (Van Klink et al., 2020). Reports of significant decline of insect pollinators in recent decades (National Research Council et al., 2007) have prioritized pollinator protection goals for regulatory and environmental agencies in the United States and across the globe (European Food Safety Authority [EFSA], 2021; Pollinator Health Task Force, 2015). Because agricultural chemicals have the potential to cause adverse ecological effects, the likelihood and magnitude of potential adverse effects on organisms that are not targeted by the pesticide are assessed through environmental risk assessment (US Environmental Protection Agency [USEPA], 2021a, 2021b).

The current guidance for assessing pesticide risks to honey bees was issued in 2014 by the USEPA, the Health Canada Pest Management Regulatory Agency, and the California (USA) Department of Pesticide Regulation (USEPA, PMRA, & CDPR, 2014). This guidance provides a framework for characterizing the potential risks of pesticides to honey bees (*Apis mellifera*), which are also used as a surrogate species for bees and other insect pollinators. The pollinator risk characterization framework follows a tiered process similar to the USEPA ecological risk assessment framework for other organisms (USEPA, 2021a, 2021b), in which a screening‐level (tier 1) assessment is first applied using a relatively simple and conservative approach. In this screening‐level assessment, estimated environmental concentrations (EECs) and toxicity estimates of a compound are used to calculate pollinator‐specific risk quotients (RQs), which can then be compared with defined acute and chronic levels of concern (LOCs) to determine whether any higher tier assessments are required.

To date, the USEPA pollinator risk assessment guidance has been developed and primarily applied to pesticide‐active ingredients, because the technical‐grade‐active ingredients are the basis for regulatory review for agricultural chemicals. However, studies in the published literature indicate that “inert” ingredients of agricultural pesticide formulations may have potential impacts on pollinators (Chen & Mullin, 2015; Kordecki, 2019; Mesnage & Antoniou, 2018; Mullin, 2015; Mullin et al., 2015). Agricultural “inert” ingredients may include solvents, surfactants, carriers, adjuvants, or tank‐mix additives included in pesticide formulations to improve the safety, effectiveness, and efficiency of agricultural‐active ingredients. Although the trisiloxane polyether surfactants are commonly refered to as “inerts,” this is actually a misnomer because these chemicals play an important role as adjuvants, dispersants, emulsifiers, or antifoam agents. Thus, these trisiloxane polyether surfactants are not the active ingredient but function as coformulates. The hydrophobic characteristics of plant surfaces act as a natural barrier to wetting, which can negatively impact the application and efficacy of plant protection products. For this reason, surfactants are widely used with pesticides. Specifically, trisiloxane polyether surfactants (also referred to as organomodified siloxanes) have the unique ability to significantly reduce the surface tension of aqueous solutions to promote a rapid spreading of aqueous solutions on hydrophobic surfaces (Hill, 2003). The use of trisiloxane polyether surfactants can reduce the required amount of plant protection products. In addition, these surfactants can improve the efficiency of plant protection products by enabling reduction of spray volumes and runoff and can therefore contribute to a reduction of pesticide‐active ingredient in the environment (Gaskin et al., 2004, 2005).

Studies in published literature have suggested a potential toxicity of trisiloxane polyether surfactants to bees and other insects. One of the first such studies reported that a trisiloxane polyether surfactant is capable of deterring honey bees from visiting pond water at a concentration of 500 mg/L (Moffett & Morton, 1975). Ciarlo et al. (2012) reported a significant reduction in honey bee olfactory learning ability following oral ingestion of trisiloxane polyether surfactants. Mullin et al. (2015) report that trisiloxane polyether surfactants have insecticidal properties, with toxicity to a range of terrestrial arthropods (aphids, fruit flies, citrus leafminers, spider mites, and thrips). Mullin et al. (2015) also state that trisiloxane polyether surfactants are potentially more toxic to bees compared with other nonionic adjuvants. The same authors report that median lethal dose (LC50) values can be as low as 10 mg/L following oral consumption of commercial trisiloxane polyether surfactant (neat material). It should be noted that this value is presented as a single solution concentration, no other doses were used in the present study, and the rate was not corrected for consumption, as is the standard procedure for a pollinator risk assessment (i.e., µg/bee). Interest in the potential impacts of trisiloxane polyether surfactants on pollinators has also been prompted by the increase in product use in agricultural settings and historical limitations in analytical methodology (Mullin et al., 2015, 2016). Until robust analytical methods were developed, it was not possible to reliably detect trisiloxane polyether surfactants at low concentrations.

The purpose of the present risk assessment was to evaluate the ecological risk of trisiloxane polyether surfactants using the screening‐level pollinator risk assessment framework accepted for assessing pesticides in the United States. Honey bees (*Apis mellifera*) are used as a surrogate, to be representative of pollinator species, and exposure to the three trisiloxane polyether surfactants was characterized from modeled exposure data and environmental residue data. The potential risk to insect pollinators was evaluated using the established USEPA, PMRA, & CDPR, (2014) pollinator risk assessment framework and the BeeREX model developed by these regulatory agencies.

## MATERIALS AND METHODS

### Trisiloxane polyether surfactants

Three trisiloxane polyether surfactants were included in our assessment: trisiloxane‐317 (oxirane, methyl‐, polymer with oxirane mono[3‐[1,3,3,3‐tetramethyl‐1‐[(trimethylsilyl)oxy]disiloxanyl]propyl]ether; Chemical Abstract Service Registry Number [CAS RN] 134180‐76‐0), trisiloxane‐OH (3[hydroxyl(polyethyleneoxy)propyl]‐heptamethyltrisiloxane; CAS RN 67674‐67‐3), and trisiloxane‐acetoxy (3‐{2‐[acetoxy(polyethyleneoxy)propyl]} heptamethyltrisiloxane; CAS RN 125997‐17‐3). These three substances are representative of the class of trisiloxane polyether surfactants and are used in several commercial products. These products are tank‐mix adjuvants (rather than built into the formulated plant protection product, i.e., the grower adds them to the tank), which are combined with formulated pesticide products prior to spray applications. The trisiloxane polyether surfactant products are used neat or as formulated materials (blended with other materials) prior to tank‐mix with insecticides, herbicides, fungicides, plant growth regulators, and fertilizers for all crop types (Hill, 2002, 2003).

### Screening‐level pollinator risk assessment

**Figure 1:**
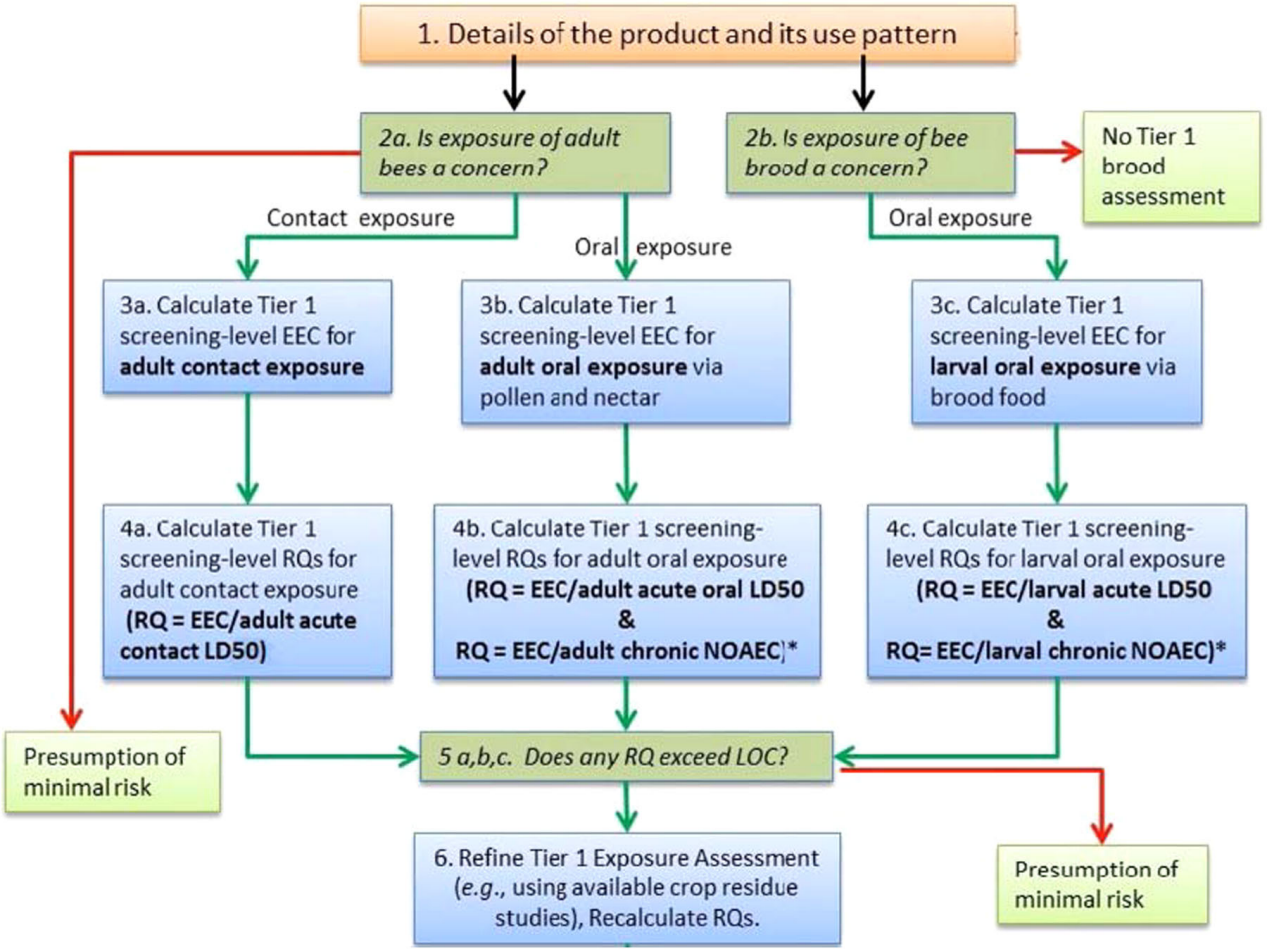
Screening‐level pollinator risk assessment approach for foliar spray applications. ∗ = risk quotient. RQ, risk quotient; LD50, median lethal dose; LOC, level of concern; EEC, estimated environmental concentration; NOAEC, no‐observed adverse effect concentration (reprinted from US Environmental Protection Agency, 2014).

The screening‐level BeeREX modeling tool is intended to estimate the exposure of bees to pesticides and calculate RQs that can be used within a tier 1 risk assessment under the regulatory guidance of the USEPA, the Canadian PMRA, and California's CDPR. The screening‐level pollinator risk assessment framework is presented in Figure 1. We used the BeeREX model (USEPA, PMRA, & CDPR, 2014) to estimate exposure and calculate RQs from EEC data and toxicological effects endpoints. The RQs were then compared with USEPA‐established LOCs to evaluate pollinator risk.

### Exposure characterization

Information derived from two sources was used to define the EEC: (1) grower use‐data reported in the CDPR Pesticide Use Registry (PUR) database (CDPR, 2017) for all crops, and (2) residue concentrations measured during an almond orchard residue study conducted with the three trisiloxane polyether surfactants. The respective use and residue data were used as inputs into the BeeREX model to quantify potential honey bee exposure.

The State of California defines tank‐mix adjuvants (packaged and sold separately from pesticides) as pesticide products and requires their registration (CDPR, 2011). Based on California's definition of adjuvant products, the CDPR PUR database includes monthly adjuvant use entries. Therefore, the CDPR PUR database is a relatively dependable source of use data for the trisiloxane polyether surfactants for all crops grown in California (Wilhoit, 2018). For the purpose of the present assessment, we used 2017 data because it was the most current and comprehensive annual use data available at the time of our study. Queries were designed based on CAS RN and chemical name to extract the relevant use data (in pounds/acre) for all 2017 applications of the three trisiloxane polyether surfactants by crop.

The resulting use rate data from the queries of the CDPR PUR database were reviewed by crop. In some instances, use values were orders of magnitude above the feasible use rates of the trisiloxane polyether surfactants in agricultural settings, which would likely cause high crop phytotoxicity (Falk et al., 1994; Sun et al., 2003). As expected with large‐scale user‐reporting databases, entry or transcription errors can be expected, and the CDPR acknowledges the existence of such errors within the database (CDPR, 2017). In the case of tank‐mix adjuvant products, the units of use rates may have been an additional source of data entry error because these products are typically added as a volume percentage to the tank mix. Therefore, an outlier analysis was conducted using the interquartile range (IQR) method (Han et al., 2012). The upper limit was defined as the value of the third quartile plus 1.5 × the IQR. All application rates above this defined upper‐bound limit were considered outliers and were excluded from the analysis. Following the outlier analysis, an additional empirical review of each data set was conducted to identify values in the CDPR PUR database that likely represent input errors. Likely input errors were defined as reported application rates more than 1 order of magnitude higher than the highest use rates on commercial product labels. The resulting queried data set was compared with the highest label rate for the three trisiloxane polyether surfactants on commercial labels. Trisiloxane‐317 is used in adjuvant blend products as well as a neat product. Accurate characterization of the exposure to the trisiloxane polyether surfactant of interest would target rates of trisiloxane‐317 alone, without representing other ingredients within a blend or mixture. Therefore, products reported as using this material neat were identified to further filter the data with all mixtures removed. Because neat products could be identified in the CDPR PUR database for trisiloxane‐OH or trisiloxane‐acetoxy, the 90th percentile of the application rates was used to conservatively estimate “neat material” maximums for these two trisiloxane polyether surfactants (Lee et al., 2019). The query definitions and resulting queried data are presented in the Supporting Information. Peak application rates from the queried data set were then used as BeeREX exposure inputs. The total number of PUR database entries applicable in this project was 61 143, with 112 identified as outliers (0.18%) and 152 identified as likely data errors (0.25%).

In addition to data from the CDPR PUR database, data from an almond orchard residue study for the three trisiloxane polyether surfactants in honey bee‐associated matrices conducted by Syntech Research Laboratory Services were used to characterize exposure. The present study was conducted from February 2018 through March 2019 at an almond orchard located in Sanger, California.

One untreated control plot (plot 1) and three treated plots (plots 2 through 4, one plot for application of each of the three trisiloxane polyether surfactants) were used. The control plot consisted of one subplot, and each of the treatment plots consisted of three subplots. Each subplot consisted of a netted tunnel containing two rows of almond trees (20 trees/row) measuring 32 × 12 m (384 m^2^) with a height of approximately 6 m. The control plot was located at a minimum distance of 18 m from the treated plots, and a 4.5‐m buffer was established between each of the treated plots. Tunnel frames were covered with insect‐proof mesh netting (4–5 mm).

Honey bee colonies selected for use in our study met the following criteria: adequately fed, healthy, and queen‐right, with approximately 6000–10 000 honey bees, approximately two frames of honey, 0.5 frames of pollen, and all stages of brood present. Colonies had previously been treated with both oxalic acid and Fumagilin‐B for mite treatment but had not been chemically treated in the 4 weeks prior to study initiation. Queens were all less than 1 year of age at initiation, and hives had been determined to be healthy and visibly disease free. A single honey bee colony was randomly assigned to each tunnel, placed at the approximate middle of the southern edge of the designated tunnel, and covered during test material application. Hives were set up in each of the tunnels 2 days prior to the first application and maintained within the tunnels from the first application (0 days after first application [DAA]) through 11 DAA (end of exposure phase). The second application was conducted on 5 DAA. Following the exposure phase, the colonies were moved out of the almond orchard portion of the field location and moved to designated locations separated by a minimum of 201 m. During the postexposure sampling phase, colonies were provided sugar syrup and supplemental protein as needed, allowed to forage freely and treated for mites using typical apicultural practices, as needed.

Two applications of each of the trisiloxane polyether surfactants, at the highest label rate for these materials on commercial labels, were made to almond trees at full bloom (Brungardt, 2020). Weather criteria for applications were met as follows: less than 1 mph wind speed and no precipitation within 24 h of application. For trisiloxane‐317, the first and second applications were made at rates of 1.02 and 1.05 kg/ha, respectively (total application = 2.07 kg/ha). For trisiloxane‐OH, the first and second applications were made at rates of 0.189 and 0.191 kg/ha, respectively (total application = 0.380 kg/ha). For trisiloxane‐acetoxy, the first and second applications were made at rates of 0.130 and 0.130 kg/ha, respectively (total application = 0.260 kg/ha). Whole adult bees, larvae, stored nectar, and wax samples were collected at −1, 0, 1, 4, 7, 10, 30, 60, 90, 120, 150, and 180 DAA. Forager pollen, forager nectar, and bee bread samples were collected at 0, 1, 4, 7, and 10 DAA.

Forager pollen was collected using a 10‐frame bottom pollen trap placed on hives the night before sampling events after foraging activity had ceased. All available pollen was collected for the sample and placed in amber glass vials. Forager nectar was collected by blocking hive entrances with tape and capturing returning foraging bees with nets. The bees were transferred to jars containing dry ice and stored frozen until honey stomach processing could be conducted. Stored pollen (bee bread) and stored nectar samples were collected using two dedicated sampling frames that were placed into the hives following application of the test substance. One frame was placed within the center of the brood nest, and one frame was placed just outside the brood nest. A pollen punch extractor was inserted into multiple cells on the sampling frame, and the collected bee bread samples were transferred to amber glass vials. Wide‐bore syringes were used to collect stored nectar samples from cells. Nectar samples were then deposited into a labeled microcentrifuge tube. Whole adult bees were collected from a centrally located brood frame in the hives and stored in capped sample vials while forager bees were in flight. Larvae were collected from the sampling frames using a grafting tool or forceps and placed into amber glass vials. Wax samples were also taken from the same sampling frames. All treatment samples were stored frozen within 4 h of collection. Control samples were frozen within 8 h of collection. Samples were transferred to the analytical facility by freezer truck and maintained under frozen conditions at the laboratory until processing. Freezer temperatures during the in‐field phase ranged from −38 °C to −15 °C, and laboratory frozen temperature was maintained at approximately −18 °C during storage, prior to analysis of samples.

To collect the forager nectar, honey stomachs were removed by thawing the specimen and separating the abdomen from the thorax using forceps while leaving the esophagus exposed. A separate pair of forceps was used solely for the collection of the honey stomach to reduce the likelihood of contamination.

The target minimum sample size for the adult bee and larvae samples was 5.0 g (absolute minimum sample size of 1.0 g). The target minimum sample size for all remaining matrices was 1.0 g (absolute minimum sample size of 100 mg). Triplicate samples were collected and analyzed for each sampling interval. The nectar and honey samples were centrifuged to remove suspended solids prior to analysis. Aliquots (0.05 g) of nectar samples were vortexed for 10 s with 5 ml of acetonitrile:water (1:1) and then diluted 1:3 with acetonitrile:water (1:1) prior to analysis. Aliquots (0.05 g) of pollen and bee bread samples were shaken for 30 min with 5 ml of acetonitrile:water (1:1) and then diluted to 1:3 with acetonitrile:water (1:1) prior to analysis. Aliquots (0.25 g) of bee wax samples were heated for 30 min at 70 °C in a water bath with 25 ml of acetonitrile:water (1:1), and then placed in a freezer for 30 min prior to dilution to 1:3 with acetonitrile:water (1:1) and analysis. Aliquots (0.25 g) of larvae samples were centrifuged with 5 ml of acetonitrile:water (1:1) for 5 min, vortexed for 10 s and then shaken for 30 min prior to dilution to 1:19 with acetonitrile:water (1:1) and analysis. Aliquots (1.0 g) of adult bee samples were homogenized with 50 ml of acetonitrile:water (1:1) for 1 min, centrifuged, and then a 0.125‐ml aliquot was diluted with 0.875 ml of acetonitrile:water (1:1) prior to analysis.

Samples from each matrix were analyzed for residue concentrations of the three trisiloxane polyether surfactants using established liquid chromatography–tandem mass spectrometry. The protocol and final report documents were audited for conformance to USEPA Good Laboratory Practice Standards as defined in 40 CFR Part 160 (USEPA, 1989). Inspection and audit of two critical field phase events, one critical analytical phase event, and raw data were also conducted to ensure quality of the methods, documentation, and results of the present study. Frozen storage stability was verified for all sample matrices with analytical intervals of 0, 60, 120, 180, 240, 300, and 360 days, which covered the storage duration for all samples in our study. In addition, the stability of nectar was verified at approximately 37.8 °C for 0, 10, 30, and 60 days and after three freeze/thaw cycles.

Peak empirical residue data and the application rates for the almond orchard residue study were then used as BeeREX exposure inputs.

### Effects characterization

There are several routes of honey bee exposure to agricultural chemicals (USEPA, PMRA, & CDPR, 2014). Exposure can occur through oral or direct contact in an adult stage honey bee. Foraging honey bees can be exposed through ingestion or contact with water, pollen, nectar, or particles in air. Larval‐stage honey bees are immobile within their comb cells of the hive and partially submerged in their appropriate feeding solution (consisting of royal jelly or nectar and pollen); therefore, dietary and contact exposure occur simultaneously, and laboratory tests simulate this exposure scenario. Separate laboratory studies were conducted for each of the three trisiloxane polyether surfactants, according to validated, globally accepted test methodologies, and following international Good Laboratory Practice standards. Acute contact and oral toxicity laboratory studies were conducted to assess the effects of the three trisiloxane polyether surfactants to adult stage honey bees (Organisation for Economic Co‐operation and Development [OECD] test guidelines 213 [1998a] and 214 [1998b]). In addition, chronic dietary studies were conducted with both adult and larval stage honey bees (OECD test guideline 245 [2017]/EFSA guidance from [2013] and OECD guidance document 239 [2016], respectively). The chronic larval toxicity study (OECD guidance document 239 [2016]) also included an interim evaluation interval that characterized the acute oral toxicity for larval stage honey bees, equivalent to the acute endpoint generated in OECD guidance document 237 (2013). These studies were considered to provide the most appropriate endpoints for use in our risk assessment (Picard, 2019a, 2019b, 2019c; Sesso, 2003a, 2003b; Taylor, 2015; Tome & Porch, 2017a, 2017b, 2019a, 2019b, 2019c). The adult oral LD50 and adult contact LD50 values were used as the acute toxicological input into the BeeREX model. The no‐observed‐effect dose (NOED) was used as the adult and larval chronic toxicological inputs.

The adult acute contact toxicity test was conducted using treatment levels established based on range‐finding preliminary experiments. Three replicates, each consisting of 10 bees, were established for each treatment level. Bees were anesthetized with carbon dioxide, and then a 1.0‐µl aliquot of each test solution was applied to the surface of the dorsal thorax. Following dosing, bees were maintained in test cages for observation of lethal effects after 4, 24, and 48 h. A dimethoate reference solution was also used to confirm the sensitivity of the test species.

The adult chronic toxicity test was conducted using an oral exposure under laboratory conditions for a period of 10 days. Five concentrations of the test substances were used with three replicates (each comprising of 10 bees) maintained for each concentration. Diets were prepared based on dilutions with 50% sucrose solutions, similar to the acute oral exposure just described. A negative control (50% sucrose solution) was maintained, and a positive control (dimethoate) was used to confirm the sensitivity of the test species. Syringe feeders were provided and replenished at 24‐h intervals. Evaporative losses and feed consumption were measured. All bees were observed on a daily basis for lethal and sublethal effects. Because the same laboratory conducted all three studies, the control data were pooled for all three exposures (one for each of the three trisiloxanes). The pooled control data were used for statistical comparison.

The larval chronic toxicity test was conducted using a 22‐day exposure of larvae to treated diet, initiated on day 3 and continued through day 6, resulting in both dermal and oral exposure until pupation (day 7 or 8). Test organisms were allowed to complete development through adult emergence. Therefore, this test design proved an 8‐day larval and 22‐day pupal and adult emergence exposure. Cumulative 4‐day doses were used to determine study endpoints. Isolated brood cells were grafted from hive frames using a grafting tool that had been sanitized with 70% ethyl alcohol to reduce potential for contamination. Larvae were acclimated to laboratory conditions for 2 days. Diet consisted of three specific combinations of deionized water, d‐glucose, d‐fructose, yeast extract, and royal jelly. Ratios of each ingredient were based on the age of the larvae. The health of the larvae was observed and recorded daily. Stock solutions were prepared in acetone as a solvent carrier, and thus a solvent control group was established.

The adult acute oral toxicity test was conducted using treatment levels established based on range‐finding preliminary experiments. Three replicates, each consisting of 10 bees, were established for each treatment level. Treatment diets were prepared by dissolving aliquots of the test material with 50% sucrose solution, and the treated or control diet was fed to the bees during a 4‐h period. Following the 4‐h treatment period, the treated diet was replaced with 50% sucrose solution, and the bees were evaluated for lethal effects after 24 and 48 h. A dimethoate reference solution was also used to confirm the sensitivity of the test system.

The LD50 values for the adult acute contact and oral studies for trisiloxane‐317 were determined using the trimmed Spearman–Karber statistical method (Hamilton et al., 1978). This method also determined the 95% confidence limits for these data. The LD50 values for the adult acute contact and oral studies for trisiloxane‐OH and trisiloxane‐acetoxy were all empirically estimated to be greater than the highest dose tested because no treatment level tested resulted in 50% or more lethality during these laboratory exposures. The adult chronic dietary NOED values for all three trisiloxanes were determined using Williams' multiple comparison statistical test (Williams, 1971, 1972). The pooled control data (from the control groups of all three exposures) were used for statistical comparison with the treatment data using CETIS Ver 1.9 (Ives, 2019). The larval acute LD50 values for all three trisiloxanes were empirically estimated to be greater than the highest dose tested because no treatment level tested resulted in 50% of more lethality during these laboratory exposures. The larval chronic NOED for trisiloxane‐OH was determined using the Cochrane–Armitage step‐down statistical test (Armitage, 1955; Cochran, 1954) based on the monotonicity of the data. Because we used a solvent control, the negative and solvent control data were first compared and because no significant difference was observed, the negative control group was used to evaluate treatment performance. The larval chronic NOED for trisiloxane‐317 and trisiloxane‐acetoxy were determined using Fisher's exact test with Bonferroni–Holmes adjustment statistical test (Fisher, 1974) based on the nonmonotonicity of the data. The treatment data for all larval chronic studies were compared with the negative control data in CETIS Ver 1.9 (Ives, 2019).

### Risk characterization

Risk quotients for honey bees potentially exposed to the three trisiloxane polyether surfactants used in the field study pesticide applications were calculated using the BeeREX model, wherein the RQs are derived from the estimates of exposure and toxicity. Risk quotients were determined based on the model‐EEC and applicable toxicity endpoints when empirical residue data are not used as inputs. When empirical residue concentrations are inserted into the model, they are used to calculate exposure, which avoids the BeeREX default residue/unit dose (RUD) value of 110 ppm for both nectar and pollen as follows:

(1) AcuteRQ=EEC/AcuteLD50

(2) ChronicRQ=EEC/Chronic NOED

The EEC is calculated as follows:

(3) EEC=RUD×Application rate

The RUD is defined as the residue concentration in ppm/lb a.i. applied/acre.

BeeREX was run in two ways: 1) with the maximum CDPR PUR values as application rates plus the BeeREX model default RUD assumptions and resulting EEC calculation (there were no empirical residue concentration data applied); and (2) with the almond orchard study application rates plus the maximum measured (empirical) nectar and pollen residue concentrations determined during the field study. The default RUD values for nectar and pollen in the BeeREX model are the upper‐bound pesticide residue concentrations determined for tall grasses as reported in the Kenaga nomogram (Hoerger & Kenaga, 1972) as modified by Fletcher et al. (1994). The default RUD value is 110 ppm for both nectar and pollen, and this value is assumed to be a suitable surrogate for both matrices. Calculated RQ values were then compared with LOC values specified in the risk assessment guidance (0.4 for acute risk and 1.0 for chronic risk) for screening‐level risk pollinator risk assessment (USEPA, PMRA, & CDPR, 2014).

## RESULTS

The LOQs and minimum detectable limits for each of the analytes and matrices from the almond orchard residue study are presented in Table 1. Residue concentrations of the three trisiloxane polyether surfactants measured in bee‐relevant matrices from the almond orchard residue study are provided in Table 2. The highest residue value measured in each matrix (Table 3) was used for BeeREX inputs. The peak forager pollen residue values from all sampling intervals occurred at the day 0–1 sampling interval for all three trisiloxane polyether surfactants; peak forager pollen residue concentrations were greater than peak bee bread residue concentrations, and peak residue concentrations in forager nectar were greater than in stored nectar.

**Table 1:** Summary of limits of quantitation (LOQs) and average total minimum detectable limits (MDLs) for residue analysis from honey bee–relevant matrices from almond orchard residue study

	Trisiloxane‐OH (mg/kg)		Trisiloxane‐acetoxy (mg/kg)		Trisiloxane 317 (mg/kg)	
Matrix	LOQ	Average total MDL	LOQ	Average total MDL	LOQ	Average total MDL
Pollen	0.100	0.0152	0.100	0.0151	0.400	0.0713
Nectar	0.100	0.0143	0.100	0.0119	0.400	0.0634
Bee bread	0.100	0.0100	0.100	0.0072	0.400	0.0954
Larvae	0.100	0.0207	0.100	0.0138	0.400	0.0666
Bee wax	0.100	0.0148	0.100	0.0109	0.320	0.0688
Whole adult bees	0.100	0.0168	0.100	0.0131	0.400	0.0509

**Table 2:** Summary of residues for honey bee–relevant matrices from almond orchard residue study

	Trisiloxane‐317 Plot 2			Trisiloxane‐OH Plot 3			Trisiloxane‐acetoxy Plot 4		
	Application 1: 1.02 kg/ha Application 2: 1.05 kg/ha			Application 1: 0.189 kg/ha Application 2: 0.191 kg/ha			Application 1: 0.130 kg/ha Application 2: 0.130 kg/ha		
	Total: 2.06 kg/ha			Total: 0.380 kg/ha			Total: 0.260 kg/ha		
	Min	Max	Peak day	Min	Max	Peak day	Min	Max	Peak day
Matrix	(mg/kg)	(mg/kg)	(DA2A)	(mg/kg)	(mg/kg)	(DA2A)	(mg/kg)	(mg/kg)	(DA2A)
Forager pollen	6.77	76.2	1	0.163	26.5	0	0.112	4.53	0
Forager nectar	<0.0634	2.12	10	0.0213	0.48	7	<0.0119	—	NA
Stored nectar	<0.0634	0.177	7	<0.0143	0.0662	10	<0.0119	—	NA
Bee bread	<0.0954	40.5	7	2.99	17.3	1	0.545	2.65	1
Larvae	<0.0666	0.768	7	<0.0207	0.147	10	<0.0138	—	NA
Wax	<0.0688	12.1	7	<0.0148	1.22	7	<0.0109	0.332	10
Whole bees	<0.0509	8.32	7	<0.0168	2.45	7	<0.0131	—a	—a

^a^ The data for this matrix are invalid due to instability during frozen storage.

Residues from plot 1 (untreated control plot) were all below the minimum detectable limit.

Values have been rounded to three significant figures. Total application rates were calculated using unrounded values. <, values presented in the table indicate analytical results below the minimum detectable limit for each matrix and analyte. —, indicates no maximum residue because all samples were below the minimum detectable limit.

DA2A = days after second application; NA = not applicable (because all measured residues were below the minimum detectable limit).

**Table 3:** BeeREX exposure inputs based on almond orchard residue study and California Department of Pesticide Regulation (CDPR) Pesticide Use Registry (PUR) database

Input description	Trisiloxane‐317	Trisiloxane‐OH	Trisiloxane‐acetoxy
Application rate (kg/ha) from CDPR PUR database	1.42	0.404	0.688
Application rate (kg/ha) from the almond orchard residue study^a^	2.06	0.380	0.260
Application method	Foliar spray	Foliar spray	Foliar spray
Empirical residue in pollen/bread (mg/kg)	76.2^b^	26.5^b^	4.53^b^
Empirical residue in nectar (mg/kg)	2.12^c^	0.48^c^	0.1^e^
Empirical residue in jelly (mg/kg)	0.768^d^	0.15^d^	0.1^e^

^a^ Based on the actual application rates in the almond orchard residue study.

^b^ Based on the peak residue value for all pollen samples (forager pollen and bee bread) from all sampling intervals (peak value occurred in day 0 forager pollen for all analytes.

^c^ Based on the peak residue value for all nectar samples (forager and stored nectar) from all sampling intervals (peak value occurred in forager nectar for all samples).

^d^ Based on peak larvae value from all sampling intervals.

^e^ The matrix limit of quantitation (LOQ) was used as a conservative estimate because all residues were below the LOQ.

The default exposure values in BeeREX are applied based on estimated pesticide residues on tall grass (application rates using the Kenaga nomogram from T‐REX, which is incorporated into BeeREX (US Environmental Protection Agency [USEPA] et al. (2014). The USEPA considers estimated residues on tall grasses to be a suitable surrogate for residues in pollen and nectar of flowers that are directly sprayed.

The total amount of trisiloxane‐317 applied (two applications) during the almond orchard residue study was 2.056 kg/ha, greater than the maximum application rate derived from the CDPR PUR database query following removal of outliers and values determined to be likely entry errors, which was 1.42 kg/ha. However, the individual application rates for trisiloxane‐317 in the almond orchard residue study were both below the CDPR PUR value. The total amount of trisiloxane‐OH applied during the almond orchard residue study (0.380 kg/ha) was below the maximum application rates from the CDPR PUR database query (0.404 kg/ha). The total amount of trisiloxane‐acetoxy applied during the almond orchard residue study (0.260 kg/ha) was below the maximum application rate derived from the CDPR PUR database query (0.688 kg/ha).

The effects endpoints determined from laboratory testing are summarized in Table 4. Tables 5 and 6 present the results of the RQ calculations using both the CDPR queries and the almond orchard residue results. In both cases, utilizing agricultural use data from the CDPR database as well as the empirical residue data for almond tree applications, all RQ values were below the acute (0.4) and chronic (1.0) LOCs.

**Table 4:** Toxicity values selected for use in the risk assessment

		Measurement endpoints (µg/bee or µg/bee/day)		
Honey bee life stage	Exposure duration and route	Trisiloxane‐317	Trisiloxane‐OH	Trisiloxane‐acetoxy
Adult	Acute contact (LD50)	43.67^a^	100^b^	150^b^
	Acute oral (LD50)	285.16^c^	100^b^	304^b^
	Chronic dietary (NOAEL)	85.9^d^	12.8^d^	94^d^
Larvae	Acute^e^ (LD50)	100^b^	100^b^	100^b^
	Chronic (NOAEL)	33^f^	40^g^	33^f^

^a^ Determined using trimmed Spearman–Karber statistical method. The 95% confidence limits were determined to be 37.30–51.13 µg/bee.

^b^ The median lethal dose (LD50) values were empirically estimated to be greater than the value presented in the table because none of the dose rates in the laboratory studies resulted in 50% inhibition of the measured parameter. However, as a conservative estimate, the absolute value of the endpoint is used to determine the risk quotients in the BeeREX model.

^c^ Determined using trimmed Spearman–Karber statistical method. The 95% confidence limits were determined to be 247.84–328.10 µg/bee.

^d^ Determined using Williams' multiple comparison statistical test, comparing treatment data with the pooled control data in CETIS Ver 1.9.

^e^ The acute larval endpoints were taken from the 3–8‐day survival endpoint from the larval chronic exposure studies.

^f^ Determined using Fisher's exact test with Bonferroni–Holmes adjustment statistical test based on nonmonotonicity of the data. The treatment data were compared with the negative control data in CETIS Ver 1.9.

^g^ Determined using Cochrane–Armitage step‐down statistical test based on monotonicity of the data. The treatment data were compared with the negative control data in CETIS Ver 1.9.

Acute endpoints are reported in units of µg/bee, and chronic endpoints are reported in units of µg/bee/day.

LD50 = median lethal dose; NOAEL = no‐observed‐adverse‐effects level.

**Table 5:** Tier 1 risk quotients based on application rates from the California Department of Pesticide Regulation Pesticide Use Registrydatabase and BeeREX default estimated environmental concentrations

	Trisiloxane‐317		Trisiloxane‐OH		Trisiloxane‐acetoxy	
Exposure	Adults	Larvae	Adults	Larvae	Adults	Larvae
Acute contact	0.079	NA	0.010	NA	0.011	NA
Acute dietary	0.14	0.17	0.12	0.05	0.065	0.084
Chronic dietary	0.47	0.52	0.90	0.12	0.21	0.21
Summary result	No acute or chronic risk indicated (based on comparison with the 0.4 acute level of concern established for regulatory risk assessment)					

NA, not applicable per US Environmental Protection Agency (2014).

**Table 6:** Tier 1 risk quotients based on exposure inputs from the almond orchard residue study

	Trisiloxane‐317		Trisiloxane‐OH		Trisiloxane‐acetoxy	
Exposure	Adults	Larvae	Adults	Larvae	Adults	Larvae
Acute contact	0.113	NA	0.0092	NA	0.0042	NA
Acute dietary	0.004	0.005	<0.001	<0.001	0.0002	0.0003
Chronic dietary	0.012	0.016	0.03	<0.001	0.0006	0.0007
Summary result	No acute or chronic risk indicated (based on comparison to the 1.0 chronic level of concern established for regulatory risk assessment)					

NA, not applicable per US Environmental Protection Agency et al. (2014).

## DISCUSSION

We conducted a screening‐level pollinator risk assessment with the aim of assessing the potential risk of trisiloxane polyether surfactants to pollinators posed by agricultural uses as coformulants to pesticide formulations. Some studies from the published literature have indicated potential toxic effects of trisiloxane polyether surfactants on honey bees and other insect species (Acheampong & Stark, 2004; Mullin et al., 2015, 2016). Wernecke et al. (2021) investigated direct‐contact pollinator mortality based on exposure to adjuvants, including trisiloxane polyether surfactants. The results showed that no significant mortality resulted from exposure to the trisiloxane polyether surfactants relative to the water control. In addition to the published literature, laboratory guideline studies were performed for the three trisiloxane polyether surfactants, deriving toxicity endpoints indicating that bee mortality only occurs at concentrations that exceed the application rates of pesticide formulations containing any of the three substances. Furthermore, application of the pesticide screening‐level risk assessment process to nonpesticide formulation constituents was incorporated to further characterize risk, which represents a novel methodology.

The toxicological pathway to insecticidal activity of trisiloxane polyether surfactants was investigated by Cowles et al. (2000) based on known miticidal properties to the two spotted spider mite (*Tetranychus urticae*). They proposed that trisiloxane polyether surfactants can display insecticidal properties through multiple pathways, the first of which is similar to detergent compounds that allow water to permeate respiratory structures leading to drowning. Furthermore, physicochemical toxicity can occur through interaction of the trisiloxane polyether surfactants with biological membranes and interference with cellular signaling processes (Alberti & Crooker, 1985; Cowles et al., 2000; Imai et al., 1994). Although the potential toxicity of trisiloxane polyether surfactants to honey bees has been incorporated in studies in the published literature based on mortality and sublethal effects (i.e., olfactory learning impairment; Ciarlo et al., 2012), the specific toxicological pathway for bees has not been characterized. Although the study published by Ciarlo et al. (2012) has been referenced in several other studies in the past decade, challenges to the Ciarlo et al. methods have been cited (Anderson, 2018). These challenges include a lack of exposure quantification, sampling and analysis of nectar, and experimental design uncertainty based on the proboscis extension reflect assay used in the Ciarlo et al. (2012) study.

The toxicological profile of trisiloxane polyether surfactants alone is not sufficient for characterizing and quantifying potential adverse effects to pollinators. Routes of exposure must also be considered. Because trisiloxane polyether surfactants are used in conjunction with spray applications of pesticides, the routes of exposure can be assessed in the same manner as pesticide‐active ingredients. Because honey bees forage in and around treated crop areas, foraging bees can be exposed to pesticides (and other spray tank surfactants and adjuvants) through oral uptake and contact exposures (USEPA, PMRA, & CDPR, 2014). Contact exposure can occur when a bee is foraging in a treatment area at time of application via direct spray. Oral exposure can occur due to the consumption of nectar and pollen by adult bees and larvae in the hive (Rortais et al., 2005, 2017). Exposures to individuals are based on activity and consumption rates dependent on developmental stage. The BeeREX model takes each of these exposure routes into account in a conservative manner, and our empirical residue inputs are used to refine the exposure characterization. Moreover, the exposure to foraging bees depends on the degree of crop‐attractiveness as well as the timing of applications with respect to bloom and peak foraging times of honey bees (Sanchez‐Bayo & Goka, 2016).

Mullins et al. (2015, 2016) cite an increase in the use of trisiloxane polyether surfactants over the course of the previous decade prior to their publication and discuss this increase in use as a cause for concern regarding exposure of these materials to honeybees. However, it should be noted that the cited increase in use is based on the CDPR PUR database reporting. The history of the database and reporting requirements must be considered when assessing the proposed increase in product use. At the inception of the database, no adjuvant materials were included or reported. The database was expanded to include use reporting for adjuvants but only optionally, and then, more recently, reporting adjuvant uses became a requirement for the state of California. Therefore, the increase in cited uses since approximtely 2007 is really a function of reporting rather than an increase in product use.

Our risk assessment was intended to be highly conservative, to improve the certainty that further higher tier assessment is not required to adequately characterize risk. It is important to consider the multiple assumptions built into the screening‐level assessment, to put the risk conclusions into context. The acute laboratory effects studies, in most cases, generated LD50 values that were empirically greater than the highest dose level tested. Use of the highest dose value as the toxicological endpoint (e.g., use of 100 µg/bee as an LD50 input into BeeREX when the present study indicated an LD50 value of more than 100 µg/bee) adds conservatism to our assessment.

When applying CDPR PUR 2017 data, we used the “neat materials” maximum application rate or the “mixtures” 90th percentile of the application rates for all crops for all recorded instances (with the exception of mentioned outliers). These values were similar to the application rates used in the almond orchard residue study, which were based on maximum label rates, and should be considered highest likely real‐world application rates. The use of these rates represents another level of conservatism applied to the screening‐level assessment. In addition, from an exposure perspective, the inputs into BeeREX for this screening‐level assessment assume that honey bees are foraging and provisioning the hive exclusively from treated crops. This assumption is applicable in the almond orchard study for the 3‐day period during which the hives were enclosed in the netted tunnels. Degradation and dissipation rates are also not included in the exposure characterization, adding further to the conservative nature of our assessment. The BeeREX model determines dietary exposure values for honey bee larvae of differing developmental stages and adults for the various castes, including the queen. Contact exposure is also estimated with which to generate an RQ for an adult acute contact exposure. The exposure values estimated in BeeREX are determined based on the product application type and rate and conservatively estimated pollen and nectar consumption rates for the different honey bee castes. According to the 2014 guidance document (USEPA, PMRA, & CDPR, 2014), “the (tier 1) model‐generated exposure estimates, while intended to represent environmentally relevant exposure levels, are nonetheless considered high‐end estimates.” As a screening‐level tool, the model incorporates levels of conservativism in the RQ calculations such that the derived RQ values can be considered highest likely real‐world estimates of risk. The use rates and toxicological profile of various trisiloxane polyether surfactant can vary, so the results of the screening‐level risk assessment in our study may not be completely representative of all surfactants within this class. Our results provide a useful method by which the screening‐level risk assessment framework can be applied to this class of surfactant materials in furtherance of on‐going research efforts to identify potential adverse effects on honey bees and other insect pollinators.

Our study provides insight into the risk of trisiloxane polyether surfactants to pollinators; however, some limitations exist regarding this approach that could be considered for future research. First, the toxicological inputs in the screening‐level risk assessment focus on mortality‐based endpoints and do not take sublethal effects into account. Foraging efficiency, flight time, and learning capacity, for example, represent sublethal parameters that have been investigated in pollinator research. However, sublethal effects are not incorporated into the standard bee risk assessments. Furthermore, our study examines direct exposure and effects from the surfactant materials alone and does not provide insight into mixture effects or potential synergistic effects of tank‐mix additives and pesticide products. Wernecke et al. (2021) examine the synergistic effects of adjuvants, including trisiloxane polyether surfactants and insecticide. Their research indicated higher mortality in approximately 50% of adjuvant and insecticide combinations under acute direct‐contact study designs. Further investigation is required to characterize these interactions and potential synergistic effects.

Although recent research indicates an increased interest in the potential adverse effects of adjuvants to pollinators, to date a formal regulatory framework with supporting methodologies to characterize risk has not been published. Our research applied closely examined exposure data to the BeeREX model to provide a refined screening‐level assessment that can serve as a model for future research. This approach followed the standardized pollinator risk assessment framework (tier 1; Figure 1) in which the calculated RQ values were compared with the established acute and chronic LOCs (0.4 and 1.0, respectively). Our assessment concluded that minimal risk to pollinators will result from the labeled use of three trisiloxane polyether surfactants (trisiloxane‐317, trisiloxane‐OH, and trisiloxane‐acetoxy), and we suggest that higher tier assessments on this topic are unnecessary.

## Supporting Information

The Supporting Information is available on the Wiley Online Library at https://doi.org/10.1002/etc.5479.

## Author Contributions Statement

**Jennifer K. Collins**: Conceptualization; Data curation; Formal analysis; Investigation; Methodology; Project administration; Resources; Supervision; Visualization; Writing—original draft. **Jennifer M. Jackson**: Data curation; Project administration; Resources; Writing—review & editing.

## Supporting information

This article includes online‐only Supporting Information.

Supporting information.

## Data Availability

The supporting Pesticide Use Registry database is available in the Supporting Information. The BeeREX screening‐level model, Ver 1.0, and User Guidance Document is publicly available for download through the US Environmental Protection Agency (https://www.epa.gov/pesticide-science-and-assessing-pesticide-risks/models-pesticide-risk-assessment). Copies of the unpublished industry study reports may be requested from the Silicones Environmental, Health, and Safety Center by contacting tracy_guerrero@americanchemistry.com. Data, associated metadata, and calculation tools are also available from the corresponding author (collinsj@waterborne-env.com).
