# Supplementary material for: Application of a Screening‐Level Pollinator Risk Assessment Framework to Trisiloxane Polyether Surfactants
Source: Environ Toxicol Chem. 2022 Oct 20;41(12):3084–94. doi: 10.1002/etc.5479 (PMC9828746; doi:10.1002/etc.5479)
Supplement: Supplementary file 1 — Supporting information. [file ETC-41-3084-s001.docx]

**Supplemental Material**

CDPUR Database Query Search Terms and Results

CAS RN Query Definitions from CDPR PUR Database

| **Query** | **CAS RN** | **Chemical Name**  **(DPR Common Name)** | **Year** | **Number of**  **Active Product Labels**  **in 2017** | **Use Records in 2017** |
| --- | --- | --- | --- | --- | --- |
| Query A | 67674-67-3 | heptamethyltrisiloxane ethoxylated | 2017 | 9 | 6599 |
| Query B | 134180-76-0 | heptamethyltrisiloxane-1,3-propanediol ether, ethoxylated propoxylated | 2017 | 20 | 11879 |
| Query C | 134180-76-0 | methyl silicone resins | 2017 | 11 | 14072 |
| Query D | 125997-17-3 | 2-(3-hydroxypropyl)-heptamethyl trisiloxane, ethoxylated, acetate | 2017 | 2 | 28593 |

NOTE: Use records represent individual application records

Chemical Name Query from CDPR PUR Database

| **Chemical Name** | **Chemical Code** | **Number of**  **Active Product**  **Labels**  **in 2017** | **Use Records in 2017** |
| --- | --- | --- | --- |
| dimethylpolysiloxane | 1861 | 80 | 138979 |
| polyether modified polysiloxane | 5223 | 13 | 33031 |
| polyalkene oxide modified heptamethyl trisiloxane | 3520 | 12 | 36718 |
| organo/modified polysiloxane | 3997 | 1 | 1291 |
| oxyethylene methyl siloxane | 2730 | 0 | 0 |
| phenyl trimethyl siloxane | 3728 | 0 | 0 |
| polysiloxane | 5718 | 5 | 1173 |
| silica filled polydimethylsiloxane | 6081 | 11 | 1315 |
| compounded silicone | 2124 | 0 | 0 |
| dimethyl silicone fluid emulsion | 2028 | 1 | 17641 |
| organosilicone, polyoxyalkylene ether copolymer | 5231 | 6 | 2680 |
| silicone | 3796 | 3 | 234 |
| silicone defoamer | 1917 | 2 | 11217 |
| polyalkyleneoxide modified polydimethylsiloxane | 5203 | 12 | 3205 |
| silicone mold release agent SM 2140 | 2854 | 0 | 0 |

NOTE: Use records represent individual application records

**CDPR PUR Database Queries with Post-Processing for Trisiloxane-317**

| **Crop** | **90^th^ Percentile Values Application Rate (lb/A)** | | | | |
| --- | --- | --- | --- | --- | --- |
|  | **Redacted 1** | **Redacted 2** | **Redacted3** | **Redacted4** | **Redacted5** |
| Alfalfa |  |  | 0.1201 | 0.0460 | 0.0391 |
| Almond | 0.3395 |  | 0.3748 | 1.0448 | 1.2540 |
| Apple |  |  |  |  | 0.1670 |
| Avocado |  |  | 0.2023 |  |  |
| Beans |  |  | 0.0526 |  |  |
| Blackberry |  |  | 0.5353 | 0.3265 | 0.1267 |
| Blueberry |  |  |  | 1.2696 |  |
| Cantaloupe |  |  | 0.0075 |  |  |
| Carrots |  |  | 0.0095 |  |  |
| Cauliflower |  |  | 0.0332 |  |  |
| Celery |  |  | 0.5324 |  |  |
| Cherry | 0.1415 |  |  |  | 0.6522 |
| Corn |  |  | 0.1335 |  | 0.0262 |
| Endive |  |  | 0.0998 |  |  |
| Garbanzos |  |  | 0.0278 |  |  |
| Garlic |  |  | 0.1331 |  |  |
| Grapes | 0.1358 |  | 0.8518 |  |  |
| Grapes, Wine | 0.0849 | 1.057 | 1.2105 | 0.3931 | 0.3840 |
| Kale |  |  | 0.0998 |  |  |
| Lettuce |  |  | 0.1314 |  |  |
| Melons |  |  | 0.0076 |  |  |
| Onions |  |  | 0.0284 | 0.0789 |  |
| Orange |  |  | 0.1746 | 1.0480 | 0.9185 |
| Peach | 0.2653 | 0.0160 |  | 0.1299 |  |
| Persimmon | 0.1061 |  |  |  |  |
| Pistachio | 0.1363 |  | 0.2147 | 0.2593 | 1.2612 |
| Raspberry |  |  | 0.5537 | 0.3267 | 1.0805 |
| Strawberry |  |  | 1.0739 |  | 0.2618 |
| Sugarbeet |  |  | 0.0078 |  |  |
| Tangerine |  |  | 1.1867 |  | 1.0471 |
| Tomatoes |  |  | 0.0284 | 0.0843 |  |
| Walnut | 0.4244 | 0.2087 | 0.5335 | 0.5263 | 0.4174 |
| Wheat | 0.0637 |  |  |  |  |
| **Overall Peak** | **0.4244** | **1.0570** | **1.2105** | **1.2696** | **1.2612** |

**CDPR PUR Database Queries with Post-Processing for Trisiloxane-OH**

| YEAR | DATE | COUNTY_NAME | SITE_NAME | PRODUCT NAME | POUNDS_PRODUCT_APPLIED | AMOUNT_  TREATED | UNIT_  TREATED | AERIAL_GROUND_INDICATOR | Application Rate (lb/A) |
| --- | --- | --- | --- | --- | --- | --- | --- | --- | --- |
| 2017 | 3-Aug-17 | IMPERIAL | ALFALFA | Redacted1 | 8.2051 | 21 | A | A | 0.3595 |
| 2017 | 23-Feb-17 | IMPERIAL | ALFALFA | Redacted1 | 44.6355 | 115 | A | A | 0.3571 |
| 2017 | 23-Feb-17 | IMPERIAL | ALFALFA | Redacted1 | 31.0151 | 80 | A | A | 0.3567 |
| 2017 | 24-Feb-17 | IMPERIAL | ALFALFA | Redacted1 | 31.0151 | 80 | A | A | 0.3567 |
| 2017 | 10-Feb-17 | IMPERIAL | ALFALFA | Redacted1 | 13.3742 | 65 | A | A | 0.1893 |
| 2017 | 24-Jun-17 | MERCED | ALFALFA | Redacted1 | 13.3742 | 65 | A | G | 0.1893 |
| 2017 | 10-Feb-17 | IMPERIAL | ALFALFA | Redacted1 | 12.3076 | 60 | A | A | 0.1887 |
| 2017 | 28-Jul-17 | TULARE | ALFALFA | Redacted1 | 16.4101 | 90 | A | A | 0.1677 |
| 2017 | 13-Mar-17 | FRESNO | ALMOND | Redacted1 | 23.8459 | 60 | A | G | 0.3656 |
| 2017 | 21-Apr-17 | TEHAMA | ALMOND | Redacted1 | 1.3128 | 4 | A | G | 0.3019 |
| 2017 | 21-Apr-17 | TEHAMA | ALMOND | Redacted1 | 1.641 | 5 | A | G | 0.3019 |
| 2017 | 21-Apr-17 | TEHAMA | ALMOND | Redacted1 | 1.641 | 5 | A | G | 0.3019 |
| 2017 | 1-Jul-17 | FRESNO | ALMOND | Redacted1 | 20.5126 | 64 | A | G | 0.2949 |
| 2017 | 24-Feb-17 | FRESNO | ALMOND | Redacted1 | 29.4561 | 92 | A | A | 0.2946 |
| 2017 | 24-Feb-17 | FRESNO | ALMOND | Redacted1 | 25.5998 | 80 | A | A | 0.2944 |
| 2017 | 21-Apr-17 | TEHAMA | ALMOND | Redacted1 | 2.5436 | 8 | A | G | 0.2925 |
| 2017 | 21-Apr-17 | TEHAMA | ALMOND | Redacted1 | 2.2154 | 7 | A | G | 0.2912 |
| 2017 | 21-Apr-17 | TEHAMA | ALMOND | Redacted1 | 1.8872 | 6 | A | G | 0.2894 |
| 2017 | 15-Jul-17 | FRESNO | ALMOND | Redacted1 | 4.1025 | 18 | A | G | 0.2097 |
| 2017 | 19-Jun-17 | FRESNO | ALMOND | Redacted1 | 4.1025 | 18 | A | G | 0.2097 |
| 2017 | 18-Mar-17 | MADERA | ALMOND | Redacted1 | 15.4255 | 75 | A | G | 0.1892 |
| 2017 | 18-Mar-17 | MADERA | ALMOND | Redacted1 | 15.4255 | 75 | A | G | 0.1892 |
| 2017 | 18-Feb-17 | KINGS | ALMOND | Redacted1 | 8.1886 | 40 | A | A | 0.1883 |
| 2017 | 18-Feb-17 | KINGS | ALMOND | Redacted1 | 9.6163 | 47 | A | A | 0.1882 |
| 2017 | 28-Feb-17 | FRESNO | ALMOND | Redacted1 | 14.8511 | 103.66 | A | G | 0.1318 |
| 2017 | 7-Jul-17 | TEHAMA | ALMOND | Redacted1 | 0.4103 | 5 | A | G | 0.0755 |
| 2017 | 10-Mar-17 | BUTTE | ALMOND | Redacted1 | 6.564 | 80 | A | A | 0.0755 |
| 2017 | 28-Feb-17 | KINGS | ALMOND | Redacted1 | 1.9692 | 47 | A | A | 0.0385 |
| 2017 | 28-Feb-17 | KERN | ALMOND | Redacted1 | 2.5436 | 61 | A | A | 0.0384 |
| 2017 | 28-Feb-17 | KERN | ALMOND | Redacted1 | 1.559 | 38 | A | A | 0.0377 |
| 2017 | 27-May-17 | FRESNO | GRAPES | Redacted1 | 2.0513 | 10 | A | G | 0.1887 |
| 2017 | 31-May-17 | FRESNO | GRAPES | Redacted1 | 2.0513 | 10 | A | G | 0.1887 |
| 2017 | 31-May-17 | FRESNO | GRAPES | Redacted1 | 2.0513 | 10 | A | G | 0.1887 |
| 2017 | 30-May-17 | FRESNO | GRAPES | Redacted1 | 2.0513 | 10 | A | G | 0.1887 |
| 2017 | 31-May-17 | FRESNO | GRAPES | Redacted1 | 5.1282 | 25 | A | G | 0.1887 |
| 2017 | 31-May-17 | FRESNO | GRAPES | Redacted1 | 3.6923 | 18 | A | G | 0.1887 |
| 2017 | 31-May-17 | FRESNO | GRAPES | Redacted1 | 3.6923 | 18 | A | G | 0.1887 |
| 2017 | 30-May-17 | FRESNO | GRAPES | Redacted1 | 5.9487 | 29 | A | G | 0.1887 |
| 2017 | 31-May-17 | FRESNO | GRAPES | Redacted1 | 8.2051 | 40 | A | G | 0.1887 |
| 2017 | 30-May-17 | FRESNO | GRAPES | Redacted1 | 8.2051 | 40 | A | G | 0.1887 |
| 2017 | 2-Jun-17 | FRESNO | GRAPES | Redacted1 | 8.2051 | 40 | A | G | 0.1887 |
| 2017 | 31-May-17 | FRESNO | GRAPES | Redacted1 | 8.2051 | 40 | A | G | 0.1887 |
| 2017 | 31-May-17 | FRESNO | GRAPES | Redacted1 | 8.2051 | 40 | A | G | 0.1887 |
| 2017 | 29-May-17 | FRESNO | GRAPES | Redacted1 | 8.2051 | 40 | A | G | 0.1887 |
| 2017 | 30-May-17 | FRESNO | GRAPES | Redacted1 | 8.2051 | 40 | A | G | 0.1887 |
| 2017 | 30-May-17 | FRESNO | GRAPES | Redacted1 | 8.2051 | 40 | A | G | 0.1887 |
| 2017 | 29-May-17 | FRESNO | GRAPES | Redacted1 | 8.2051 | 40 | A | G | 0.1887 |
| 2017 | 30-May-17 | FRESNO | GRAPES | Redacted1 | 8.2051 | 40 | A | G | 0.1887 |
| 2017 | 29-May-17 | FRESNO | GRAPES | Redacted1 | 8.2051 | 40 | A | G | 0.1887 |
| 2017 | 30-May-17 | FRESNO | GRAPES | Redacted1 | 8.2051 | 40 | A | G | 0.1887 |
| 2017 | 31-May-17 | FRESNO | GRAPES | Redacted1 | 8.2051 | 40 | A | G | 0.1887 |
| 2017 | 30-May-17 | FRESNO | GRAPES | Redacted1 | 8.2051 | 40 | A | G | 0.1887 |
| 2017 | 29-May-17 | FRESNO | GRAPES | Redacted1 | 8.2051 | 40 | A | G | 0.1887 |
| 2017 | 30-May-17 | FRESNO | GRAPES | Redacted1 | 8.2051 | 40 | A | G | 0.1887 |
| 2017 | 30-May-17 | FRESNO | GRAPES | Redacted1 | 8.2051 | 40 | A | G | 0.1887 |
| 2017 | 29-May-17 | FRESNO | GRAPES | Redacted1 | 8.2051 | 40 | A | G | 0.1887 |
| 2017 | 31-May-17 | FRESNO | GRAPES | Redacted1 | 8.2051 | 40 | A | G | 0.1887 |
| 2017 | 27-May-17 | FRESNO | GRAPES | Redacted1 | 8.2051 | 40 | A | G | 0.1887 |
| 2017 | 30-May-17 | FRESNO | GRAPES | Redacted1 | 8.2051 | 40 | A | G | 0.1887 |
| 2017 | 30-May-17 | FRESNO | GRAPES | Redacted1 | 8.2051 | 40 | A | G | 0.1887 |
| 2017 | 29-May-17 | FRESNO | GRAPES | Redacted1 | 8.2051 | 40 | A | G | 0.1887 |
| 2017 | 31-May-17 | FRESNO | GRAPES | Redacted1 | 4.5128 | 22 | A | G | 0.1887 |
| 2017 | 31-May-17 | FRESNO | GRAPES | Redacted1 | 6.1538 | 30 | A | G | 0.1887 |
| 2017 | 31-May-17 | FRESNO | GRAPES | Redacted1 | 3.0769 | 15 | A | G | 0.1887 |
| 2017 | 31-May-17 | FRESNO | GRAPES | Redacted1 | 6.1538 | 30 | A | G | 0.1887 |
| 2017 | 30-May-17 | FRESNO | GRAPES | Redacted1 | 24.6152 | 120 | A | G | 0.1887 |
| 2017 | 30-May-17 | FRESNO | GRAPES | Redacted1 | 12.3076 | 60 | A | G | 0.1887 |
| 2017 | 30-May-17 | FRESNO | GRAPES | Redacted1 | 12.3076 | 60 | A | G | 0.1887 |
| 2017 | 31-May-17 | FRESNO | GRAPES | Redacted1 | 6.1538 | 30 | A | G | 0.1887 |
| 2017 | 31-May-17 | FRESNO | GRAPES | Redacted1 | 3.0769 | 15 | A | G | 0.1887 |
| 2017 | 31-May-17 | FRESNO | GRAPES | Redacted1 | 3.0769 | 15 | A | G | 0.1887 |
| 2017 | 30-May-17 | FRESNO | GRAPES | Redacted1 | 6.1538 | 30 | A | G | 0.1887 |
| 2017 | 31-May-17 | FRESNO | GRAPES | Redacted1 | 3.0769 | 15 | A | G | 0.1887 |
| 2017 | 30-May-17 | FRESNO | GRAPES | Redacted1 | 12.3076 | 60 | A | G | 0.1887 |
| 2017 | 29-May-17 | FRESNO | GRAPES | Redacted1 | 6.1538 | 30 | A | G | 0.1887 |
| 2017 | 29-May-17 | FRESNO | GRAPES | Redacted1 | 12.3076 | 60 | A | G | 0.1887 |
| 2017 | 31-May-17 | FRESNO | GRAPES | Redacted1 | 6.1538 | 30 | A | G | 0.1887 |
| 2017 | 31-May-17 | FRESNO | GRAPES | Redacted1 | 10.0512 | 49 | A | G | 0.1887 |
| 2017 | 29-May-17 | FRESNO | GRAPES | Redacted1 | 34.8715 | 170 | A | G | 0.1887 |
| 2017 | 29-May-17 | FRESNO | GRAPES | Redacted1 | 28.7177 | 140 | A | G | 0.1887 |
| 2017 | 29-May-17 | FRESNO | GRAPES | Redacted1 | 16.4101 | 80 | A | G | 0.1887 |
| 2017 | 30-May-17 | FRESNO | GRAPES | Redacted1 | 16.4101 | 80 | A | G | 0.1887 |
| 2017 | 29-May-17 | FRESNO | GRAPES | Redacted1 | 16.4101 | 80 | A | G | 0.1887 |
| 2017 | 31-May-17 | FRESNO | GRAPES | Redacted1 | 16.4101 | 80 | A | G | 0.1887 |
| 2017 | 27-May-17 | FRESNO | GRAPES | Redacted1 | 32.8202 | 160 | A | G | 0.1887 |
| 2017 | 31-May-17 | FRESNO | GRAPES | Redacted1 | 4.7179 | 23 | A | G | 0.1887 |
| 2017 | 31-May-17 | FRESNO | GRAPES | Redacted1 | 20.5126 | 100 | A | G | 0.1887 |
| 2017 | 30-May-17 | FRESNO | GRAPES | Redacted1 | 10.2563 | 50 | A | G | 0.1887 |
| 2017 | 30-May-17 | FRESNO | GRAPES | Redacted1 | 7.1794 | 35 | A | G | 0.1887 |
| 2017 | 30-May-17 | FRESNO | GRAPES | Redacted1 | 7.1794 | 35 | A | G | 0.1887 |
| 2017 | 31-May-17 | FRESNO | GRAPES | Redacted1 | 9.6409 | 47 | A | G | 0.1887 |
| 2017 | 31-May-17 | FRESNO | GRAPES | Redacted1 | 4.1025 | 20 | A | G | 0.1887 |
| 2017 | 31-May-17 | FRESNO | GRAPES | Redacted1 | 4.1025 | 20 | A | G | 0.1887 |
| 2017 | 30-May-17 | FRESNO | GRAPES | Redacted1 | 4.1025 | 20 | A | G | 0.1887 |
| 2017 | 30-May-17 | FRESNO | GRAPES | Redacted1 | 4.1025 | 20 | A | G | 0.1887 |
| 2017 | 31-May-17 | FRESNO | GRAPES | Redacted1 | 4.1025 | 20 | A | G | 0.1887 |
| 2017 | 31-May-17 | FRESNO | GRAPES | Redacted1 | 3.282 | 16 | A | G | 0.1887 |
| 2017 | 31-May-17 | FRESNO | GRAPES | Redacted1 | 4.1025 | 20 | A | G | 0.1887 |
| 2017 | 29-May-17 | FRESNO | GRAPES | Redacted1 | 4.1025 | 20 | A | G | 0.1887 |
| 2017 | 31-May-17 | FRESNO | GRAPES | Redacted1 | 4.1025 | 20 | A | G | 0.1887 |
| 2017 | 30-May-17 | FRESNO | GRAPES | Redacted1 | 4.1025 | 20 | A | G | 0.1887 |
| 2017 | 29-May-17 | FRESNO | GRAPES | Redacted1 | 4.1025 | 20 | A | G | 0.1887 |
| 2017 | 31-May-17 | FRESNO | GRAPES | Redacted1 | 4.1025 | 20 | A | G | 0.1887 |
| 2017 | 29-May-17 | FRESNO | GRAPES | Redacted1 | 4.1025 | 20 | A | G | 0.1887 |
| 2017 | 27-May-17 | FRESNO | GRAPES | Redacted1 | 4.1025 | 20 | A | G | 0.1887 |
| 2017 | 27-May-17 | FRESNO | GRAPES | Redacted1 | 4.1025 | 20 | A | G | 0.1887 |
| 2017 | 31-May-17 | FRESNO | GRAPES | Redacted1 | 4.1025 | 20 | A | G | 0.1887 |
| 2017 | 31-May-17 | FRESNO | GRAPES | Redacted1 | 4.1025 | 20 | A | G | 0.1887 |
| 2017 | 31-May-17 | FRESNO | GRAPES | Redacted1 | 4.1025 | 20 | A | G | 0.1887 |
| 2017 | 31-May-17 | FRESNO | GRAPES | Redacted1 | 4.1025 | 20 | A | G | 0.1887 |
| 2017 | 31-May-17 | FRESNO | GRAPES | Redacted1 | 4.1025 | 20 | A | G | 0.1887 |
| 2017 | 31-May-17 | FRESNO | GRAPES | Redacted1 | 4.1025 | 20 | A | G | 0.1887 |
| 2017 | 31-May-17 | FRESNO | GRAPES | Redacted1 | 4.1025 | 20 | A | G | 0.1887 |
| 2017 | 31-May-17 | FRESNO | GRAPES | Redacted1 | 4.1025 | 20 | A | G | 0.1887 |
| 2017 | 30-May-17 | FRESNO | GRAPES | Redacted1 | 4.1025 | 20 | A | G | 0.1887 |
| 2017 | 31-May-17 | FRESNO | GRAPES | Redacted1 | 4.1025 | 20 | A | G | 0.1887 |
| 2017 | 31-May-17 | FRESNO | GRAPES | Redacted1 | 3.7948 | 18.5 | A | G | 0.1887 |
| 2017 | 29-May-17 | FRESNO | GRAPES | Redacted1 | 3.4871 | 17 | A | G | 0.1887 |
| 2017 | 31-May-17 | FRESNO | GRAPES | Redacted1 | 3.4871 | 17 | A | G | 0.1887 |
| 2017 | 31-May-17 | FRESNO | GRAPES | Redacted1 | 3.4871 | 17 | A | G | 0.1887 |
| 2017 | 31-May-17 | FRESNO | GRAPES | Redacted1 | 2.6666 | 13 | A | G | 0.1887 |
| 2017 | 31-May-17 | FRESNO | GRAPES | Redacted1 | 1.0256 | 5 | A | G | 0.1887 |
| 2017 | 25-Jan-17 | FRESNO | ORANGE (ALL OR UNSPEC) | Redacted1 | 1.7231 | 21 | A | G | 0.0755 |
| 2017 | 25-Jan-17 | FRESNO | ORANGE (ALL OR UNSPEC) | Redacted1 | 4.1846 | 51 | A | G | 0.0755 |
| 2017 | 26-Jan-17 | FRESNO | ORANGE (ALL OR UNSPEC) | Redacted1 | 1.641 | 20 | A | G | 0.0755 |
| 2017 | 26-Jan-17 | FRESNO | ORANGE (ALL OR UNSPEC) | Redacted1 | 5.5794 | 68 | A | G | 0.0755 |
| 2017 | 26-Jan-17 | FRESNO | ORANGE (ALL OR UNSPEC) | Redacted1 | 1.9692 | 24 | A | G | 0.0755 |
| 2017 | 6-Jun-17 | MADERA | PISTACHIO (PISTACHE NUT) | Redacted1 | 5.0871 | 66 | A | A | 0.0709 |
| 2017 | 6-Jun-17 | MADERA | PISTACHIO (PISTACHE NUT) | Redacted1 | 6.2358 | 81 | A | A | 0.0708 |
| 2017 | 6-Jun-17 | MADERA | PISTACHIO (PISTACHE NUT) | Redacted1 | 6.0717 | 79 | A | A | 0.0707 |
| 2017 | 6-Jun-17 | MADERA | PISTACHIO (PISTACHE NUT) | Redacted1 | 6.0717 | 79 | A | A | 0.0707 |
| 2017 | 6-Jun-17 | MADERA | PISTACHIO (PISTACHE NUT) | Redacted1 | 4.841 | 63 | A | A | 0.0707 |
| 2017 | 6-Jun-17 | MADERA | PISTACHIO (PISTACHE NUT) | Redacted1 | 5.9897 | 78 | A | A | 0.0706 |
| 2017 | 6-Jun-17 | MADERA | PISTACHIO (PISTACHE NUT) | Redacted1 | 5.9897 | 78 | A | A | 0.0706 |
| 2017 | 6-Jun-17 | MADERA | PISTACHIO (PISTACHE NUT) | Redacted1 | 5.9897 | 78 | A | A | 0.0706 |
| 2017 | 6-Jun-17 | MADERA | PISTACHIO (PISTACHE NUT) | Redacted1 | 5.9897 | 78 | A | A | 0.0706 |
| 2017 | 14-Apr-17 | SANTA BARBARA | STRAWBERRY (ALL OR UNSPEC) | Redacted1 | 3.8564 | 10 | A | G | 0.3548 |
| 2017 | 15-Apr-17 | SANTA BARBARA | STRAWBERRY (ALL OR UNSPEC) | Redacted1 | 3.0769 | 8 | A | G | 0.3538 |
| 2017 | 25-Jan-17 | FRESNO | TANGERINE | Redacted1 | 4.0205 | 49 | A | G | 0.0755 |
| 2017 | 26-Jan-17 | FRESNO | TANGERINE | Redacted1 | 0.8205 | 10 | A | G | 0.0755 |
| 2017 | 26-Jan-17 | FRESNO | TANGERINE | Redacted1 | 0.8205 | 10 | A | G | 0.0755 |
| 2017 | 10-Mar-17 | IMPERIAL | WHEAT, GENERAL | Redacted1 | 4.1025 | 16 | A | G | 0.2359 |
| 2017 | 5-Mar-17 | IMPERIAL | WHEAT, GENERAL | Redacted1 | 9.2717 | 36.2 | A | G | 0.2356 |
| 2017 | 10-Mar-17 | IMPERIAL | WHEAT, GENERAL | Redacted1 | 9.5999 | 37.5 | A | G | 0.2355 |
| 2017 | 10-Mar-17 | IMPERIAL | WHEAT, GENERAL | Redacted1 | 7.1384 | 27.9 | A | G | 0.2354 |
| 2017 | 5-Mar-17 | IMPERIAL | WHEAT, GENERAL | Redacted1 | 4.5948 | 18 | A | G | 0.2348 |
| 2017 | 10-Mar-17 | IMPERIAL | WHEAT, GENERAL | Redacted1 | 4.5948 | 18 | A | G | 0.2348 |
| 2017 | 5-Mar-17 | IMPERIAL | WHEAT, GENERAL | Redacted1 | 8.3692 | 32.8 | A | G | 0.2347 |
| 2017 | 5-Mar-17 | IMPERIAL | WHEAT, GENERAL | Redacted1 | 4.5128 | 17.7 | A | G | 0.2346 |
| 2017 | 5-Mar-17 | IMPERIAL | WHEAT, GENERAL | Redacted1 | 9.8461 | 38.7 | A | G | 0.2341 |
| 2017 | 5-Mar-17 | IMPERIAL | WHEAT, GENERAL | Redacted1 | 3.9384 | 15.5 | A | G | 0.2338 |
| 2017 | 5-Mar-17 | IMPERIAL | WHEAT, GENERAL | Redacted1 | 4.5948 | 18.1 | A | G | 0.2335 |

| YEAR | DATE | COUNTY_NAME | SITE_NAME | PRODUCT_NAME | POUNDS_PRODUCT_APPLIED | AMOUNT_TREATED | UNIT_TREATED | AERIAL_GROUND_INDICATOR | Application Rate (lb/A) |
| --- | --- | --- | --- | --- | --- | --- | --- | --- | --- |
| 2017 | 4-May-17 | STANISLAUS | ALMOND | Redacted2 | 41.6417 | 200 | A | G | 0.192 |
| 2017 | 30-Apr-17 | STANISLAUS | ALMOND | Redacted2 | 80.3684 | 386 | A | G | 0.192 |
| 2017 | 18-Feb-17 | STANISLAUS | ALMOND | Redacted2 | 12.9089 | 66 | A | G | 0.180 |
| 2017 | 17-Feb-17 | STANISLAUS | ALMOND | Redacted2 | 31.2313 | 160 | A | G | 0.180 |
| 2017 | 13-Feb-17 | STANISLAUS | ALMOND | Redacted2 | 58.5482 | 300 | A | G | 0.180 |
| 2017 | 13-Feb-17 | STANISLAUS | ALMOND | Redacted2 | 13.2421 | 68 | A | G | 0.179 |
| 2017 | 24-Feb-17 | STANISLAUS | ALMOND | Redacted2 | 15.1576 | 78 | A | G | 0.179 |
| 2017 | 25-May-17 | COLUSA | ALMOND | Redacted2 | 3.6645 | 19 | A | G | 0.177 |
| 2017 | 30-May-17 | COLUSA | ALMOND | Redacted2 | 3.6645 | 19 | A | G | 0.177 |
| 2017 | 30-May-17 | COLUSA | ALMOND | Redacted2 | 7.4955 | 39 | A | G | 0.177 |
| 2017 | 29-May-17 | COLUSA | ALMOND | Redacted2 | 7.4955 | 39 | A | G | 0.177 |
| 2017 | 30-Jun-17 | COLUSA | ALMOND | Redacted2 | 7.6621 | 40 | A | G | 0.176 |
| 2017 | 19-May-17 | COLUSA | ALMOND | Redacted2 | 15.3241 | 80 | A | G | 0.176 |
| 2017 | 13-Feb-17 | MERCED | ALMOND | Redacted2 | 3.6645 | 28 | A | G | 0.120 |
| 2017 | 13-Feb-17 | MERCED | ALMOND | Redacted2 | 5.0803 | 39 | A | G | 0.120 |
| 2017 | 14-Feb-17 | MERCED | ALMOND | Redacted2 | 5.0803 | 39 | A | G | 0.120 |
| 2017 | 14-Feb-17 | MERCED | ALMOND | Redacted2 | 2.3319 | 18 | A | G | 0.119 |
| 2017 | 19-Feb-17 | MERCED | ALMOND | Redacted2 | 2.3319 | 18 | A | G | 0.119 |
| 2017 | 21-Feb-17 | MERCED | ALMOND | Redacted2 | 2.3319 | 18 | A | G | 0.119 |
| 2017 | 14-Feb-17 | MERCED | ALMOND | Redacted2 | 3.9976 | 31 | A | G | 0.119 |
| 2017 | 14-Feb-17 | MERCED | ALMOND | Redacted2 | 3.0815 | 24 | A | G | 0.118 |
| 2017 | 14-Feb-17 | MERCED | ALMOND | Redacted2 | 3.0815 | 24 | A | G | 0.118 |
| 2017 | 22-Feb-17 | MERCED | ALMOND | Redacted2 | 3.0815 | 24 | A | G | 0.118 |
| 2017 | 13-Feb-17 | MERCED | ALMOND | Redacted2 | 4.7472 | 37 | A | G | 0.118 |
| 2017 | 16-Feb-17 | MERCED | ALMOND | Redacted2 | 4.997 | 39 | A | G | 0.118 |
| 2017 | 14-Feb-17 | MERCED | ALMOND | Redacted2 | 3.5812 | 28 | A | G | 0.118 |
| 2017 | 14-Feb-17 | MERCED | ALMOND | Redacted2 | 2.1654 | 17 | A | G | 0.117 |
| 2017 | 15-Feb-17 | MERCED | ALMOND | Redacted2 | 2.6651 | 21 | A | G | 0.117 |
| 2017 | 15-Feb-17 | MERCED | ALMOND | Redacted2 | 2.6651 | 21 | A | G | 0.117 |
| 2017 | 14-Feb-17 | MERCED | ALMOND | Redacted2 | 2.6651 | 21 | A | G | 0.117 |
| 2017 | 14-Feb-17 | MERCED | ALMOND | Redacted2 | 2.6651 | 21 | A | G | 0.117 |
| 2017 | 21-Feb-17 | MERCED | ALMOND | Redacted2 | 2.4985 | 19.7 | A | G | 0.117 |
| 2017 | 14-Feb-17 | MERCED | ALMOND | Redacted2 | 2.4985 | 19.7 | A | G | 0.117 |
| 2017 | 24-Feb-17 | STANISLAUS | ALMOND | Redacted2 | 1.4991 | 15 | A | G | 0.092 |
| 2017 | 15-Feb-17 | STANISLAUS | ALMOND | Redacted2 | 5.7466 | 60 | A | G | 0.088 |
| 2017 | 14-Feb-17 | STANISLAUS | ALMOND | Redacted2 | 3.831 | 40 | A | G | 0.088 |
| 2017 | 14-Feb-17 | STANISLAUS | ALMOND | Redacted2 | 1.9155 | 20 | A | G | 0.088 |
| 2017 | 15-Feb-17 | STANISLAUS | ALMOND | Redacted2 | 3.831 | 40 | A | G | 0.088 |
| 2017 | 23-Feb-17 | MERCED | ALMOND | Redacted2 | 1.749 | 22 | A | G | 0.073 |
| 2017 | 22-Feb-17 | MERCED | ALMOND | Redacted2 | 2.9149 | 37 | A | G | 0.072 |
| 2017 | 15-Feb-17 | MERCED | ALMOND | Redacted2 | 2.6651 | 34 | A | G | 0.072 |
| 2017 | 22-Feb-17 | MERCED | ALMOND | Redacted2 | 6.2463 | 80 | A | G | 0.072 |
| 2017 | 22-Feb-17 | MERCED | ALMOND | Redacted2 | 1.166 | 15 | A | G | 0.072 |
| 2017 | 30-Apr-17 | MONTEREY | ARTICHOKE (GLOBE) (ALL OR UNSPEC) | Redacted2 | 6.6627 | 17 | A | G | 0.361 |
| 2017 | 18-May-17 | MONTEREY | ARTICHOKE (GLOBE) (ALL OR UNSPEC) | Redacted2 | 1.9155 | 6.5 | A | G | 0.271 |
| 2017 | 15-May-17 | MONTEREY | ARTICHOKE (GLOBE) (ALL OR UNSPEC) | Redacted2 | 4.8304 | 16.5 | A | G | 0.269 |
| 2017 | 18-May-17 | MONTEREY | ARTICHOKE (GLOBE) (ALL OR UNSPEC) | Redacted2 | 2.9149 | 10 | A | G | 0.268 |
| 2017 | 23-May-17 | MONTEREY | ARTICHOKE (GLOBE) (ALL OR UNSPEC) | Redacted2 | 1.7802 | 17.1 | A | A | 0.096 |
| 2017 | 5-May-17 | MONTEREY | ARTICHOKE (GLOBE) (ALL OR UNSPEC) | Redacted2 | 1.3195 | 16.9 | A | A | 0.072 |
| 2017 | 22-May-17 | MONTEREY | ARTICHOKE (GLOBE) (ALL OR UNSPEC) | Redacted2 | 1.3273 | 17 | A | A | 0.072 |
| 2017 | 26-Apr-17 | MONTEREY | BLACKBERRY | Redacted2 | 0.4164 | 1 | A | G | 0.383 |
| 2017 | 26-Apr-17 | MONTEREY | BLACKBERRY | Redacted2 | 0.4164 | 1 | A | G | 0.383 |
| 2017 | 27-Apr-17 | MONTEREY | BLACKBERRY | Redacted2 | 0.2499 | 0.62 | A | G | 0.371 |
| 2017 | 27-Apr-17 | MONTEREY | BLACKBERRY | Redacted2 | 1.9155 | 4.81 | A | G | 0.366 |
| 2017 | 11-Jul-17 | MONTEREY | BLACKBERRY | Redacted2 | 1.749 | 4.4 | A | G | 0.366 |
| 2017 | 28-Apr-17 | MONTEREY | BLACKBERRY | Redacted2 | 1.8322 | 4.63 | A | G | 0.364 |
| 2017 | 29-Apr-17 | MONTEREY | BLACKBERRY | Redacted2 | 1.8322 | 4.63 | A | G | 0.364 |
| 2017 | 28-Apr-17 | MONTEREY | BLACKBERRY | Redacted2 | 1.0827 | 2.75 | A | G | 0.362 |
| 2017 | 29-Apr-17 | MONTEREY | BLACKBERRY | Redacted2 | 1.0827 | 2.75 | A | G | 0.362 |
| 2017 | 28-Apr-17 | MONTEREY | BLACKBERRY | Redacted2 | 4.6639 | 11.86 | A | G | 0.362 |
| 2017 | 29-Apr-17 | MONTEREY | BLACKBERRY | Redacted2 | 1.3325 | 3.4 | A | G | 0.361 |
| 2017 | 28-Apr-17 | MONTEREY | BLACKBERRY | Redacted2 | 1.3325 | 3.4 | A | G | 0.361 |
| 2017 | 27-Apr-17 | MONTEREY | BLACKBERRY | Redacted2 | 2.1654 | 5.6 | A | G | 0.356 |
| 2017 | 29-Apr-17 | MONTEREY | BLACKBERRY | Redacted2 | 0.4164 | 1.08 | A | G | 0.355 |
| 2017 | 28-Apr-17 | MONTEREY | BLACKBERRY | Redacted2 | 0.4164 | 1.08 | A | G | 0.355 |
| 2017 | 4-Mar-17 | MONTEREY | BLACKBERRY | Redacted2 | 0.026 | 0.2 | A | G | 0.120 |
| 2017 | 4-Mar-17 | MONTEREY | BLACKBERRY | Redacted2 | 0.1549 | 1.2 | A | G | 0.119 |
| 2017 | 4-Mar-17 | MONTEREY | BLACKBERRY | Redacted2 | 0.2499 | 2 | A | G | 0.115 |
| 2017 | 28-Feb-17 | MONTEREY | BLACKBERRY | Redacted2 | 0.2499 | 2 | A | G | 0.115 |
| 2017 | 4-Mar-17 | MONTEREY | BLACKBERRY | Redacted2 | 0.2499 | 2 | A | G | 0.115 |
| 2017 | 13-Mar-17 | MONTEREY | BLACKBERRY | Redacted2 | 0.0755 | 0.64 | A | G | 0.109 |
| 2017 | 13-Mar-17 | MONTEREY | BLACKBERRY | Redacted2 | 0.2316 | 1.97 | A | G | 0.108 |
| 2017 | 13-Mar-17 | MONTEREY | BLACKBERRY | Redacted2 | 0.2186 | 1.86 | A | G | 0.108 |
| 2017 | 13-Mar-17 | MONTEREY | BLACKBERRY | Redacted2 | 0.2362 | 2.01 | A | G | 0.108 |
| 2017 | 13-Mar-17 | MONTEREY | BLACKBERRY | Redacted2 | 0.4958 | 4.22 | A | G | 0.108 |
| 2017 | 13-Mar-17 | MONTEREY | BLACKBERRY | Redacted2 | 0.5862 | 4.99 | A | G | 0.108 |
| 2017 | 13-Mar-17 | MONTEREY | BLACKBERRY | Redacted2 | 0.3019 | 2.57 | A | G | 0.108 |
| 2017 | 13-Mar-17 | MONTEREY | BLACKBERRY | Redacted2 | 1.1582 | 9.86 | A | G | 0.108 |
| 2017 | 13-Mar-17 | MONTEREY | BLACKBERRY | Redacted2 | 0.2102 | 1.79 | A | G | 0.108 |
| 2017 | 13-Mar-17 | MONTEREY | BLACKBERRY | Redacted2 | 0.2583 | 2.2 | A | G | 0.108 |
| 2017 | 13-Mar-17 | MONTEREY | BLACKBERRY | Redacted2 | 0.5706 | 4.86 | A | G | 0.108 |
| 2017 | 13-Mar-17 | MONTEREY | BLACKBERRY | Redacted2 | 0.8393 | 7.15 | A | G | 0.108 |
| 2017 | 13-Mar-17 | MONTEREY | BLACKBERRY | Redacted2 | 0.3357 | 2.86 | A | G | 0.108 |
| 2017 | 13-Mar-17 | MONTEREY | BLACKBERRY | Redacted2 | 0.3826 | 3.26 | A | G | 0.108 |
| 2017 | 13-Mar-17 | MONTEREY | BLACKBERRY | Redacted2 | 0.3097 | 2.64 | A | G | 0.108 |
| 2017 | 13-Mar-17 | MONTEREY | BLACKBERRY | Redacted2 | 0.1113 | 0.95 | A | G | 0.108 |
| 2017 | 28-Feb-17 | MONTEREY | BLACKBERRY | Redacted2 | 0.1666 | 1.5 | A | G | 0.102 |
| 2017 | 25-May-17 | MONTEREY | CARROTS, GENERAL | Redacted2 | 1.464 | 22.5 | A | A | 0.060 |
| 2017 | 24-May-17 | MONTEREY | CARROTS, GENERAL | Redacted2 | 0.6897 | 10.6 | A | A | 0.060 |
| 2017 | 24-May-17 | MONTEREY | CARROTS, GENERAL | Redacted2 | 0.6663 | 10.6 | A | A | 0.058 |
| 2017 | 11-May-17 | MONTEREY | CELERY, GENERAL | Redacted2 | 1.9155 | 6.5 | A | G | 0.271 |
| 2017 | 3-May-17 | MONTEREY | CELERY, GENERAL | Redacted2 | 4.0809 | 14 | A | G | 0.268 |
| 2017 | 18-May-17 | MONTEREY | CELERY, GENERAL | Redacted2 | 4.0809 | 14 | A | G | 0.268 |
| 2017 | 23-May-17 | MONTEREY | CELERY, GENERAL | Redacted2 | 2.6651 | 9.2 | A | G | 0.267 |
| 2017 | 18-May-17 | MONTEREY | CELERY, GENERAL | Redacted2 | 0.6663 | 2.4 | A | G | 0.255 |
| 2017 | 3-May-17 | MONTEREY | CELERY, GENERAL | Redacted2 | 0.6663 | 2.4 | A | G | 0.255 |
| 2017 | 16-May-17 | MONTEREY | CILANTRO (CHINESE PARSLEY, CORIANDER LEAVES) | Redacted2 | 0.4164 | 2.65 | A | G | 0.145 |
| 2017 | 16-May-17 | MONTEREY | CILANTRO (CHINESE PARSLEY, CORIANDER LEAVES) | Redacted2 | 1.0827 | 7.34 | A | G | 0.136 |
| 2017 | 16-May-17 | MONTEREY | CILANTRO (CHINESE PARSLEY, CORIANDER LEAVES) | Redacted2 | 1.0827 | 7.77 | A | G | 0.128 |
| 2017 | 24-May-17 | MONTEREY | CILANTRO (CHINESE PARSLEY, CORIANDER LEAVES) | Redacted2 | 0.6663 | 7.04 | A | G | 0.087 |
| 2017 | 7-May-17 | MONTEREY | ENDIVE (ESCAROLE) | Redacted2 | 0.583 | 2.25 | A | G | 0.238 |
| 2017 | 15-May-17 | MONTEREY | ENDIVE (ESCAROLE) | Redacted2 | 0.583 | 2.25 | A | G | 0.238 |
| 2017 | 20-May-17 | MONTEREY | ENDIVE (ESCAROLE) | Redacted2 | 0.583 | 2.31 | A | G | 0.232 |
| 2017 | 17-May-17 | MONTEREY | ENDIVE (ESCAROLE) | Redacted2 | 0.2499 | 1 | A | G | 0.230 |
| 2017 | 21-May-17 | MONTEREY | ENDIVE (ESCAROLE) | Redacted2 | 0.9994 | 4 | A | G | 0.230 |
| 2017 | 3-May-17 | MONTEREY | ENDIVE (ESCAROLE) | Redacted2 | 1.0827 | 4.37 | A | G | 0.228 |
| 2017 | 6-May-17 | MONTEREY | ENDIVE (ESCAROLE) | Redacted2 | 1.0827 | 4.4 | A | G | 0.226 |
| 2017 | 13-May-17 | MONTEREY | ENDIVE (ESCAROLE) | Redacted2 | 1.0827 | 4.4 | A | G | 0.226 |
| 2017 | 7-May-17 | MONTEREY | ENDIVE (ESCAROLE) | Redacted2 | 0.9994 | 4.12 | A | G | 0.223 |
| 2017 | 15-May-17 | MONTEREY | ENDIVE (ESCAROLE) | Redacted2 | 0.9994 | 4.12 | A | G | 0.223 |
| 2017 | 21-May-17 | MONTEREY | ENDIVE (ESCAROLE) | Redacted2 | 0.9994 | 4.13 | A | G | 0.223 |
| 2017 | 7-May-17 | MONTEREY | ENDIVE (ESCAROLE) | Redacted2 | 0.4997 | 2.1 | A | G | 0.219 |
| 2017 | 17-May-17 | MONTEREY | ENDIVE (ESCAROLE) | Redacted2 | 0.4997 | 2.1 | A | G | 0.219 |
| 2017 | 3-May-17 | MONTEREY | ENDIVE (ESCAROLE) | Redacted2 | 0.6663 | 2.84 | A | G | 0.216 |
| 2017 | 7-May-17 | MONTEREY | ENDIVE (ESCAROLE) | Redacted2 | 0.7496 | 3.2 | A | G | 0.216 |
| 2017 | 17-May-17 | MONTEREY | ENDIVE (ESCAROLE) | Redacted2 | 0.7496 | 3.2 | A | G | 0.216 |
| 2017 | 13-May-17 | MONTEREY | ENDIVE (ESCAROLE) | Redacted2 | 0.583 | 2.5 | A | G | 0.215 |
| 2017 | 6-May-17 | MONTEREY | ENDIVE (ESCAROLE) | Redacted2 | 0.583 | 2.5 | A | G | 0.215 |
| 2017 | 21-May-17 | MONTEREY | ENDIVE (ESCAROLE) | Redacted2 | 0.4997 | 2.22 | A | G | 0.207 |
| 2017 | 9-May-17 | MONTEREY | ENDIVE (ESCAROLE) | Redacted2 | 0.4164 | 2.22 | A | G | 0.173 |
| 2017 | 7-May-17 | MONTEREY | ENDIVE (ESCAROLE) | Redacted2 | 0.7496 | 4 | A | G | 0.172 |
| 2017 | 7-May-17 | MONTEREY | ENDIVE (ESCAROLE) | Redacted2 | 0.1666 | 1 | A | G | 0.153 |
| 2017 | 11-May-17 | MONTEREY | ENDIVE (ESCAROLE) | Redacted2 | 0.1666 | 2.31 | A | A | 0.066 |
| 2017 | 11-May-17 | MONTEREY | ENDIVE (ESCAROLE) | Redacted2 | 0.1503 | 2.31 | A | A | 0.060 |
| 2017 | 11-May-17 | MONTEREY | ENDIVE (ESCAROLE) | Redacted2 | 0.2687 | 4.13 | A | A | 0.060 |
| 2017 | 11-May-17 | MONTEREY | ENDIVE (ESCAROLE) | Redacted2 | 0.2499 | 4.13 | A | A | 0.056 |
| 2017 | 9-May-17 | MONTEREY | GARLIC | Redacted2 | 0.4997 | 2 | A | G | 0.230 |
| 2017 | 9-May-17 | MONTEREY | GARLIC | Redacted2 | 5.2469 | 21.4 | A | G | 0.226 |
| 2017 | 9-May-17 | MONTEREY | GARLIC | Redacted2 | 2.9149 | 11.9 | A | G | 0.225 |
| 2017 | 9-May-17 | MONTEREY | GARLIC | Redacted2 | 3.4979 | 14.3 | A | G | 0.225 |
| 2017 | 9-May-17 | MONTEREY | GARLIC | Redacted2 | 2.9149 | 12 | A | G | 0.223 |
| 2017 | 9-May-17 | MONTEREY | GARLIC | Redacted2 | 1.6657 | 7 | A | G | 0.219 |
| 2017 | 29-Jun-17 | SAN JOAQUIN | GRAPES, WINE | Redacted2 | 3.3313 | 8.5 | A | G | 0.361 |
| 2017 | 30-Jun-17 | SAN JOAQUIN | GRAPES, WINE | Redacted2 | 6.2463 | 16 | A | G | 0.359 |
| 2017 | 26-Jun-17 | SAN JOAQUIN | GRAPES, WINE | Redacted2 | 3.1231 | 9 | A | G | 0.319 |
| 2017 | 10-Mar-17 | SAN JOAQUIN | GRAPES, WINE | Redacted2 | 5.226 | 26.78 | A | G | 0.180 |
| 2017 | 24-May-17 | MONTEREY | LEEK | Redacted2 | 0.9161 | 3.6 | A | G | 0.234 |
| 2017 | 5-May-17 | MONTEREY | LEEK | Redacted2 | 0.9994 | 4.1 | A | G | 0.224 |
| 2017 | 5-May-17 | MONTEREY | LEEK | Redacted2 | 0.9994 | 4.1 | A | G | 0.224 |
| 2017 | 24-May-17 | MONTEREY | LEEK | Redacted2 | 0.6663 | 2.8 | A | G | 0.219 |
| 2017 | 5-May-17 | MONTEREY | LEEK | Redacted2 | 0.7496 | 3.2 | A | G | 0.216 |
| 2017 | 5-May-17 | MONTEREY | LEEK | Redacted2 | 0.6663 | 2.9 | A | G | 0.211 |
| 2017 | 24-May-17 | MONTEREY | LEEK | Redacted2 | 0.3331 | 1.5 | A | G | 0.204 |
| 2017 | 5-May-17 | MONTEREY | LEEK | Redacted2 | 0.3331 | 1.5 | A | G | 0.204 |
| 2017 | 29-Apr-17 | MONTEREY | LETTUCE, HEAD (ALL OR UNSPEC) | Redacted2 | 7.2457 | 18.6 | A | G | 0.358 |
| 2017 | 17-May-17 | MONTEREY | LETTUCE, HEAD (ALL OR UNSPEC) | Redacted2 | 3.6645 | 12.4 | A | G | 0.272 |
| 2017 | 10-May-17 | MONTEREY | LETTUCE, HEAD (ALL OR UNSPEC) | Redacted2 | 3.7478 | 12.7 | A | G | 0.271 |
| 2017 | 17-May-17 | MONTEREY | LETTUCE, HEAD (ALL OR UNSPEC) | Redacted2 | 3.831 | 13 | A | G | 0.271 |
| 2017 | 22-May-17 | MONTEREY | LETTUCE, HEAD (ALL OR UNSPEC) | Redacted2 | 4.8304 | 16.4 | A | G | 0.271 |
| 2017 | 25-May-17 | MONTEREY | LETTUCE, HEAD (ALL OR UNSPEC) | Redacted2 | 3.9143 | 13.3 | A | G | 0.271 |
| 2017 | 30-Apr-17 | MONTEREY | LETTUCE, HEAD (ALL OR UNSPEC) | Redacted2 | 7.4122 | 25.3 | A | G | 0.270 |
| 2017 | 21-May-17 | MONTEREY | LETTUCE, HEAD (ALL OR UNSPEC) | Redacted2 | 3.2481 | 11.2 | A | G | 0.267 |
| 2017 | 23-May-17 | MONTEREY | LETTUCE, HEAD (ALL OR UNSPEC) | Redacted2 | 3.2481 | 14 | A | G | 0.213 |
| 2017 | 29-Apr-17 | MONTEREY | LETTUCE, HEAD (ALL OR UNSPEC) | Redacted2 | 3.2481 | 16.4 | A | G | 0.182 |
| 2017 | 20-May-17 | MONTEREY | LETTUCE, HEAD (ALL OR UNSPEC) | Redacted2 | 2.2487 | 11.4 | A | G | 0.181 |
| 2017 | 13-May-17 | MONTEREY | LETTUCE, HEAD (ALL OR UNSPEC) | Redacted2 | 2.2487 | 11.4 | A | G | 0.181 |
| 2017 | 18-May-17 | MONTEREY | LETTUCE, HEAD (ALL OR UNSPEC) | Redacted2 | 2.1654 | 11 | A | G | 0.181 |
| 2017 | 12-May-17 | MONTEREY | LETTUCE, HEAD (ALL OR UNSPEC) | Redacted2 | 1.6657 | 8.5 | A | G | 0.180 |
| 2017 | 9-May-17 | MONTEREY | LETTUCE, HEAD (ALL OR UNSPEC) | Redacted2 | 4.414 | 22.7 | A | G | 0.179 |
| 2017 | 14-May-17 | MONTEREY | LETTUCE, HEAD (ALL OR UNSPEC) | Redacted2 | 2.5818 | 13.3 | A | G | 0.179 |
| 2017 | 16-May-17 | MONTEREY | LETTUCE, HEAD (ALL OR UNSPEC) | Redacted2 | 2.9149 | 15.1 | A | G | 0.178 |
| 2017 | 9-May-17 | MONTEREY | LETTUCE, HEAD (ALL OR UNSPEC) | Redacted2 | 1.9988 | 25.5 | A | A | 0.072 |
| 2017 | 6-May-17 | MONTEREY | LETTUCE, HEAD (ALL OR UNSPEC) | Redacted2 | 0.6715 | 8.6 | A | A | 0.072 |
| 2017 | 9-May-17 | MONTEREY | LETTUCE, HEAD (ALL OR UNSPEC) | Redacted2 | 1.991 | 25.5 | A | A | 0.072 |
| 2017 | 7-May-17 | MONTEREY | LETTUCE, HEAD (ALL OR UNSPEC) | Redacted2 | 0.6507 | 10 | A | A | 0.060 |
| 2017 | 18-May-17 | MONTEREY | LETTUCE, LEAF (ALL OR UNSPEC) | Redacted2 | 0.8328 | 2.8 | A | G | 0.274 |
| 2017 | 22-May-17 | MONTEREY | LETTUCE, LEAF (ALL OR UNSPEC) | Redacted2 | 2.2487 | 7.6 | A | G | 0.272 |
| 2017 | 6-May-17 | MONTEREY | LETTUCE, LEAF (ALL OR UNSPEC) | Redacted2 | 5.6633 | 19.2 | A | G | 0.271 |
| 2017 | 10-May-17 | MONTEREY | LETTUCE, LEAF (ALL OR UNSPEC) | Redacted2 | 1.4991 | 5.1 | A | G | 0.270 |
| 2017 | 4-May-17 | MONTEREY | LETTUCE, LEAF (ALL OR UNSPEC) | Redacted2 | 1.4991 | 5.1 | A | G | 0.270 |
| 2017 | 17-May-17 | MONTEREY | LETTUCE, LEAF (ALL OR UNSPEC) | Redacted2 | 8.0785 | 27.5 | A | G | 0.270 |
| 2017 | 23-May-17 | MONTEREY | LETTUCE, LEAF (ALL OR UNSPEC) | Redacted2 | 8.0785 | 27.5 | A | G | 0.270 |
| 2017 | 9-May-17 | MONTEREY | LETTUCE, LEAF (ALL OR UNSPEC) | Redacted2 | 8.0785 | 27.5 | A | G | 0.270 |
| 2017 | 16-May-17 | MONTEREY | LETTUCE, LEAF (ALL OR UNSPEC) | Redacted2 | 6.8292 | 23.3 | A | G | 0.270 |
| 2017 | 22-May-17 | MONTEREY | LETTUCE, LEAF (ALL OR UNSPEC) | Redacted2 | 6.8292 | 23.3 | A | G | 0.270 |
| 2017 | 18-May-17 | MONTEREY | LETTUCE, LEAF (ALL OR UNSPEC) | Redacted2 | 4.9137 | 16.8 | A | G | 0.269 |
| 2017 | 23-May-17 | MONTEREY | LETTUCE, LEAF (ALL OR UNSPEC) | Redacted2 | 1.166 | 4 | A | G | 0.268 |
| 2017 | 23-May-17 | MONTEREY | LETTUCE, LEAF (ALL OR UNSPEC) | Redacted2 | 3.1648 | 10.9 | A | G | 0.267 |
| 2017 | 7-May-17 | MONTEREY | LETTUCE, LEAF (ALL OR UNSPEC) | Redacted2 | 0.4997 | 1.92 | A | G | 0.239 |
| 2017 | 17-May-17 | MONTEREY | LETTUCE, LEAF (ALL OR UNSPEC) | Redacted2 | 0.4997 | 1.92 | A | G | 0.239 |
| 2017 | 7-May-17 | MONTEREY | LETTUCE, LEAF (ALL OR UNSPEC) | Redacted2 | 0.8328 | 3.3 | A | G | 0.232 |
| 2017 | 17-May-17 | MONTEREY | LETTUCE, LEAF (ALL OR UNSPEC) | Redacted2 | 0.8328 | 3.3 | A | G | 0.232 |
| 2017 | 21-May-17 | MONTEREY | LETTUCE, LEAF (ALL OR UNSPEC) | Redacted2 | 0.8328 | 3.33 | A | G | 0.230 |
| 2017 | 30-Apr-17 | MONTEREY | LETTUCE, LEAF (ALL OR UNSPEC) | Redacted2 | 1.4991 | 6 | A | G | 0.230 |
| 2017 | 29-Apr-17 | MONTEREY | LETTUCE, LEAF (ALL OR UNSPEC) | Redacted2 | 2.1654 | 8.8 | A | G | 0.226 |
| 2017 | 4-May-17 | MONTEREY | LETTUCE, LEAF (ALL OR UNSPEC) | Redacted2 | 0.8328 | 3.4 | A | G | 0.225 |
| 2017 | 19-May-17 | MONTEREY | LETTUCE, LEAF (ALL OR UNSPEC) | Redacted2 | 2.9149 | 12 | A | G | 0.223 |
| 2017 | 3-May-17 | MONTEREY | LETTUCE, LEAF (ALL OR UNSPEC) | Redacted2 | 2.9149 | 12 | A | G | 0.223 |
| 2017 | 16-May-17 | MONTEREY | LETTUCE, LEAF (ALL OR UNSPEC) | Redacted2 | 2.4152 | 10 | A | G | 0.222 |
| 2017 | 17-May-17 | MONTEREY | LETTUCE, LEAF (ALL OR UNSPEC) | Redacted2 | 1.2493 | 5.22 | A | G | 0.220 |
| 2017 | 21-May-17 | MONTEREY | LETTUCE, LEAF (ALL OR UNSPEC) | Redacted2 | 0.4164 | 1.76 | A | G | 0.218 |
| 2017 | 22-May-17 | MONTEREY | LETTUCE, LEAF (ALL OR UNSPEC) | Redacted2 | 2.7484 | 11.7 | A | G | 0.216 |
| 2017 | 24-May-17 | MONTEREY | LETTUCE, LEAF (ALL OR UNSPEC) | Redacted2 | 2.1654 | 9.3 | A | G | 0.214 |
| 2017 | 20-May-17 | MONTEREY | LETTUCE, LEAF (ALL OR UNSPEC) | Redacted2 | 2.6651 | 11.5 | A | G | 0.213 |
| 2017 | 3-May-17 | MONTEREY | LETTUCE, LEAF (ALL OR UNSPEC) | Redacted2 | 1.749 | 7.6 | A | G | 0.212 |
| 2017 | 7-May-17 | MONTEREY | LETTUCE, LEAF (ALL OR UNSPEC) | Redacted2 | 0.2499 | 1.13 | A | G | 0.203 |
| 2017 | 4-May-17 | MONTEREY | LETTUCE, LEAF (ALL OR UNSPEC) | Redacted2 | 0.1666 | 0.8 | A | G | 0.192 |
| 2017 | 9-May-17 | MONTEREY | LETTUCE, LEAF (ALL OR UNSPEC) | Redacted2 | 0.6663 | 3.33 | A | G | 0.184 |
| 2017 | 10-May-17 | MONTEREY | LETTUCE, LEAF (ALL OR UNSPEC) | Redacted2 | 1.9988 | 10 | A | G | 0.184 |
| 2017 | 19-May-17 | MONTEREY | LETTUCE, LEAF (ALL OR UNSPEC) | Redacted2 | 0.9994 | 5 | A | G | 0.184 |
| 2017 | 19-May-17 | MONTEREY | LETTUCE, LEAF (ALL OR UNSPEC) | Redacted2 | 0.9994 | 5 | A | G | 0.184 |
| 2017 | 15-May-17 | MONTEREY | LETTUCE, LEAF (ALL OR UNSPEC) | Redacted2 | 1.5824 | 8 | A | G | 0.182 |
| 2017 | 13-May-17 | MONTEREY | LETTUCE, LEAF (ALL OR UNSPEC) | Redacted2 | 2.1654 | 11 | A | G | 0.181 |
| 2017 | 6-May-17 | MONTEREY | LETTUCE, LEAF (ALL OR UNSPEC) | Redacted2 | 0.6663 | 3.39 | A | G | 0.181 |
| 2017 | 23-May-17 | MONTEREY | LETTUCE, LEAF (ALL OR UNSPEC) | Redacted2 | 2.4152 | 12.3 | A | G | 0.181 |
| 2017 | 23-May-17 | MONTEREY | LETTUCE, LEAF (ALL OR UNSPEC) | Redacted2 | 1.6657 | 8.5 | A | G | 0.180 |
| 2017 | 19-May-17 | MONTEREY | LETTUCE, LEAF (ALL OR UNSPEC) | Redacted2 | 2.5818 | 13.2 | A | G | 0.180 |
| 2017 | 6-May-17 | MONTEREY | LETTUCE, LEAF (ALL OR UNSPEC) | Redacted2 | 1.166 | 6 | A | G | 0.179 |
| 2017 | 7-May-17 | MONTEREY | LETTUCE, LEAF (ALL OR UNSPEC) | Redacted2 | 1.9155 | 10 | A | G | 0.176 |
| 2017 | 9-May-17 | MONTEREY | LETTUCE, LEAF (ALL OR UNSPEC) | Redacted2 | 1.9155 | 10 | A | G | 0.176 |
| 2017 | 20-May-17 | MONTEREY | LETTUCE, LEAF (ALL OR UNSPEC) | Redacted2 | 1.9155 | 10 | A | G | 0.176 |
| 2017 | 13-May-17 | MONTEREY | LETTUCE, LEAF (ALL OR UNSPEC) | Redacted2 | 1.9155 | 10 | A | G | 0.176 |
| 2017 | 17-May-17 | MONTEREY | LETTUCE, LEAF (ALL OR UNSPEC) | Redacted2 | 1.9155 | 10 | A | G | 0.176 |
| 2017 | 5-May-17 | MONTEREY | LETTUCE, LEAF (ALL OR UNSPEC) | Redacted2 | 1.3325 | 7 | A | G | 0.175 |
| 2017 | 9-May-17 | MONTEREY | LETTUCE, LEAF (ALL OR UNSPEC) | Redacted2 | 0.3331 | 1.76 | A | G | 0.174 |
| 2017 | 6-May-17 | MONTEREY | LETTUCE, LEAF (ALL OR UNSPEC) | Redacted2 | 0.7496 | 4 | A | G | 0.172 |
| 2017 | 6-May-17 | MONTEREY | LETTUCE, LEAF (ALL OR UNSPEC) | Redacted2 | 0.3331 | 1.92 | A | G | 0.160 |
| 2017 | 7-May-17 | MONTEREY | LETTUCE, LEAF (ALL OR UNSPEC) | Redacted2 | 0.6663 | 10 | A | A | 0.061 |
| 2017 | 25-May-17 | SANTA CRUZ | RASPBERRY (ALL OR UNSPEC) | Redacted2 | 1.0827 | 2.12 | A | G | 0.470 |
| 2017 | 7-Jun-17 | MONTEREY | RASPBERRY (ALL OR UNSPEC) | Redacted2 | 0.8328 | 2.05 | A | G | 0.374 |
| 2017 | 18-May-17 | MONTEREY | RASPBERRY (ALL OR UNSPEC) | Redacted2 | 1.4158 | 3.55 | A | G | 0.367 |
| 2017 | 28-Apr-17 | MONTEREY | RASPBERRY (ALL OR UNSPEC) | Redacted2 | 1.8322 | 4.63 | A | G | 0.364 |
| 2017 | 29-Apr-17 | MONTEREY | RASPBERRY (ALL OR UNSPEC) | Redacted2 | 1.0827 | 2.75 | A | G | 0.362 |
| 2017 | 11-Jul-17 | MONTEREY | RASPBERRY (ALL OR UNSPEC) | Redacted2 | 2.6651 | 6.78 | A | G | 0.362 |
| 2017 | 14-Jul-17 | MONTEREY | RASPBERRY (ALL OR UNSPEC) | Redacted2 | 2.4985 | 6.36 | A | G | 0.361 |
| 2017 | 27-Apr-17 | MONTEREY | RASPBERRY (ALL OR UNSPEC) | Redacted2 | 0.1256 | 0.32 | A | G | 0.361 |
| 2017 | 17-May-17 | MONTEREY | RASPBERRY (ALL OR UNSPEC) | Redacted2 | 0.1256 | 0.32 | A | G | 0.361 |
| 2017 | 17-May-17 | MONTEREY | RASPBERRY (ALL OR UNSPEC) | Redacted2 | 0.149 | 0.38 | A | G | 0.361 |
| 2017 | 29-Apr-17 | MONTEREY | RASPBERRY (ALL OR UNSPEC) | Redacted2 | 1.3325 | 3.4 | A | G | 0.361 |
| 2017 | 17-May-17 | MONTEREY | RASPBERRY (ALL OR UNSPEC) | Redacted2 | 0.4229 | 1.08 | A | G | 0.360 |
| 2017 | 17-May-17 | MONTEREY | RASPBERRY (ALL OR UNSPEC) | Redacted2 | 0.4542 | 1.16 | A | G | 0.360 |
| 2017 | 17-May-17 | MONTEREY | RASPBERRY (ALL OR UNSPEC) | Redacted2 | 1.3586 | 3.47 | A | G | 0.360 |
| 2017 | 27-Apr-17 | MONTEREY | RASPBERRY (ALL OR UNSPEC) | Redacted2 | 1.883 | 4.81 | A | G | 0.360 |
| 2017 | 27-Apr-17 | MONTEREY | RASPBERRY (ALL OR UNSPEC) | Redacted2 | 0.2427 | 0.62 | A | G | 0.360 |
| 2017 | 27-Apr-17 | MONTEREY | RASPBERRY (ALL OR UNSPEC) | Redacted2 | 2.192 | 5.6 | A | G | 0.360 |
| 2017 | 17-May-17 | MONTEREY | RASPBERRY (ALL OR UNSPEC) | Redacted2 | 0.8023 | 2.05 | A | G | 0.360 |
| 2017 | 17-May-17 | MONTEREY | RASPBERRY (ALL OR UNSPEC) | Redacted2 | 1.3306 | 3.4 | A | G | 0.360 |
| 2017 | 17-May-17 | MONTEREY | RASPBERRY (ALL OR UNSPEC) | Redacted2 | 1.0762 | 2.75 | A | G | 0.360 |
| 2017 | 26-May-17 | SANTA CRUZ | RASPBERRY (ALL OR UNSPEC) | Redacted2 | 9.7442 | 24.92 | A | G | 0.360 |
| 2017 | 17-May-17 | MONTEREY | RASPBERRY (ALL OR UNSPEC) | Redacted2 | 0.0937 | 0.24 | A | G | 0.359 |
| 2017 | 1-Jun-17 | MONTEREY | RASPBERRY (ALL OR UNSPEC) | Redacted2 | 0.9526 | 2.44 | A | G | 0.359 |
| 2017 | 2-Jun-17 | MONTEREY | RASPBERRY (ALL OR UNSPEC) | Redacted2 | 1.0736 | 2.75 | A | G | 0.359 |
| 2017 | 1-Jun-17 | MONTEREY | RASPBERRY (ALL OR UNSPEC) | Redacted2 | 1.3859 | 3.55 | A | G | 0.359 |
| 2017 | 2-Jun-17 | MONTEREY | RASPBERRY (ALL OR UNSPEC) | Redacted2 | 1.8075 | 4.63 | A | G | 0.359 |
| 2017 | 2-Jun-17 | MONTEREY | RASPBERRY (ALL OR UNSPEC) | Redacted2 | 1.3273 | 3.4 | A | G | 0.359 |
| 2017 | 2-Jun-17 | MONTEREY | RASPBERRY (ALL OR UNSPEC) | Redacted2 | 0.4216 | 1.08 | A | G | 0.359 |
| 2017 | 2-Jun-17 | MONTEREY | RASPBERRY (ALL OR UNSPEC) | Redacted2 | 0.242 | 0.62 | A | G | 0.359 |
| 2017 | 2-Jun-17 | MONTEREY | RASPBERRY (ALL OR UNSPEC) | Redacted2 | 0.1249 | 0.32 | A | G | 0.359 |
| 2017 | 18-May-17 | MONTEREY | RASPBERRY (ALL OR UNSPEC) | Redacted2 | 2.1654 | 5.55 | A | G | 0.359 |
| 2017 | 13-Jul-17 | MONTEREY | RASPBERRY (ALL OR UNSPEC) | Redacted2 | 5.0803 | 13.04 | A | G | 0.358 |
| 2017 | 12-Jul-17 | MONTEREY | RASPBERRY (ALL OR UNSPEC) | Redacted2 | 2.4152 | 6.26 | A | G | 0.355 |
| 2017 | 28-Apr-17 | MONTEREY | RASPBERRY (ALL OR UNSPEC) | Redacted2 | 0.4164 | 1.08 | A | G | 0.355 |
| 2017 | 18-May-17 | MONTEREY | RASPBERRY (ALL OR UNSPEC) | Redacted2 | 0.7496 | 2 | A | G | 0.345 |
| 2017 | 5-Apr-17 | SANTA CRUZ | RASPBERRY (ALL OR UNSPEC) | Redacted2 | 0.354 | 1.37 | A | G | 0.238 |
| 2017 | 6-Apr-17 | SANTA CRUZ | RASPBERRY (ALL OR UNSPEC) | Redacted2 | 0.5218 | 2.02 | A | G | 0.238 |
| 2017 | 6-Apr-17 | SANTA CRUZ | RASPBERRY (ALL OR UNSPEC) | Redacted2 | 0.7645 | 2.96 | A | G | 0.238 |
| 2017 | 6-Apr-17 | SANTA CRUZ | RASPBERRY (ALL OR UNSPEC) | Redacted2 | 0.5062 | 1.96 | A | G | 0.238 |
| 2017 | 5-Apr-17 | SANTA CRUZ | RASPBERRY (ALL OR UNSPEC) | Redacted2 | 0.408 | 1.58 | A | G | 0.238 |
| 2017 | 6-Apr-17 | SANTA CRUZ | RASPBERRY (ALL OR UNSPEC) | Redacted2 | 1.7145 | 6.64 | A | G | 0.238 |
| 2017 | 5-Apr-17 | SANTA CRUZ | RASPBERRY (ALL OR UNSPEC) | Redacted2 | 3.5012 | 13.56 | A | G | 0.238 |
| 2017 | 6-Apr-17 | SANTA CRUZ | RASPBERRY (ALL OR UNSPEC) | Redacted2 | 0.4828 | 1.87 | A | G | 0.238 |
| 2017 | 5-Apr-17 | SANTA CRUZ | RASPBERRY (ALL OR UNSPEC) | Redacted2 | 0.568 | 2.2 | A | G | 0.238 |
| 2017 | 5-Apr-17 | SANTA CRUZ | RASPBERRY (ALL OR UNSPEC) | Redacted2 | 1.3761 | 5.33 | A | G | 0.238 |
| 2017 | 6-Apr-17 | SANTA CRUZ | RASPBERRY (ALL OR UNSPEC) | Redacted2 | 0.7229 | 2.8 | A | G | 0.238 |
| 2017 | 6-Apr-17 | SANTA CRUZ | RASPBERRY (ALL OR UNSPEC) | Redacted2 | 0.4776 | 1.85 | A | G | 0.238 |
| 2017 | 6-Apr-17 | SANTA CRUZ | RASPBERRY (ALL OR UNSPEC) | Redacted2 | 0.7073 | 2.74 | A | G | 0.237 |
| 2017 | 6-Apr-17 | SANTA CRUZ | RASPBERRY (ALL OR UNSPEC) | Redacted2 | 0.6376 | 2.47 | A | G | 0.237 |
| 2017 | 6-Apr-17 | SANTA CRUZ | RASPBERRY (ALL OR UNSPEC) | Redacted2 | 0.2349 | 0.91 | A | G | 0.237 |
| 2017 | 6-Apr-17 | SANTA CRUZ | RASPBERRY (ALL OR UNSPEC) | Redacted2 | 0.6324 | 2.45 | A | G | 0.237 |
| 2017 | 6-Apr-17 | SANTA CRUZ | RASPBERRY (ALL OR UNSPEC) | Redacted2 | 0.9369 | 3.63 | A | G | 0.237 |
| 2017 | 5-Apr-17 | SANTA CRUZ | RASPBERRY (ALL OR UNSPEC) | Redacted2 | 0.3767 | 1.46 | A | G | 0.237 |
| 2017 | 6-Apr-17 | SANTA CRUZ | RASPBERRY (ALL OR UNSPEC) | Redacted2 | 0.2967 | 1.15 | A | G | 0.237 |
| 2017 | 22-Apr-17 | MONTEREY | RASPBERRY (ALL OR UNSPEC) | Redacted2 | 0.1666 | 1.72 | A | G | 0.089 |
| 2017 | 20-Mar-17 | SANTA CRUZ | RASPBERRY (ALL OR UNSPEC) | Redacted2 | 0.0833 | 0.91 | A | G | 0.084 |
| 2017 | 25-Feb-17 | SANTA CRUZ | RASPBERRY (ALL OR UNSPEC) | Redacted2 | 0.0833 | 0.91 | A | G | 0.084 |
| 2017 | 25-Feb-17 | SANTA CRUZ | RASPBERRY (ALL OR UNSPEC) | Redacted2 | 0.1666 | 1.85 | A | G | 0.083 |
| 2017 | 20-Mar-17 | SANTA CRUZ | RASPBERRY (ALL OR UNSPEC) | Redacted2 | 0.1666 | 1.85 | A | G | 0.083 |
| 2017 | 25-Feb-17 | SANTA CRUZ | RASPBERRY (ALL OR UNSPEC) | Redacted2 | 0.2499 | 2.8 | A | G | 0.082 |
| 2017 | 20-Mar-17 | SANTA CRUZ | RASPBERRY (ALL OR UNSPEC) | Redacted2 | 0.2499 | 2.8 | A | G | 0.082 |
| 2017 | 25-Feb-17 | SANTA CRUZ | RASPBERRY (ALL OR UNSPEC) | Redacted2 | 0.1666 | 1.87 | A | G | 0.082 |
| 2017 | 25-Feb-17 | SANTA CRUZ | RASPBERRY (ALL OR UNSPEC) | Redacted2 | 0.1666 | 1.96 | A | G | 0.078 |
| 2017 | 20-Mar-17 | SANTA CRUZ | RASPBERRY (ALL OR UNSPEC) | Redacted2 | 0.1666 | 1.96 | A | G | 0.078 |
| 2017 | 20-Mar-17 | SANTA CRUZ | RASPBERRY (ALL OR UNSPEC) | Redacted2 | 0.2499 | 2.96 | A | G | 0.078 |
| 2017 | 20-Mar-17 | SANTA CRUZ | RASPBERRY (ALL OR UNSPEC) | Redacted2 | 0.2499 | 2.98 | A | G | 0.077 |
| 2017 | 25-Feb-17 | SANTA CRUZ | RASPBERRY (ALL OR UNSPEC) | Redacted2 | 0.1666 | 2.02 | A | G | 0.076 |
| 2017 | 20-Mar-17 | SANTA CRUZ | RASPBERRY (ALL OR UNSPEC) | Redacted2 | 0.1666 | 2.02 | A | G | 0.076 |
| 2017 | 28-Mar-17 | MONTEREY | RASPBERRY (ALL OR UNSPEC) | Redacted2 | 0.583 | 7.22 | A | G | 0.074 |
| 2017 | 3-Mar-17 | MONTEREY | RASPBERRY (ALL OR UNSPEC) | Redacted2 | 0.583 | 7.22 | A | G | 0.074 |
| 2017 | 4-Mar-17 | MONTEREY | RASPBERRY (ALL OR UNSPEC) | Redacted2 | 0.583 | 7.37 | A | G | 0.073 |
| 2017 | 28-Mar-17 | MONTEREY | RASPBERRY (ALL OR UNSPEC) | Redacted2 | 0.583 | 7.37 | A | G | 0.073 |
| 2017 | 15-May-17 | SANTA CRUZ | RASPBERRY (ALL OR UNSPEC) | Redacted2 | 0.583 | 7.37 | A | G | 0.073 |
| 2017 | 4-Mar-17 | MONTEREY | RASPBERRY (ALL OR UNSPEC) | Redacted2 | 0.583 | 7.4 | A | G | 0.072 |
| 2017 | 28-Mar-17 | MONTEREY | RASPBERRY (ALL OR UNSPEC) | Redacted2 | 0.583 | 7.4 | A | G | 0.072 |
| 2017 | 10-Mar-17 | SANTA CRUZ | RASPBERRY (ALL OR UNSPEC) | Redacted2 | 0.2499 | 3.19 | A | G | 0.072 |
| 2017 | 31-Mar-17 | SANTA CRUZ | RASPBERRY (ALL OR UNSPEC) | Redacted2 | 0.2499 | 3.19 | A | G | 0.072 |
| 2017 | 9-Mar-17 | SANTA CRUZ | RASPBERRY (ALL OR UNSPEC) | Redacted2 | 0.4164 | 5.33 | A | G | 0.072 |
| 2017 | 28-Mar-17 | MONTEREY | RASPBERRY (ALL OR UNSPEC) | Redacted2 | 0.583 | 7.49 | A | G | 0.072 |
| 2017 | 3-Mar-17 | MONTEREY | RASPBERRY (ALL OR UNSPEC) | Redacted2 | 0.583 | 7.49 | A | G | 0.072 |
| 2017 | 3-Mar-17 | MONTEREY | RASPBERRY (ALL OR UNSPEC) | Redacted2 | 0.583 | 7.49 | A | G | 0.072 |
| 2017 | 28-Mar-17 | MONTEREY | RASPBERRY (ALL OR UNSPEC) | Redacted2 | 0.583 | 7.49 | A | G | 0.072 |
| 2017 | 31-Mar-17 | SANTA CRUZ | RASPBERRY (ALL OR UNSPEC) | Redacted2 | 0.7496 | 9.66 | A | G | 0.071 |
| 2017 | 9-Mar-17 | SANTA CRUZ | RASPBERRY (ALL OR UNSPEC) | Redacted2 | 0.4164 | 5.38 | A | G | 0.071 |
| 2017 | 20-Mar-17 | SANTA CRUZ | RASPBERRY (ALL OR UNSPEC) | Redacted2 | 2.1654 | 28.22 | A | G | 0.071 |
| 2017 | 4-Mar-17 | MONTEREY | RASPBERRY (ALL OR UNSPEC) | Redacted2 | 0.583 | 7.67 | A | G | 0.070 |
| 2017 | 28-Mar-17 | MONTEREY | RASPBERRY (ALL OR UNSPEC) | Redacted2 | 0.583 | 7.67 | A | G | 0.070 |
| 2017 | 15-May-17 | SANTA CRUZ | RASPBERRY (ALL OR UNSPEC) | Redacted2 | 0.583 | 7.67 | A | G | 0.070 |
| 2017 | 22-Apr-17 | MONTEREY | RASPBERRY (ALL OR UNSPEC) | Redacted2 | 0.2499 | 3.3 | A | G | 0.070 |
| 2017 | 9-Mar-17 | SANTA CRUZ | RASPBERRY (ALL OR UNSPEC) | Redacted2 | 0.1666 | 2.2 | A | G | 0.070 |
| 2017 | 28-Mar-17 | MONTEREY | RASPBERRY (ALL OR UNSPEC) | Redacted2 | 0.4997 | 6.62 | A | G | 0.069 |
| 2017 | 4-Mar-17 | MONTEREY | RASPBERRY (ALL OR UNSPEC) | Redacted2 | 0.4997 | 6.62 | A | G | 0.069 |
| 2017 | 31-Mar-17 | SANTA CRUZ | RASPBERRY (ALL OR UNSPEC) | Redacted2 | 0.6663 | 8.84 | A | G | 0.069 |
| 2017 | 22-Apr-17 | MONTEREY | RASPBERRY (ALL OR UNSPEC) | Redacted2 | 0.2499 | 3.32 | A | G | 0.069 |
| 2017 | 4-Mar-17 | MONTEREY | RASPBERRY (ALL OR UNSPEC) | Redacted2 | 0.4997 | 6.64 | A | G | 0.069 |
| 2017 | 28-Mar-17 | MONTEREY | RASPBERRY (ALL OR UNSPEC) | Redacted2 | 0.4997 | 6.64 | A | G | 0.069 |
| 2017 | 25-Feb-17 | SANTA CRUZ | RASPBERRY (ALL OR UNSPEC) | Redacted2 | 0.4997 | 6.64 | A | G | 0.069 |
| 2017 | 20-Mar-17 | SANTA CRUZ | RASPBERRY (ALL OR UNSPEC) | Redacted2 | 0.4997 | 6.64 | A | G | 0.069 |
| 2017 | 31-Mar-17 | SANTA CRUZ | RASPBERRY (ALL OR UNSPEC) | Redacted2 | 0.2499 | 3.38 | A | G | 0.068 |
| 2017 | 28-Mar-17 | MONTEREY | RASPBERRY (ALL OR UNSPEC) | Redacted2 | 0.4997 | 6.76 | A | G | 0.068 |
| 2017 | 3-Mar-17 | MONTEREY | RASPBERRY (ALL OR UNSPEC) | Redacted2 | 0.4997 | 6.76 | A | G | 0.068 |
| 2017 | 10-Mar-17 | SANTA CRUZ | RASPBERRY (ALL OR UNSPEC) | Redacted2 | 0.583 | 7.94 | A | G | 0.068 |
| 2017 | 10-Mar-17 | SANTA CRUZ | RASPBERRY (ALL OR UNSPEC) | Redacted2 | 0.4164 | 5.68 | A | G | 0.067 |
| 2017 | 10-Mar-17 | SANTA CRUZ | RASPBERRY (ALL OR UNSPEC) | Redacted2 | 0.4997 | 6.84 | A | G | 0.067 |
| 2017 | 25-Feb-17 | SANTA CRUZ | RASPBERRY (ALL OR UNSPEC) | Redacted2 | 0.0833 | 1.15 | A | G | 0.067 |
| 2017 | 28-Mar-17 | MONTEREY | RASPBERRY (ALL OR UNSPEC) | Redacted2 | 0.4997 | 6.92 | A | G | 0.066 |
| 2017 | 4-Mar-17 | MONTEREY | RASPBERRY (ALL OR UNSPEC) | Redacted2 | 0.4997 | 6.92 | A | G | 0.066 |
| 2017 | 4-Mar-17 | MONTEREY | RASPBERRY (ALL OR UNSPEC) | Redacted2 | 0.1666 | 2.32 | A | G | 0.066 |
| 2017 | 28-Mar-17 | MONTEREY | RASPBERRY (ALL OR UNSPEC) | Redacted2 | 0.1666 | 2.32 | A | G | 0.066 |
| 2017 | 22-Apr-17 | MONTEREY | RASPBERRY (ALL OR UNSPEC) | Redacted2 | 0.1666 | 2.33 | A | G | 0.066 |
| 2017 | 20-Mar-17 | SANTA CRUZ | RASPBERRY (ALL OR UNSPEC) | Redacted2 | 0.2499 | 3.63 | A | G | 0.063 |
| 2017 | 25-Feb-17 | SANTA CRUZ | RASPBERRY (ALL OR UNSPEC) | Redacted2 | 0.2499 | 3.63 | A | G | 0.063 |
| 2017 | 22-Apr-17 | SANTA CRUZ | RASPBERRY (ALL OR UNSPEC) | Redacted2 | 0.2499 | 3.66 | A | G | 0.063 |
| 2017 | 25-Feb-17 | SANTA CRUZ | RASPBERRY (ALL OR UNSPEC) | Redacted2 | 0.1666 | 2.45 | A | G | 0.063 |
| 2017 | 21-Apr-17 | MONTEREY | RASPBERRY (ALL OR UNSPEC) | Redacted2 | 0.2499 | 3.7 | A | G | 0.062 |
| 2017 | 25-Feb-17 | SANTA CRUZ | RASPBERRY (ALL OR UNSPEC) | Redacted2 | 0.1666 | 2.47 | A | G | 0.062 |
| 2017 | 20-Mar-17 | SANTA CRUZ | RASPBERRY (ALL OR UNSPEC) | Redacted2 | 0.1666 | 2.47 | A | G | 0.062 |
| 2017 | 21-Apr-17 | MONTEREY | RASPBERRY (ALL OR UNSPEC) | Redacted2 | 0.2499 | 3.71 | A | G | 0.062 |
| 2017 | 22-Apr-17 | SANTA CRUZ | RASPBERRY (ALL OR UNSPEC) | Redacted2 | 0.2499 | 3.71 | A | G | 0.062 |
| 2017 | 9-Mar-17 | SANTA CRUZ | RASPBERRY (ALL OR UNSPEC) | Redacted2 | 0.0833 | 1.37 | A | G | 0.056 |
| 2017 | 9-Mar-17 | SANTA CRUZ | RASPBERRY (ALL OR UNSPEC) | Redacted2 | 0.0833 | 1.42 | A | G | 0.054 |
| 2017 | 9-Mar-17 | SANTA CRUZ | RASPBERRY (ALL OR UNSPEC) | Redacted2 | 0.0833 | 1.46 | A | G | 0.052 |
| 2017 | 9-Mar-17 | SANTA CRUZ | RASPBERRY (ALL OR UNSPEC) | Redacted2 | 0.0833 | 1.58 | A | G | 0.049 |
| 2017 | 14-Dec-17 | SAN DIEGO | STRAWBERRY (ALL OR UNSPEC) | Redacted2 | 1.5616 | 5 | A | G | 0.287 |
| 2017 | 5-Apr-17 | MONTEREY | STRAWBERRY (ALL OR UNSPEC) | Redacted2 | 2.2487 | 7.2 | A | G | 0.287 |
| 2017 | 5-Apr-17 | MONTEREY | STRAWBERRY (ALL OR UNSPEC) | Redacted2 | 2.4152 | 7.8 | A | G | 0.285 |
| 2017 | 5-Apr-17 | MONTEREY | STRAWBERRY (ALL OR UNSPEC) | Redacted2 | 3.0815 | 10 | A | G | 0.283 |
| 2017 | 1-Apr-17 | MONTEREY | STRAWBERRY (ALL OR UNSPEC) | Redacted2 | 0.9161 | 3.4 | A | G | 0.248 |
| 2017 | 14-Apr-17 | MONTEREY | STRAWBERRY (ALL OR UNSPEC) | Redacted2 | 0.6663 | 2.5 | A | G | 0.245 |
| 2017 | 14-Apr-17 | MONTEREY | STRAWBERRY (ALL OR UNSPEC) | Redacted2 | 0.6663 | 2.5 | A | G | 0.245 |
| 2017 | 14-Apr-17 | MONTEREY | STRAWBERRY (ALL OR UNSPEC) | Redacted2 | 0.6663 | 2.5 | A | G | 0.245 |
| 2017 | 14-Apr-17 | MONTEREY | STRAWBERRY (ALL OR UNSPEC) | Redacted2 | 0.6663 | 2.5 | A | G | 0.245 |
| 2017 | 22-Apr-17 | MONTEREY | STRAWBERRY (ALL OR UNSPEC) | Redacted2 | 1.5824 | 6 | A | G | 0.243 |
| 2017 | 4-Apr-17 | MONTEREY | STRAWBERRY (ALL OR UNSPEC) | Redacted2 | 1.4991 | 5.7 | A | G | 0.242 |
| 2017 | 3-Apr-17 | MONTEREY | STRAWBERRY (ALL OR UNSPEC) | Redacted2 | 3.5812 | 13.77 | A | G | 0.239 |
| 2017 | 4-Apr-17 | MONTEREY | STRAWBERRY (ALL OR UNSPEC) | Redacted2 | 9.994 | 38.64 | A | G | 0.238 |
| 2017 | 3-Apr-17 | MONTEREY | STRAWBERRY (ALL OR UNSPEC) | Redacted2 | 7.4955 | 29 | A | G | 0.238 |
| 2017 | 17-Mar-17 | MONTEREY | STRAWBERRY (ALL OR UNSPEC) | Redacted2 | 2.5818 | 10 | A | G | 0.238 |
| 2017 | 3-Apr-17 | MONTEREY | STRAWBERRY (ALL OR UNSPEC) | Redacted2 | 6.5794 | 25.5 | A | G | 0.237 |
| 2017 | 6-Apr-17 | MONTEREY | STRAWBERRY (ALL OR UNSPEC) | Redacted2 | 9.6609 | 37.5 | A | G | 0.237 |
| 2017 | 5-Apr-17 | MONTEREY | STRAWBERRY (ALL OR UNSPEC) | Redacted2 | 9.6609 | 37.5 | A | G | 0.237 |
| 2017 | 3-Apr-17 | MONTEREY | STRAWBERRY (ALL OR UNSPEC) | Redacted2 | 13.0755 | 50.78 | A | G | 0.237 |
| 2017 | 17-Mar-17 | MONTEREY | STRAWBERRY (ALL OR UNSPEC) | Redacted2 | 1.9988 | 7.8 | A | G | 0.236 |
| 2017 | 4-Apr-17 | MONTEREY | STRAWBERRY (ALL OR UNSPEC) | Redacted2 | 1.4991 | 5.89 | A | G | 0.234 |
| 2017 | 17-Mar-17 | MONTEREY | STRAWBERRY (ALL OR UNSPEC) | Redacted2 | 1.8322 | 7.2 | A | G | 0.234 |
| 2017 | 4-Apr-17 | MONTEREY | STRAWBERRY (ALL OR UNSPEC) | Redacted2 | 1.4158 | 5.58 | A | G | 0.233 |
| 2017 | 1-Apr-17 | MONTEREY | STRAWBERRY (ALL OR UNSPEC) | Redacted2 | 0.8328 | 3.3 | A | G | 0.232 |
| 2017 | 1-Apr-17 | MONTEREY | STRAWBERRY (ALL OR UNSPEC) | Redacted2 | 0.8328 | 3.3 | A | G | 0.232 |
| 2017 | 16-Mar-17 | MONTEREY | STRAWBERRY (ALL OR UNSPEC) | Redacted2 | 0.7496 | 3 | A | G | 0.230 |
| 2017 | 16-Mar-17 | MONTEREY | STRAWBERRY (ALL OR UNSPEC) | Redacted2 | 0.7496 | 3 | A | G | 0.230 |
| 2017 | 16-Mar-17 | MONTEREY | STRAWBERRY (ALL OR UNSPEC) | Redacted2 | 0.7496 | 3 | A | G | 0.230 |
| 2017 | 16-Mar-17 | MONTEREY | STRAWBERRY (ALL OR UNSPEC) | Redacted2 | 0.7496 | 3 | A | G | 0.230 |
| 2017 | 4-Apr-17 | MONTEREY | STRAWBERRY (ALL OR UNSPEC) | Redacted2 | 0.4997 | 2 | A | G | 0.230 |
| 2017 | 4-Apr-17 | MONTEREY | STRAWBERRY (ALL OR UNSPEC) | Redacted2 | 0.4997 | 2 | A | G | 0.230 |
| 2017 | 4-Apr-17 | MONTEREY | STRAWBERRY (ALL OR UNSPEC) | Redacted2 | 0.4997 | 2 | A | G | 0.230 |
| 2017 | 4-Apr-17 | MONTEREY | STRAWBERRY (ALL OR UNSPEC) | Redacted2 | 0.4997 | 2 | A | G | 0.230 |
| 2017 | 4-Apr-17 | MONTEREY | STRAWBERRY (ALL OR UNSPEC) | Redacted2 | 0.4997 | 2 | A | G | 0.230 |
| 2017 | 28-Dec-17 | SAN DIEGO | STRAWBERRY (ALL OR UNSPEC) | Redacted2 | 1.5616 | 10 | A | G | 0.144 |
| 2017 | 6-Apr-17 | STANISLAUS | WALNUT (ENGLISH WALNUT, PERSIAN WALNUT) | Redacted2 | 3.7478 | 18 | A | G | 0.192 |
| 2017 | 6-Apr-17 | STANISLAUS | WALNUT (ENGLISH WALNUT, PERSIAN WALNUT) | Redacted2 | 3.7478 | 18 | A | G | 0.192 |
| 2017 | 30-Apr-17 | STANISLAUS | WALNUT (ENGLISH WALNUT, PERSIAN WALNUT) | Redacted2 | 3.7478 | 18 | A | G | 0.192 |
| 2017 | 30-Apr-17 | STANISLAUS | WALNUT (ENGLISH WALNUT, PERSIAN WALNUT) | Redacted2 | 9.5776 | 46 | A | G | 0.192 |
| 2017 | 5-Apr-17 | STANISLAUS | WALNUT (ENGLISH WALNUT, PERSIAN WALNUT) | Redacted2 | 7.4955 | 36 | A | G | 0.192 |
| 2017 | 30-Apr-17 | STANISLAUS | WALNUT (ENGLISH WALNUT, PERSIAN WALNUT) | Redacted2 | 7.4955 | 36 | A | G | 0.192 |
| 2017 | 6-Apr-17 | STANISLAUS | WALNUT (ENGLISH WALNUT, PERSIAN WALNUT) | Redacted2 | 5.1636 | 25 | A | G | 0.190 |
| 2017 | 5-Apr-17 | STANISLAUS | WALNUT (ENGLISH WALNUT, PERSIAN WALNUT) | Redacted2 | 4.7472 | 23 | A | G | 0.190 |
| 2017 | 6-Apr-17 | BUTTE | WALNUT (ENGLISH WALNUT, PERSIAN WALNUT) | Redacted2 | 1.952 | 10 | A | G | 0.180 |
| 2017 | 18-Jun-17 | TEHAMA | WALNUT (ENGLISH WALNUT, PERSIAN WALNUT) | Redacted2 | 8.7448 | 45 | A | G | 0.179 |
| 2017 | 31-May-17 | TEHAMA | WALNUT (ENGLISH WALNUT, PERSIAN WALNUT) | Redacted2 | 8.7448 | 45 | A | G | 0.179 |
| 2017 | 4-Apr-17 | BUTTE | WALNUT (ENGLISH WALNUT, PERSIAN WALNUT) | Redacted2 | 3.831 | 20 | A | G | 0.176 |
| 2017 | 4-Apr-17 | COLUSA | WALNUT (ENGLISH WALNUT, PERSIAN WALNUT) | Redacted2 | 8.9946 | 47 | A | G | 0.176 |
| 2017 | 11-Apr-17 | COLUSA | WALNUT (ENGLISH WALNUT, PERSIAN WALNUT) | Redacted2 | 8.9946 | 47 | A | G | 0.176 |
| 2017 | 26-Apr-17 | STANISLAUS | WALNUT (ENGLISH WALNUT, PERSIAN WALNUT) | Redacted2 | 4.1642 | 25 | A | G | 0.153 |
| 2017 | 26-Apr-17 | STANISLAUS | WALNUT (ENGLISH WALNUT, PERSIAN WALNUT) | Redacted2 | 2.9982 | 18 | A | G | 0.153 |
| 2017 | 26-Apr-17 | STANISLAUS | WALNUT (ENGLISH WALNUT, PERSIAN WALNUT) | Redacted2 | 3.3313 | 20 | A | G | 0.153 |
| 2017 | 4-Oct-17 | TEHAMA | WALNUT (ENGLISH WALNUT, PERSIAN WALNUT) | Redacted2 | 5.2469 | 90 | A | A | 0.054 |

| YEAR | DATE | COUNTY_NAME | SITE_NAME | PRODUCT_NAME | POUNDS_PRODUCT_APPLIED | AMOUNT_TREATED | UNIT_TREATED | AERIAL_GROUND_INDICATOR | Application Rate (lb/A) |
| --- | --- | --- | --- | --- | --- | --- | --- | --- | --- |
| 2017 | 6-May-17 | KERN | CHERRY | Redacted3 | 14.949 | 76 | A | G | 0.196697368 |
| 2017 | 12-May-17 | KERN | ONION (DRY, SPANISH, WHITE, YELLOW, RED, ETC.) | Redacted3 | 17.8884 | 68 | A | A | 0.263064706 |
| 2017 | 30-Aug-17 | MADERA | PISTACHIO (PISTACHE NUT) | Redacted3 | 2.2675 | 10 | A | A | 0.22675 |
| 2017 | 30-Aug-17 | MADERA | PISTACHIO (PISTACHE NUT) | Redacted3 | 1.1758 | 5 | A | A | 0.23516 |
| 2017 | 8-Jul-17 | FRESNO | TOMATOES, FOR PROCESSING/CANNING | Redacted3 | 13.1014 | 40 | A | A | 0.327535 |

| YEAR | DATE | COUNTY_NAME | SITE_NAME | PRODUCT_NAME | POUNDS_PRODUCT_APPLIED | AMOUNT_TREATED | UNIT_TREATED | AERIAL_GROUND_INDICATOR | Application Rate (lb/A) |
| --- | --- | --- | --- | --- | --- | --- | --- | --- | --- |
| 2017 | 5-Jul-17 | FRESNO | ALMOND | Redacted4 | 5.5429 | 30 | A | G | 0.0924 |
| 2017 | 5-Jul-17 | FRESNO | ALMOND | Redacted4 | 19.1481 | 103 | A | G | 0.0930 |
| 2017 | 5-Jul-17 | FRESNO | ALMOND | Redacted4 | 17.3005 | 93 | A | G | 0.0930 |
| 2017 | 8-Jul-17 | FRESNO | ALMOND | Redacted4 | 16.0408 | 86 | A | G | 0.0933 |
| 2017 | 8-Jul-17 | FRESNO | ALMOND | Redacted4 | 8.3983 | 45 | A | G | 0.0933 |
| 2017 | 30-Jul-17 | FRESNO | ALMOND | Redacted4 | 14.4116 | 76.8 | A | G | 0.0938 |
| 2017 | 30-Jul-17 | FRESNO | ALMOND | Redacted4 | 5.6748 | 30.24 | A | G | 0.0938 |
| 2017 | 27-Jul-17 | KERN | ALMOND | Redacted4 | 3.8632 | 20 | A | G | 0.0966 |
| 2017 | 8-Sep-17 | FRESNO | ALMOND | Redacted4 | 15.2849 | 78 | A | A | 0.0980 |
| 2017 | 31-Aug-17 | FRESNO | ALMOND | Redacted4 | 19.82 | 101 | A | A | 0.0981 |
| 2017 | 8-Sep-17 | FRESNO | ALMOND | Redacted4 | 15.7048 | 80 | A | A | 0.0982 |
| 2017 | 27-Jul-17 | KERN | ALMOND | Redacted4 | 15.7048 | 80 | A | G | 0.0982 |
| 2017 | 5-Sep-17 | FRESNO | ALMOND | Redacted4 | 27.8824 | 142 | A | A | 0.0982 |
| 2017 | 31-Aug-17 | FRESNO | ALMOND | Redacted4 | 24.943 | 127 | A | A | 0.0982 |
| 2017 | 31-Aug-17 | FRESNO | ALMOND | Redacted4 | 30.6538 | 156 | A | A | 0.0982 |
| 2017 | 29-Jul-17 | KERN | ALMOND | Redacted4 | 9.826 | 50 | A | G | 0.0983 |
| 2017 | 29-Jul-17 | KERN | ALMOND | Redacted4 | 49.1301 | 250 | A | G | 0.0983 |
| 2017 | 5-Sep-17 | FRESNO | ALMOND | Redacted4 | 17.8884 | 91 | A | A | 0.0983 |
| 2017 | 24-Jul-17 | FRESNO | ALMOND | Redacted4 | 10.4198 | 53 | A | G | 0.0983 |
| 2017 | 24-Jul-17 | FRESNO | ALMOND | Redacted4 | 19.8567 | 101 | A | G | 0.0983 |
| 2017 | 24-Jul-17 | FRESNO | ALMOND | Redacted4 | 32.8328 | 167 | A | G | 0.0983 |
| 2017 | 24-Jul-17 | FRESNO | ALMOND | Redacted4 | 20.6434 | 105 | A | G | 0.0983 |
| 2017 | 31-Aug-17 | FRESNO | ALMOND | Redacted4 | 32.8374 | 167 | A | A | 0.0983 |
| 2017 | 8-Sep-17 | FRESNO | ALMOND | Redacted4 | 30.4859 | 155 | A | A | 0.0983 |
| 2017 | 8-Sep-17 | FRESNO | ALMOND | Redacted4 | 30.4859 | 155 | A | A | 0.0983 |
| 2017 | 5-Sep-17 | FRESNO | ALMOND | Redacted4 | 35.6088 | 181 | A | A | 0.0984 |
| 2017 | 8-Sep-17 | FRESNO | ALMOND | Redacted4 | 27.5464 | 140 | A | A | 0.0984 |
| 2017 | 31-Aug-17 | FRESNO | ALMOND | Redacted4 | 20.6598 | 105 | A | A | 0.0984 |
| 2017 | 31-Aug-17 | FRESNO | ALMOND | Redacted4 | 19.8788 | 101 | A | A | 0.0984 |
| 2017 | 31-Aug-17 | FRESNO | ALMOND | Redacted4 | 30.7042 | 156 | A | A | 0.0984 |
| 2017 | 31-Aug-17 | FRESNO | ALMOND | Redacted4 | 32.871 | 167 | A | A | 0.0984 |
| 2017 | 22-Jul-17 | FRESNO | ALMOND | Redacted4 | 14.1721 | 72 | A | G | 0.0984 |
| 2017 | 22-Jul-17 | FRESNO | ALMOND | Redacted4 | 28.1474 | 143 | A | G | 0.0984 |
| 2017 | 5-Sep-17 | FRESNO | ALMOND | Redacted4 | 31.4936 | 160 | A | A | 0.0984 |
| 2017 | 25-Jul-17 | FRESNO | ALMOND | Redacted4 | 30.7063 | 156 | A | G | 0.0984 |
| 2017 | 22-Jul-17 | FRESNO | ALMOND | Redacted4 | 24.9981 | 127 | A | G | 0.0984 |
| 2017 | 31-Aug-17 | FRESNO | ALMOND | Redacted4 | 20.6682 | 105 | A | A | 0.0984 |
| 2017 | 31-Aug-17 | FRESNO | ALMOND | Redacted4 | 28.1511 | 143 | A | A | 0.0984 |
| 2017 | 31-Aug-17 | FRESNO | ALMOND | Redacted4 | 25.0018 | 127 | A | A | 0.0984 |
| 2017 | 28-Jul-17 | FRESNO | ALMOND | Redacted4 | 3.9472 | 20 | A | G | 0.0987 |
| 2017 | 26-Jul-17 | FRESNO | ALMOND | Redacted4 | 14.1721 | 71 | A | G | 0.0998 |
| 2017 | 6-Jul-17 | FRESNO | ALMOND | Redacted4 | 33.9292 | 152 | A | G | 0.1116 |
| 2017 | 31-Aug-17 | FRESNO | ALMOND | Redacted4 | 8.3983 | 37.5 | A | G | 0.1120 |
| 2017 | 27-Aug-17 | FRESNO | ALMOND | Redacted4 | 7.1701 | 32 | A | G | 0.1120 |
| 2017 | 27-Aug-17 | FRESNO | ALMOND | Redacted4 | 17.9258 | 80 | A | G | 0.1120 |
| 2017 | 12-Jul-17 | FRESNO | ALMOND | Redacted4 | 1.4277 | 6.36 | A | G | 0.1122 |
| 2017 | 29-Jul-17 | MADERA | ALMOND | Redacted4 | 10.1042 | 45 | A | G | 0.1123 |
| 2017 | 21-Jul-17 | MADERA | ALMOND | Redacted4 | 4.5351 | 20 | A | G | 0.1134 |
| 2017 | 27-Jul-17 | FRESNO | ALMOND | Redacted4 | 11.4217 | 50 | A | G | 0.1142 |
| 2017 | 25-Apr-17 | FRESNO | ALMOND | Redacted4 | 11.4217 | 50 | A | G | 0.1142 |
| 2017 | 7-Oct-17 | FRESNO | ALMOND | Redacted4 | 2.3515 | 10.29 | A | G | 0.1143 |
| 2017 | 25-Jul-17 | FRESNO | ALMOND | Redacted4 | 13.2693 | 58 | A | G | 0.1144 |
| 2017 | 22-Jul-17 | FRESNO | ALMOND | Redacted4 | 9.1542 | 40 | A | G | 0.1144 |
| 2017 | 21-Jul-17 | FRESNO | ALMOND | Redacted4 | 9.1542 | 40 | A | G | 0.1144 |
| 2017 | 28-Aug-17 | FRESNO | ALMOND | Redacted4 | 9.1542 | 40 | A | G | 0.1144 |
| 2017 | 28-Aug-17 | FRESNO | ALMOND | Redacted4 | 9.1542 | 40 | A | G | 0.1144 |
| 2017 | 24-Apr-17 | FRESNO | ALMOND | Redacted4 | 17.4685 | 76.3 | A | G | 0.1145 |
| 2017 | 5-Jul-17 | FRESNO | ALMOND | Redacted4 | 17.4685 | 76.3 | A | G | 0.1145 |
| 2017 | 16-May-17 | FRESNO | ALMOND | Redacted4 | 9.994 | 43.64 | A | G | 0.1145 |
| 2017 | 29-Aug-17 | FRESNO | ALMOND | Redacted4 | 22.6754 | 99 | A | G | 0.1145 |
| 2017 | 22-Jul-17 | FRESNO | ALMOND | Redacted4 | 16.0408 | 70 | A | G | 0.1146 |
| 2017 | 15-Mar-17 | FRESNO | ALMOND | Redacted4 | 13.5213 | 59 | A | G | 0.1146 |
| 2017 | 17-Apr-17 | FRESNO | ALMOND | Redacted4 | 13.5213 | 59 | A | G | 0.1146 |
| 2017 | 24-Jul-17 | FRESNO | ALMOND | Redacted4 | 16.9646 | 74 | A | G | 0.1146 |
| 2017 | 1-Aug-17 | MERCED | ALMOND | Redacted4 | 8.4823 | 37 | A | G | 0.1146 |
| 2017 | 2-Aug-17 | MERCED | ALMOND | Redacted4 | 16.9646 | 74 | A | G | 0.1146 |
| 2017 | 17-Apr-17 | FRESNO | ALMOND | Redacted4 | 22.9274 | 100 | A | G | 0.1146 |
| 2017 | 15-Mar-17 | FRESNO | ALMOND | Redacted4 | 22.9274 | 100 | A | G | 0.1146 |
| 2017 | 28-Aug-17 | FRESNO | ALMOND | Redacted4 | 29.3941 | 128.2 | A | G | 0.1146 |
| 2017 | 2-Aug-17 | FRESNO | ALMOND | Redacted4 | 7.2225 | 31.5 | A | G | 0.1146 |
| 2017 | 18-Jul-17 | FRESNO | ALMOND | Redacted4 | 7.2225 | 31.5 | A | G | 0.1146 |
| 2017 | 18-Jul-17 | FRESNO | ALMOND | Redacted4 | 7.2225 | 31.5 | A | G | 0.1146 |
| 2017 | 16-May-17 | FRESNO | ALMOND | Redacted4 | 3.6113 | 15.75 | A | G | 0.1146 |
| 2017 | 14-Jul-17 | FRESNO | ALMOND | Redacted4 | 11.6736 | 50.91 | A | G | 0.1146 |
| 2017 | 25-Apr-17 | FRESNO | ALMOND | Redacted4 | 11.9256 | 52 | A | G | 0.1147 |
| 2017 | 26-Jul-17 | FRESNO | ALMOND | Redacted4 | 11.9256 | 52 | A | G | 0.1147 |
| 2017 | 24-Apr-17 | FRESNO | ALMOND | Redacted4 | 21.3317 | 93 | A | G | 0.1147 |
| 2017 | 25-Jul-17 | FRESNO | ALMOND | Redacted4 | 21.3317 | 93 | A | G | 0.1147 |
| 2017 | 18-Apr-17 | FRESNO | ALMOND | Redacted4 | 15.3689 | 67 | A | G | 0.1147 |
| 2017 | 25-Jul-17 | FRESNO | ALMOND | Redacted4 | 15.3689 | 67 | A | G | 0.1147 |
| 2017 | 16-Mar-17 | FRESNO | ALMOND | Redacted4 | 15.3689 | 67 | A | G | 0.1147 |
| 2017 | 1-Aug-17 | MADERA | ALMOND | Redacted4 | 15.3689 | 67 | A | G | 0.1147 |
| 2017 | 18-Apr-17 | FRESNO | ALMOND | Redacted4 | 34.853 | 151.9 | A | G | 0.1147 |
| 2017 | 24-Apr-17 | FRESNO | ALMOND | Redacted4 | 19.736 | 86 | A | G | 0.1147 |
| 2017 | 26-Jul-17 | FRESNO | ALMOND | Redacted4 | 19.736 | 86 | A | G | 0.1147 |
| 2017 | 15-Mar-17 | FRESNO | ALMOND | Redacted4 | 26.6226 | 116 | A | G | 0.1148 |
| 2017 | 17-Apr-17 | FRESNO | ALMOND | Redacted4 | 26.6226 | 116 | A | G | 0.1148 |
| 2017 | 18-Apr-17 | FRESNO | ALMOND | Redacted4 | 13.7732 | 60 | A | G | 0.1148 |
| 2017 | 24-Apr-17 | FRESNO | ALMOND | Redacted4 | 10.3299 | 45 | A | G | 0.1148 |
| 2017 | 26-Jul-17 | FRESNO | ALMOND | Redacted4 | 6.8866 | 30 | A | G | 0.1148 |
| 2017 | 17-Apr-17 | FRESNO | ALMOND | Redacted4 | 17.2165 | 75 | A | G | 0.1148 |
| 2017 | 2-May-17 | FRESNO | ALMOND | Redacted4 | 6.8866 | 30 | A | G | 0.1148 |
| 2017 | 16-Mar-17 | FRESNO | ALMOND | Redacted4 | 13.7732 | 60 | A | G | 0.1148 |
| 2017 | 15-Mar-17 | FRESNO | ALMOND | Redacted4 | 20.6598 | 90 | A | G | 0.1148 |
| 2017 | 4-Jul-17 | FRESNO | ALMOND | Redacted4 | 6.8866 | 30 | A | G | 0.1148 |
| 2017 | 15-Mar-17 | FRESNO | ALMOND | Redacted4 | 17.2165 | 75 | A | G | 0.1148 |
| 2017 | 24-Apr-17 | FRESNO | ALMOND | Redacted4 | 6.8866 | 30 | A | G | 0.1148 |
| 2017 | 5-Jul-17 | FRESNO | ALMOND | Redacted4 | 17.2165 | 75 | A | G | 0.1148 |
| 2017 | 25-Jul-17 | FRESNO | ALMOND | Redacted4 | 10.3299 | 45 | A | G | 0.1148 |
| 2017 | 17-Apr-17 | FRESNO | ALMOND | Redacted4 | 20.6598 | 90 | A | G | 0.1148 |
| 2017 | 18-Apr-17 | FRESNO | ALMOND | Redacted4 | 17.2165 | 75 | A | G | 0.1148 |
| 2017 | 24-Apr-17 | FRESNO | ALMOND | Redacted4 | 17.2165 | 75 | A | G | 0.1148 |
| 2017 | 16-Mar-17 | FRESNO | ALMOND | Redacted4 | 17.2165 | 75 | A | G | 0.1148 |
| 2017 | 15-Mar-17 | FRESNO | ALMOND | Redacted4 | 34.4331 | 150 | A | G | 0.1148 |
| 2017 | 17-Apr-17 | FRESNO | ALMOND | Redacted4 | 34.4331 | 150 | A | G | 0.1148 |
| 2017 | 17-Apr-17 | FRESNO | ALMOND | Redacted4 | 14.697 | 64 | A | G | 0.1148 |
| 2017 | 15-Mar-17 | FRESNO | ALMOND | Redacted4 | 14.697 | 64 | A | G | 0.1148 |
| 2017 | 17-Apr-17 | FRESNO | ALMOND | Redacted4 | 14.697 | 64 | A | G | 0.1148 |
| 2017 | 15-Mar-17 | FRESNO | ALMOND | Redacted4 | 14.697 | 64 | A | G | 0.1148 |
| 2017 | 18-Apr-17 | FRESNO | ALMOND | Redacted4 | 33.7612 | 147 | A | G | 0.1148 |
| 2017 | 17-Apr-17 | FRESNO | ALMOND | Redacted4 | 15.6208 | 68 | A | G | 0.1149 |
| 2017 | 15-Mar-17 | FRESNO | ALMOND | Redacted4 | 15.6208 | 68 | A | G | 0.1149 |
| 2017 | 15-Mar-17 | FRESNO | ALMOND | Redacted4 | 35.6088 | 155 | A | G | 0.1149 |
| 2017 | 17-Apr-17 | FRESNO | ALMOND | Redacted4 | 35.6088 | 155 | A | G | 0.1149 |
| 2017 | 16-Mar-17 | FRESNO | ALMOND | Redacted4 | 19.988 | 87 | A | G | 0.1149 |
| 2017 | 17-Apr-17 | FRESNO | ALMOND | Redacted4 | 19.988 | 87 | A | G | 0.1149 |
| 2017 | 18-Apr-17 | FRESNO | ALMOND | Redacted4 | 32.1655 | 140 | A | G | 0.1149 |
| 2017 | 24-Apr-17 | FRESNO | ALMOND | Redacted4 | 32.1655 | 140 | A | G | 0.1149 |
| 2017 | 5-Jul-17 | FRESNO | ALMOND | Redacted4 | 32.1655 | 140 | A | G | 0.1149 |
| 2017 | 21-Jul-17 | MADERA | ALMOND | Redacted4 | 17.2165 | 74.92 | A | G | 0.1149 |
| 2017 | 23-Aug-17 | FRESNO | ALMOND | Redacted4 | 10.9178 | 47.5 | A | G | 0.1149 |
| 2017 | 28-Aug-17 | FRESNO | ALMOND | Redacted4 | 22.7594 | 99 | A | G | 0.1149 |
| 2017 | 24-Apr-17 | FRESNO | ALMOND | Redacted4 | 23.6832 | 103 | A | G | 0.1150 |
| 2017 | 28-Aug-17 | FRESNO | ALMOND | Redacted4 | 29.4781 | 128.2 | A | G | 0.1150 |
| 2017 | 15-Mar-17 | FRESNO | ALMOND | Redacted4 | 19.3161 | 84 | A | G | 0.1150 |
| 2017 | 17-Apr-17 | FRESNO | ALMOND | Redacted4 | 19.3161 | 84 | A | G | 0.1150 |
| 2017 | 24-Sep-17 | FRESNO | ALMOND | Redacted4 | 28.9742 | 126 | A | G | 0.1150 |
| 2017 | 1-Aug-17 | MADERA | ALMOND | Redacted4 | 14.949 | 65 | A | G | 0.1150 |
| 2017 | 4-Jul-17 | FRESNO | ALMOND | Redacted4 | 22.0875 | 96 | A | G | 0.1150 |
| 2017 | 16-Mar-17 | FRESNO | ALMOND | Redacted4 | 16.7966 | 73 | A | G | 0.1150 |
| 2017 | 18-Apr-17 | FRESNO | ALMOND | Redacted4 | 16.7966 | 73 | A | G | 0.1150 |
| 2017 | 16-Mar-17 | FRESNO | ALMOND | Redacted4 | 17.7204 | 77 | A | G | 0.1151 |
| 2017 | 17-Apr-17 | FRESNO | ALMOND | Redacted4 | 17.7204 | 77 | A | G | 0.1151 |
| 2017 | 27-Jun-17 | FRESNO | ALMOND | Redacted4 | 3.0234 | 13.13 | A | G | 0.1151 |
| 2017 | 14-Jul-17 | FRESNO | ALMOND | Redacted4 | 4.6191 | 20.05 | A | G | 0.1152 |
| 2017 | 2-Aug-17 | FRESNO | ALMOND | Redacted4 | 9.4061 | 40.79 | A | G | 0.1153 |
| 2017 | 18-Jul-17 | FRESNO | ALMOND | Redacted4 | 9.4061 | 40.79 | A | G | 0.1153 |
| 2017 | 18-Jul-17 | FRESNO | ALMOND | Redacted4 | 9.4061 | 40.79 | A | G | 0.1153 |
| 2017 | 28-Jun-17 | FRESNO | ALMOND | Redacted4 | 4.2831 | 18.57 | A | G | 0.1153 |
| 2017 | 24-Jul-17 | FRESNO | ALMOND | Redacted4 | 3.0234 | 13.1 | A | G | 0.1154 |
| 2017 | 2-Aug-17 | FRESNO | ALMOND | Redacted4 | 2.9394 | 12.73 | A | G | 0.1155 |
| 2017 | 2-Aug-17 | FRESNO | ALMOND | Redacted4 | 7.3905 | 32 | A | G | 0.1155 |
| 2017 | 18-Jun-17 | FRESNO | ALMOND | Redacted4 | 7.3905 | 32 | A | G | 0.1155 |
| 2017 | 5-Aug-17 | FRESNO | ALMOND | Redacted4 | 4.6191 | 20 | A | G | 0.1155 |
| 2017 | 24-Jul-17 | FRESNO | ALMOND | Redacted4 | 3.9472 | 17 | A | G | 0.1161 |
| 2017 | 1-Aug-17 | MERCED | ALMOND | Redacted4 | 3.0234 | 13 | A | G | 0.1163 |
| 2017 | 4-Aug-17 | FRESNO | ALMOND | Redacted4 | 1.1758 | 5 | A | G | 0.1176 |
| 2017 | 25-Jul-17 | FRESNO | ALMOND | Redacted4 | 4.787 | 20.3 | A | G | 0.1179 |
| 2017 | 24-Jul-17 | FRESNO | ALMOND | Redacted4 | 6.3827 | 27 | A | G | 0.1182 |
| 2017 | 24-Jul-17 | FRESNO | ALMOND | Redacted4 | 15.3689 | 65 | A | G | 0.1182 |
| 2017 | 10-Jul-17 | FRESNO | ALMOND | Redacted4 | 4.1152 | 17 | A | G | 0.1210 |
| 2017 | 10-Jul-17 | FRESNO | ALMOND | Redacted4 | 3.1914 | 13.1 | A | G | 0.1218 |
| 2017 | 10-Jul-17 | FRESNO | ALMOND | Redacted4 | 4.955 | 20.3 | A | G | 0.1220 |
| 2017 | 10-Jul-17 | FRESNO | ALMOND | Redacted4 | 12.2615 | 50 | A | G | 0.1226 |
| 2017 | 10-Jul-17 | FRESNO | ALMOND | Redacted4 | 12.7654 | 52 | A | G | 0.1227 |
| 2017 | 12-Jul-17 | FRESNO | ALMOND | Redacted4 | 15.9568 | 65 | A | G | 0.1227 |
| 2017 | 10-Jul-17 | FRESNO | ALMOND | Redacted4 | 6.6347 | 27 | A | G | 0.1229 |
| 2017 | 28-Jul-17 | MADERA | ALMOND | Redacted4 | 24.8012 | 100 | A | G | 0.1240 |
| 2017 | 22-Jul-17 | FRESNO | ALMOND | Redacted4 | 18.6442 | 75 | A | G | 0.1243 |
| 2017 | 22-Jul-17 | FRESNO | ALMOND | Redacted4 | 31.1577 | 125 | A | G | 0.1246 |
| 2017 | 21-Jul-17 | KERN | ALMOND | Redacted4 | 8.0624 | 31 | A | G | 0.1300 |
| 2017 | 29-Jun-17 | FRESNO | ALMOND | Redacted4 | 15.4529 | 59 | A | G | 0.1310 |
| 2017 | 21-Jul-17 | KERN | ALMOND | Redacted4 | 11.0018 | 42 | A | G | 0.1310 |
| 2017 | 30-Jun-17 | FRESNO | ALMOND | Redacted4 | 17.5525 | 67 | A | G | 0.1310 |
| 2017 | 30-Jun-17 | FRESNO | ALMOND | Redacted4 | 19.652 | 75 | A | G | 0.1310 |
| 2017 | 29-Jun-17 | FRESNO | ALMOND | Redacted4 | 19.652 | 75 | A | G | 0.1310 |
| 2017 | 24-Jul-17 | MADERA | ALMOND | Redacted4 | 19.652 | 75 | A | G | 0.1310 |
| 2017 | 20-Jul-17 | MADERA | ALMOND | Redacted4 | 19.652 | 75 | A | G | 0.1310 |
| 2017 | 25-Jul-17 | MADERA | ALMOND | Redacted4 | 19.652 | 75 | A | G | 0.1310 |
| 2017 | 1-Jul-17 | FRESNO | ALMOND | Redacted4 | 13.1014 | 50 | A | G | 0.1310 |
| 2017 | 1-Jul-17 | FRESNO | ALMOND | Redacted4 | 13.1014 | 50 | A | G | 0.1310 |
| 2017 | 29-Jun-17 | FRESNO | ALMOND | Redacted4 | 23.5992 | 90 | A | G | 0.1311 |
| 2017 | 30-Jun-17 | FRESNO | ALMOND | Redacted4 | 38.5482 | 147 | A | G | 0.1311 |
| 2017 | 29-Jun-17 | FRESNO | ALMOND | Redacted4 | 40.6478 | 155 | A | G | 0.1311 |
| 2017 | 30-Jun-17 | FRESNO | ALMOND | Redacted4 | 19.1481 | 73 | A | G | 0.1312 |
| 2017 | 30-Jul-17 | KERN | ALMOND | Redacted4 | 48.5422 | 185 | A | G | 0.1312 |
| 2017 | 21-Jun-17 | FRESNO | ALMOND | Redacted4 | 5.2489 | 20 | A | G | 0.1312 |
| 2017 | 31-May-17 | FRESNO | ALMOND | Redacted4 | 5.7738 | 22 | A | G | 0.1312 |
| 2017 | 1-Jul-17 | FRESNO | ALMOND | Redacted4 | 41.9915 | 160 | A | G | 0.1312 |
| 2017 | 1-Jul-17 | FRESNO | ALMOND | Redacted4 | 8.3983 | 32 | A | G | 0.1312 |
| 2017 | 1-Jul-17 | FRESNO | ALMOND | Redacted4 | 41.9915 | 160 | A | G | 0.1312 |
| 2017 | 29-Jun-17 | FRESNO | ALMOND | Redacted4 | 16.7966 | 64 | A | G | 0.1312 |
| 2017 | 17-Jun-17 | FRESNO | ALMOND | Redacted4 | 41.9915 | 160 | A | A | 0.1312 |
| 2017 | 29-Jun-17 | FRESNO | ALMOND | Redacted4 | 16.7966 | 64 | A | G | 0.1312 |
| 2017 | 1-Jul-17 | FRESNO | ALMOND | Redacted4 | 8.3983 | 32 | A | G | 0.1312 |
| 2017 | 27-Jul-17 | KERN | ALMOND | Redacted4 | 67.1864 | 256 | A | G | 0.1312 |
| 2017 | 1-Jul-17 | FRESNO | ALMOND | Redacted4 | 10.4979 | 40 | A | G | 0.1312 |
| 2017 | 21-Jul-17 | KERN | ALMOND | Redacted4 | 10.4979 | 40 | A | G | 0.1312 |
| 2017 | 30-Jun-17 | FRESNO | ALMOND | Redacted4 | 39.2201 | 149.4 | A | G | 0.1313 |
| 2017 | 30-Jun-17 | FRESNO | ALMOND | Redacted4 | 22.8434 | 87 | A | G | 0.1313 |
| 2017 | 29-Jun-17 | FRESNO | ALMOND | Redacted4 | 39.3881 | 150 | A | G | 0.1313 |
| 2017 | 26-Jul-17 | MADERA | ALMOND | Redacted4 | 39.3881 | 150 | A | G | 0.1313 |
| 2017 | 12-Sep-17 | FRESNO | ALMOND | Redacted4 | 9.3221 | 35.5 | A | A | 0.1313 |
| 2017 | 1-Jul-17 | FRESNO | ALMOND | Redacted4 | 17.8044 | 67.8 | A | G | 0.1313 |
| 2017 | 30-Jun-17 | FRESNO | ALMOND | Redacted4 | 39.892 | 151.9 | A | G | 0.1313 |
| 2017 | 30-Jun-17 | FRESNO | ALMOND | Redacted4 | 36.7846 | 140 | A | G | 0.1314 |
| 2017 | 29-Jun-17 | FRESNO | ALMOND | Redacted4 | 30.4859 | 116 | A | G | 0.1314 |
| 2017 | 30-Jun-17 | FRESNO | ALMOND | Redacted4 | 20.2399 | 77 | A | G | 0.1314 |
| 2017 | 29-Jun-17 | FRESNO | ALMOND | Redacted4 | 26.2867 | 100 | A | G | 0.1314 |
| 2017 | 29-Jun-17 | FRESNO | ALMOND | Redacted4 | 22.0875 | 84 | A | G | 0.1315 |
| 2017 | 29-Jun-17 | FRESNO | ALMOND | Redacted4 | 17.8884 | 68 | A | G | 0.1315 |
| 2017 | 30-Jun-17 | FRESNO | ALMOND | Redacted4 | 15.7888 | 60 | A | G | 0.1316 |
| 2017 | 2-Jun-17 | FRESNO | ALMOND | Redacted4 | 5.2909 | 20 | A | G | 0.1323 |
| 2017 | 7-Sep-17 | FRESNO | ALMOND | Redacted4 | 25.027 | 93 | A | G | 0.1346 |
| 2017 | 19-Jul-17 | FRESNO | ALMOND | Redacted4 | 20.9958 | 76.3 | A | G | 0.1376 |
| 2017 | 15-Mar-17 | FRESNO | ALMOND | Redacted4 | 20.9958 | 76.3 | A | G | 0.1376 |
| 2017 | 15-Mar-17 | FRESNO | ALMOND | Redacted4 | 38.5482 | 140 | A | G | 0.1377 |
| 2017 | 19-Jul-17 | FRESNO | ALMOND | Redacted4 | 38.5482 | 140 | A | G | 0.1377 |
| 2017 | 19-Jul-17 | FRESNO | ALMOND | Redacted4 | 20.6598 | 75 | A | G | 0.1377 |
| 2017 | 15-Mar-17 | FRESNO | ALMOND | Redacted4 | 20.6598 | 75 | A | G | 0.1377 |
| 2017 | 21-Jul-17 | FRESNO | ALMOND | Redacted4 | 41.9075 | 152 | A | G | 0.1379 |
| 2017 | 11-Jul-17 | FRESNO | ALMOND | Redacted4 | 39.1361 | 135 | A | G | 0.1449 |
| 2017 | 23-Jul-17 | FRESNO | ALMOND | Redacted4 | 5.5429 | 17 | A | G | 0.1630 |
| 2017 | 21-Jul-17 | FRESNO | ALMOND | Redacted4 | 5.039 | 15.45 | A | G | 0.1631 |
| 2017 | 22-Aug-17 | MADERA | ALMOND | Redacted4 | 26.2027 | 80 | A | G | 0.1638 |
| 2017 | 23-Aug-17 | MADERA | ALMOND | Redacted4 | 49.1301 | 150 | A | G | 0.1638 |
| 2017 | 22-Jul-17 | MERCED | ALMOND | Redacted4 | 16.3767 | 50 | A | G | 0.1638 |
| 2017 | 24-Jul-17 | MADERA | ALMOND | Redacted4 | 6.5507 | 20 | A | G | 0.1638 |
| 2017 | 12-Aug-17 | FRESNO | ALMOND | Redacted4 | 41.3197 | 126 | A | A | 0.1640 |
| 2017 | 2-Jul-17 | FRESNO | ALMOND | Redacted4 | 32.8374 | 100 | A | A | 0.1642 |
| 2017 | 29-Aug-17 | FRESNO | ALMOND | Redacted4 | 32.8374 | 100 | A | A | 0.1642 |
| 2017 | 16-Jul-17 | FRESNO | ALMOND | Redacted4 | 11.8416 | 36 | A | G | 0.1645 |
| 2017 | 24-Jul-17 | MADERA | ALMOND | Redacted4 | 29.814 | 90 | A | G | 0.1656 |
| 2017 | 25-Jul-17 | MADERA | ALMOND | Redacted4 | 6.6347 | 20 | A | G | 0.1659 |
| 2017 | 25-Jul-17 | MADERA | ALMOND | Redacted4 | 26.8746 | 81 | A | G | 0.1659 |
| 2017 | 28-Aug-17 | MADERA | ALMOND | Redacted4 | 5.3749 | 15 | A | G | 0.1792 |
| 2017 | 23-Jul-17 | FRESNO | ALMOND | Redacted4 | 8.9862 | 25 | A | G | 0.1797 |
| 2017 | 23-Jul-17 | FRESNO | ALMOND | Redacted4 | 8.9862 | 25 | A | G | 0.1797 |
| 2017 | 30-Jul-17 | FRESNO | ALMOND | Redacted4 | 9.1542 | 25.45 | A | G | 0.1798 |
| 2017 | 2-Sep-17 | MADERA | ALMOND | Redacted4 | 24.1031 | 67 | A | G | 0.1799 |
| 2017 | 22-Jul-17 | FRESNO | ALMOND | Redacted4 | 13.8572 | 38.5 | A | G | 0.1800 |
| 2017 | 22-Jul-17 | FRESNO | ALMOND | Redacted4 | 15.117 | 42 | A | G | 0.1800 |
| 2017 | 27-Jul-17 | MADERA | ALMOND | Redacted4 | 12.5975 | 35 | A | G | 0.1800 |
| 2017 | 24-Jul-17 | MADERA | ALMOND | Redacted4 | 12.5975 | 35 | A | G | 0.1800 |
| 2017 | 28-Jul-17 | MERCED | ALMOND | Redacted4 | 7.5585 | 21 | A | G | 0.1800 |
| 2017 | 21-Jul-17 | FRESNO | ALMOND | Redacted4 | 16.2087 | 45 | A | G | 0.1801 |
| 2017 | 27-Jul-17 | FRESNO | ALMOND | Redacted4 | 27.3785 | 76 | A | G | 0.1801 |
| 2017 | 28-Jul-17 | MADERA | ALMOND | Redacted4 | 24.859 | 69 | A | G | 0.1801 |
| 2017 | 1-Jul-17 | FRESNO | ALMOND | Redacted4 | 13.1014 | 36.36 | A | G | 0.1802 |
| 2017 | 12-Jul-17 | FRESNO | ALMOND | Redacted4 | 13.1014 | 36.36 | A | G | 0.1802 |
| 2017 | 23-Jul-17 | FRESNO | ALMOND | Redacted4 | 27.3785 | 75.95 | A | G | 0.1802 |
| 2017 | 21-Jul-17 | FRESNO | ALMOND | Redacted4 | 23.4313 | 65 | A | G | 0.1802 |
| 2017 | 30-Aug-17 | MADERA | ALMOND | Redacted4 | 23.4313 | 65 | A | G | 0.1802 |
| 2017 | 21-Jul-17 | FRESNO | ALMOND | Redacted4 | 20.9118 | 58 | A | G | 0.1803 |
| 2017 | 21-Jul-17 | FRESNO | ALMOND | Redacted4 | 27.0425 | 75 | A | G | 0.1803 |
| 2017 | 21-Jul-17 | FRESNO | ALMOND | Redacted4 | 27.0425 | 75 | A | G | 0.1803 |
| 2017 | 21-Jul-17 | FRESNO | ALMOND | Redacted4 | 27.0425 | 75 | A | G | 0.1803 |
| 2017 | 21-Jul-17 | FRESNO | ALMOND | Redacted4 | 27.0425 | 75 | A | G | 0.1803 |
| 2017 | 22-Jul-17 | FRESNO | ALMOND | Redacted4 | 13.5213 | 37.5 | A | G | 0.1803 |
| 2017 | 22-Jul-17 | FRESNO | ALMOND | Redacted4 | 13.5213 | 37.5 | A | G | 0.1803 |
| 2017 | 21-Jul-17 | FRESNO | ALMOND | Redacted4 | 12.2615 | 34 | A | G | 0.1803 |
| 2017 | 24-Jul-17 | MADERA | ALMOND | Redacted4 | 24.5231 | 68 | A | G | 0.1803 |
| 2017 | 28-Aug-17 | MADERA | ALMOND | Redacted4 | 6.1308 | 17 | A | G | 0.1803 |
| 2017 | 21-Jul-17 | FRESNO | ALMOND | Redacted4 | 26.9586 | 74.75 | A | G | 0.1803 |
| 2017 | 29-Aug-17 | MADERA | ALMOND | Redacted4 | 15.8728 | 44 | A | G | 0.1804 |
| 2017 | 3-Sep-17 | MADERA | ALMOND | Redacted4 | 29.2261 | 81 | A | G | 0.1804 |
| 2017 | 21-Jul-17 | FRESNO | ALMOND | Redacted4 | 27.9664 | 77.5 | A | G | 0.1804 |
| 2017 | 23-Jul-17 | FRESNO | ALMOND | Redacted4 | 26.9586 | 74.7 | A | G | 0.1804 |
| 2017 | 28-Jul-17 | FRESNO | ALMOND | Redacted4 | 26.7066 | 74 | A | G | 0.1805 |
| 2017 | 26-Jul-17 | MADERA | ALMOND | Redacted4 | 13.3533 | 37 | A | G | 0.1805 |
| 2017 | 25-Jul-17 | MADERA | ALMOND | Redacted4 | 13.3533 | 37 | A | G | 0.1805 |
| 2017 | 1-Sep-17 | MADERA | ALMOND | Redacted4 | 24.1871 | 67 | A | G | 0.1805 |
| 2017 | 22-Jul-17 | FRESNO | ALMOND | Redacted4 | 12.0936 | 33.5 | A | G | 0.1805 |
| 2017 | 22-Jul-17 | FRESNO | ALMOND | Redacted4 | 15.7048 | 43.5 | A | G | 0.1805 |
| 2017 | 23-Jul-17 | FRESNO | ALMOND | Redacted4 | 26.5386 | 73.5 | A | G | 0.1805 |
| 2017 | 23-Jul-17 | FRESNO | ALMOND | Redacted4 | 7.2225 | 20 | A | G | 0.1806 |
| 2017 | 15-Jul-17 | FRESNO | ALMOND | Redacted4 | 7.2225 | 20 | A | A | 0.1806 |
| 2017 | 2-Sep-17 | MADERA | ALMOND | Redacted4 | 7.2225 | 20 | A | G | 0.1806 |
| 2017 | 29-Aug-17 | MADERA | ALMOND | Redacted4 | 7.2225 | 20 | A | G | 0.1806 |
| 2017 | 28-Jul-17 | FRESNO | ALMOND | Redacted4 | 10.8338 | 30 | A | G | 0.1806 |
| 2017 | 22-Jul-17 | FRESNO | ALMOND | Redacted4 | 10.8338 | 30 | A | G | 0.1806 |
| 2017 | 29-Aug-17 | MADERA | ALMOND | Redacted4 | 32.5014 | 90 | A | G | 0.1806 |
| 2017 | 23-Jul-17 | FRESNO | ALMOND | Redacted4 | 25.2789 | 70 | A | G | 0.1806 |
| 2017 | 23-Jul-17 | FRESNO | ALMOND | Redacted4 | 28.8902 | 80 | A | G | 0.1806 |
| 2017 | 23-Jul-17 | FRESNO | ALMOND | Redacted4 | 28.8902 | 80 | A | G | 0.1806 |
| 2017 | 24-Jul-17 | MERCED | ALMOND | Redacted4 | 14.4451 | 40 | A | G | 0.1806 |
| 2017 | 21-Jul-17 | FRESNO | ALMOND | Redacted4 | 18.0564 | 50 | A | G | 0.1806 |
| 2017 | 22-Jul-17 | FRESNO | ALMOND | Redacted4 | 13.1853 | 36.5 | A | G | 0.1806 |
| 2017 | 21-Jul-17 | FRESNO | ALMOND | Redacted4 | 27.4625 | 76 | A | G | 0.1807 |
| 2017 | 22-Jul-17 | MADERA | ALMOND | Redacted4 | 20.2399 | 56 | A | G | 0.1807 |
| 2017 | 22-Jul-17 | FRESNO | ALMOND | Redacted4 | 10.6658 | 29.5 | A | G | 0.1808 |
| 2017 | 23-Jul-17 | FRESNO | ALMOND | Redacted4 | 12.2615 | 33.9 | A | G | 0.1808 |
| 2017 | 25-Jul-17 | FRESNO | ALMOND | Redacted4 | 11.8416 | 32.73 | A | G | 0.1809 |
| 2017 | 23-Jul-17 | FRESNO | ALMOND | Redacted4 | 5.7948 | 16 | A | G | 0.1811 |
| 2017 | 23-Jul-17 | FRESNO | ALMOND | Redacted4 | 5.7948 | 16 | A | G | 0.1811 |
| 2017 | 22-Jul-17 | FRESNO | ALMOND | Redacted4 | 11.5897 | 32 | A | G | 0.1811 |
| 2017 | 22-Jul-17 | FRESNO | ALMOND | Redacted4 | 11.5897 | 32 | A | G | 0.1811 |
| 2017 | 27-Jun-17 | FRESNO | ALMOND | Redacted4 | 18.6442 | 51.14 | A | G | 0.1823 |
| 2017 | 27-Jul-17 | FRESNO | ALMOND | Redacted4 | 92.1189 | 150 | A | G | 0.3071 |
| 2017 | 14-May-17 | MADERA | APPLE | Redacted4 | 3.9472 | 30 | A | G | 0.0658 |
| 2017 | 26-Aug-17 | MONTEREY | ARTICHOKE (GLOBE) (ALL OR UNSPEC) | Redacted4 | 2.8554 | 5.5 | A | G | 0.2596 |
| 2017 | 11-Sep-17 | MONTEREY | ARTICHOKE (GLOBE) (ALL OR UNSPEC) | Redacted4 | 2.8554 | 5.5 | A | G | 0.2596 |
| 2017 | 8-Dec-17 | MONTEREY | ARTICHOKE (GLOBE) (ALL OR UNSPEC) | Redacted4 | 2.8554 | 5.5 | A | G | 0.2596 |
| 2017 | 26-Aug-17 | MONTEREY | ARTICHOKE (GLOBE) (ALL OR UNSPEC) | Redacted4 | 2.6035 | 5 | A | G | 0.2604 |
| 2017 | 10-Aug-17 | MONTEREY | ARTICHOKE (GLOBE) (ALL OR UNSPEC) | Redacted4 | 2.6035 | 5 | A | G | 0.2604 |
| 2017 | 11-Sep-17 | MONTEREY | ARTICHOKE (GLOBE) (ALL OR UNSPEC) | Redacted4 | 2.6035 | 5 | A | G | 0.2604 |
| 2017 | 28-Oct-17 | MONTEREY | ARTICHOKE (GLOBE) (ALL OR UNSPEC) | Redacted4 | 13.1014 | 25 | A | G | 0.2620 |
| 2017 | 8-Dec-17 | MONTEREY | ARTICHOKE (GLOBE) (ALL OR UNSPEC) | Redacted4 | 6.4667 | 12.3 | A | G | 0.2629 |
| 2017 | 28-Oct-17 | MONTEREY | ARTICHOKE (GLOBE) (ALL OR UNSPEC) | Redacted4 | 11.5897 | 22 | A | G | 0.2634 |
| 2017 | 16-Oct-17 | MONTEREY | ARTICHOKE (GLOBE) (ALL OR UNSPEC) | Redacted4 | 3.1914 | 6 | A | G | 0.2660 |
| 2017 | 8-Dec-17 | MONTEREY | ARTICHOKE (GLOBE) (ALL OR UNSPEC) | Redacted4 | 1.3437 | 2.5 | A | G | 0.2687 |
| 2017 | 7-Aug-17 | SAN DIEGO | AVOCADO (ALL OR UNSPEC) | Redacted4 | 9.1542 | 40 | A | A | 0.1144 |
| 2017 | 18-Aug-17 | SAN DIEGO | AVOCADO (ALL OR UNSPEC) | Redacted4 | 1.8371 | 8 | A | A | 0.1148 |
| 2017 | 18-Aug-17 | SAN DIEGO | AVOCADO (ALL OR UNSPEC) | Redacted4 | 1.1482 | 5 | A | A | 0.1148 |
| 2017 | 18-Aug-17 | SAN DIEGO | AVOCADO (ALL OR UNSPEC) | Redacted4 | 0.9186 | 4 | A | A | 0.1148 |
| 2017 | 1-Jun-17 | SAN DIEGO | AVOCADO (ALL OR UNSPEC) | Redacted4 | 2.0996 | 9 | A | A | 0.1166 |
| 2017 | 5-Sep-17 | SAN DIEGO | AVOCADO (ALL OR UNSPEC) | Redacted4 | 1.1758 | 5 | A | A | 0.1176 |
| 2017 | 5-Jun-17 | SAN DIEGO | AVOCADO (ALL OR UNSPEC) | Redacted4 | 20.9958 | 80 | A | A | 0.1312 |
| 2017 | 23-Jun-17 | SAN DIEGO | AVOCADO (ALL OR UNSPEC) | Redacted4 | 2.0996 | 8 | A | A | 0.1312 |
| 2017 | 21-Jul-17 | SANTA CLARA | BEANS (ALL OR UNSPEC) | Redacted4 | 8.6503 | 44 | A | G | 0.0983 |
| 2017 | 8-Aug-17 | SANTA CLARA | BEANS (ALL OR UNSPEC) | Redacted4 | 8.6503 | 44 | A | G | 0.0983 |
| 2017 | 21-Jul-17 | SANTA CLARA | BEANS (ALL OR UNSPEC) | Redacted4 | 3.1914 | 16 | A | G | 0.0997 |
| 2017 | 10-Aug-17 | SANTA CLARA | BEANS (ALL OR UNSPEC) | Redacted4 | 3.1914 | 16 | A | G | 0.0997 |
| 2017 | 12-Jul-17 | SANTA CLARA | BEANS, SUCCULENT (OTHER THAN LIMA) | Redacted4 | 1.9316 | 7.5 | A | G | 0.1288 |
| 2017 | 25-Oct-17 | SANTA CLARA | BEANS, SUCCULENT (OTHER THAN LIMA) | Redacted4 | 1.9316 | 7.5 | A | G | 0.1288 |
| 2017 | 17-Aug-17 | SANTA CLARA | BEANS, SUCCULENT (OTHER THAN LIMA) | Redacted4 | 1.9316 | 7.5 | A | G | 0.1288 |
| 2017 | 29-Jul-17 | SANTA CLARA | BEANS, SUCCULENT (OTHER THAN LIMA) | Redacted4 | 1.9316 | 7.5 | A | G | 0.1288 |
| 2017 | 26-Jul-17 | VENTURA | BEANS, SUCCULENT (OTHER THAN LIMA) | Redacted4 | 4.4511 | 17 | A | A | 0.1309 |
| 2017 | 11-Jul-17 | SANTA CLARA | BEANS, SUCCULENT (OTHER THAN LIMA) | Redacted4 | 1.5957 | 6 | A | G | 0.1330 |
| 2017 | 16-Sep-17 | SANTA CLARA | BEANS, SUCCULENT (OTHER THAN LIMA) | Redacted4 | 0.7558 | 2.8 | A | G | 0.1350 |
| 2017 | 30-Jun-17 | VENTURA | BLACKBERRY | Redacted4 | 0.0381 | 0.58 | A | G | 0.0328 |
| 2017 | 15-May-17 | VENTURA | BLACKBERRY | Redacted4 | 0.1115 | 0.49 | A | G | 0.1138 |
| 2017 | 8-May-17 | VENTURA | BLACKBERRY | Redacted4 | 0.1115 | 0.49 | A | G | 0.1138 |
| 2017 | 18-Aug-17 | VENTURA | BLACKBERRY | Redacted4 | 0.4462 | 1.95 | A | G | 0.1144 |
| 2017 | 26-Jul-17 | VENTURA | BLACKBERRY | Redacted4 | 0.4462 | 1.95 | A | G | 0.1144 |
| 2017 | 14-Jun-17 | VENTURA | BLACKBERRY | Redacted4 | 0.5446 | 2.37 | A | G | 0.1149 |
| 2017 | 21-Dec-17 | VENTURA | BLACKBERRY | Redacted4 | 2.5589 | 11.13 | A | G | 0.1150 |
| 2017 | 26-Apr-17 | VENTURA | BLACKBERRY | Redacted4 | 2.5589 | 11.13 | A | G | 0.1150 |
| 2017 | 12-Apr-17 | VENTURA | BLACKBERRY | Redacted4 | 0.5774 | 2.5 | A | G | 0.1155 |
| 2017 | 17-Mar-17 | SANTA CRUZ | BLACKBERRY | Redacted4 | 0.8398 | 2.6 | A | G | 0.1615 |
| 2017 | 5-Apr-17 | SANTA CRUZ | BLACKBERRY | Redacted4 | 1.3437 | 2.61 | A | G | 0.2574 |
| 2017 | 5-Apr-17 | SANTA CRUZ | BLACKBERRY | Redacted4 | 1.3437 | 2.6 | A | G | 0.2584 |
| 2017 | 30-Jun-17 | MONTEREY | BLACKBERRY | Redacted4 | 16.7966 | 27 | A | G | 0.3110 |
| 2017 | 12-Jul-17 | SANTA CRUZ | BLACKBERRY | Redacted4 | 4.3671 | 7 | A | G | 0.3119 |
| 2017 | 31-Jul-17 | MONTEREY | BLACKBERRY | Redacted4 | 17.4685 | 28 | A | G | 0.3119 |
| 2017 | 11-Jul-17 | SANTA CRUZ | BLACKBERRY | Redacted4 | 9.994 | 16 | A | G | 0.3123 |
| 2017 | 19-Sep-17 | MONTEREY | BLACKBERRY | Redacted4 | 11.2537 | 18 | A | G | 0.3126 |
| 2017 | 29-Apr-17 | VENTURA | BLUEBERRY | Redacted4 | 0.6561 | 5 | A | G | 0.0656 |
| 2017 | 13-May-17 | VENTURA | BLUEBERRY | Redacted4 | 1.9093 | 8.3 | A | G | 0.1150 |
| 2017 | 18-Apr-17 | VENTURA | BLUEBERRY | Redacted4 | 1.9093 | 8.3 | A | G | 0.1150 |
| 2017 | 24-May-17 | VENTURA | BLUEBERRY | Redacted4 | 1.9093 | 8.3 | A | G | 0.1150 |
| 2017 | 6-Jun-17 | VENTURA | BLUEBERRY | Redacted4 | 1.9093 | 8.3 | A | G | 0.1150 |
| 2017 | 27-Apr-17 | VENTURA | BLUEBERRY | Redacted4 | 1.9093 | 8.3 | A | G | 0.1150 |
| 2017 | 11-Apr-17 | VENTURA | BLUEBERRY | Redacted4 | 1.9093 | 8.3 | A | G | 0.1150 |
| 2017 | 16-Jun-17 | SAN LUIS OBISPO | BOK CHOY (WONG BOK) | Redacted4 | 0.5039 | 2.6 | A | G | 0.0969 |
| 2017 | 12-Jul-17 | SANTA BARBARA | BOK CHOY (WONG BOK) | Redacted4 | 0.168 | 0.6 | A | G | 0.1400 |
| 2017 | 20-Jun-17 | SANTA CLARA | BROCCOLI | Redacted4 | 2.6035 | 20 | A | G | 0.0651 |
| 2017 | 21-Jun-17 | SANTA CLARA | BROCCOLI | Redacted4 | 3.9472 | 30 | A | G | 0.0658 |
| 2017 | 12-Jul-17 | SANTA CLARA | BROCCOLI | Redacted4 | 6.8866 | 30 | A | G | 0.1148 |
| 2017 | 14-Jun-17 | SANTA BARBARA | BROCCOLI | Redacted4 | 1.3437 | 5.2 | A | G | 0.1292 |
| 2017 | 22-Jun-17 | SAN LUIS OBISPO | BROCCOLI | Redacted4 | 1.7059 | 6.5 | A | G | 0.1312 |
| 2017 | 15-Jun-17 | SAN LUIS OBISPO | BROCCOLI | Redacted4 | 1.6272 | 6.2 | A | G | 0.1312 |
| 2017 | 17-May-17 | SANTA BARBARA | BROCCOLI | Redacted4 | 1.4435 | 5.5 | A | G | 0.1312 |
| 2017 | 17-May-17 | SANTA BARBARA | BROCCOLI | Redacted4 | 1.4435 | 5.5 | A | G | 0.1312 |
| 2017 | 18-May-17 | SANTA CLARA | BROCCOLI | Redacted4 | 7.8944 | 30 | A | G | 0.1316 |
| 2017 | 19-May-17 | SANTA CLARA | BROCCOLI | Redacted4 | 5.2909 | 20 | A | G | 0.1323 |
| 2017 | 2-Jun-17 | SANTA BARBARA | BROCCOLI | Redacted4 | 0.3359 | 1.1 | A | G | 0.1527 |
| 2017 | 6-Jul-17 | SANTA BARBARA | BROCCOLI | Redacted4 | 1.7636 | 5.5 | A | G | 0.1603 |
| 2017 | 2-Jun-17 | SANTA BARBARA | BROCCOLI | Redacted4 | 1.7636 | 5.5 | A | G | 0.1603 |
| 2017 | 2-Jun-17 | SANTA BARBARA | BROCCOLI | Redacted4 | 1.7636 | 5.5 | A | G | 0.1603 |
| 2017 | 30-May-17 | SAN LUIS OBISPO | BROCCOLI | Redacted4 | 2.1836 | 6.7 | A | G | 0.1630 |
| 2017 | 21-Jul-17 | SANTA BARBARA | BROCCOLI | Redacted4 | 5.5429 | 17 | A | G | 0.1630 |
| 2017 | 5-Jun-17 | SANTA BARBARA | BROCCOLI | Redacted4 | 8.8182 | 27 | A | G | 0.1633 |
| 2017 | 5-Jun-17 | SANTA BARBARA | BROCCOLI | Redacted4 | 5.8788 | 18 | A | G | 0.1633 |
| 2017 | 2-Aug-17 | SAN LUIS OBISPO | BROCCOLI | Redacted4 | 1.1758 | 3.6 | A | G | 0.1633 |
| 2017 | 20-Nov-17 | SANTA BARBARA | BROCCOLI | Redacted4 | 1.8043 | 5.5 | A | G | 0.1640 |
| 2017 | 7-Jul-17 | SANTA BARBARA | BROCCOLI | Redacted4 | 5.2489 | 16 | A | G | 0.1640 |
| 2017 | 24-Jun-17 | SANTA BARBARA | BROCCOLI | Redacted4 | 2.2964 | 7 | A | G | 0.1640 |
| 2017 | 22-Jun-17 | SANTA BARBARA | BROCCOLI | Redacted4 | 4.5928 | 14 | A | G | 0.1640 |
| 2017 | 7-Jul-17 | SANTA BARBARA | BROCCOLI | Redacted4 | 10.8259 | 33 | A | G | 0.1640 |
| 2017 | 23-Jun-17 | SANTA BARBARA | BROCCOLI | Redacted4 | 7.7094 | 23.5 | A | G | 0.1640 |
| 2017 | 7-Jul-17 | SANTA BARBARA | BROCCOLI | Redacted4 | 13.1224 | 40 | A | G | 0.1640 |
| 2017 | 20-Jul-17 | SANTA BARBARA | BROCCOLI | Redacted4 | 1.6403 | 5 | A | G | 0.1640 |
| 2017 | 20-Jul-17 | SANTA BARBARA | BROCCOLI | Redacted4 | 1.6403 | 5 | A | G | 0.1640 |
| 2017 | 9-Jun-17 | SAN LUIS OBISPO | BROCCOLI | Redacted4 | 2.1324 | 6.5 | A | G | 0.1640 |
| 2017 | 24-Jun-17 | SANTA BARBARA | BROCCOLI | Redacted4 | 4.2648 | 13 | A | G | 0.1640 |
| 2017 | 23-May-17 | SAN LUIS OBISPO | BROCCOLI | Redacted4 | 2.034 | 6.2 | A | G | 0.1640 |
| 2017 | 13-Jul-17 | SANTA BARBARA | BROCCOLI | Redacted4 | 11.8416 | 36 | A | G | 0.1645 |
| 2017 | 6-Jul-17 | SANTA BARBARA | BROCCOLI | Redacted4 | 1.6797 | 5 | A | G | 0.1680 |
| 2017 | 8-Dec-17 | MONTEREY | BROCCOLI | Redacted4 | 13.0174 | 28.4 | A | G | 0.2292 |
| 2017 | 6-Dec-17 | MONTEREY | BROCCOLI | Redacted4 | 6.2147 | 13.5 | A | G | 0.2302 |
| 2017 | 1-Dec-17 | MONTEREY | BROCCOLI | Redacted4 | 9.4901 | 20.6 | A | G | 0.2303 |
| 2017 | 12-Jun-17 | SAN LUIS OBISPO | BRUSSELS SPROUTS | Redacted4 | 1.5957 | 6.2 | A | G | 0.1287 |
| 2017 | 17-Jul-17 | VENTURA | BRUSSELS SPROUTS | Redacted4 | 4.7031 | 18 | A | G | 0.1306 |
| 2017 | 16-Aug-17 | SAN LUIS OBISPO | BRUSSELS SPROUTS | Redacted4 | 3.6113 | 13.8 | A | G | 0.1308 |
| 2017 | 22-Jun-17 | SAN LUIS OBISPO | BRUSSELS SPROUTS | Redacted4 | 0.1312 | 0.5 | A | G | 0.1312 |
| 2017 | 31-Jul-17 | SAN LUIS OBISPO | BRUSSELS SPROUTS | Redacted4 | 3.3593 | 12.8 | A | G | 0.1312 |
| 2017 | 31-Jul-17 | SAN LUIS OBISPO | BRUSSELS SPROUTS | Redacted4 | 2.8869 | 11 | A | G | 0.1312 |
| 2017 | 30-Jun-17 | SAN LUIS OBISPO | BRUSSELS SPROUTS | Redacted4 | 3.648 | 13.9 | A | G | 0.1312 |
| 2017 | 20-Dec-17 | SAN DIEGO | BRUSSELS SPROUTS | Redacted4 | 14.1721 | 54 | A | G | 0.1312 |
| 2017 | 14-Dec-17 | SAN DIEGO | BRUSSELS SPROUTS | Redacted4 | 26.2447 | 100 | A | G | 0.1312 |
| 2017 | 23-Dec-17 | SAN DIEGO | BRUSSELS SPROUTS | Redacted4 | 11.0228 | 42 | A | G | 0.1312 |
| 2017 | 13-Jun-17 | SAN LUIS OBISPO | BRUSSELS SPROUTS | Redacted4 | 2.635 | 10.04 | A | G | 0.1312 |
| 2017 | 1-Jun-17 | SAN LUIS OBISPO | BRUSSELS SPROUTS | Redacted4 | 1.391 | 5.3 | A | G | 0.1312 |
| 2017 | 1-Jun-17 | SAN LUIS OBISPO | BRUSSELS SPROUTS | Redacted4 | 1.2073 | 4.6 | A | G | 0.1312 |
| 2017 | 30-Jun-17 | SAN LUIS OBISPO | BRUSSELS SPROUTS | Redacted4 | 0.3412 | 1.3 | A | G | 0.1312 |
| 2017 | 16-Aug-17 | SAN LUIS OBISPO | BRUSSELS SPROUTS | Redacted4 | 3.0234 | 11.5 | A | G | 0.1315 |
| 2017 | 30-May-17 | SAN LUIS OBISPO | BRUSSELS SPROUTS | Redacted4 | 0.9238 | 3.5 | A | G | 0.1320 |
| 2017 | 12-Jun-17 | SAN LUIS OBISPO | BRUSSELS SPROUTS | Redacted4 | 2.5195 | 9.5 | A | G | 0.1326 |
| 2017 | 30-May-17 | SAN LUIS OBISPO | BRUSSELS SPROUTS | Redacted4 | 2.6875 | 10.1 | A | G | 0.1330 |
| 2017 | 21-Jun-17 | SAN LUIS OBISPO | BRUSSELS SPROUTS | Redacted4 | 1.5957 | 5 | A | G | 0.1596 |
| 2017 | 17-May-17 | SAN LUIS OBISPO | BRUSSELS SPROUTS | Redacted4 | 1.9316 | 6 | A | G | 0.1610 |
| 2017 | 19-May-17 | SAN LUIS OBISPO | BRUSSELS SPROUTS | Redacted4 | 2.5195 | 7.8 | A | G | 0.1615 |
| 2017 | 17-Jul-17 | SAN LUIS OBISPO | BRUSSELS SPROUTS | Redacted4 | 3.2753 | 10.1 | A | G | 0.1621 |
| 2017 | 17-May-17 | SAN LUIS OBISPO | BRUSSELS SPROUTS | Redacted4 | 2.0156 | 6.2 | A | G | 0.1625 |
| 2017 | 10-Aug-17 | VENTURA | BRUSSELS SPROUTS | Redacted4 | 2.8554 | 8.75 | A | G | 0.1632 |
| 2017 | 28-Sep-17 | VENTURA | BRUSSELS SPROUTS | Redacted4 | 5.8788 | 18 | A | G | 0.1633 |
| 2017 | 5-Oct-17 | VENTURA | BRUSSELS SPROUTS | Redacted4 | 5.8788 | 18 | A | G | 0.1633 |
| 2017 | 25-May-17 | SAN LUIS OBISPO | BRUSSELS SPROUTS | Redacted4 | 2.5195 | 7.7 | A | G | 0.1636 |
| 2017 | 4-Aug-17 | SAN LUIS OBISPO | BRUSSELS SPROUTS | Redacted4 | 2.6875 | 8.2 | A | G | 0.1639 |
| 2017 | 8-Jul-17 | SAN LUIS OBISPO | BRUSSELS SPROUTS | Redacted4 | 2.4932 | 7.6 | A | G | 0.1640 |
| 2017 | 14-Jun-17 | SAN LUIS OBISPO | BRUSSELS SPROUTS | Redacted4 | 2.4932 | 7.6 | A | G | 0.1640 |
| 2017 | 22-May-17 | SAN LUIS OBISPO | BRUSSELS SPROUTS | Redacted4 | 3.0017 | 9.15 | A | G | 0.1640 |
| 2017 | 8-Jun-17 | SAN LUIS OBISPO | BRUSSELS SPROUTS | Redacted4 | 2.9853 | 9.1 | A | G | 0.1640 |
| 2017 | 4-Aug-17 | SAN LUIS OBISPO | BRUSSELS SPROUTS | Redacted4 | 2.9853 | 9.1 | A | G | 0.1640 |
| 2017 | 7-Jun-17 | SAN LUIS OBISPO | BRUSSELS SPROUTS | Redacted4 | 2.9197 | 8.9 | A | G | 0.1640 |
| 2017 | 11-Aug-17 | SAN LUIS OBISPO | BRUSSELS SPROUTS | Redacted4 | 4.6584 | 14.2 | A | G | 0.1640 |
| 2017 | 29-Jul-17 | SAN LUIS OBISPO | BRUSSELS SPROUTS | Redacted4 | 3.4446 | 10.5 | A | G | 0.1640 |
| 2017 | 23-May-17 | SAN LUIS OBISPO | BRUSSELS SPROUTS | Redacted4 | 3.4446 | 10.5 | A | G | 0.1640 |
| 2017 | 13-Jul-17 | SAN LUIS OBISPO | BRUSSELS SPROUTS | Redacted4 | 1.1482 | 3.5 | A | G | 0.1640 |
| 2017 | 11-Aug-17 | SAN LUIS OBISPO | BRUSSELS SPROUTS | Redacted4 | 4.0023 | 12.2 | A | G | 0.1640 |
| 2017 | 19-Jul-17 | SAN LUIS OBISPO | BRUSSELS SPROUTS | Redacted4 | 4.56 | 13.9 | A | G | 0.1640 |
| 2017 | 3-Aug-17 | SAN LUIS OBISPO | BRUSSELS SPROUTS | Redacted4 | 4.56 | 13.9 | A | G | 0.1640 |
| 2017 | 11-Aug-17 | SAN LUIS OBISPO | BRUSSELS SPROUTS | Redacted4 | 3.4118 | 10.4 | A | G | 0.1640 |
| 2017 | 14-Jul-17 | SAN LUIS OBISPO | BRUSSELS SPROUTS | Redacted4 | 3.2937 | 10.04 | A | G | 0.1640 |
| 2017 | 25-May-17 | SAN LUIS OBISPO | BRUSSELS SPROUTS | Redacted4 | 2.7885 | 8.5 | A | G | 0.1640 |
| 2017 | 20-Jun-17 | SAN LUIS OBISPO | BRUSSELS SPROUTS | Redacted4 | 1.6403 | 5 | A | G | 0.1640 |
| 2017 | 23-May-17 | SAN LUIS OBISPO | BRUSSELS SPROUTS | Redacted4 | 2.385 | 7.27 | A | G | 0.1640 |
| 2017 | 11-Aug-17 | SAN LUIS OBISPO | BRUSSELS SPROUTS | Redacted4 | 4.7372 | 14.44 | A | G | 0.1640 |
| 2017 | 13-Jul-17 | SAN LUIS OBISPO | BRUSSELS SPROUTS | Redacted4 | 4.7372 | 14.44 | A | G | 0.1640 |
| 2017 | 20-Jun-17 | SAN LUIS OBISPO | BRUSSELS SPROUTS | Redacted4 | 4.7372 | 14.44 | A | G | 0.1640 |
| 2017 | 19-Jul-17 | SAN LUIS OBISPO | BRUSSELS SPROUTS | Redacted4 | 2.6901 | 8.2 | A | G | 0.1640 |
| 2017 | 13-Jul-17 | SAN LUIS OBISPO | BRUSSELS SPROUTS | Redacted4 | 5.9051 | 18 | A | G | 0.1640 |
| 2017 | 11-Aug-17 | SAN LUIS OBISPO | BRUSSELS SPROUTS | Redacted4 | 5.9051 | 18 | A | G | 0.1640 |
| 2017 | 11-Aug-17 | SAN LUIS OBISPO | BRUSSELS SPROUTS | Redacted4 | 3.1822 | 9.7 | A | G | 0.1640 |
| 2017 | 22-Jun-17 | SAN LUIS OBISPO | BRUSSELS SPROUTS | Redacted4 | 4.7569 | 14.5 | A | G | 0.1640 |
| 2017 | 3-Aug-17 | SAN LUIS OBISPO | BRUSSELS SPROUTS | Redacted4 | 3.1494 | 9.6 | A | G | 0.1640 |
| 2017 | 14-Aug-17 | SAN LUIS OBISPO | BRUSSELS SPROUTS | Redacted4 | 3.1166 | 9.5 | A | G | 0.1640 |
| 2017 | 3-Aug-17 | SAN LUIS OBISPO | BRUSSELS SPROUTS | Redacted4 | 3.1166 | 9.5 | A | G | 0.1640 |
| 2017 | 19-Jul-17 | SAN LUIS OBISPO | BRUSSELS SPROUTS | Redacted4 | 2.0668 | 6.3 | A | G | 0.1640 |
| 2017 | 26-May-17 | SAN LUIS OBISPO | BRUSSELS SPROUTS | Redacted4 | 0.9842 | 3 | A | G | 0.1640 |
| 2017 | 25-May-17 | SAN LUIS OBISPO | BRUSSELS SPROUTS | Redacted4 | 1.9684 | 6 | A | G | 0.1640 |
| 2017 | 13-Jul-17 | SAN LUIS OBISPO | BRUSSELS SPROUTS | Redacted4 | 0.9842 | 3 | A | G | 0.1640 |
| 2017 | 22-May-17 | SAN LUIS OBISPO | BRUSSELS SPROUTS | Redacted4 | 2.8554 | 8.7 | A | G | 0.1641 |
| 2017 | 19-May-17 | SAN LUIS OBISPO | BRUSSELS SPROUTS | Redacted4 | 0.7558 | 2.3 | A | G | 0.1643 |
| 2017 | 18-Jul-17 | SAN LUIS OBISPO | BRUSSELS SPROUTS | Redacted4 | 1.5117 | 4.6 | A | G | 0.1643 |
| 2017 | 25-May-17 | SAN LUIS OBISPO | BRUSSELS SPROUTS | Redacted4 | 3.3593 | 10.2 | A | G | 0.1647 |
| 2017 | 18-Jul-17 | SAN LUIS OBISPO | BRUSSELS SPROUTS | Redacted4 | 3.6953 | 11.2 | A | G | 0.1650 |
| 2017 | 18-May-17 | SAN LUIS OBISPO | BRUSSELS SPROUTS | Redacted4 | 3.4433 | 10.4 | A | G | 0.1655 |
| 2017 | 19-May-17 | SAN LUIS OBISPO | BRUSSELS SPROUTS | Redacted4 | 2.5195 | 7.6 | A | G | 0.1658 |
| 2017 | 8-Aug-17 | SAN LUIS OBISPO | BRUSSELS SPROUTS | Redacted4 | 3.0234 | 9.1 | A | G | 0.1661 |
| 2017 | 4-Aug-17 | SAN LUIS OBISPO | BRUSSELS SPROUTS | Redacted4 | 2.0996 | 6.3 | A | G | 0.1666 |
| 2017 | 28-Oct-17 | VENTURA | BRUSSELS SPROUTS | Redacted4 | 7.0546 | 18 | A | G | 0.1960 |
| 2017 | 25-Nov-17 | VENTURA | BRUSSELS SPROUTS | Redacted4 | 7.0546 | 18 | A | G | 0.1960 |
| 2017 | 2-Dec-17 | VENTURA | BRUSSELS SPROUTS | Redacted4 | 7.0546 | 18 | A | G | 0.1960 |
| 2017 | 17-Jul-17 | SAN LUIS OBISPO | BRUSSELS SPROUTS | Redacted4 | 2.4355 | 6.2 | A | G | 0.1964 |
| 2017 | 27-Jun-17 | SAN LUIS OBISPO | BRUSSELS SPROUTS | Redacted4 | 1.7715 | 4.5 | A | G | 0.1968 |
| 2017 | 27-Jun-17 | SAN LUIS OBISPO | BRUSSELS SPROUTS | Redacted4 | 3.5824 | 9.1 | A | G | 0.1968 |
| 2017 | 27-Jun-17 | SAN LUIS OBISPO | BRUSSELS SPROUTS | Redacted4 | 3.7399 | 9.5 | A | G | 0.1968 |
| 2017 | 27-Jun-17 | SAN LUIS OBISPO | BRUSSELS SPROUTS | Redacted4 | 3.5037 | 8.9 | A | G | 0.1968 |
| 2017 | 14-Jul-17 | SAN LUIS OBISPO | BRUSSELS SPROUTS | Redacted4 | 3.6113 | 9.1 | A | G | 0.1984 |
| 2017 | 17-Jul-17 | SAN LUIS OBISPO | BRUSSELS SPROUTS | Redacted4 | 3.7792 | 9.5 | A | G | 0.1989 |
| 2017 | 4-Nov-17 | VENTURA | BRUSSELS SPROUTS | Redacted4 | 8.2303 | 18 | A | G | 0.2286 |
| 2017 | 7-Jun-17 | SANTA CRUZ | BRUSSELS SPROUTS | Redacted4 | 0.7558 | 1.5 | A | G | 0.2519 |
| 2017 | 23-Jul-17 | MONTEREY | BRUSSELS SPROUTS | Redacted4 | 1.5117 | 3 | A | G | 0.2520 |
| 2017 | 14-Jul-17 | SANTA CRUZ | BRUSSELS SPROUTS | Redacted4 | 0.5039 | 1 | A | G | 0.2520 |
| 2017 | 14-Aug-17 | SANTA CRUZ | BRUSSELS SPROUTS | Redacted4 | 0.5039 | 1 | A | G | 0.2520 |
| 2017 | 7-Jun-17 | SANTA CRUZ | BRUSSELS SPROUTS | Redacted4 | 0.5039 | 1 | A | G | 0.2520 |
| 2017 | 22-Aug-17 | MONTEREY | BRUSSELS SPROUTS | Redacted4 | 1.1758 | 2.3 | A | G | 0.2556 |
| 2017 | 7-Sep-17 | MONTEREY | BRUSSELS SPROUTS | Redacted4 | 1.1758 | 2.3 | A | G | 0.2556 |
| 2017 | 7-Jun-17 | MONTEREY | BRUSSELS SPROUTS | Redacted4 | 1.1758 | 2.3 | A | G | 0.2556 |
| 2017 | 1-Jul-17 | MONTEREY | BRUSSELS SPROUTS | Redacted4 | 1.1758 | 2.3 | A | G | 0.2556 |
| 2017 | 18-Jul-17 | MONTEREY | BRUSSELS SPROUTS | Redacted4 | 1.1758 | 2.3 | A | G | 0.2556 |
| 2017 | 20-Sep-17 | MONTEREY | BRUSSELS SPROUTS | Redacted4 | 3.6113 | 7 | A | G | 0.2580 |
| 2017 | 5-Oct-17 | MONTEREY | BRUSSELS SPROUTS | Redacted4 | 5.7108 | 11 | A | G | 0.2596 |
| 2017 | 6-Sep-17 | MONTEREY | BRUSSELS SPROUTS | Redacted4 | 3.2753 | 6.3 | A | G | 0.2599 |
| 2017 | 6-Jun-17 | MONTEREY | BRUSSELS SPROUTS | Redacted4 | 3.2753 | 6.3 | A | G | 0.2599 |
| 2017 | 4-Jul-17 | MONTEREY | BRUSSELS SPROUTS | Redacted4 | 3.2753 | 6.3 | A | G | 0.2599 |
| 2017 | 22-Jul-17 | MONTEREY | BRUSSELS SPROUTS | Redacted4 | 3.2753 | 6.3 | A | G | 0.2599 |
| 2017 | 7-Nov-17 | MONTEREY | BRUSSELS SPROUTS | Redacted4 | 6.9706 | 13.4 | A | G | 0.2601 |
| 2017 | 16-Jun-17 | MONTEREY | BRUSSELS SPROUTS | Redacted4 | 4.787 | 9.2 | A | G | 0.2602 |
| 2017 | 17-Aug-17 | MONTEREY | BRUSSELS SPROUTS | Redacted4 | 4.787 | 9.2 | A | G | 0.2602 |
| 2017 | 24-Sep-17 | MONTEREY | BRUSSELS SPROUTS | Redacted4 | 4.787 | 9.2 | A | A | 0.2602 |
| 2017 | 11-Sep-17 | MONTEREY | BRUSSELS SPROUTS | Redacted4 | 4.787 | 9.2 | A | A | 0.2602 |
| 2017 | 12-Jul-17 | MONTEREY | BRUSSELS SPROUTS | Redacted4 | 4.787 | 9.2 | A | G | 0.2602 |
| 2017 | 26-Jul-17 | MONTEREY | BRUSSELS SPROUTS | Redacted4 | 4.787 | 9.2 | A | G | 0.2602 |
| 2017 | 27-Jun-17 | MONTEREY | BRUSSELS SPROUTS | Redacted4 | 4.5351 | 8.7 | A | G | 0.2606 |
| 2017 | 9-Sep-17 | MONTEREY | BRUSSELS SPROUTS | Redacted4 | 4.5351 | 8.7 | A | G | 0.2606 |
| 2017 | 5-Sep-17 | MONTEREY | BRUSSELS SPROUTS | Redacted4 | 4.5351 | 8.7 | A | G | 0.2606 |
| 2017 | 3-Jul-17 | MONTEREY | BRUSSELS SPROUTS | Redacted4 | 4.5351 | 8.7 | A | G | 0.2606 |
| 2017 | 23-May-17 | MONTEREY | BRUSSELS SPROUTS | Redacted4 | 4.5351 | 8.7 | A | G | 0.2606 |
| 2017 | 22-Aug-17 | MONTEREY | BRUSSELS SPROUTS | Redacted4 | 4.5351 | 8.7 | A | G | 0.2606 |
| 2017 | 19-Sep-17 | MONTEREY | BRUSSELS SPROUTS | Redacted4 | 4.5351 | 8.7 | A | G | 0.2606 |
| 2017 | 28-Oct-17 | MONTEREY | BRUSSELS SPROUTS | Redacted4 | 3.0234 | 5.8 | A | G | 0.2606 |
| 2017 | 7-Nov-17 | MONTEREY | BRUSSELS SPROUTS | Redacted4 | 7.5585 | 14.5 | A | G | 0.2606 |
| 2017 | 10-Sep-17 | MONTEREY | BRUSSELS SPROUTS | Redacted4 | 3.4433 | 6.6 | A | G | 0.2609 |
| 2017 | 20-Jul-17 | MONTEREY | BRUSSELS SPROUTS | Redacted4 | 3.4433 | 6.6 | A | G | 0.2609 |
| 2017 | 12-Jul-17 | MONTEREY | BRUSSELS SPROUTS | Redacted4 | 3.4433 | 6.6 | A | G | 0.2609 |
| 2017 | 23-Jun-17 | MONTEREY | BRUSSELS SPROUTS | Redacted4 | 3.4433 | 6.6 | A | G | 0.2609 |
| 2017 | 5-Jul-17 | MONTEREY | BRUSSELS SPROUTS | Redacted4 | 5.3749 | 10.3 | A | G | 0.2609 |
| 2017 | 17-May-17 | MONTEREY | BRUSSELS SPROUTS | Redacted4 | 5.3749 | 10.3 | A | G | 0.2609 |
| 2017 | 22-Jul-17 | MONTEREY | BRUSSELS SPROUTS | Redacted4 | 5.3749 | 10.3 | A | G | 0.2609 |
| 2017 | 10-Aug-17 | MONTEREY | BRUSSELS SPROUTS | Redacted4 | 5.3749 | 10.3 | A | A | 0.2609 |
| 2017 | 25-Aug-17 | MONTEREY | BRUSSELS SPROUTS | Redacted4 | 5.3749 | 10.3 | A | G | 0.2609 |
| 2017 | 15-Jun-17 | MONTEREY | BRUSSELS SPROUTS | Redacted4 | 5.3749 | 10.3 | A | G | 0.2609 |
| 2017 | 6-Aug-17 | MONTEREY | BRUSSELS SPROUTS | Redacted4 | 6.2147 | 11.9 | A | A | 0.2611 |
| 2017 | 29-Jul-17 | MONTEREY | BRUSSELS SPROUTS | Redacted4 | 6.2147 | 11.9 | A | G | 0.2611 |
| 2017 | 25-Jul-17 | MONTEREY | BRUSSELS SPROUTS | Redacted4 | 6.2147 | 11.9 | A | G | 0.2611 |
| 2017 | 21-Jun-17 | MONTEREY | BRUSSELS SPROUTS | Redacted4 | 6.2147 | 11.9 | A | G | 0.2611 |
| 2017 | 21-Aug-17 | MONTEREY | BRUSSELS SPROUTS | Redacted4 | 6.2147 | 11.9 | A | G | 0.2611 |
| 2017 | 26-Aug-17 | MONTEREY | BRUSSELS SPROUTS | Redacted4 | 6.2147 | 11.9 | A | G | 0.2611 |
| 2017 | 17-May-17 | MONTEREY | BRUSSELS SPROUTS | Redacted4 | 6.2147 | 11.9 | A | G | 0.2611 |
| 2017 | 5-Oct-17 | MONTEREY | BRUSSELS SPROUTS | Redacted4 | 6.2147 | 11.9 | A | G | 0.2611 |
| 2017 | 23-Sep-17 | MONTEREY | BRUSSELS SPROUTS | Redacted4 | 6.2147 | 11.9 | A | G | 0.2611 |
| 2017 | 12-Jul-17 | MONTEREY | BRUSSELS SPROUTS | Redacted4 | 6.2147 | 11.9 | A | G | 0.2611 |
| 2017 | 29-Oct-17 | MONTEREY | BRUSSELS SPROUTS | Redacted4 | 4.2831 | 8.2 | A | A | 0.2612 |
| 2017 | 12-Jul-17 | MONTEREY | BRUSSELS SPROUTS | Redacted4 | 2.3515 | 4.5 | A | G | 0.2613 |
| 2017 | 20-Jul-17 | MONTEREY | BRUSSELS SPROUTS | Redacted4 | 2.3515 | 4.5 | A | G | 0.2613 |
| 2017 | 6-Jun-17 | MONTEREY | BRUSSELS SPROUTS | Redacted4 | 2.3515 | 4.5 | A | G | 0.2613 |
| 2017 | 4-Jul-17 | MONTEREY | BRUSSELS SPROUTS | Redacted4 | 2.3515 | 4.5 | A | G | 0.2613 |
| 2017 | 7-Aug-17 | MONTEREY | BRUSSELS SPROUTS | Redacted4 | 2.3515 | 4.5 | A | G | 0.2613 |
| 2017 | 14-Sep-17 | MONTEREY | BRUSSELS SPROUTS | Redacted4 | 2.3515 | 4.5 | A | G | 0.2613 |
| 2017 | 1-Jun-17 | MONTEREY | BRUSSELS SPROUTS | Redacted4 | 4.7031 | 9 | A | G | 0.2613 |
| 2017 | 25-Jul-17 | MONTEREY | BRUSSELS SPROUTS | Redacted4 | 4.7031 | 9 | A | G | 0.2613 |
| 2017 | 7-Aug-17 | MONTEREY | BRUSSELS SPROUTS | Redacted4 | 4.7031 | 9 | A | G | 0.2613 |
| 2017 | 3-Jul-17 | MONTEREY | BRUSSELS SPROUTS | Redacted4 | 4.7031 | 9 | A | G | 0.2613 |
| 2017 | 17-Aug-17 | MONTEREY | BRUSSELS SPROUTS | Redacted4 | 4.7031 | 9 | A | G | 0.2613 |
| 2017 | 19-Sep-17 | MONTEREY | BRUSSELS SPROUTS | Redacted4 | 4.7031 | 9 | A | G | 0.2613 |
| 2017 | 29-Jun-17 | MONTEREY | BRUSSELS SPROUTS | Redacted4 | 4.7031 | 9 | A | G | 0.2613 |
| 2017 | 22-Aug-17 | MONTEREY | BRUSSELS SPROUTS | Redacted4 | 4.7031 | 9 | A | G | 0.2613 |
| 2017 | 17-Jul-17 | MONTEREY | BRUSSELS SPROUTS | Redacted4 | 4.7031 | 9 | A | G | 0.2613 |
| 2017 | 13-Aug-17 | MONTEREY | BRUSSELS SPROUTS | Redacted4 | 4.7031 | 9 | A | G | 0.2613 |
| 2017 | 6-Oct-17 | MONTEREY | BRUSSELS SPROUTS | Redacted4 | 4.7031 | 9 | A | G | 0.2613 |
| 2017 | 27-Jun-17 | MONTEREY | BRUSSELS SPROUTS | Redacted4 | 4.7031 | 9 | A | G | 0.2613 |
| 2017 | 12-Sep-17 | MONTEREY | BRUSSELS SPROUTS | Redacted4 | 4.7031 | 9 | A | G | 0.2613 |
| 2017 | 21-Jul-17 | MONTEREY | BRUSSELS SPROUTS | Redacted4 | 4.7031 | 9 | A | G | 0.2613 |
| 2017 | 27-May-17 | MONTEREY | BRUSSELS SPROUTS | Redacted4 | 4.7031 | 9 | A | G | 0.2613 |
| 2017 | 8-Sep-17 | MONTEREY | BRUSSELS SPROUTS | Redacted4 | 4.7031 | 9 | A | G | 0.2613 |
| 2017 | 18-Jul-17 | MONTEREY | BRUSSELS SPROUTS | Redacted4 | 4.7031 | 9 | A | G | 0.2613 |
| 2017 | 5-Sep-17 | MONTEREY | BRUSSELS SPROUTS | Redacted4 | 4.7031 | 9 | A | G | 0.2613 |
| 2017 | 21-Aug-17 | MONTEREY | BRUSSELS SPROUTS | Redacted4 | 5.5429 | 10.6 | A | G | 0.2615 |
| 2017 | 20-Jul-17 | MONTEREY | BRUSSELS SPROUTS | Redacted4 | 5.5429 | 10.6 | A | G | 0.2615 |
| 2017 | 31-Oct-17 | MONTEREY | BRUSSELS SPROUTS | Redacted4 | 5.5429 | 10.6 | A | G | 0.2615 |
| 2017 | 23-Sep-17 | MONTEREY | BRUSSELS SPROUTS | Redacted4 | 5.5429 | 10.6 | A | G | 0.2615 |
| 2017 | 29-Aug-17 | MONTEREY | BRUSSELS SPROUTS | Redacted4 | 5.9628 | 11.4 | A | G | 0.2615 |
| 2017 | 24-Jun-17 | MONTEREY | BRUSSELS SPROUTS | Redacted4 | 5.9628 | 11.4 | A | G | 0.2615 |
| 2017 | 24-Jul-17 | MONTEREY | BRUSSELS SPROUTS | Redacted4 | 5.9628 | 11.4 | A | G | 0.2615 |
| 2017 | 6-Oct-17 | MONTEREY | BRUSSELS SPROUTS | Redacted4 | 5.9628 | 11.4 | A | G | 0.2615 |
| 2017 | 24-May-17 | MONTEREY | BRUSSELS SPROUTS | Redacted4 | 5.9628 | 11.4 | A | G | 0.2615 |
| 2017 | 21-Aug-17 | MONTEREY | BRUSSELS SPROUTS | Redacted4 | 9.1542 | 17.5 | A | G | 0.2615 |
| 2017 | 12-Jul-17 | MONTEREY | BRUSSELS SPROUTS | Redacted4 | 9.1542 | 17.5 | A | G | 0.2615 |
| 2017 | 31-Jul-17 | MONTEREY | BRUSSELS SPROUTS | Redacted4 | 9.1542 | 17.5 | A | G | 0.2615 |
| 2017 | 17-Jun-17 | MONTEREY | BRUSSELS SPROUTS | Redacted4 | 9.994 | 19.1 | A | G | 0.2616 |
| 2017 | 12-Jul-17 | MONTEREY | BRUSSELS SPROUTS | Redacted4 | 9.994 | 19.1 | A | G | 0.2616 |
| 2017 | 23-May-17 | MONTEREY | BRUSSELS SPROUTS | Redacted4 | 9.994 | 19.1 | A | G | 0.2616 |
| 2017 | 23-Sep-17 | MONTEREY | BRUSSELS SPROUTS | Redacted4 | 9.994 | 19.1 | A | G | 0.2616 |
| 2017 | 7-Oct-17 | MONTEREY | BRUSSELS SPROUTS | Redacted4 | 9.994 | 19.1 | A | G | 0.2616 |
| 2017 | 27-Jul-17 | MONTEREY | BRUSSELS SPROUTS | Redacted4 | 9.994 | 19.1 | A | G | 0.2616 |
| 2017 | 18-Aug-17 | MONTEREY | BRUSSELS SPROUTS | Redacted4 | 9.994 | 19.1 | A | G | 0.2616 |
| 2017 | 8-Sep-17 | MONTEREY | BRUSSELS SPROUTS | Redacted4 | 3.6113 | 6.9 | A | G | 0.2617 |
| 2017 | 31-May-17 | MONTEREY | BRUSSELS SPROUTS | Redacted4 | 3.6113 | 6.9 | A | G | 0.2617 |
| 2017 | 3-Nov-17 | MONTEREY | BRUSSELS SPROUTS | Redacted4 | 3.6113 | 6.9 | A | G | 0.2617 |
| 2017 | 21-Sep-17 | MONTEREY | BRUSSELS SPROUTS | Redacted4 | 3.6113 | 6.9 | A | G | 0.2617 |
| 2017 | 5-Jul-17 | MONTEREY | BRUSSELS SPROUTS | Redacted4 | 3.6113 | 6.9 | A | G | 0.2617 |
| 2017 | 28-Jul-17 | MONTEREY | BRUSSELS SPROUTS | Redacted4 | 3.6113 | 6.9 | A | G | 0.2617 |
| 2017 | 22-Sep-17 | MONTEREY | BRUSSELS SPROUTS | Redacted4 | 8.4823 | 16.2 | A | G | 0.2618 |
| 2017 | 13-Jul-17 | MONTEREY | BRUSSELS SPROUTS | Redacted4 | 8.4823 | 16.2 | A | G | 0.2618 |
| 2017 | 20-Aug-17 | MONTEREY | BRUSSELS SPROUTS | Redacted4 | 8.4823 | 16.2 | A | G | 0.2618 |
| 2017 | 23-Jun-17 | MONTEREY | BRUSSELS SPROUTS | Redacted4 | 8.4823 | 16.2 | A | G | 0.2618 |
| 2017 | 3-Jul-17 | MONTEREY | BRUSSELS SPROUTS | Redacted4 | 8.9022 | 17 | A | G | 0.2618 |
| 2017 | 21-Aug-17 | MONTEREY | BRUSSELS SPROUTS | Redacted4 | 8.9022 | 17 | A | G | 0.2618 |
| 2017 | 18-Sep-17 | MONTEREY | BRUSSELS SPROUTS | Redacted4 | 8.9022 | 17 | A | G | 0.2618 |
| 2017 | 27-Jul-17 | MONTEREY | BRUSSELS SPROUTS | Redacted4 | 8.9022 | 17 | A | G | 0.2618 |
| 2017 | 12-Aug-17 | SANTA CRUZ | BRUSSELS SPROUTS | Redacted4 | 4.4511 | 8.5 | A | G | 0.2618 |
| 2017 | 4-Sep-17 | SANTA CRUZ | BRUSSELS SPROUTS | Redacted4 | 4.4511 | 8.5 | A | G | 0.2618 |
| 2017 | 14-Jul-17 | SANTA CRUZ | BRUSSELS SPROUTS | Redacted4 | 4.4511 | 8.5 | A | G | 0.2618 |
| 2017 | 9-Sep-17 | MONTEREY | BRUSSELS SPROUTS | Redacted4 | 10.162 | 19.4 | A | G | 0.2619 |
| 2017 | 19-Aug-17 | MONTEREY | BRUSSELS SPROUTS | Redacted4 | 10.162 | 19.4 | A | G | 0.2619 |
| 2017 | 26-Jul-17 | MONTEREY | BRUSSELS SPROUTS | Redacted4 | 10.162 | 19.4 | A | G | 0.2619 |
| 2017 | 26-Aug-17 | MONTEREY | BRUSSELS SPROUTS | Redacted4 | 10.5819 | 20.2 | A | G | 0.2619 |
| 2017 | 5-Oct-17 | MONTEREY | BRUSSELS SPROUTS | Redacted4 | 10.5819 | 20.2 | A | G | 0.2619 |
| 2017 | 17-May-17 | MONTEREY | BRUSSELS SPROUTS | Redacted4 | 10.5819 | 20.2 | A | G | 0.2619 |
| 2017 | 12-Jul-17 | MONTEREY | BRUSSELS SPROUTS | Redacted4 | 10.5819 | 20.2 | A | G | 0.2619 |
| 2017 | 25-Jul-17 | MONTEREY | BRUSSELS SPROUTS | Redacted4 | 10.5819 | 20.2 | A | G | 0.2619 |
| 2017 | 21-Jun-17 | MONTEREY | BRUSSELS SPROUTS | Redacted4 | 10.5819 | 20.2 | A | G | 0.2619 |
| 2017 | 12-Jul-17 | MONTEREY | BRUSSELS SPROUTS | Redacted4 | 6.9706 | 13.3 | A | G | 0.2621 |
| 2017 | 26-Jun-17 | MONTEREY | BRUSSELS SPROUTS | Redacted4 | 6.9706 | 13.3 | A | G | 0.2621 |
| 2017 | 25-Jul-17 | MONTEREY | BRUSSELS SPROUTS | Redacted4 | 6.9706 | 13.3 | A | G | 0.2621 |
| 2017 | 22-Aug-17 | MONTEREY | BRUSSELS SPROUTS | Redacted4 | 6.9706 | 13.3 | A | G | 0.2621 |
| 2017 | 31-May-17 | MONTEREY | BRUSSELS SPROUTS | Redacted4 | 6.9706 | 13.3 | A | G | 0.2621 |
| 2017 | 11-Jul-17 | MONTEREY | BRUSSELS SPROUTS | Redacted4 | 14.3611 | 27.4 | A | G | 0.2621 |
| 2017 | 31-Oct-17 | MONTEREY | BRUSSELS SPROUTS | Redacted4 | 14.3611 | 27.4 | A | G | 0.2621 |
| 2017 | 19-Aug-17 | MONTEREY | BRUSSELS SPROUTS | Redacted4 | 14.3611 | 27.4 | A | G | 0.2621 |
| 2017 | 8-Sep-17 | MONTEREY | BRUSSELS SPROUTS | Redacted4 | 14.3611 | 27.4 | A | G | 0.2621 |
| 2017 | 21-Jul-17 | MONTEREY | BRUSSELS SPROUTS | Redacted4 | 8.2303 | 15.7 | A | G | 0.2621 |
| 2017 | 31-Oct-17 | MONTEREY | BRUSSELS SPROUTS | Redacted4 | 8.2303 | 15.7 | A | G | 0.2621 |
| 2017 | 21-Aug-17 | MONTEREY | BRUSSELS SPROUTS | Redacted4 | 8.2303 | 15.7 | A | G | 0.2621 |
| 2017 | 23-Sep-17 | MONTEREY | BRUSSELS SPROUTS | Redacted4 | 8.2303 | 15.7 | A | G | 0.2621 |
| 2017 | 29-Jun-17 | MONTEREY | BRUSSELS SPROUTS | Redacted4 | 17.1325 | 32.68 | A | G | 0.2621 |
| 2017 | 16-Jul-17 | MONTEREY | BRUSSELS SPROUTS | Redacted4 | 17.1325 | 32.68 | A | G | 0.2621 |
| 2017 | 30-Jul-17 | MONTEREY | BRUSSELS SPROUTS | Redacted4 | 17.1325 | 32.68 | A | G | 0.2621 |
| 2017 | 31-May-17 | MONTEREY | BRUSSELS SPROUTS | Redacted4 | 17.1325 | 32.68 | A | G | 0.2621 |
| 2017 | 8-Jul-17 | MONTEREY | BRUSSELS SPROUTS | Redacted4 | 4.3671 | 8.33 | A | G | 0.2621 |
| 2017 | 18-Sep-17 | MONTEREY | BRUSSELS SPROUTS | Redacted4 | 4.3671 | 8.33 | A | G | 0.2621 |
| 2017 | 8-Jul-17 | MONTEREY | BRUSSELS SPROUTS | Redacted4 | 13.3533 | 25.47 | A | G | 0.2621 |
| 2017 | 23-Aug-17 | SANTA CRUZ | BRUSSELS SPROUTS | Redacted4 | 21.4997 | 41 | A | G | 0.2622 |
| 2017 | 3-Aug-17 | SANTA CRUZ | BRUSSELS SPROUTS | Redacted4 | 21.4997 | 41 | A | G | 0.2622 |
| 2017 | 21-Sep-17 | SANTA CRUZ | BRUSSELS SPROUTS | Redacted4 | 21.4997 | 41 | A | G | 0.2622 |
| 2017 | 16-Oct-17 | SANTA CRUZ | BRUSSELS SPROUTS | Redacted4 | 21.4997 | 41 | A | G | 0.2622 |
| 2017 | 19-Oct-17 | MONTEREY | BRUSSELS SPROUTS | Redacted4 | 12.4295 | 23.7 | A | G | 0.2622 |
| 2017 | 21-Sep-17 | MONTEREY | BRUSSELS SPROUTS | Redacted4 | 12.4295 | 23.7 | A | G | 0.2622 |
| 2017 | 19-Aug-17 | MONTEREY | BRUSSELS SPROUTS | Redacted4 | 12.4295 | 23.7 | A | G | 0.2622 |
| 2017 | 2-Aug-17 | MONTEREY | BRUSSELS SPROUTS | Redacted4 | 12.4295 | 23.7 | A | G | 0.2622 |
| 2017 | 30-Oct-17 | MONTEREY | BRUSSELS SPROUTS | Redacted4 | 12.4295 | 23.7 | A | G | 0.2622 |
| 2017 | 18-Jul-17 | MONTEREY | BRUSSELS SPROUTS | Redacted4 | 12.4295 | 23.7 | A | G | 0.2622 |
| 2017 | 6-Aug-17 | MONTEREY | BRUSSELS SPROUTS | Redacted4 | 6.6347 | 12.65 | A | G | 0.2622 |
| 2017 | 7-Sep-17 | MONTEREY | BRUSSELS SPROUTS | Redacted4 | 6.6347 | 12.65 | A | G | 0.2622 |
| 2017 | 21-Jul-17 | MONTEREY | BRUSSELS SPROUTS | Redacted4 | 14.1092 | 26.9 | A | G | 0.2623 |
| 2017 | 19-Sep-17 | MONTEREY | BRUSSELS SPROUTS | Redacted4 | 14.1092 | 26.9 | A | G | 0.2623 |
| 2017 | 28-Jun-17 | MONTEREY | BRUSSELS SPROUTS | Redacted4 | 14.1092 | 26.9 | A | G | 0.2623 |
| 2017 | 20-Aug-17 | MONTEREY | BRUSSELS SPROUTS | Redacted4 | 14.1092 | 26.9 | A | G | 0.2623 |
| 2017 | 6-Sep-17 | MONTEREY | BRUSSELS SPROUTS | Redacted4 | 14.1092 | 26.9 | A | G | 0.2623 |
| 2017 | 30-Oct-17 | MONTEREY | BRUSSELS SPROUTS | Redacted4 | 15.7888 | 30.1 | A | G | 0.2623 |
| 2017 | 21-Sep-17 | MONTEREY | BRUSSELS SPROUTS | Redacted4 | 15.7888 | 30.1 | A | G | 0.2623 |
| 2017 | 22-Aug-17 | MONTEREY | BRUSSELS SPROUTS | Redacted4 | 15.7888 | 30.1 | A | G | 0.2623 |
| 2017 | 26-Jul-17 | MONTEREY | BRUSSELS SPROUTS | Redacted4 | 15.7888 | 30.1 | A | G | 0.2623 |
| 2017 | 7-Sep-17 | MONTEREY | BRUSSELS SPROUTS | Redacted4 | 3.3593 | 6.4 | A | G | 0.2624 |
| 2017 | 11-Jul-17 | MONTEREY | BRUSSELS SPROUTS | Redacted4 | 3.3593 | 6.4 | A | G | 0.2624 |
| 2017 | 23-May-17 | MONTEREY | BRUSSELS SPROUTS | Redacted4 | 3.3593 | 6.4 | A | G | 0.2624 |
| 2017 | 22-Aug-17 | MONTEREY | BRUSSELS SPROUTS | Redacted4 | 3.3593 | 6.4 | A | G | 0.2624 |
| 2017 | 16-Jun-17 | MONTEREY | BRUSSELS SPROUTS | Redacted4 | 6.2987 | 12 | A | G | 0.2624 |
| 2017 | 12-Jul-17 | MONTEREY | BRUSSELS SPROUTS | Redacted4 | 6.2987 | 12 | A | G | 0.2624 |
| 2017 | 10-Aug-17 | MONTEREY | BRUSSELS SPROUTS | Redacted4 | 6.2987 | 12 | A | A | 0.2624 |
| 2017 | 14-Sep-17 | MONTEREY | BRUSSELS SPROUTS | Redacted4 | 6.2987 | 12 | A | G | 0.2624 |
| 2017 | 27-Jul-17 | MONTEREY | BRUSSELS SPROUTS | Redacted4 | 6.2987 | 12 | A | G | 0.2624 |
| 2017 | 3-Jun-17 | MONTEREY | BRUSSELS SPROUTS | Redacted4 | 6.2987 | 12 | A | G | 0.2624 |
| 2017 | 9-Sep-17 | MONTEREY | BRUSSELS SPROUTS | Redacted4 | 6.2987 | 12 | A | G | 0.2624 |
| 2017 | 24-Aug-17 | MONTEREY | BRUSSELS SPROUTS | Redacted4 | 6.2987 | 12 | A | G | 0.2624 |
| 2017 | 22-Oct-17 | SANTA CRUZ | BRUSSELS SPROUTS | Redacted4 | 6.2987 | 12 | A | G | 0.2624 |
| 2017 | 14-Aug-17 | SANTA CRUZ | BRUSSELS SPROUTS | Redacted4 | 6.2987 | 12 | A | G | 0.2624 |
| 2017 | 23-Jul-17 | SANTA CRUZ | BRUSSELS SPROUTS | Redacted4 | 6.2987 | 12 | A | G | 0.2624 |
| 2017 | 1-Sep-17 | SANTA CRUZ | BRUSSELS SPROUTS | Redacted4 | 6.2987 | 12 | A | G | 0.2624 |
| 2017 | 28-Sep-17 | SANTA CRUZ | BRUSSELS SPROUTS | Redacted4 | 6.2987 | 12 | A | G | 0.2624 |
| 2017 | 22-Aug-17 | MONTEREY | BRUSSELS SPROUTS | Redacted4 | 9.2381 | 17.6 | A | G | 0.2624 |
| 2017 | 6-Sep-17 | MONTEREY | BRUSSELS SPROUTS | Redacted4 | 9.2381 | 17.6 | A | G | 0.2624 |
| 2017 | 11-Jul-17 | MONTEREY | BRUSSELS SPROUTS | Redacted4 | 9.2381 | 17.6 | A | G | 0.2624 |
| 2017 | 25-Jul-17 | MONTEREY | BRUSSELS SPROUTS | Redacted4 | 9.2381 | 17.6 | A | G | 0.2624 |
| 2017 | 18-Jul-17 | MONTEREY | BRUSSELS SPROUTS | Redacted4 | 9.2381 | 17.6 | A | G | 0.2624 |
| 2017 | 4-Aug-17 | MONTEREY | BRUSSELS SPROUTS | Redacted4 | 2.9394 | 5.6 | A | G | 0.2624 |
| 2017 | 20-Jul-17 | MONTEREY | BRUSSELS SPROUTS | Redacted4 | 2.9394 | 5.6 | A | G | 0.2624 |
| 2017 | 7-Sep-17 | MONTEREY | BRUSSELS SPROUTS | Redacted4 | 5.8788 | 11.2 | A | G | 0.2624 |
| 2017 | 19-Aug-17 | MONTEREY | BRUSSELS SPROUTS | Redacted4 | 2.9394 | 5.6 | A | G | 0.2624 |
| 2017 | 2-Nov-17 | MONTEREY | BRUSSELS SPROUTS | Redacted4 | 2.9394 | 5.6 | A | G | 0.2624 |
| 2017 | 6-Sep-17 | MONTEREY | BRUSSELS SPROUTS | Redacted4 | 2.9394 | 5.6 | A | G | 0.2624 |
| 2017 | 15-Sep-17 | MONTEREY | BRUSSELS SPROUTS | Redacted4 | 8.3983 | 16 | A | G | 0.2624 |
| 2017 | 7-Aug-17 | MONTEREY | BRUSSELS SPROUTS | Redacted4 | 8.3983 | 16 | A | G | 0.2624 |
| 2017 | 11-Jul-17 | MONTEREY | BRUSSELS SPROUTS | Redacted4 | 8.3983 | 16 | A | G | 0.2624 |
| 2017 | 11-Jul-17 | MONTEREY | BRUSSELS SPROUTS | Redacted4 | 5.4589 | 10.4 | A | G | 0.2624 |
| 2017 | 17-Jun-17 | MONTEREY | BRUSSELS SPROUTS | Redacted4 | 5.4589 | 10.4 | A | G | 0.2624 |
| 2017 | 23-Jul-17 | MONTEREY | BRUSSELS SPROUTS | Redacted4 | 5.4589 | 10.4 | A | G | 0.2624 |
| 2017 | 25-Sep-17 | MONTEREY | BRUSSELS SPROUTS | Redacted4 | 5.4589 | 10.4 | A | G | 0.2624 |
| 2017 | 18-Aug-17 | MONTEREY | BRUSSELS SPROUTS | Redacted4 | 5.4589 | 10.4 | A | G | 0.2624 |
| 2017 | 19-Jul-17 | MONTEREY | BRUSSELS SPROUTS | Redacted4 | 5.4589 | 10.4 | A | G | 0.2624 |
| 2017 | 6-Sep-17 | MONTEREY | BRUSSELS SPROUTS | Redacted4 | 5.4589 | 10.4 | A | G | 0.2624 |
| 2017 | 11-Jul-17 | MONTEREY | BRUSSELS SPROUTS | Redacted4 | 7.9784 | 15.2 | A | G | 0.2624 |
| 2017 | 26-Aug-17 | MONTEREY | BRUSSELS SPROUTS | Redacted4 | 7.9784 | 15.2 | A | G | 0.2624 |
| 2017 | 24-May-17 | MONTEREY | BRUSSELS SPROUTS | Redacted4 | 7.9784 | 15.2 | A | G | 0.2624 |
| 2017 | 24-Jul-17 | MONTEREY | BRUSSELS SPROUTS | Redacted4 | 7.9784 | 15.2 | A | G | 0.2624 |
| 2017 | 7-Sep-17 | MONTEREY | BRUSSELS SPROUTS | Redacted4 | 10.4979 | 20 | A | G | 0.2624 |
| 2017 | 4-Aug-17 | MONTEREY | BRUSSELS SPROUTS | Redacted4 | 10.4979 | 20 | A | G | 0.2624 |
| 2017 | 10-Jul-17 | MONTEREY | BRUSSELS SPROUTS | Redacted4 | 10.4979 | 20 | A | G | 0.2624 |
| 2017 | 28-Jul-17 | MONTEREY | BRUSSELS SPROUTS | Redacted4 | 10.4979 | 20 | A | G | 0.2624 |
| 2017 | 26-Aug-17 | MONTEREY | BRUSSELS SPROUTS | Redacted4 | 10.4979 | 20 | A | G | 0.2624 |
| 2017 | 5-Oct-17 | MONTEREY | BRUSSELS SPROUTS | Redacted4 | 10.4979 | 20 | A | G | 0.2624 |
| 2017 | 8-Aug-17 | MONTEREY | BRUSSELS SPROUTS | Redacted4 | 10.4979 | 20 | A | G | 0.2624 |
| 2017 | 16-Jun-17 | MONTEREY | BRUSSELS SPROUTS | Redacted4 | 10.4979 | 20 | A | G | 0.2624 |
| 2017 | 9-Sep-17 | MONTEREY | BRUSSELS SPROUTS | Redacted4 | 10.4979 | 20 | A | G | 0.2624 |
| 2017 | 17-Jun-17 | MONTEREY | BRUSSELS SPROUTS | Redacted4 | 10.4979 | 20 | A | G | 0.2624 |
| 2017 | 26-Jul-17 | MONTEREY | BRUSSELS SPROUTS | Redacted4 | 10.4979 | 20 | A | G | 0.2624 |
| 2017 | 4-Jul-17 | SANTA CRUZ | BRUSSELS SPROUTS | Redacted4 | 10.4979 | 20 | A | G | 0.2624 |
| 2017 | 6-Sep-17 | SANTA CRUZ | BRUSSELS SPROUTS | Redacted4 | 10.4979 | 20 | A | G | 0.2624 |
| 2017 | 16-Aug-17 | SANTA CRUZ | BRUSSELS SPROUTS | Redacted4 | 10.4979 | 20 | A | G | 0.2624 |
| 2017 | 27-Jul-17 | SANTA CRUZ | BRUSSELS SPROUTS | Redacted4 | 10.4979 | 20 | A | G | 0.2624 |
| 2017 | 2-Oct-17 | SANTA CRUZ | BRUSSELS SPROUTS | Redacted4 | 10.4979 | 20 | A | G | 0.2624 |
| 2017 | 28-Sep-17 | SANTA CRUZ | BRUSSELS SPROUTS | Redacted4 | 12.5975 | 24 | A | G | 0.2624 |
| 2017 | 22-Oct-17 | SANTA CRUZ | BRUSSELS SPROUTS | Redacted4 | 12.5975 | 24 | A | G | 0.2624 |
| 2017 | 14-Aug-17 | SANTA CRUZ | BRUSSELS SPROUTS | Redacted4 | 12.5975 | 24 | A | G | 0.2624 |
| 2017 | 23-Jul-17 | SANTA CRUZ | BRUSSELS SPROUTS | Redacted4 | 12.5975 | 24 | A | G | 0.2624 |
| 2017 | 1-Sep-17 | SANTA CRUZ | BRUSSELS SPROUTS | Redacted4 | 12.5975 | 24 | A | G | 0.2624 |
| 2017 | 16-Jun-17 | MONTEREY | BRUSSELS SPROUTS | Redacted4 | 7.1386 | 13.6 | A | G | 0.2624 |
| 2017 | 19-Aug-17 | MONTEREY | BRUSSELS SPROUTS | Redacted4 | 7.1386 | 13.6 | A | G | 0.2624 |
| 2017 | 26-Jul-17 | MONTEREY | BRUSSELS SPROUTS | Redacted4 | 7.1386 | 13.6 | A | G | 0.2624 |
| 2017 | 13-Jul-17 | MONTEREY | BRUSSELS SPROUTS | Redacted4 | 7.1386 | 13.6 | A | G | 0.2624 |
| 2017 | 17-Jul-17 | MONTEREY | BRUSSELS SPROUTS | Redacted4 | 4.6191 | 8.8 | A | G | 0.2624 |
| 2017 | 17-May-17 | MONTEREY | BRUSSELS SPROUTS | Redacted4 | 4.6191 | 8.8 | A | G | 0.2624 |
| 2017 | 4-Jul-17 | MONTEREY | BRUSSELS SPROUTS | Redacted4 | 4.6191 | 8.8 | A | G | 0.2624 |
| 2017 | 23-Jul-17 | MONTEREY | BRUSSELS SPROUTS | Redacted4 | 4.6191 | 8.8 | A | G | 0.2624 |
| 2017 | 5-Sep-17 | MONTEREY | BRUSSELS SPROUTS | Redacted4 | 4.6191 | 8.8 | A | G | 0.2624 |
| 2017 | 15-Jun-17 | MONTEREY | BRUSSELS SPROUTS | Redacted4 | 4.6191 | 8.8 | A | G | 0.2624 |
| 2017 | 22-Aug-17 | MONTEREY | BRUSSELS SPROUTS | Redacted4 | 4.6191 | 8.8 | A | G | 0.2624 |
| 2017 | 5-Aug-17 | MONTEREY | BRUSSELS SPROUTS | Redacted4 | 4.1992 | 8 | A | G | 0.2625 |
| 2017 | 25-May-17 | MONTEREY | BRUSSELS SPROUTS | Redacted4 | 4.1992 | 8 | A | G | 0.2625 |
| 2017 | 16-Jul-17 | MONTEREY | BRUSSELS SPROUTS | Redacted4 | 4.1992 | 8 | A | G | 0.2625 |
| 2017 | 11-Sep-17 | MONTEREY | BRUSSELS SPROUTS | Redacted4 | 4.1992 | 8 | A | G | 0.2625 |
| 2017 | 20-Jun-17 | MONTEREY | BRUSSELS SPROUTS | Redacted4 | 4.1992 | 8 | A | G | 0.2625 |
| 2017 | 5-Aug-17 | SANTA CRUZ | BRUSSELS SPROUTS | Redacted4 | 4.1992 | 8 | A | G | 0.2625 |
| 2017 | 16-Jul-17 | MONTEREY | BRUSSELS SPROUTS | Redacted4 | 16.1247 | 30.7 | A | G | 0.2626 |
| 2017 | 6-Jun-17 | MONTEREY | BRUSSELS SPROUTS | Redacted4 | 16.1247 | 30.7 | A | G | 0.2626 |
| 2017 | 11-Aug-17 | MONTEREY | BRUSSELS SPROUTS | Redacted4 | 16.1247 | 30.7 | A | G | 0.2626 |
| 2017 | 28-Aug-17 | MONTEREY | BRUSSELS SPROUTS | Redacted4 | 9.826 | 18.7 | A | G | 0.2627 |
| 2017 | 3-Jun-17 | MONTEREY | BRUSSELS SPROUTS | Redacted4 | 9.826 | 18.7 | A | G | 0.2627 |
| 2017 | 1-Jul-17 | MONTEREY | BRUSSELS SPROUTS | Redacted4 | 9.826 | 18.7 | A | G | 0.2627 |
| 2017 | 21-Aug-17 | MONTEREY | BRUSSELS SPROUTS | Redacted4 | 9.826 | 18.7 | A | G | 0.2627 |
| 2017 | 6-Oct-17 | MONTEREY | BRUSSELS SPROUTS | Redacted4 | 9.826 | 18.7 | A | G | 0.2627 |
| 2017 | 21-Jul-17 | MONTEREY | BRUSSELS SPROUTS | Redacted4 | 9.826 | 18.7 | A | G | 0.2627 |
| 2017 | 12-Sep-17 | MONTEREY | BRUSSELS SPROUTS | Redacted4 | 9.826 | 18.7 | A | G | 0.2627 |
| 2017 | 29-Jul-17 | SANTA CRUZ | BRUSSELS SPROUTS | Redacted4 | 16.2927 | 31 | A | G | 0.2628 |
| 2017 | 12-Jul-17 | SANTA CRUZ | BRUSSELS SPROUTS | Redacted4 | 16.2927 | 31 | A | G | 0.2628 |
| 2017 | 11-Aug-17 | SANTA CRUZ | BRUSSELS SPROUTS | Redacted4 | 16.2927 | 31 | A | G | 0.2628 |
| 2017 | 14-Nov-17 | SANTA CRUZ | BRUSSELS SPROUTS | Redacted4 | 16.2927 | 31 | A | G | 0.2628 |
| 2017 | 19-Sep-17 | SANTA CRUZ | BRUSSELS SPROUTS | Redacted4 | 16.2927 | 31 | A | G | 0.2628 |
| 2017 | 14-Oct-17 | SANTA CRUZ | BRUSSELS SPROUTS | Redacted4 | 16.2927 | 31 | A | G | 0.2628 |
| 2017 | 10-Sep-17 | MONTEREY | BRUSSELS SPROUTS | Redacted4 | 8.1464 | 15.5 | A | G | 0.2628 |
| 2017 | 23-Aug-17 | MONTEREY | BRUSSELS SPROUTS | Redacted4 | 8.1464 | 15.5 | A | G | 0.2628 |
| 2017 | 11-Jul-17 | MONTEREY | BRUSSELS SPROUTS | Redacted4 | 8.1464 | 15.5 | A | G | 0.2628 |
| 2017 | 22-Jun-17 | MONTEREY | BRUSSELS SPROUTS | Redacted4 | 6.4667 | 12.3 | A | G | 0.2629 |
| 2017 | 6-Sep-17 | MONTEREY | BRUSSELS SPROUTS | Redacted4 | 6.4667 | 12.3 | A | G | 0.2629 |
| 2017 | 2-Nov-17 | MONTEREY | BRUSSELS SPROUTS | Redacted4 | 6.4667 | 12.3 | A | G | 0.2629 |
| 2017 | 10-Aug-17 | MONTEREY | BRUSSELS SPROUTS | Redacted4 | 6.4667 | 12.3 | A | A | 0.2629 |
| 2017 | 18-Jul-17 | MONTEREY | BRUSSELS SPROUTS | Redacted4 | 6.4667 | 12.3 | A | G | 0.2629 |
| 2017 | 24-Jul-17 | MONTEREY | BRUSSELS SPROUTS | Redacted4 | 6.4667 | 12.3 | A | G | 0.2629 |
| 2017 | 15-Jul-17 | MONTEREY | BRUSSELS SPROUTS | Redacted4 | 6.0468 | 11.5 | A | G | 0.2629 |
| 2017 | 5-Sep-17 | MONTEREY | BRUSSELS SPROUTS | Redacted4 | 6.0468 | 11.5 | A | G | 0.2629 |
| 2017 | 2-Nov-17 | MONTEREY | BRUSSELS SPROUTS | Redacted4 | 6.0468 | 11.5 | A | G | 0.2629 |
| 2017 | 2-Aug-17 | MONTEREY | BRUSSELS SPROUTS | Redacted4 | 5.6269 | 10.7 | A | G | 0.2629 |
| 2017 | 6-Aug-17 | MONTEREY | BRUSSELS SPROUTS | Redacted4 | 5.4589 | 10.38 | A | G | 0.2630 |
| 2017 | 7-Sep-17 | MONTEREY | BRUSSELS SPROUTS | Redacted4 | 5.4589 | 10.38 | A | G | 0.2630 |
| 2017 | 7-Sep-17 | MONTEREY | BRUSSELS SPROUTS | Redacted4 | 5.2069 | 9.9 | A | G | 0.2630 |
| 2017 | 30-Oct-17 | MONTEREY | BRUSSELS SPROUTS | Redacted4 | 5.2069 | 9.9 | A | G | 0.2630 |
| 2017 | 22-Aug-17 | MONTEREY | BRUSSELS SPROUTS | Redacted4 | 5.2069 | 9.9 | A | G | 0.2630 |
| 2017 | 17-Sep-17 | MONTEREY | BRUSSELS SPROUTS | Redacted4 | 5.2069 | 9.9 | A | G | 0.2630 |
| 2017 | 19-Jul-17 | MONTEREY | BRUSSELS SPROUTS | Redacted4 | 5.2069 | 9.9 | A | G | 0.2630 |
| 2017 | 1-Jul-17 | MONTEREY | BRUSSELS SPROUTS | Redacted4 | 5.2069 | 9.9 | A | G | 0.2630 |
| 2017 | 21-Aug-17 | MONTEREY | BRUSSELS SPROUTS | Redacted4 | 5.2069 | 9.9 | A | G | 0.2630 |
| 2017 | 7-Jun-17 | MONTEREY | BRUSSELS SPROUTS | Redacted4 | 5.2069 | 9.9 | A | G | 0.2630 |
| 2017 | 7-Aug-17 | MONTEREY | BRUSSELS SPROUTS | Redacted4 | 9.4901 | 18.04 | A | G | 0.2630 |
| 2017 | 7-Sep-17 | MONTEREY | BRUSSELS SPROUTS | Redacted4 | 9.4901 | 18.04 | A | G | 0.2630 |
| 2017 | 11-Sep-17 | MONTEREY | BRUSSELS SPROUTS | Redacted4 | 9.1542 | 17.4 | A | G | 0.2631 |
| 2017 | 11-Jul-17 | MONTEREY | BRUSSELS SPROUTS | Redacted4 | 9.1542 | 17.4 | A | G | 0.2631 |
| 2017 | 23-Aug-17 | MONTEREY | BRUSSELS SPROUTS | Redacted4 | 9.1542 | 17.4 | A | G | 0.2631 |
| 2017 | 14-Jul-17 | MONTEREY | BRUSSELS SPROUTS | Redacted4 | 7.8944 | 15 | A | G | 0.2631 |
| 2017 | 25-May-17 | MONTEREY | BRUSSELS SPROUTS | Redacted4 | 7.8944 | 15 | A | G | 0.2631 |
| 2017 | 11-Sep-17 | MONTEREY | BRUSSELS SPROUTS | Redacted4 | 7.8944 | 15 | A | G | 0.2631 |
| 2017 | 20-Jun-17 | MONTEREY | BRUSSELS SPROUTS | Redacted4 | 7.8944 | 15 | A | G | 0.2631 |
| 2017 | 1-Aug-17 | MONTEREY | BRUSSELS SPROUTS | Redacted4 | 15.7888 | 30 | A | G | 0.2631 |
| 2017 | 5-Aug-17 | MONTEREY | BRUSSELS SPROUTS | Redacted4 | 7.8944 | 15 | A | G | 0.2631 |
| 2017 | 12-Jul-17 | MONTEREY | BRUSSELS SPROUTS | Redacted4 | 7.4745 | 14.2 | A | G | 0.2632 |
| 2017 | 19-Aug-17 | MONTEREY | BRUSSELS SPROUTS | Redacted4 | 7.4745 | 14.2 | A | G | 0.2632 |
| 2017 | 26-Jul-17 | MONTEREY | BRUSSELS SPROUTS | Redacted4 | 7.4745 | 14.2 | A | G | 0.2632 |
| 2017 | 16-Jun-17 | MONTEREY | BRUSSELS SPROUTS | Redacted4 | 7.4745 | 14.2 | A | G | 0.2632 |
| 2017 | 24-Jun-17 | MONTEREY | BRUSSELS SPROUTS | Redacted4 | 3.5273 | 6.7 | A | G | 0.2632 |
| 2017 | 6-Oct-17 | MONTEREY | BRUSSELS SPROUTS | Redacted4 | 3.5273 | 6.7 | A | G | 0.2632 |
| 2017 | 23-Jul-17 | MONTEREY | BRUSSELS SPROUTS | Redacted4 | 3.5273 | 6.7 | A | G | 0.2632 |
| 2017 | 10-Aug-17 | MONTEREY | BRUSSELS SPROUTS | Redacted4 | 3.5273 | 6.7 | A | A | 0.2632 |
| 2017 | 24-May-17 | MONTEREY | BRUSSELS SPROUTS | Redacted4 | 3.5273 | 6.7 | A | G | 0.2632 |
| 2017 | 24-Aug-17 | MONTEREY | BRUSSELS SPROUTS | Redacted4 | 3.5273 | 6.7 | A | G | 0.2632 |
| 2017 | 8-Sep-17 | SANTA CRUZ | BRUSSELS SPROUTS | Redacted4 | 13.6892 | 26 | A | G | 0.2633 |
| 2017 | 2-Nov-17 | SANTA CRUZ | BRUSSELS SPROUTS | Redacted4 | 13.6892 | 26 | A | G | 0.2633 |
| 2017 | 6-Jul-17 | SANTA CRUZ | BRUSSELS SPROUTS | Redacted4 | 13.6892 | 26 | A | G | 0.2633 |
| 2017 | 10-Aug-17 | SANTA CRUZ | BRUSSELS SPROUTS | Redacted4 | 13.6892 | 26 | A | G | 0.2633 |
| 2017 | 21-Jul-17 | SANTA CRUZ | BRUSSELS SPROUTS | Redacted4 | 13.6892 | 26 | A | G | 0.2633 |
| 2017 | 28-Aug-17 | SANTA CRUZ | BRUSSELS SPROUTS | Redacted4 | 13.6892 | 26 | A | G | 0.2633 |
| 2017 | 5-Oct-17 | SANTA CRUZ | BRUSSELS SPROUTS | Redacted4 | 13.6892 | 26 | A | G | 0.2633 |
| 2017 | 4-Aug-17 | SANTA CRUZ | BRUSSELS SPROUTS | Redacted4 | 13.6892 | 26 | A | G | 0.2633 |
| 2017 | 25-Oct-17 | SANTA CRUZ | BRUSSELS SPROUTS | Redacted4 | 13.6892 | 26 | A | G | 0.2633 |
| 2017 | 25-Nov-17 | SANTA CRUZ | BRUSSELS SPROUTS | Redacted4 | 13.6892 | 26 | A | G | 0.2633 |
| 2017 | 12-Oct-17 | SANTA CRUZ | BRUSSELS SPROUTS | Redacted4 | 13.6892 | 26 | A | G | 0.2633 |
| 2017 | 5-Sep-17 | SANTA CRUZ | BRUSSELS SPROUTS | Redacted4 | 13.6892 | 26 | A | G | 0.2633 |
| 2017 | 26-Sep-17 | SANTA CRUZ | BRUSSELS SPROUTS | Redacted4 | 13.6892 | 26 | A | G | 0.2633 |
| 2017 | 11-Aug-17 | SANTA CRUZ | BRUSSELS SPROUTS | Redacted4 | 13.6892 | 26 | A | G | 0.2633 |
| 2017 | 9-Sep-17 | SANTA CRUZ | BRUSSELS SPROUTS | Redacted4 | 13.6892 | 26 | A | G | 0.2633 |
| 2017 | 1-Jul-17 | MONTEREY | BRUSSELS SPROUTS | Redacted4 | 10.162 | 19.3 | A | G | 0.2633 |
| 2017 | 20-Jul-17 | MONTEREY | BRUSSELS SPROUTS | Redacted4 | 10.162 | 19.3 | A | G | 0.2633 |
| 2017 | 31-May-17 | MONTEREY | BRUSSELS SPROUTS | Redacted4 | 10.162 | 19.3 | A | G | 0.2633 |
| 2017 | 20-Sep-17 | MONTEREY | BRUSSELS SPROUTS | Redacted4 | 10.162 | 19.3 | A | G | 0.2633 |
| 2017 | 26-Aug-17 | MONTEREY | BRUSSELS SPROUTS | Redacted4 | 10.162 | 19.3 | A | G | 0.2633 |
| 2017 | 8-Jul-17 | SANTA CRUZ | BRUSSELS SPROUTS | Redacted4 | 9.742 | 18.5 | A | G | 0.2633 |
| 2017 | 8-Aug-17 | SANTA CRUZ | BRUSSELS SPROUTS | Redacted4 | 9.742 | 18.5 | A | G | 0.2633 |
| 2017 | 29-Jul-17 | SANTA CRUZ | BRUSSELS SPROUTS | Redacted4 | 9.742 | 18.5 | A | G | 0.2633 |
| 2017 | 19-Sep-17 | SANTA CRUZ | BRUSSELS SPROUTS | Redacted4 | 9.742 | 18.5 | A | G | 0.2633 |
| 2017 | 2-Nov-17 | SANTA CRUZ | BRUSSELS SPROUTS | Redacted4 | 9.742 | 18.5 | A | G | 0.2633 |
| 2017 | 16-Oct-17 | SANTA CRUZ | BRUSSELS SPROUTS | Redacted4 | 9.742 | 18.5 | A | G | 0.2633 |
| 2017 | 27-May-17 | MONTEREY | BRUSSELS SPROUTS | Redacted4 | 5.7948 | 11 | A | G | 0.2634 |
| 2017 | 6-Jul-17 | MONTEREY | BRUSSELS SPROUTS | Redacted4 | 5.7948 | 11 | A | G | 0.2634 |
| 2017 | 3-Aug-17 | MONTEREY | BRUSSELS SPROUTS | Redacted4 | 5.7948 | 11 | A | G | 0.2634 |
| 2017 | 6-Sep-17 | MONTEREY | BRUSSELS SPROUTS | Redacted4 | 5.7948 | 11 | A | G | 0.2634 |
| 2017 | 28-Jul-17 | MONTEREY | BRUSSELS SPROUTS | Redacted4 | 5.7948 | 11 | A | G | 0.2634 |
| 2017 | 28-Jun-17 | MONTEREY | BRUSSELS SPROUTS | Redacted4 | 11.5897 | 22 | A | G | 0.2634 |
| 2017 | 9-Sep-17 | MONTEREY | BRUSSELS SPROUTS | Redacted4 | 11.5897 | 22 | A | G | 0.2634 |
| 2017 | 22-Aug-17 | MONTEREY | BRUSSELS SPROUTS | Redacted4 | 11.5897 | 22 | A | G | 0.2634 |
| 2017 | 31-May-17 | MONTEREY | BRUSSELS SPROUTS | Redacted4 | 11.5897 | 22 | A | G | 0.2634 |
| 2017 | 17-Jul-17 | MONTEREY | BRUSSELS SPROUTS | Redacted4 | 11.5897 | 22 | A | G | 0.2634 |
| 2017 | 25-Nov-17 | SANTA CRUZ | BRUSSELS SPROUTS | Redacted4 | 11.5897 | 22 | A | G | 0.2634 |
| 2017 | 6-Sep-17 | SANTA CRUZ | BRUSSELS SPROUTS | Redacted4 | 11.5897 | 22 | A | G | 0.2634 |
| 2017 | 1-Oct-17 | SANTA CRUZ | BRUSSELS SPROUTS | Redacted4 | 11.5897 | 22 | A | G | 0.2634 |
| 2017 | 29-Oct-17 | SANTA CRUZ | BRUSSELS SPROUTS | Redacted4 | 11.5897 | 22 | A | G | 0.2634 |
| 2017 | 16-Aug-17 | SANTA CRUZ | BRUSSELS SPROUTS | Redacted4 | 11.5897 | 22 | A | G | 0.2634 |
| 2017 | 11-Jul-17 | MONTEREY | BRUSSELS SPROUTS | Redacted4 | 8.0624 | 15.3 | A | G | 0.2635 |
| 2017 | 22-Sep-17 | MONTEREY | BRUSSELS SPROUTS | Redacted4 | 8.0624 | 15.3 | A | G | 0.2635 |
| 2017 | 18-Aug-17 | MONTEREY | BRUSSELS SPROUTS | Redacted4 | 8.0624 | 15.3 | A | G | 0.2635 |
| 2017 | 18-Jul-17 | MONTEREY | BRUSSELS SPROUTS | Redacted4 | 8.0624 | 15.3 | A | G | 0.2635 |
| 2017 | 23-May-17 | MONTEREY | BRUSSELS SPROUTS | Redacted4 | 8.0624 | 15.3 | A | G | 0.2635 |
| 2017 | 25-Jul-17 | MONTEREY | BRUSSELS SPROUTS | Redacted4 | 8.0624 | 15.3 | A | G | 0.2635 |
| 2017 | 23-Jun-17 | MONTEREY | BRUSSELS SPROUTS | Redacted4 | 8.0624 | 15.3 | A | G | 0.2635 |
| 2017 | 22-Aug-17 | MONTEREY | BRUSSELS SPROUTS | Redacted4 | 10.3299 | 19.6 | A | G | 0.2635 |
| 2017 | 10-Aug-17 | MONTEREY | BRUSSELS SPROUTS | Redacted4 | 10.3299 | 19.6 | A | G | 0.2635 |
| 2017 | 17-Jun-17 | MONTEREY | BRUSSELS SPROUTS | Redacted4 | 10.3299 | 19.6 | A | G | 0.2635 |
| 2017 | 5-Aug-17 | MONTEREY | BRUSSELS SPROUTS | Redacted4 | 10.3299 | 19.6 | A | G | 0.2635 |
| 2017 | 12-Jul-17 | MONTEREY | BRUSSELS SPROUTS | Redacted4 | 10.3299 | 19.6 | A | G | 0.2635 |
| 2017 | 26-Jul-17 | MONTEREY | BRUSSELS SPROUTS | Redacted4 | 7.6425 | 14.5 | A | G | 0.2635 |
| 2017 | 7-Jun-17 | MONTEREY | BRUSSELS SPROUTS | Redacted4 | 7.6425 | 14.5 | A | G | 0.2635 |
| 2017 | 1-Jul-17 | MONTEREY | BRUSSELS SPROUTS | Redacted4 | 7.6425 | 14.5 | A | G | 0.2635 |
| 2017 | 19-Jul-17 | MONTEREY | BRUSSELS SPROUTS | Redacted4 | 7.6425 | 14.5 | A | G | 0.2635 |
| 2017 | 6-Sep-17 | MONTEREY | BRUSSELS SPROUTS | Redacted4 | 7.6425 | 14.5 | A | G | 0.2635 |
| 2017 | 6-Oct-17 | MONTEREY | BRUSSELS SPROUTS | Redacted4 | 4.955 | 9.4 | A | G | 0.2636 |
| 2017 | 25-Aug-17 | MONTEREY | BRUSSELS SPROUTS | Redacted4 | 4.955 | 9.4 | A | G | 0.2636 |
| 2017 | 24-May-17 | MONTEREY | BRUSSELS SPROUTS | Redacted4 | 4.955 | 9.4 | A | G | 0.2636 |
| 2017 | 2-Nov-17 | MONTEREY | BRUSSELS SPROUTS | Redacted4 | 4.955 | 9.4 | A | G | 0.2636 |
| 2017 | 24-Jul-17 | MONTEREY | BRUSSELS SPROUTS | Redacted4 | 4.955 | 9.4 | A | G | 0.2636 |
| 2017 | 10-Aug-17 | MONTEREY | BRUSSELS SPROUTS | Redacted4 | 9.91 | 18.8 | A | A | 0.2636 |
| 2017 | 6-Sep-17 | MONTEREY | BRUSSELS SPROUTS | Redacted4 | 4.955 | 9.4 | A | G | 0.2636 |
| 2017 | 13-Jun-17 | MONTEREY | BRUSSELS SPROUTS | Redacted4 | 9.91 | 18.8 | A | G | 0.2636 |
| 2017 | 24-Jun-17 | MONTEREY | BRUSSELS SPROUTS | Redacted4 | 4.955 | 9.4 | A | G | 0.2636 |
| 2017 | 21-Jul-17 | MONTEREY | BRUSSELS SPROUTS | Redacted4 | 9.91 | 18.8 | A | G | 0.2636 |
| 2017 | 23-Jul-17 | MONTEREY | BRUSSELS SPROUTS | Redacted4 | 4.955 | 9.4 | A | G | 0.2636 |
| 2017 | 24-Aug-17 | MONTEREY | BRUSSELS SPROUTS | Redacted4 | 9.91 | 18.8 | A | G | 0.2636 |
| 2017 | 10-Aug-17 | MONTEREY | BRUSSELS SPROUTS | Redacted4 | 4.955 | 9.4 | A | A | 0.2636 |
| 2017 | 9-Sep-17 | MONTEREY | BRUSSELS SPROUTS | Redacted4 | 9.91 | 18.8 | A | G | 0.2636 |
| 2017 | 6-Jul-17 | MONTEREY | BRUSSELS SPROUTS | Redacted4 | 9.91 | 18.8 | A | G | 0.2636 |
| 2017 | 24-Aug-17 | MONTEREY | BRUSSELS SPROUTS | Redacted4 | 2.2675 | 4.3 | A | G | 0.2637 |
| 2017 | 10-Sep-17 | MONTEREY | BRUSSELS SPROUTS | Redacted4 | 2.2675 | 4.3 | A | G | 0.2637 |
| 2017 | 20-Jul-17 | MONTEREY | BRUSSELS SPROUTS | Redacted4 | 2.2675 | 4.3 | A | G | 0.2637 |
| 2017 | 12-Jul-17 | MONTEREY | BRUSSELS SPROUTS | Redacted4 | 2.2675 | 4.3 | A | G | 0.2637 |
| 2017 | 23-Jun-17 | MONTEREY | BRUSSELS SPROUTS | Redacted4 | 2.2675 | 4.3 | A | G | 0.2637 |
| 2017 | 7-Jun-17 | MONTEREY | BRUSSELS SPROUTS | Redacted4 | 4.5351 | 8.6 | A | G | 0.2637 |
| 2017 | 7-Sep-17 | MONTEREY | BRUSSELS SPROUTS | Redacted4 | 4.5351 | 8.6 | A | G | 0.2637 |
| 2017 | 1-Jul-17 | MONTEREY | BRUSSELS SPROUTS | Redacted4 | 4.5351 | 8.6 | A | G | 0.2637 |
| 2017 | 19-Jul-17 | MONTEREY | BRUSSELS SPROUTS | Redacted4 | 4.5351 | 8.6 | A | G | 0.2637 |
| 2017 | 22-Aug-17 | MONTEREY | BRUSSELS SPROUTS | Redacted4 | 4.5351 | 8.6 | A | G | 0.2637 |
| 2017 | 8-Sep-17 | MONTEREY | BRUSSELS SPROUTS | Redacted4 | 4.1152 | 7.8 | A | G | 0.2638 |
| 2017 | 22-Sep-17 | MONTEREY | BRUSSELS SPROUTS | Redacted4 | 4.1152 | 7.8 | A | G | 0.2638 |
| 2017 | 5-Jul-17 | MONTEREY | BRUSSELS SPROUTS | Redacted4 | 4.1152 | 7.8 | A | G | 0.2638 |
| 2017 | 6-Jun-17 | MONTEREY | BRUSSELS SPROUTS | Redacted4 | 4.1152 | 7.8 | A | G | 0.2638 |
| 2017 | 28-Jul-17 | MONTEREY | BRUSSELS SPROUTS | Redacted4 | 4.1152 | 7.8 | A | G | 0.2638 |
| 2017 | 28-Aug-17 | MONTEREY | BRUSSELS SPROUTS | Redacted4 | 7.3905 | 14 | A | G | 0.2639 |
| 2017 | 22-Jul-17 | MONTEREY | BRUSSELS SPROUTS | Redacted4 | 7.3905 | 14 | A | G | 0.2639 |
| 2017 | 19-Sep-17 | MONTEREY | BRUSSELS SPROUTS | Redacted4 | 7.3905 | 14 | A | G | 0.2639 |
| 2017 | 25-Jun-17 | MONTEREY | BRUSSELS SPROUTS | Redacted4 | 7.3905 | 14 | A | G | 0.2639 |
| 2017 | 13-Aug-17 | MONTEREY | BRUSSELS SPROUTS | Redacted4 | 7.3905 | 14 | A | G | 0.2639 |
| 2017 | 2-Aug-17 | MONTEREY | BRUSSELS SPROUTS | Redacted4 | 7.3905 | 14 | A | G | 0.2639 |
| 2017 | 3-Jun-17 | MONTEREY | BRUSSELS SPROUTS | Redacted4 | 7.3905 | 14 | A | G | 0.2639 |
| 2017 | 30-Jun-17 | SANTA CRUZ | BRUSSELS SPROUTS | Redacted4 | 7.3905 | 14 | A | G | 0.2639 |
| 2017 | 24-May-17 | SANTA CRUZ | BRUSSELS SPROUTS | Redacted4 | 7.3905 | 14 | A | G | 0.2639 |
| 2017 | 17-Jul-17 | SANTA CRUZ | BRUSSELS SPROUTS | Redacted4 | 7.3905 | 14 | A | G | 0.2639 |
| 2017 | 17-Jun-17 | MONTEREY | BRUSSELS SPROUTS | Redacted4 | 3.6953 | 7 | A | G | 0.2640 |
| 2017 | 30-Jul-17 | MONTEREY | BRUSSELS SPROUTS | Redacted4 | 3.6953 | 7 | A | G | 0.2640 |
| 2017 | 14-Jun-17 | MONTEREY | BRUSSELS SPROUTS | Redacted4 | 3.6953 | 7 | A | G | 0.2640 |
| 2017 | 14-Jul-17 | MONTEREY | BRUSSELS SPROUTS | Redacted4 | 3.6953 | 7 | A | G | 0.2640 |
| 2017 | 25-Jul-17 | MONTEREY | BRUSSELS SPROUTS | Redacted4 | 3.6953 | 7 | A | G | 0.2640 |
| 2017 | 21-Jul-17 | MONTEREY | BRUSSELS SPROUTS | Redacted4 | 3.6953 | 7 | A | G | 0.2640 |
| 2017 | 30-Jul-17 | MONTEREY | BRUSSELS SPROUTS | Redacted4 | 3.6953 | 7 | A | G | 0.2640 |
| 2017 | 12-Sep-17 | MONTEREY | BRUSSELS SPROUTS | Redacted4 | 3.6953 | 7 | A | G | 0.2640 |
| 2017 | 13-Aug-17 | MONTEREY | BRUSSELS SPROUTS | Redacted4 | 3.6953 | 7 | A | G | 0.2640 |
| 2017 | 7-Aug-17 | MONTEREY | BRUSSELS SPROUTS | Redacted4 | 3.6953 | 7 | A | G | 0.2640 |
| 2017 | 21-Aug-17 | MONTEREY | BRUSSELS SPROUTS | Redacted4 | 3.6953 | 7 | A | G | 0.2640 |
| 2017 | 25-May-17 | MONTEREY | BRUSSELS SPROUTS | Redacted4 | 3.6953 | 7 | A | G | 0.2640 |
| 2017 | 17-Jun-17 | MONTEREY | BRUSSELS SPROUTS | Redacted4 | 3.6953 | 7 | A | G | 0.2640 |
| 2017 | 29-Jun-17 | MONTEREY | BRUSSELS SPROUTS | Redacted4 | 3.6953 | 7 | A | G | 0.2640 |
| 2017 | 25-May-17 | MONTEREY | BRUSSELS SPROUTS | Redacted4 | 3.6953 | 7 | A | G | 0.2640 |
| 2017 | 17-Jul-17 | MONTEREY | BRUSSELS SPROUTS | Redacted4 | 3.6953 | 7 | A | G | 0.2640 |
| 2017 | 7-Sep-17 | MONTEREY | BRUSSELS SPROUTS | Redacted4 | 3.6953 | 7 | A | G | 0.2640 |
| 2017 | 27-May-17 | MONTEREY | BRUSSELS SPROUTS | Redacted4 | 3.6953 | 7 | A | G | 0.2640 |
| 2017 | 14-Jul-17 | MONTEREY | BRUSSELS SPROUTS | Redacted4 | 3.6953 | 7 | A | G | 0.2640 |
| 2017 | 20-Sep-17 | MONTEREY | BRUSSELS SPROUTS | Redacted4 | 3.6953 | 7 | A | G | 0.2640 |
| 2017 | 4-Jul-17 | MONTEREY | BRUSSELS SPROUTS | Redacted4 | 3.6953 | 7 | A | G | 0.2640 |
| 2017 | 22-Aug-17 | MONTEREY | BRUSSELS SPROUTS | Redacted4 | 6.9706 | 13.2 | A | G | 0.2640 |
| 2017 | 7-Sep-17 | MONTEREY | BRUSSELS SPROUTS | Redacted4 | 6.9706 | 13.2 | A | G | 0.2640 |
| 2017 | 23-May-17 | MONTEREY | BRUSSELS SPROUTS | Redacted4 | 6.9706 | 13.2 | A | G | 0.2640 |
| 2017 | 7-Jun-17 | MONTEREY | BRUSSELS SPROUTS | Redacted4 | 6.9706 | 13.2 | A | G | 0.2640 |
| 2017 | 29-Jun-17 | MONTEREY | BRUSSELS SPROUTS | Redacted4 | 6.9706 | 13.2 | A | G | 0.2640 |
| 2017 | 18-Jul-17 | MONTEREY | BRUSSELS SPROUTS | Redacted4 | 6.9706 | 13.2 | A | G | 0.2640 |
| 2017 | 23-May-17 | MONTEREY | BRUSSELS SPROUTS | Redacted4 | 5.123 | 9.7 | A | G | 0.2641 |
| 2017 | 10-Jul-17 | MONTEREY | BRUSSELS SPROUTS | Redacted4 | 5.123 | 9.7 | A | G | 0.2641 |
| 2017 | 22-Aug-17 | MONTEREY | BRUSSELS SPROUTS | Redacted4 | 5.123 | 9.7 | A | G | 0.2641 |
| 2017 | 7-Sep-17 | MONTEREY | BRUSSELS SPROUTS | Redacted4 | 5.123 | 9.7 | A | G | 0.2641 |
| 2017 | 21-Jun-17 | MONTEREY | BRUSSELS SPROUTS | Redacted4 | 5.123 | 9.7 | A | G | 0.2641 |
| 2017 | 20-Jul-17 | MONTEREY | BRUSSELS SPROUTS | Redacted4 | 5.123 | 9.7 | A | G | 0.2641 |
| 2017 | 24-Aug-17 | MONTEREY | BRUSSELS SPROUTS | Redacted4 | 3.2753 | 6.2 | A | G | 0.2641 |
| 2017 | 18-Jul-17 | MONTEREY | BRUSSELS SPROUTS | Redacted4 | 3.2753 | 6.2 | A | G | 0.2641 |
| 2017 | 22-Jun-17 | MONTEREY | BRUSSELS SPROUTS | Redacted4 | 3.2753 | 6.2 | A | G | 0.2641 |
| 2017 | 18-Jul-17 | MONTEREY | BRUSSELS SPROUTS | Redacted4 | 3.2753 | 6.2 | A | G | 0.2641 |
| 2017 | 22-Jun-17 | MONTEREY | BRUSSELS SPROUTS | Redacted4 | 3.2753 | 6.2 | A | G | 0.2641 |
| 2017 | 20-Jul-17 | MONTEREY | BRUSSELS SPROUTS | Redacted4 | 6.5507 | 12.4 | A | G | 0.2641 |
| 2017 | 4-Aug-17 | MONTEREY | BRUSSELS SPROUTS | Redacted4 | 6.5507 | 12.4 | A | G | 0.2641 |
| 2017 | 6-Sep-17 | MONTEREY | BRUSSELS SPROUTS | Redacted4 | 6.5507 | 12.4 | A | G | 0.2641 |
| 2017 | 2-Nov-17 | MONTEREY | BRUSSELS SPROUTS | Redacted4 | 6.5507 | 12.4 | A | G | 0.2641 |
| 2017 | 18-Aug-17 | MONTEREY | BRUSSELS SPROUTS | Redacted4 | 6.5507 | 12.4 | A | G | 0.2641 |
| 2017 | 3-Nov-17 | MONTEREY | BRUSSELS SPROUTS | Redacted4 | 4.7031 | 8.9 | A | G | 0.2642 |
| 2017 | 6-Sep-17 | MONTEREY | BRUSSELS SPROUTS | Redacted4 | 4.7031 | 8.9 | A | G | 0.2642 |
| 2017 | 25-Jul-17 | MONTEREY | BRUSSELS SPROUTS | Redacted4 | 4.7031 | 8.9 | A | G | 0.2642 |
| 2017 | 19-Jul-17 | MONTEREY | BRUSSELS SPROUTS | Redacted4 | 4.7031 | 8.9 | A | G | 0.2642 |
| 2017 | 10-Aug-17 | MONTEREY | BRUSSELS SPROUTS | Redacted4 | 4.7031 | 8.9 | A | A | 0.2642 |
| 2017 | 25-Sep-17 | MONTEREY | BRUSSELS SPROUTS | Redacted4 | 4.7031 | 8.9 | A | G | 0.2642 |
| 2017 | 1-Jun-17 | MONTEREY | BRUSSELS SPROUTS | Redacted4 | 4.7031 | 8.9 | A | G | 0.2642 |
| 2017 | 23-Jul-17 | MONTEREY | BRUSSELS SPROUTS | Redacted4 | 4.7031 | 8.9 | A | G | 0.2642 |
| 2017 | 5-Oct-17 | MONTEREY | BRUSSELS SPROUTS | Redacted4 | 4.7031 | 8.9 | A | G | 0.2642 |
| 2017 | 24-May-17 | MONTEREY | BRUSSELS SPROUTS | Redacted4 | 4.7031 | 8.9 | A | G | 0.2642 |
| 2017 | 24-Jun-17 | MONTEREY | BRUSSELS SPROUTS | Redacted4 | 4.7031 | 8.9 | A | G | 0.2642 |
| 2017 | 18-Aug-17 | MONTEREY | BRUSSELS SPROUTS | Redacted4 | 4.7031 | 8.9 | A | G | 0.2642 |
| 2017 | 11-Sep-17 | MONTEREY | BRUSSELS SPROUTS | Redacted4 | 4.7031 | 8.9 | A | G | 0.2642 |
| 2017 | 17-Jun-17 | MONTEREY | BRUSSELS SPROUTS | Redacted4 | 5.7108 | 10.8 | A | G | 0.2644 |
| 2017 | 24-Jul-17 | MONTEREY | BRUSSELS SPROUTS | Redacted4 | 5.7108 | 10.8 | A | G | 0.2644 |
| 2017 | 22-Jun-17 | MONTEREY | BRUSSELS SPROUTS | Redacted4 | 1.4277 | 2.7 | A | G | 0.2644 |
| 2017 | 14-Jul-17 | MONTEREY | BRUSSELS SPROUTS | Redacted4 | 5.7108 | 10.8 | A | G | 0.2644 |
| 2017 | 18-Jul-17 | MONTEREY | BRUSSELS SPROUTS | Redacted4 | 1.4277 | 2.7 | A | G | 0.2644 |
| 2017 | 19-Jul-17 | MONTEREY | BRUSSELS SPROUTS | Redacted4 | 5.7108 | 10.8 | A | G | 0.2644 |
| 2017 | 6-Sep-17 | MONTEREY | BRUSSELS SPROUTS | Redacted4 | 5.7108 | 10.8 | A | G | 0.2644 |
| 2017 | 26-Sep-17 | MONTEREY | BRUSSELS SPROUTS | Redacted4 | 5.7108 | 10.8 | A | G | 0.2644 |
| 2017 | 19-Aug-17 | MONTEREY | BRUSSELS SPROUTS | Redacted4 | 5.7108 | 10.8 | A | G | 0.2644 |
| 2017 | 10-Aug-17 | MONTEREY | BRUSSELS SPROUTS | Redacted4 | 1.4277 | 2.7 | A | A | 0.2644 |
| 2017 | 18-Jul-17 | MONTEREY | BRUSSELS SPROUTS | Redacted4 | 4.2831 | 8.1 | A | G | 0.2644 |
| 2017 | 22-Jun-17 | MONTEREY | BRUSSELS SPROUTS | Redacted4 | 4.2831 | 8.1 | A | G | 0.2644 |
| 2017 | 13-Jul-17 | MONTEREY | BRUSSELS SPROUTS | Redacted4 | 5.2909 | 10 | A | G | 0.2645 |
| 2017 | 12-Jul-17 | MONTEREY | BRUSSELS SPROUTS | Redacted4 | 5.2909 | 10 | A | G | 0.2645 |
| 2017 | 2-Nov-17 | MONTEREY | BRUSSELS SPROUTS | Redacted4 | 5.2909 | 10 | A | G | 0.2645 |
| 2017 | 10-Aug-17 | MONTEREY | BRUSSELS SPROUTS | Redacted4 | 5.2909 | 10 | A | G | 0.2645 |
| 2017 | 18-Aug-17 | MONTEREY | BRUSSELS SPROUTS | Redacted4 | 5.2909 | 10 | A | G | 0.2645 |
| 2017 | 16-Jun-17 | MONTEREY | BRUSSELS SPROUTS | Redacted4 | 5.2909 | 10 | A | G | 0.2645 |
| 2017 | 29-Jul-17 | MONTEREY | BRUSSELS SPROUTS | Redacted4 | 5.2909 | 10 | A | G | 0.2645 |
| 2017 | 6-Sep-17 | MONTEREY | BRUSSELS SPROUTS | Redacted4 | 5.2909 | 10 | A | G | 0.2645 |
| 2017 | 27-May-17 | MONTEREY | BRUSSELS SPROUTS | Redacted4 | 5.2909 | 10 | A | G | 0.2645 |
| 2017 | 26-Jul-17 | MONTEREY | BRUSSELS SPROUTS | Redacted4 | 5.2909 | 10 | A | G | 0.2645 |
| 2017 | 20-Jul-17 | MONTEREY | BRUSSELS SPROUTS | Redacted4 | 5.2909 | 10 | A | G | 0.2645 |
| 2017 | 2-Aug-17 | MONTEREY | BRUSSELS SPROUTS | Redacted4 | 5.2909 | 10 | A | G | 0.2645 |
| 2017 | 17-Sep-17 | MONTEREY | BRUSSELS SPROUTS | Redacted4 | 5.2909 | 10 | A | G | 0.2645 |
| 2017 | 6-Sep-17 | MONTEREY | BRUSSELS SPROUTS | Redacted4 | 5.2909 | 10 | A | G | 0.2645 |
| 2017 | 23-Sep-17 | MONTEREY | BRUSSELS SPROUTS | Redacted4 | 5.2909 | 10 | A | G | 0.2645 |
| 2017 | 4-Nov-17 | MONTEREY | BRUSSELS SPROUTS | Redacted4 | 5.2909 | 10 | A | G | 0.2645 |
| 2017 | 19-Jul-17 | MONTEREY | BRUSSELS SPROUTS | Redacted4 | 5.2909 | 10 | A | G | 0.2645 |
| 2017 | 2-Nov-17 | MONTEREY | BRUSSELS SPROUTS | Redacted4 | 5.2909 | 10 | A | G | 0.2645 |
| 2017 | 3-Aug-17 | MONTEREY | BRUSSELS SPROUTS | Redacted4 | 5.2909 | 10 | A | G | 0.2645 |
| 2017 | 19-Aug-17 | MONTEREY | BRUSSELS SPROUTS | Redacted4 | 5.2909 | 10 | A | G | 0.2645 |
| 2017 | 21-Aug-17 | MONTEREY | BRUSSELS SPROUTS | Redacted4 | 5.2909 | 10 | A | G | 0.2645 |
| 2017 | 18-Aug-17 | MONTEREY | BRUSSELS SPROUTS | Redacted4 | 5.2909 | 10 | A | G | 0.2645 |
| 2017 | 6-Sep-17 | MONTEREY | BRUSSELS SPROUTS | Redacted4 | 5.2909 | 10 | A | G | 0.2645 |
| 2017 | 2-Aug-17 | SANTA CRUZ | BRUSSELS SPROUTS | Redacted4 | 5.2909 | 10 | A | G | 0.2645 |
| 2017 | 19-Aug-17 | SANTA CRUZ | BRUSSELS SPROUTS | Redacted4 | 5.2909 | 10 | A | G | 0.2645 |
| 2017 | 10-Jul-17 | SANTA CRUZ | BRUSSELS SPROUTS | Redacted4 | 5.2909 | 10 | A | G | 0.2645 |
| 2017 | 17-Jun-17 | SANTA CRUZ | BRUSSELS SPROUTS | Redacted4 | 5.2909 | 10 | A | G | 0.2645 |
| 2017 | 27-Jul-17 | MONTEREY | BRUSSELS SPROUTS | Redacted4 | 3.4433 | 6.5 | A | G | 0.2649 |
| 2017 | 15-Jul-17 | MONTEREY | BRUSSELS SPROUTS | Redacted4 | 3.4433 | 6.5 | A | G | 0.2649 |
| 2017 | 3-Jun-17 | MONTEREY | BRUSSELS SPROUTS | Redacted4 | 3.4433 | 6.5 | A | G | 0.2649 |
| 2017 | 18-Aug-17 | MONTEREY | BRUSSELS SPROUTS | Redacted4 | 3.4433 | 6.5 | A | G | 0.2649 |
| 2017 | 6-Oct-17 | MONTEREY | BRUSSELS SPROUTS | Redacted4 | 3.4433 | 6.5 | A | G | 0.2649 |
| 2017 | 13-Jun-17 | MONTEREY | BRUSSELS SPROUTS | Redacted4 | 3.4433 | 6.5 | A | G | 0.2649 |
| 2017 | 21-Jun-17 | MONTEREY | BRUSSELS SPROUTS | Redacted4 | 4.4511 | 8.4 | A | G | 0.2649 |
| 2017 | 24-May-17 | MONTEREY | BRUSSELS SPROUTS | Redacted4 | 4.4511 | 8.4 | A | G | 0.2649 |
| 2017 | 11-Jul-17 | MONTEREY | BRUSSELS SPROUTS | Redacted4 | 4.4511 | 8.4 | A | G | 0.2649 |
| 2017 | 25-Jul-17 | MONTEREY | BRUSSELS SPROUTS | Redacted4 | 4.4511 | 8.4 | A | G | 0.2649 |
| 2017 | 4-Jul-17 | MONTEREY | BRUSSELS SPROUTS | Redacted4 | 3.1914 | 6 | A | G | 0.2660 |
| 2017 | 17-May-17 | MONTEREY | BRUSSELS SPROUTS | Redacted4 | 3.1914 | 6 | A | G | 0.2660 |
| 2017 | 17-Jul-17 | MONTEREY | BRUSSELS SPROUTS | Redacted4 | 3.1914 | 6 | A | G | 0.2660 |
| 2017 | 27-Jul-17 | MONTEREY | BRUSSELS SPROUTS | Redacted4 | 2.7714 | 5.2 | A | G | 0.2665 |
| 2017 | 13-Jun-17 | MONTEREY | BRUSSELS SPROUTS | Redacted4 | 2.7714 | 5.2 | A | G | 0.2665 |
| 2017 | 18-Aug-17 | MONTEREY | BRUSSELS SPROUTS | Redacted4 | 2.7714 | 5.2 | A | G | 0.2665 |
| 2017 | 3-Jun-17 | MONTEREY | BRUSSELS SPROUTS | Redacted4 | 2.7714 | 5.2 | A | G | 0.2665 |
| 2017 | 15-Jul-17 | MONTEREY | BRUSSELS SPROUTS | Redacted4 | 2.7714 | 5.2 | A | G | 0.2665 |
| 2017 | 10-Aug-17 | MONTEREY | BRUSSELS SPROUTS | Redacted4 | 2.7714 | 5.2 | A | A | 0.2665 |
| 2017 | 6-Oct-17 | MONTEREY | BRUSSELS SPROUTS | Redacted4 | 2.7714 | 5.2 | A | G | 0.2665 |
| 2017 | 19-Jul-17 | MONTEREY | BRUSSELS SPROUTS | Redacted4 | 1.7636 | 3.3 | A | G | 0.2672 |
| 2017 | 24-Aug-17 | MONTEREY | BRUSSELS SPROUTS | Redacted4 | 1.7636 | 3.3 | A | G | 0.2672 |
| 2017 | 8-Sep-17 | MONTEREY | BRUSSELS SPROUTS | Redacted4 | 1.7636 | 3.3 | A | G | 0.2672 |
| 2017 | 3-Jul-17 | MONTEREY | BRUSSELS SPROUTS | Redacted4 | 1.7636 | 3.3 | A | G | 0.2672 |
| 2017 | 10-Aug-17 | MONTEREY | BRUSSELS SPROUTS | Redacted4 | 1.7636 | 3.3 | A | A | 0.2672 |
| 2017 | 31-Oct-17 | MONTEREY | BRUSSELS SPROUTS | Redacted4 | 1.7636 | 3.3 | A | G | 0.2672 |
| 2017 | 24-Sep-17 | MONTEREY | BRUSSELS SPROUTS | Redacted4 | 1.5117 | 2.8 | A | A | 0.2699 |
| 2017 | 24-Aug-17 | MONTEREY | BRUSSELS SPROUTS | Redacted4 | 1.5117 | 2.8 | A | G | 0.2699 |
| 2017 | 10-Aug-17 | MONTEREY | BRUSSELS SPROUTS | Redacted4 | 1.5117 | 2.8 | A | A | 0.2699 |
| 2017 | 18-Jul-17 | MONTEREY | BRUSSELS SPROUTS | Redacted4 | 1.5117 | 2.8 | A | G | 0.2699 |
| 2017 | 22-Jun-17 | MONTEREY | BRUSSELS SPROUTS | Redacted4 | 1.5117 | 2.8 | A | G | 0.2699 |
| 2017 | 14-Sep-17 | VENTURA | BRUSSELS SPROUTS | Redacted4 | 48.6262 | 78 | A | G | 0.3117 |
| 2017 | 14-Jun-17 | VENTURA | BRUSSELS SPROUTS | Redacted4 | 39.808 | 63.83 | A | G | 0.3118 |
| 2017 | 18-Jul-17 | VENTURA | BRUSSELS SPROUTS | Redacted4 | 39.808 | 63.83 | A | G | 0.3118 |
| 2017 | 27-Oct-17 | KERN | CABBAGE | Redacted4 | 8.3983 | 40 | A | A | 0.1050 |
| 2017 | 22-Sep-17 | KERN | CABBAGE | Redacted4 | 8.2303 | 36 | A | A | 0.1143 |
| 2017 | 22-May-17 | VENTURA | CABBAGE | Redacted4 | 3.1074 | 12 | A | G | 0.1295 |
| 2017 | 31-May-17 | VENTURA | CABBAGE | Redacted4 | 3.2753 | 12.6 | A | G | 0.1300 |
| 2017 | 19-May-17 | VENTURA | CABBAGE | Redacted4 | 3.2753 | 12.6 | A | G | 0.1300 |
| 2017 | 7-Jun-17 | VENTURA | CABBAGE | Redacted4 | 3.2753 | 12.6 | A | G | 0.1300 |
| 2017 | 15-Jun-17 | VENTURA | CABBAGE | Redacted4 | 3.2753 | 12.6 | A | G | 0.1300 |
| 2017 | 12-Jun-17 | VENTURA | CABBAGE | Redacted4 | 2.6035 | 10 | A | G | 0.1302 |
| 2017 | 18-Oct-17 | VENTURA | CABBAGE | Redacted4 | 5.7108 | 21.9 | A | G | 0.1304 |
| 2017 | 12-Jun-17 | VENTURA | CABBAGE | Redacted4 | 2.6875 | 10.3 | A | G | 0.1305 |
| 2017 | 12-Jun-17 | VENTURA | CABBAGE | Redacted4 | 5.2909 | 20.25 | A | G | 0.1306 |
| 2017 | 19-May-17 | VENTURA | CABBAGE | Redacted4 | 5.2909 | 20.25 | A | G | 0.1306 |
| 2017 | 12-Jul-17 | VENTURA | CABBAGE | Redacted4 | 4.7031 | 18 | A | G | 0.1306 |
| 2017 | 8-Jul-17 | VENTURA | CABBAGE | Redacted4 | 6.2987 | 24.1 | A | G | 0.1307 |
| 2017 | 28-Aug-17 | VENTURA | CABBAGE | Redacted4 | 3.1914 | 12.2 | A | G | 0.1308 |
| 2017 | 4-Oct-17 | KERN | CABBAGE | Redacted4 | 8.9022 | 34 | A | A | 0.1309 |
| 2017 | 13-Jun-17 | VENTURA | CABBAGE | Redacted4 | 7.7264 | 29.5 | A | G | 0.1310 |
| 2017 | 3-Jun-17 | VENTURA | CABBAGE | Redacted4 | 7.7264 | 29.5 | A | G | 0.1310 |
| 2017 | 21-Jun-17 | VENTURA | CABBAGE | Redacted4 | 7.7264 | 29.5 | A | G | 0.1310 |
| 2017 | 3-Jul-17 | VENTURA | CABBAGE | Redacted4 | 7.7264 | 29.5 | A | G | 0.1310 |
| 2017 | 3-Jul-17 | VENTURA | CABBAGE | Redacted4 | 6.2987 | 24 | A | G | 0.1312 |
| 2017 | 13-Sep-17 | VENTURA | CABBAGE | Redacted4 | 3.4433 | 13.1 | A | G | 0.1314 |
| 2017 | 23-Nov-17 | VENTURA | CABBAGE | Redacted4 | 1.5117 | 5.75 | A | G | 0.1315 |
| 2017 | 12-Jun-17 | VENTURA | CABBAGE | Redacted4 | 4.871 | 18.5 | A | G | 0.1316 |
| 2017 | 14-Oct-17 | KERN | CABBAGE | Redacted4 | 9.4901 | 36 | A | A | 0.1318 |
| 2017 | 26-Jun-17 | VENTURA | CABBAGE | Redacted4 | 2.8554 | 10.8 | A | G | 0.1322 |
| 2017 | 15-Jun-17 | VENTURA | CABBAGE | Redacted4 | 3.1914 | 12 | A | G | 0.1330 |
| 2017 | 1-Jul-17 | VENTURA | CABBAGE | Redacted4 | 3.1914 | 12 | A | G | 0.1330 |
| 2017 | 28-Oct-17 | VENTURA | CABBAGE | Redacted4 | 1.0918 | 4 | A | G | 0.1365 |
| 2017 | 22-Nov-17 | VENTURA | CABBAGE | Redacted4 | 1.0918 | 4 | A | G | 0.1365 |
| 2017 | 28-Sep-17 | VENTURA | CABBAGE | Redacted4 | 1.2597 | 4 | A | G | 0.1575 |
| 2017 | 22-Aug-17 | VENTURA | CABBAGE | Redacted4 | 6.2987 | 19.4 | A | G | 0.1623 |
| 2017 | 20-Sep-17 | VENTURA | CABBAGE | Redacted4 | 4.787 | 14.7 | A | G | 0.1628 |
| 2017 | 14-Sep-17 | VENTURA | CABBAGE | Redacted4 | 4.955 | 15.2 | A | G | 0.1630 |
| 2017 | 22-May-17 | VENTURA | CABBAGE | Redacted4 | 2.7714 | 8.5 | A | G | 0.1630 |
| 2017 | 25-Nov-17 | VENTURA | CABBAGE | Redacted4 | 5.5429 | 17 | A | G | 0.1630 |
| 2017 | 18-Jul-17 | VENTURA | CABBAGE | Redacted4 | 5.8788 | 18 | A | G | 0.1633 |
| 2017 | 21-Jul-17 | VENTURA | CABBAGE | Redacted4 | 2.9394 | 9 | A | G | 0.1633 |
| 2017 | 26-Jul-17 | VENTURA | CABBAGE | Redacted4 | 5.8788 | 18 | A | G | 0.1633 |
| 2017 | 17-Jul-17 | VENTURA | CABBAGE | Redacted4 | 2.9394 | 9 | A | G | 0.1633 |
| 2017 | 12-Jul-17 | VENTURA | CABBAGE | Redacted4 | 3.5273 | 10.8 | A | G | 0.1633 |
| 2017 | 3-Jul-17 | VENTURA | CABBAGE | Redacted4 | 3.5273 | 10.8 | A | G | 0.1633 |
| 2017 | 8-Jul-17 | VENTURA | CABBAGE | Redacted4 | 3.5273 | 10.8 | A | G | 0.1633 |
| 2017 | 23-Jun-17 | VENTURA | CABBAGE | Redacted4 | 4.1152 | 12.6 | A | G | 0.1633 |
| 2017 | 7-Jun-17 | VENTURA | CABBAGE | Redacted4 | 4.1152 | 12.6 | A | G | 0.1633 |
| 2017 | 26-Oct-17 | VENTURA | CABBAGE | Redacted4 | 9.4901 | 29.05 | A | G | 0.1633 |
| 2017 | 3-Sep-17 | VENTURA | CABBAGE | Redacted4 | 6.6347 | 20.3 | A | G | 0.1634 |
| 2017 | 21-Sep-17 | VENTURA | CABBAGE | Redacted4 | 6.6347 | 20.3 | A | G | 0.1634 |
| 2017 | 9-Sep-17 | VENTURA | CABBAGE | Redacted4 | 6.6347 | 20.3 | A | G | 0.1634 |
| 2017 | 9-Sep-17 | VENTURA | CABBAGE | Redacted4 | 6.0468 | 18.5 | A | G | 0.1634 |
| 2017 | 19-May-17 | VENTURA | CABBAGE | Redacted4 | 6.0468 | 18.5 | A | G | 0.1634 |
| 2017 | 3-Sep-17 | VENTURA | CABBAGE | Redacted4 | 6.0468 | 18.5 | A | G | 0.1634 |
| 2017 | 22-Aug-17 | VENTURA | CABBAGE | Redacted4 | 6.0468 | 18.5 | A | G | 0.1634 |
| 2017 | 30-May-17 | VENTURA | CABBAGE | Redacted4 | 6.0468 | 18.5 | A | G | 0.1634 |
| 2017 | 20-Sep-17 | VENTURA | CABBAGE | Redacted4 | 4.2831 | 13.1 | A | G | 0.1635 |
| 2017 | 17-Jun-17 | VENTURA | CABBAGE | Redacted4 | 6.2147 | 19 | A | G | 0.1635 |
| 2017 | 7-Jun-17 | VENTURA | CABBAGE | Redacted4 | 6.2147 | 19 | A | G | 0.1635 |
| 2017 | 23-Jun-17 | VENTURA | CABBAGE | Redacted4 | 6.2147 | 19 | A | G | 0.1635 |
| 2017 | 16-May-17 | VENTURA | CABBAGE | Redacted4 | 8.7342 | 26.7 | A | G | 0.1636 |
| 2017 | 28-Nov-17 | VENTURA | CABBAGE | Redacted4 | 9.5741 | 29.25 | A | G | 0.1637 |
| 2017 | 16-Oct-17 | VENTURA | CABBAGE | Redacted4 | 3.1914 | 9.75 | A | G | 0.1637 |
| 2017 | 17-Jul-17 | VENTURA | CABBAGE | Redacted4 | 9.6581 | 29.5 | A | G | 0.1637 |
| 2017 | 19-May-17 | VENTURA | CABBAGE | Redacted4 | 3.2753 | 10 | A | G | 0.1638 |
| 2017 | 13-Dec-17 | VENTURA | CABBAGE | Redacted4 | 1.3437 | 4.1 | A | G | 0.1639 |
| 2017 | 14-Dec-17 | VENTURA | CABBAGE | Redacted4 | 8.4823 | 25.85 | A | G | 0.1641 |
| 2017 | 22-May-17 | VENTURA | CABBAGE | Redacted4 | 7.8104 | 23.8 | A | G | 0.1641 |
| 2017 | 3-Nov-17 | VENTURA | CABBAGE | Redacted4 | 4.5351 | 13.8 | A | G | 0.1643 |
| 2017 | 30-Oct-17 | VENTURA | CABBAGE | Redacted4 | 5.4589 | 16.6 | A | G | 0.1644 |
| 2017 | 12-Jul-17 | VENTURA | CABBAGE | Redacted4 | 7.8944 | 24 | A | G | 0.1645 |
| 2017 | 3-Jul-17 | VENTURA | CABBAGE | Redacted4 | 3.9472 | 12 | A | G | 0.1645 |
| 2017 | 22-Jun-17 | VENTURA | CABBAGE | Redacted4 | 3.9472 | 12 | A | G | 0.1645 |
| 2017 | 29-Jun-17 | VENTURA | CABBAGE | Redacted4 | 3.9472 | 12 | A | G | 0.1645 |
| 2017 | 14-Jul-17 | VENTURA | CABBAGE | Redacted4 | 3.9472 | 12 | A | G | 0.1645 |
| 2017 | 8-Jul-17 | VENTURA | CABBAGE | Redacted4 | 3.9472 | 12 | A | G | 0.1645 |
| 2017 | 16-Oct-17 | VENTURA | CABBAGE | Redacted4 | 4.871 | 14.8 | A | G | 0.1646 |
| 2017 | 22-May-17 | VENTURA | CABBAGE | Redacted4 | 5.7108 | 17.35 | A | G | 0.1646 |
| 2017 | 31-May-17 | VENTURA | CABBAGE | Redacted4 | 5.9628 | 18.1 | A | G | 0.1647 |
| 2017 | 19-Oct-17 | VENTURA | CABBAGE | Redacted4 | 2.6875 | 8.15 | A | G | 0.1649 |
| 2017 | 13-Dec-17 | VENTURA | CABBAGE | Redacted4 | 4.5351 | 13.75 | A | G | 0.1649 |
| 2017 | 28-Sep-17 | VENTURA | CABBAGE | Redacted4 | 3.6953 | 11.2 | A | G | 0.1650 |
| 2017 | 14-Oct-17 | VENTURA | CABBAGE | Redacted4 | 4.7031 | 14.25 | A | G | 0.1650 |
| 2017 | 19-May-17 | VENTURA | CABBAGE | Redacted4 | 4.787 | 14.5 | A | G | 0.1651 |
| 2017 | 17-Jul-17 | VENTURA | CABBAGE | Redacted4 | 4.787 | 14.5 | A | G | 0.1651 |
| 2017 | 14-Oct-17 | VENTURA | CABBAGE | Redacted4 | 4.955 | 15 | A | G | 0.1652 |
| 2017 | 3-Sep-17 | VENTURA | CABBAGE | Redacted4 | 4.0312 | 12.2 | A | G | 0.1652 |
| 2017 | 9-Sep-17 | VENTURA | CABBAGE | Redacted4 | 4.0312 | 12.2 | A | G | 0.1652 |
| 2017 | 16-May-17 | VENTURA | CABBAGE | Redacted4 | 3.3593 | 10.15 | A | G | 0.1655 |
| 2017 | 7-Jun-17 | VENTURA | CABBAGE | Redacted4 | 3.3593 | 10.15 | A | G | 0.1655 |
| 2017 | 13-Sep-17 | VENTURA | CABBAGE | Redacted4 | 1.5117 | 4.5 | A | G | 0.1680 |
| 2017 | 21-Dec-17 | VENTURA | CABBAGE | Redacted4 | 0.4199 | 1.1 | A | G | 0.1909 |
| 2017 | 28-Sep-17 | VENTURA | CABBAGE | Redacted4 | 2.5195 | 6.5 | A | G | 0.1938 |
| 2017 | 9-Dec-17 | VENTURA | CABBAGE | Redacted4 | 1.5957 | 4.1 | A | G | 0.1946 |
| 2017 | 27-Sep-17 | VENTURA | CABBAGE | Redacted4 | 5.123 | 13.1 | A | G | 0.1955 |
| 2017 | 14-Oct-17 | VENTURA | CABBAGE | Redacted4 | 5.123 | 13.1 | A | G | 0.1955 |
| 2017 | 2-Nov-17 | VENTURA | CABBAGE | Redacted4 | 5.7948 | 14.8 | A | G | 0.1958 |
| 2017 | 6-Nov-17 | VENTURA | CABBAGE | Redacted4 | 3.1914 | 8.15 | A | G | 0.1958 |
| 2017 | 9-Nov-17 | VENTURA | CABBAGE | Redacted4 | 3.1914 | 8.15 | A | G | 0.1958 |
| 2017 | 23-Nov-17 | VENTURA | CABBAGE | Redacted4 | 3.1914 | 8.15 | A | G | 0.1958 |
| 2017 | 26-Sep-17 | VENTURA | CABBAGE | Redacted4 | 1.7636 | 4.5 | A | G | 0.1960 |
| 2017 | 27-Dec-17 | VENTURA | CABBAGE | Redacted4 | 1.7636 | 4.5 | A | G | 0.1960 |
| 2017 | 9-Dec-17 | VENTURA | CABBAGE | Redacted4 | 5.2909 | 13.5 | A | G | 0.1960 |
| 2017 | 2-Aug-17 | VENTURA | CABBAGE | Redacted4 | 3.5273 | 9 | A | G | 0.1960 |
| 2017 | 23-Dec-17 | VENTURA | CABBAGE | Redacted4 | 4.1152 | 10.5 | A | G | 0.1960 |
| 2017 | 13-Dec-17 | VENTURA | CABBAGE | Redacted4 | 7.7264 | 19.7 | A | G | 0.1961 |
| 2017 | 14-Oct-17 | VENTURA | CABBAGE | Redacted4 | 5.9628 | 15.2 | A | G | 0.1961 |
| 2017 | 26-Oct-17 | VENTURA | CABBAGE | Redacted4 | 8.7342 | 22.25 | A | G | 0.1963 |
| 2017 | 7-Nov-17 | VENTURA | CABBAGE | Redacted4 | 11.5057 | 29.3 | A | G | 0.1963 |
| 2017 | 1-Dec-17 | VENTURA | CABBAGE | Redacted4 | 15.033 | 38.25 | A | G | 0.1965 |
| 2017 | 11-Dec-17 | VENTURA | CABBAGE | Redacted4 | 15.033 | 38.25 | A | G | 0.1965 |
| 2017 | 22-Dec-17 | VENTURA | CABBAGE | Redacted4 | 15.033 | 38.25 | A | G | 0.1965 |
| 2017 | 11-Dec-17 | VENTURA | CABBAGE | Redacted4 | 11.5057 | 29.25 | A | G | 0.1967 |
| 2017 | 20-Nov-17 | VENTURA | CABBAGE | Redacted4 | 11.5057 | 29.25 | A | G | 0.1967 |
| 2017 | 7-Nov-17 | VENTURA | CABBAGE | Redacted4 | 11.5057 | 29.2 | A | G | 0.1970 |
| 2017 | 28-Dec-17 | VENTURA | CABBAGE | Redacted4 | 3.8632 | 9.8 | A | G | 0.1971 |
| 2017 | 11-Dec-17 | VENTURA | CABBAGE | Redacted4 | 3.8632 | 9.8 | A | G | 0.1971 |
| 2017 | 21-Dec-17 | VENTURA | CABBAGE | Redacted4 | 6.5507 | 16.6 | A | G | 0.1973 |
| 2017 | 2-Dec-17 | VENTURA | CABBAGE | Redacted4 | 6.5507 | 16.6 | A | G | 0.1973 |
| 2017 | 20-Dec-17 | VENTURA | CABBAGE | Redacted4 | 6.7186 | 17 | A | G | 0.1976 |
| 2017 | 28-Oct-17 | VENTURA | CABBAGE | Redacted4 | 9.4061 | 23.8 | A | G | 0.1976 |
| 2017 | 7-Nov-17 | VENTURA | CABBAGE | Redacted4 | 6.0468 | 15.3 | A | G | 0.1976 |
| 2017 | 26-Oct-17 | VENTURA | CABBAGE | Redacted4 | 9.4901 | 24 | A | G | 0.1977 |
| 2017 | 22-Dec-17 | VENTURA | CABBAGE | Redacted4 | 6.8026 | 17.2 | A | G | 0.1978 |
| 2017 | 20-Dec-17 | VENTURA | CABBAGE | Redacted4 | 5.4589 | 13.8 | A | G | 0.1978 |
| 2017 | 21-Dec-17 | VENTURA | CABBAGE | Redacted4 | 2.7714 | 7 | A | G | 0.1980 |
| 2017 | 13-Dec-17 | VENTURA | CABBAGE | Redacted4 | 5.5429 | 14 | A | G | 0.1980 |
| 2017 | 7-Nov-17 | VENTURA | CABBAGE | Redacted4 | 5.5429 | 14 | A | G | 0.1980 |
| 2017 | 7-Nov-17 | VENTURA | CABBAGE | Redacted4 | 5.5429 | 14 | A | G | 0.1980 |
| 2017 | 20-Dec-17 | VENTURA | CABBAGE | Redacted4 | 3.8632 | 9.75 | A | G | 0.1981 |
| 2017 | 22-Dec-17 | VENTURA | CABBAGE | Redacted4 | 5.039 | 12.7 | A | G | 0.1984 |
| 2017 | 26-Oct-17 | VENTURA | CABBAGE | Redacted4 | 1.5957 | 4 | A | G | 0.1995 |
| 2017 | 26-Oct-17 | VENTURA | CABBAGE | Redacted4 | 0.4199 | 1 | A | G | 0.2100 |
| 2017 | 31-Oct-17 | VENTURA | CABBAGE | Redacted4 | 2.6035 | 5 | A | G | 0.2604 |
| 2017 | 28-Oct-17 | VENTURA | CABBAGE | Redacted4 | 7.0546 | 13.5 | A | G | 0.2613 |
| 2017 | 31-Oct-17 | VENTURA | CABBAGE | Redacted4 | 2.0996 | 4 | A | G | 0.2625 |
| 2017 | 27-Nov-17 | VENTURA | CABBAGE | Redacted4 | 4.1992 | 8 | A | G | 0.2625 |
| 2017 | 14-Nov-17 | VENTURA | CABBAGE | Redacted4 | 7.0546 | 13.4 | A | G | 0.2632 |
| 2017 | 30-Oct-17 | VENTURA | CABBAGE | Redacted4 | 7.6425 | 14.5 | A | G | 0.2635 |
| 2017 | 1-Dec-17 | VENTURA | CABBAGE | Redacted4 | 9.0702 | 17.2 | A | G | 0.2637 |
| 2017 | 26-Oct-17 | VENTURA | CABBAGE | Redacted4 | 7.3905 | 14 | A | G | 0.2639 |
| 2017 | 6-Nov-17 | VENTURA | CABBAGE | Redacted4 | 5.5429 | 10.5 | A | G | 0.2639 |
| 2017 | 6-Nov-17 | VENTURA | CABBAGE | Redacted4 | 2.1836 | 4.1 | A | G | 0.2663 |
| 2017 | 1-Jun-17 | VENTURA | CABBAGE | Redacted4 | 1.1758 | 2 | A | G | 0.2940 |
| 2017 | 13-Jun-17 | VENTURA | CABBAGE | Redacted4 | 1.1758 | 2 | A | G | 0.2940 |
| 2017 | 18-Dec-17 | VENTURA | CABBAGE | Redacted4 | 7.4745 | 12.7 | A | G | 0.2943 |
| 2017 | 16-Dec-17 | VENTURA | CABBAGE | Redacted4 | 22.5914 | 38.25 | A | G | 0.2953 |
| 2017 | 13-Dec-17 | VENTURA | CABBAGE | Redacted4 | 10.162 | 17.2 | A | G | 0.2954 |
| 2017 | 7-Aug-17 | VENTURA | CABBAGE | Redacted4 | 7.1386 | 9 | A | G |  |
| 2017 | 5-Jul-17 | KERN | CARROTS, GENERAL | Redacted4 | 4.1992 | 16 | A | A | 0.1312 |
| 2017 | 18-May-17 | SANTA CLARA | CAULIFLOWER | Redacted4 | 1.1758 | 6 | A | G | 0.0980 |
| 2017 | 6-Jun-17 | SANTA BARBARA | CAULIFLOWER | Redacted4 | 1.9316 | 7.5 | A | G | 0.1288 |
| 2017 | 1-Jun-17 | SAN LUIS OBISPO | CAULIFLOWER | Redacted4 | 2.0156 | 7.8 | A | G | 0.1292 |
| 2017 | 12-Jul-17 | SANTA BARBARA | CAULIFLOWER | Redacted4 | 4.6191 | 17.7 | A | G | 0.1305 |
| 2017 | 29-Jun-17 | SAN LUIS OBISPO | CAULIFLOWER | Redacted4 | 3.2753 | 12.5 | A | G | 0.1310 |
| 2017 | 2-Jun-17 | SANTA BARBARA | CAULIFLOWER | Redacted4 | 1.076 | 4.1 | A | G | 0.1312 |
| 2017 | 7-Jul-17 | SANTA BARBARA | CAULIFLOWER | Redacted4 | 4.7765 | 18.2 | A | G | 0.1312 |
| 2017 | 12-Jun-17 | SANTA BARBARA | CAULIFLOWER | Redacted4 | 4.0679 | 15.5 | A | G | 0.1312 |
| 2017 | 29-Jun-17 | SANTA BARBARA | CAULIFLOWER | Redacted4 | 4.6689 | 17.79 | A | G | 0.1312 |
| 2017 | 5-Jun-17 | SANTA BARBARA | CAULIFLOWER | Redacted4 | 2.2308 | 8.5 | A | G | 0.1312 |
| 2017 | 5-Jun-17 | SANTA BARBARA | CAULIFLOWER | Redacted4 | 1.5642 | 5.96 | A | G | 0.1312 |
| 2017 | 13-Jun-17 | SANTA BARBARA | CAULIFLOWER | Redacted4 | 1.0498 | 4 | A | G | 0.1312 |
| 2017 | 9-Jun-17 | SAN LUIS OBISPO | CAULIFLOWER | Redacted4 | 1.4435 | 5.5 | A | G | 0.1312 |
| 2017 | 1-Jun-17 | SANTA BARBARA | CAULIFLOWER | Redacted4 | 3.3593 | 12.64 | A | G | 0.1329 |
| 2017 | 1-Jun-17 | SANTA BARBARA | CAULIFLOWER | Redacted4 | 1.4277 | 5.34 | A | G | 0.1337 |
| 2017 | 4-Aug-17 | SAN LUIS OBISPO | CAULIFLOWER | Redacted4 | 1.8476 | 5.7 | A | G | 0.1621 |
| 2017 | 5-Aug-17 | SAN LUIS OBISPO | CAULIFLOWER | Redacted4 | 2.6035 | 8 | A | G | 0.1627 |
| 2017 | 7-Jul-17 | SANTA BARBARA | CAULIFLOWER | Redacted4 | 2.6035 | 8 | A | G | 0.1627 |
| 2017 | 9-Jun-17 | SANTA BARBARA | CAULIFLOWER | Redacted4 | 3.9472 | 12.04 | A | G | 0.1639 |
| 2017 | 10-Jul-17 | SAN LUIS OBISPO | CAULIFLOWER | Redacted4 | 0.8201 | 2.5 | A | G | 0.1640 |
| 2017 | 26-Jun-17 | SANTA BARBARA | CAULIFLOWER | Redacted4 | 0.8201 | 2.5 | A | G | 0.1640 |
| 2017 | 23-May-17 | SANTA BARBARA | CAULIFLOWER | Redacted4 | 0.6889 | 2.1 | A | G | 0.1640 |
| 2017 | 19-Jul-17 | SAN LUIS OBISPO | CAULIFLOWER | Redacted4 | 2.4932 | 7.6 | A | G | 0.1640 |
| 2017 | 10-Jul-17 | SAN LUIS OBISPO | CAULIFLOWER | Redacted4 | 4.1007 | 12.5 | A | G | 0.1640 |
| 2017 | 18-Jul-17 | SANTA BARBARA | CAULIFLOWER | Redacted4 | 4.5928 | 14 | A | G | 0.1640 |
| 2017 | 20-Jul-17 | SAN LUIS OBISPO | CAULIFLOWER | Redacted4 | 1.7059 | 5.2 | A | G | 0.1640 |
| 2017 | 21-Jun-17 | SANTA BARBARA | CAULIFLOWER | Redacted4 | 5.9707 | 18.2 | A | G | 0.1640 |
| 2017 | 21-Jul-17 | SAN LUIS OBISPO | CAULIFLOWER | Redacted4 | 4.232 | 12.9 | A | G | 0.1640 |
| 2017 | 20-Jul-17 | SAN LUIS OBISPO | CAULIFLOWER | Redacted4 | 2.6245 | 8 | A | G | 0.1640 |
| 2017 | 28-Jul-17 | SAN LUIS OBISPO | CAULIFLOWER | Redacted4 | 2.6245 | 8 | A | G | 0.1640 |
| 2017 | 10-Jul-17 | SAN LUIS OBISPO | CAULIFLOWER | Redacted4 | 2.5917 | 7.9 | A | G | 0.1640 |
| 2017 | 26-May-17 | SAN LUIS OBISPO | CAULIFLOWER | Redacted4 | 3.0838 | 9.4 | A | G | 0.1640 |
| 2017 | 28-Jul-17 | SAN LUIS OBISPO | CAULIFLOWER | Redacted4 | 1.4763 | 4.5 | A | G | 0.1640 |
| 2017 | 28-Jul-17 | SAN LUIS OBISPO | CAULIFLOWER | Redacted4 | 1.4435 | 4.4 | A | G | 0.1640 |
| 2017 | 21-Jun-17 | SAN LUIS OBISPO | CAULIFLOWER | Redacted4 | 0.9514 | 2.9 | A | G | 0.1640 |
| 2017 | 20-Jun-17 | SAN LUIS OBISPO | CAULIFLOWER | Redacted4 | 0.3281 | 1 | A | G | 0.1641 |
| 2017 | 8-Aug-17 | SAN LUIS OBISPO | CAULIFLOWER | Redacted4 | 2.2675 | 6.9 | A | G | 0.1643 |
| 2017 | 14-Jul-17 | SAN LUIS OBISPO | CAULIFLOWER | Redacted4 | 2.4355 | 7.4 | A | G | 0.1646 |
| 2017 | 17-Jul-17 | SAN LUIS OBISPO | CAULIFLOWER | Redacted4 | 4.1152 | 12.5 | A | G | 0.1646 |
| 2017 | 5-Aug-17 | SAN LUIS OBISPO | CAULIFLOWER | Redacted4 | 3.2753 | 9.9 | A | G | 0.1654 |
| 2017 | 5-Jul-17 | VENTURA | CAULIFLOWER | Redacted4 | 1.6797 | 5 | A | G | 0.1680 |
| 2017 | 8-Jun-17 | TULARE | CHERRY | Redacted4 | 31.0737 | 95 | A | G | 0.1635 |
| 2017 | 17-Jun-17 | KERN | CHERRY | Redacted4 | 1.7636 | 5 | A | G | 0.1764 |
| 2017 | 12-Jun-17 | FRESNO | CHERRY | Redacted4 | 41.9915 | 40 | A | G |  |
| 2017 | 9-Jun-17 | FRESNO | CITRUS FRUITS (ALL OR UNSPEC) | Redacted4 | 2.6875 | 10 | A | G | 0.1344 |
| 2017 | 9-Jun-17 | FRESNO | CITRUS FRUITS (ALL OR UNSPEC) | Redacted4 | 2.6875 | 10 | A | G | 0.1344 |
| 2017 | 27-Jul-17 | KINGS | CORN (FORAGE - FODDER) | Redacted4 | 7.9784 | 101.55 | A | G | 0.0393 |
| 2017 | 27-Jul-17 | KINGS | CORN (FORAGE - FODDER) | Redacted4 | 7.9784 | 101.55 | A | G | 0.0393 |
| 2017 | 12-Jul-17 | KINGS | CORN (FORAGE - FODDER) | Redacted4 | 9.994 | 101.55 | A | G | 0.0492 |
| 2017 | 12-Jul-17 | KINGS | CORN (FORAGE - FODDER) | Redacted4 | 15.3689 | 155.6 | A | G | 0.0494 |
| 2017 | 12-Jul-17 | KINGS | CORN (FORAGE - FODDER) | Redacted4 | 15.3689 | 155.6 | A | G | 0.0494 |
| 2017 | 19-Jul-17 | KINGS | CORN (FORAGE - FODDER) | Redacted4 | 15.3689 | 155.6 | A | G | 0.0494 |
| 2017 | 19-Jun-17 | FRESNO | CORN, FIELD, DENT (GRAIN CROP) | Redacted4 | 12.4295 | 38 | A | G | 0.1635 |
| 2017 | 31-Oct-17 | FRESNO | CORN, HUMAN CONSUMPTION | Redacted4 | 4.787 | 73.43 | A | G | 0.0326 |
| 2017 | 5-Aug-17 | SAN JOAQUIN | CORN, HUMAN CONSUMPTION | Redacted4 | 6.2987 | 33 | A | G | 0.0954 |
| 2017 | 23-Jun-17 | FRESNO | CORN, HUMAN CONSUMPTION | Redacted4 | 18.3083 | 56 | A | G | 0.1635 |
| 2017 | 15-Jun-17 | FRESNO | CORN, HUMAN CONSUMPTION | Redacted4 | 9.1542 | 28 | A | G | 0.1635 |
| 2017 | 18-Jun-17 | FRESNO | CORN, HUMAN CONSUMPTION | Redacted4 | 12.4295 | 38 | A | G | 0.1635 |
| 2017 | 3-Jul-17 | FRESNO | CORN, HUMAN CONSUMPTION | Redacted4 | 31.1577 | 95 | A | G | 0.1640 |
| 2017 | 22-Jun-17 | FRESNO | GRAPES | Redacted4 | 0.3937 | 20 | A | G | 0.0098 |
| 2017 | 9-Jun-17 | FRESNO | GRAPES | Redacted4 | 0.3937 | 20 | A | G | 0.0098 |
| 2017 | 8-Jun-17 | FRESNO | GRAPES | Redacted4 | 0.3937 | 16 | A | G | 0.0123 |
| 2017 | 21-Jun-17 | FRESNO | GRAPES | Redacted4 | 0.3937 | 16 | A | G | 0.0123 |
| 2017 | 7-Jul-17 | KERN | GRAPES | Redacted4 | 5.2069 | 100 | A | G | 0.0260 |
| 2017 | 7-Jul-17 | KERN | GRAPES | Redacted4 | 2.0996 | 40 | A | G | 0.0262 |
| 2017 | 7-Jun-17 | SAN JOAQUIN | GRAPES | Redacted4 | 3.0234 | 18 | A | G | 0.0840 |
| 2017 | 22-Jul-17 | FRESNO | GRAPES | Redacted4 | 3.9472 | 20 | A | G | 0.0987 |
| 2017 | 17-Aug-17 | FRESNO | GRAPES | Redacted4 | 3.9472 | 20 | A | G | 0.0987 |
| 2017 | 22-Jul-17 | FRESNO | GRAPES | Redacted4 | 3.9472 | 20 | A | G | 0.0987 |
| 2017 | 18-Aug-17 | FRESNO | GRAPES | Redacted4 | 3.9472 | 20 | A | G | 0.0987 |
| 2017 | 17-Aug-17 | FRESNO | GRAPES | Redacted4 | 3.9472 | 20 | A | G | 0.0987 |
| 2017 | 18-Aug-17 | FRESNO | GRAPES | Redacted4 | 3.9472 | 20 | A | G | 0.0987 |
| 2017 | 23-May-17 | FRESNO | GRAPES | Redacted4 | 11.8416 | 60 | A | G | 0.0987 |
| 2017 | 25-Aug-17 | FRESNO | GRAPES | Redacted4 | 4.3671 | 22 | A | G | 0.0993 |
| 2017 | 6-Jul-17 | FRESNO | GRAPES | Redacted4 | 5.2489 | 25 | A | G | 0.1050 |
| 2017 | 6-Jul-17 | FRESNO | GRAPES | Redacted4 | 3.1494 | 15 | A | G | 0.1050 |
| 2017 | 19-May-17 | FRESNO | GRAPES | Redacted4 | 5.039 | 22 | A | G | 0.1145 |
| 2017 | 1-Jun-17 | MADERA | GRAPES | Redacted4 | 16.0408 | 70 | A | G | 0.1146 |
| 2017 | 13-Jun-17 | MADERA | GRAPES | Redacted4 | 18.5603 | 70.9 | A | G | 0.1309 |
| 2017 | 13-Jun-17 | MADERA | GRAPES | Redacted4 | 25.9508 | 99 | A | G | 0.1311 |
| 2017 | 13-Jun-17 | MADERA | GRAPES | Redacted4 | 9.1542 | 34.9 | A | G | 0.1311 |
| 2017 | 7-Jul-17 | FRESNO | GRAPES | Redacted4 | 3.9472 | 15 | A | G | 0.1316 |
| 2017 | 7-Jul-17 | FRESNO | GRAPES | Redacted4 | 3.9472 | 15 | A | G | 0.1316 |
| 2017 | 29-Jun-17 | FRESNO | GRAPES | Redacted4 | 5.2909 | 20 | A | G | 0.1323 |
| 2017 | 26-Jun-17 | FRESNO | GRAPES | Redacted4 | 6.5507 | 20 | A | G | 0.1638 |
| 2017 | 10-Jun-17 | MADERA | GRAPES | Redacted4 | 38.9681 | 99 | A | G | 0.1968 |
| 2017 | 14-Dec-17 | SAN DIEGO | GRAPES, WINE | Redacted4 | 3.4774 | 55 | A | G | 0.0316 |
| 2017 | 12-Jul-17 | RIVERSIDE | GRAPES, WINE | Redacted4 | 0.9842 | 7 | A | G | 0.0703 |
| 2017 | 27-Jun-17 | RIVERSIDE | GRAPES, WINE | Redacted4 | 0.9842 | 7 | A | G | 0.0703 |
| 2017 | 21-Aug-17 | SANTA BARBARA | GRAPES, WINE | Redacted4 | 1.6797 | 10.69 | A | G | 0.0786 |
| 2017 | 23-Aug-17 | SANTA BARBARA | GRAPES, WINE | Redacted4 | 1.0918 | 6.84 | A | G | 0.0798 |
| 2017 | 28-Jul-17 | SANTA BARBARA | GRAPES, WINE | Redacted4 | 5.039 | 30.93 | A | G | 0.0815 |
| 2017 | 25-May-17 | FRESNO | GRAPES, WINE | Redacted4 | 5.7948 | 35.45 | A | G | 0.0817 |
| 2017 | 22-May-17 | FRESNO | GRAPES, WINE | Redacted4 | 6.2147 | 38 | A | G | 0.0818 |
| 2017 | 24-May-17 | FRESNO | GRAPES, WINE | Redacted4 | 12.4295 | 76 | A | G | 0.0818 |
| 2017 | 23-Aug-17 | SANTA BARBARA | GRAPES, WINE | Redacted4 | 13.9412 | 85.24 | A | G | 0.0818 |
| 2017 | 18-Aug-17 | SANTA BARBARA | GRAPES, WINE | Redacted4 | 51.3136 | 313.01 | A | G | 0.0820 |
| 2017 | 31-Aug-17 | SANTA BARBARA | GRAPES, WINE | Redacted4 | 76.6765 | 467.36 | A | G | 0.0820 |
| 2017 | 19-Oct-17 | SANTA BARBARA | GRAPES, WINE | Redacted4 | 76.6765 | 467.36 | A | G | 0.0820 |
| 2017 | 23-Aug-17 | SANTA BARBARA | GRAPES, WINE | Redacted4 | 12.8494 | 78.28 | A | G | 0.0821 |
| 2017 | 19-Oct-17 | SANTA BARBARA | GRAPES, WINE | Redacted4 | 17.7204 | 107.87 | A | G | 0.0821 |
| 2017 | 14-Sep-17 | SANTA BARBARA | GRAPES, WINE | Redacted4 | 2.0996 | 12.77 | A | G | 0.0822 |
| 2017 | 26-Oct-17 | SANTA BARBARA | GRAPES, WINE | Redacted4 | 8.4823 | 51.52 | A | G | 0.0823 |
| 2017 | 7-Aug-17 | SANTA BARBARA | GRAPES, WINE | Redacted4 | 3.6113 | 21.9 | A | G | 0.0824 |
| 2017 | 20-Oct-17 | SANTA BARBARA | GRAPES, WINE | Redacted4 | 3.2753 | 19.84 | A | G | 0.0825 |
| 2017 | 24-Aug-17 | SANTA BARBARA | GRAPES, WINE | Redacted4 | 2.6035 | 15.74 | A | G | 0.0827 |
| 2017 | 28-Aug-17 | SANTA BARBARA | GRAPES, WINE | Redacted4 | 1.3437 | 8.07 | A | G | 0.0833 |
| 2017 | 22-Aug-17 | SANTA BARBARA | GRAPES, WINE | Redacted4 | 0.5879 | 3.45 | A | G | 0.0852 |
| 2017 | 13-Jun-17 | SAN JOAQUIN | GRAPES, WINE | Redacted4 | 2.0156 | 11.8 | A | G | 0.0854 |
| 2017 | 31-Oct-17 | SAN DIEGO | GRAPES, WINE | Redacted4 | 1.7059 | 9 | A | G | 0.0948 |
| 2017 | 23-Jun-17 | RIVERSIDE | GRAPES, WINE | Redacted4 | 2.9525 | 14 | A | G | 0.1054 |
| 2017 | 18-Jul-17 | SANTA BARBARA | GRAPES, WINE | Redacted4 | 0.2519 | 1.05 | A | G | 0.1200 |
| 2017 | 15-Jun-17 | SANTA BARBARA | GRAPES, WINE | Redacted4 | 1.5957 | 6.19 | A | G | 0.1289 |
| 2017 | 20-Jun-17 | SANTA BARBARA | GRAPES, WINE | Redacted4 | 3.0234 | 11.68 | A | G | 0.1294 |
| 2017 | 21-Jul-17 | SANTA BARBARA | GRAPES, WINE | Redacted4 | 3.0234 | 11.68 | A | G | 0.1294 |
| 2017 | 29-Jun-17 | SANTA BARBARA | GRAPES, WINE | Redacted4 | 5.4589 | 21.08 | A | G | 0.1295 |
| 2017 | 15-Jun-17 | SANTA BARBARA | GRAPES, WINE | Redacted4 | 5.4589 | 21.08 | A | G | 0.1295 |
| 2017 | 21-Jul-17 | SANTA BARBARA | GRAPES, WINE | Redacted4 | 2.2675 | 8.74 | A | G | 0.1297 |
| 2017 | 17-Jul-17 | SANTA BARBARA | GRAPES, WINE | Redacted4 | 4.1152 | 15.85 | A | G | 0.1298 |
| 2017 | 20-Jul-17 | SANTA BARBARA | GRAPES, WINE | Redacted4 | 5.039 | 19.39 | A | G | 0.1299 |
| 2017 | 22-Jul-17 | SANTA BARBARA | GRAPES, WINE | Redacted4 | 4.2831 | 16.46 | A | G | 0.1301 |
| 2017 | 13-Jun-17 | MERCED | GRAPES, WINE | Redacted4 | 7.8104 | 30 | A | G | 0.1302 |
| 2017 | 11-Aug-17 | SANTA CLARA | GRAPES, WINE | Redacted4 | 2.6035 | 10 | A | G | 0.1302 |
| 2017 | 18-Jul-17 | SANTA BARBARA | GRAPES, WINE | Redacted4 | 6.6347 | 25.48 | A | G | 0.1302 |
| 2017 | 17-Jul-17 | SANTA BARBARA | GRAPES, WINE | Redacted4 | 3.7792 | 14.5 | A | G | 0.1303 |
| 2017 | 13-Jun-17 | MADERA | GRAPES, WINE | Redacted4 | 7.5585 | 29 | A | G | 0.1303 |
| 2017 | 24-May-17 | FRESNO | GRAPES, WINE | Redacted4 | 9.91 | 38 | A | G | 0.1304 |
| 2017 | 19-Jul-17 | SANTA BARBARA | GRAPES, WINE | Redacted4 | 1.3437 | 5.14 | A | G | 0.1307 |
| 2017 | 13-Jun-17 | MADERA | GRAPES, WINE | Redacted4 | 20.6598 | 79 | A | G | 0.1308 |
| 2017 | 16-Jun-17 | SANTA BARBARA | GRAPES, WINE | Redacted4 | 23.5153 | 89.9 | A | G | 0.1308 |
| 2017 | 25-May-17 | FRESNO | GRAPES, WINE | Redacted4 | 20.4079 | 78 | A | G | 0.1308 |
| 2017 | 13-Jun-17 | SANTA BARBARA | GRAPES, WINE | Redacted4 | 17.4685 | 66.76 | A | G | 0.1308 |
| 2017 | 27-Jun-17 | SANTA BARBARA | GRAPES, WINE | Redacted4 | 17.4685 | 66.76 | A | G | 0.1308 |
| 2017 | 24-Jul-17 | SANTA BARBARA | GRAPES, WINE | Redacted4 | 7.2225 | 27.6 | A | G | 0.1308 |
| 2017 | 13-Jun-17 | MADERA | GRAPES, WINE | Redacted4 | 22.5075 | 86 | A | G | 0.1309 |
| 2017 | 13-Jun-17 | MADERA | GRAPES, WINE | Redacted4 | 22.5075 | 86 | A | G | 0.1309 |
| 2017 | 28-Jun-17 | SANTA BARBARA | GRAPES, WINE | Redacted4 | 28.6382 | 109.4 | A | G | 0.1309 |
| 2017 | 14-Jun-17 | SANTA BARBARA | GRAPES, WINE | Redacted4 | 28.6382 | 109.4 | A | G | 0.1309 |
| 2017 | 13-Jun-17 | MADERA | GRAPES, WINE | Redacted4 | 17.3845 | 66.4 | A | G | 0.1309 |
| 2017 | 29-Jun-17 | SANTA BARBARA | GRAPES, WINE | Redacted4 | 13.5213 | 51.64 | A | G | 0.1309 |
| 2017 | 19-Jul-17 | SANTA BARBARA | GRAPES, WINE | Redacted4 | 28.4703 | 108.73 | A | G | 0.1309 |
| 2017 | 20-Jun-17 | SANTA BARBARA | GRAPES, WINE | Redacted4 | 4.871 | 18.6 | A | G | 0.1309 |
| 2017 | 24-May-17 | FRESNO | GRAPES, WINE | Redacted4 | 19.904 | 76 | A | G | 0.1309 |
| 2017 | 29-Jun-17 | SANTA BARBARA | GRAPES, WINE | Redacted4 | 29.4781 | 112.55 | A | G | 0.1310 |
| 2017 | 21-Jul-17 | SANTA BARBARA | GRAPES, WINE | Redacted4 | 19.4001 | 74.07 | A | G | 0.1310 |
| 2017 | 20-Jun-17 | SANTA BARBARA | GRAPES, WINE | Redacted4 | 19.4001 | 74.07 | A | G | 0.1310 |
| 2017 | 24-Jul-17 | SANTA BARBARA | GRAPES, WINE | Redacted4 | 20.8278 | 79.49 | A | G | 0.1310 |
| 2017 | 13-Jun-17 | MADERA | GRAPES, WINE | Redacted4 | 39.3041 | 150 | A | G | 0.1310 |
| 2017 | 3-Jul-17 | SANTA BARBARA | GRAPES, WINE | Redacted4 | 33.2573 | 126.91 | A | G | 0.1310 |
| 2017 | 28-May-17 | SANTA BARBARA | GRAPES, WINE | Redacted4 | 7.3905 | 28.2 | A | G | 0.1310 |
| 2017 | 19-Jun-17 | SANTA BARBARA | GRAPES, WINE | Redacted4 | 28.3863 | 108.31 | A | G | 0.1310 |
| 2017 | 3-Jul-17 | SANTA BARBARA | GRAPES, WINE | Redacted4 | 25.8668 | 98.69 | A | G | 0.1311 |
| 2017 | 19-Jun-17 | SANTA BARBARA | GRAPES, WINE | Redacted4 | 28.5542 | 108.94 | A | G | 0.1311 |
| 2017 | 28-May-17 | SANTA BARBARA | GRAPES, WINE | Redacted4 | 28.5542 | 108.94 | A | G | 0.1311 |
| 2017 | 20-Jul-17 | SANTA BARBARA | GRAPES, WINE | Redacted4 | 28.5542 | 108.94 | A | G | 0.1311 |
| 2017 | 21-Jul-17 | SANTA BARBARA | GRAPES, WINE | Redacted4 | 19.0642 | 72.72 | A | G | 0.1311 |
| 2017 | 18-Jul-17 | SANTA BARBARA | GRAPES, WINE | Redacted4 | 15.7048 | 59.9 | A | G | 0.1311 |
| 2017 | 1-Jun-17 | SANTA BARBARA | GRAPES, WINE | Redacted4 | 40.5638 | 154.71 | A | G | 0.1311 |
| 2017 | 1-Jul-17 | SANTA BARBARA | GRAPES, WINE | Redacted4 | 2.6875 | 10.25 | A | G | 0.1311 |
| 2017 | 13-Jun-17 | SANTA BARBARA | GRAPES, WINE | Redacted4 | 17.8044 | 67.9 | A | G | 0.1311 |
| 2017 | 31-May-17 | SANTA BARBARA | GRAPES, WINE | Redacted4 | 17.8044 | 67.9 | A | G | 0.1311 |
| 2017 | 17-Jul-17 | SANTA BARBARA | GRAPES, WINE | Redacted4 | 17.8044 | 67.9 | A | G | 0.1311 |
| 2017 | 27-Jun-17 | SANTA BARBARA | GRAPES, WINE | Redacted4 | 17.8044 | 67.9 | A | G | 0.1311 |
| 2017 | 13-Jun-17 | MERCED | GRAPES, WINE | Redacted4 | 49.2981 | 188 | A | G | 0.1311 |
| 2017 | 13-Jun-17 | MADERA | GRAPES, WINE | Redacted4 | 57.6964 | 220 | A | G | 0.1311 |
| 2017 | 1-Jun-17 | SANTA BARBARA | GRAPES, WINE | Redacted4 | 8.3143 | 31.7 | A | G | 0.1311 |
| 2017 | 28-Jun-17 | SANTA BARBARA | GRAPES, WINE | Redacted4 | 8.3143 | 31.7 | A | G | 0.1311 |
| 2017 | 14-Jun-17 | SANTA BARBARA | GRAPES, WINE | Redacted4 | 8.3143 | 31.7 | A | G | 0.1311 |
| 2017 | 19-Jul-17 | SANTA BARBARA | GRAPES, WINE | Redacted4 | 25.3629 | 96.7 | A | G | 0.1311 |
| 2017 | 15-Jun-17 | SANTA BARBARA | GRAPES, WINE | Redacted4 | 25.3629 | 96.7 | A | G | 0.1311 |
| 2017 | 31-May-17 | SANTA BARBARA | GRAPES, WINE | Redacted4 | 5.6269 | 21.45 | A | G | 0.1312 |
| 2017 | 17-Jul-17 | SANTA BARBARA | GRAPES, WINE | Redacted4 | 5.6269 | 21.45 | A | G | 0.1312 |
| 2017 | 13-Jun-17 | MADERA | GRAPES, WINE | Redacted4 | 14.6131 | 55.7 | A | G | 0.1312 |
| 2017 | 2-Jun-17 | SANTA BARBARA | GRAPES, WINE | Redacted4 | 48.9621 | 186.62 | A | G | 0.1312 |
| 2017 | 19-Jul-17 | SANTA BARBARA | GRAPES, WINE | Redacted4 | 11.9256 | 45.45 | A | G | 0.1312 |
| 2017 | 18-Jul-17 | SANTA BARBARA | GRAPES, WINE | Redacted4 | 17.6364 | 67.21 | A | G | 0.1312 |
| 2017 | 17-Jun-17 | SANTA BARBARA | GRAPES, WINE | Redacted4 | 36.3647 | 138.57 | A | G | 0.1312 |
| 2017 | 1-Jul-17 | SANTA BARBARA | GRAPES, WINE | Redacted4 | 8.8182 | 33.6 | A | G | 0.1312 |
| 2017 | 13-Jun-17 | MADERA | GRAPES, WINE | Redacted4 | 41.9915 | 160 | A | G | 0.1312 |
| 2017 | 7-Jul-17 | SANTA BARBARA | GRAPES, WINE | Redacted4 | 44.616 | 170 | A | G | 0.1312 |
| 2017 | 13-Jun-17 | MADERA | GRAPES, WINE | Redacted4 | 16.3767 | 62.4 | A | G | 0.1312 |
| 2017 | 13-Jun-17 | MADERA | GRAPES, WINE | Redacted4 | 20.9958 | 80 | A | G | 0.1312 |
| 2017 | 13-Jun-17 | MADERA | GRAPES, WINE | Redacted4 | 10.4979 | 40 | A | G | 0.1312 |
| 2017 | 7-Jul-17 | SANTA BARBARA | GRAPES, WINE | Redacted4 | 4.1992 | 16 | A | G | 0.1312 |
| 2017 | 30-Jun-17 | SANTA BARBARA | GRAPES, WINE | Redacted4 | 51.1457 | 194.87 | A | G | 0.1312 |
| 2017 | 6-Jun-17 | SANTA BARBARA | GRAPES, WINE | Redacted4 | 34.937 | 133.1 | A | G | 0.1312 |
| 2017 | 3-Jun-17 | SANTA BARBARA | GRAPES, WINE | Redacted4 | 65.5068 | 249.55 | A | G | 0.1312 |
| 2017 | 17-Jul-17 | SANTA BARBARA | GRAPES, WINE | Redacted4 | 4.5351 | 17.2 | A | G | 0.1318 |
| 2017 | 3-May-17 | SANTA BARBARA | GRAPES, WINE | Redacted4 | 45.1409 | 170 | A | G | 0.1328 |
| 2017 | 31-May-17 | SANTA BARBARA | GRAPES, WINE | Redacted4 | 0.8398 | 3.12 | A | G | 0.1346 |
| 2017 | 17-Jul-17 | SANTA BARBARA | GRAPES, WINE | Redacted4 | 0.8398 | 3.12 | A | G | 0.1346 |
| 2017 | 13-Jun-17 | SANTA BARBARA | GRAPES, WINE | Redacted4 | 0.8398 | 3.12 | A | G | 0.1346 |
| 2017 | 27-Jun-17 | SANTA BARBARA | GRAPES, WINE | Redacted4 | 0.8398 | 3.12 | A | G | 0.1346 |
| 2017 | 17-May-17 | SANTA BARBARA | GRAPES, WINE | Redacted4 | 1.9316 | 6.19 | A | G | 0.1560 |
| 2017 | 16-May-17 | SANTA BARBARA | GRAPES, WINE | Redacted4 | 9.91 | 31.7 | A | G | 0.1563 |
| 2017 | 17-May-17 | SANTA BARBARA | GRAPES, WINE | Redacted4 | 21.8356 | 69.46 | A | G | 0.1572 |
| 2017 | 16-May-17 | SANTA BARBARA | GRAPES, WINE | Redacted4 | 20.9958 | 66.76 | A | G | 0.1572 |
| 2017 | 19-May-17 | SANTA BARBARA | GRAPES, WINE | Redacted4 | 17.0486 | 54.19 | A | G | 0.1573 |
| 2017 | 17-May-17 | SANTA BARBARA | GRAPES, WINE | Redacted4 | 16.9646 | 53.88 | A | G | 0.1574 |
| 2017 | 18-May-17 | SANTA BARBARA | GRAPES, WINE | Redacted4 | 54.9249 | 174.42 | A | G | 0.1575 |
| 2017 | 5-May-17 | SANTA BARBARA | GRAPES, WINE | Redacted4 | 25.6542 | 81.46 | A | G | 0.1575 |
| 2017 | 2-May-17 | SANTA BARBARA | GRAPES, WINE | Redacted4 | 29.9879 | 95.22 | A | G | 0.1575 |
| 2017 | 4-May-17 | SANTA BARBARA | GRAPES, WINE | Redacted4 | 44.349 | 140.82 | A | G | 0.1575 |
| 2017 | 3-May-17 | SANTA BARBARA | GRAPES, WINE | Redacted4 | 16.2632 | 51.64 | A | G | 0.1575 |
| 2017 | 3-May-17 | SANTA BARBARA | GRAPES, WINE | Redacted4 | 34.2428 | 108.73 | A | G | 0.1575 |
| 2017 | 1-May-17 | SANTA BARBARA | GRAPES, WINE | Redacted4 | 25.4908 | 80.94 | A | G | 0.1575 |
| 2017 | 5-May-17 | SANTA BARBARA | GRAPES, WINE | Redacted4 | 31.0809 | 98.69 | A | G | 0.1575 |
| 2017 | 2-May-17 | SANTA BARBARA | GRAPES, WINE | Redacted4 | 19.5418 | 62.05 | A | G | 0.1575 |
| 2017 | 4-May-17 | SANTA BARBARA | GRAPES, WINE | Redacted4 | 3.2281 | 10.25 | A | G | 0.1575 |
| 2017 | 3-May-17 | SANTA BARBARA | GRAPES, WINE | Redacted4 | 15.9043 | 50.5 | A | G | 0.1575 |
| 2017 | 1-May-17 | SANTA BARBARA | GRAPES, WINE | Redacted4 | 21.3842 | 67.9 | A | G | 0.1575 |
| 2017 | 2-May-17 | SANTA BARBARA | GRAPES, WINE | Redacted4 | 9.9835 | 31.7 | A | G | 0.1575 |
| 2017 | 1-May-17 | SANTA BARBARA | GRAPES, WINE | Redacted4 | 0.9829 | 3.12 | A | G | 0.1575 |
| 2017 | 19-May-17 | SANTA BARBARA | GRAPES, WINE | Redacted4 | 33.5092 | 106.32 | A | G | 0.1576 |
| 2017 | 20-May-17 | SANTA BARBARA | GRAPES, WINE | Redacted4 | 23.3473 | 74.07 | A | G | 0.1576 |
| 2017 | 20-May-17 | SANTA BARBARA | GRAPES, WINE | Redacted4 | 22.9274 | 72.72 | A | G | 0.1576 |
| 2017 | 16-May-17 | SANTA BARBARA | GRAPES, WINE | Redacted4 | 21.4157 | 67.9 | A | G | 0.1577 |
| 2017 | 20-May-17 | SANTA BARBARA | GRAPES, WINE | Redacted4 | 3.6953 | 11.68 | A | G | 0.1582 |
| 2017 | 18-May-17 | SANTA BARBARA | GRAPES, WINE | Redacted4 | 0.8398 | 2.62 | A | G | 0.1603 |
| 2017 | 16-May-17 | SANTA BARBARA | GRAPES, WINE | Redacted4 | 1.0078 | 3.12 | A | G | 0.1615 |
| 2017 | 28-Jun-17 | FRESNO | GRAPES, WINE | Redacted4 | 12.4295 | 38 | A | G | 0.1635 |
| 2017 | 15-Jun-17 | MADERA | GRAPES, WINE | Redacted4 | 24.607 | 75 | A | G | 0.1640 |
| 2017 | 22-Jun-17 | SANTA BARBARA | GRAPES, WINE | Redacted4 | 12.7654 | 32.5 | A | G | 0.1964 |
| 2017 | 6-Jul-17 | SANTA BARBARA | GRAPES, WINE | Redacted4 | 1.181 | 3 | A | G | 0.1968 |
| 2017 | 13-Jul-17 | SANTA BARBARA | GRAPES, WINE | Redacted4 | 4.1335 | 10.5 | A | G | 0.1968 |
| 2017 | 16-Jun-17 | SAN LUIS OBISPO | GRAPES, WINE | Redacted4 | 7.8734 | 20 | A | G | 0.1968 |
| 2017 | 13-Jul-17 | SAN LUIS OBISPO | GRAPES, WINE | Redacted4 | 7.8734 | 20 | A | G | 0.1968 |
| 2017 | 7-Jul-17 | SANTA BARBARA | GRAPES, WINE | Redacted4 | 12.7943 | 32.5 | A | G | 0.1968 |
| 2017 | 13-Jul-17 | SAN LUIS OBISPO | GRAPES, WINE | Redacted4 | 9.4481 | 24 | A | G | 0.1968 |
| 2017 | 15-Jun-17 | SAN LUIS OBISPO | GRAPES, WINE | Redacted4 | 9.4481 | 24 | A | G | 0.1968 |
| 2017 | 24-Jul-17 | SAN LUIS OBISPO | GRAPES, WINE | Redacted4 | 2.1652 | 5.5 | A | G | 0.1968 |
| 2017 | 7-Jul-17 | SAN LUIS OBISPO | GRAPES, WINE | Redacted4 | 2.1652 | 5.5 | A | G | 0.1968 |
| 2017 | 14-Aug-17 | SANTA BARBARA | GRAPES, WINE | Redacted4 | 1.5747 | 4 | A | G | 0.1968 |
| 2017 | 21-Jun-17 | SAN LUIS OBISPO | GRAPES, WINE | Redacted4 | 2.1836 | 5.5 | A | G | 0.1985 |
| 2017 | 17-Jul-17 | SANTA BARBARA | GRAPES, WINE | Redacted4 | 1.5957 | 4 | A | G | 0.1995 |
| 2017 | 19-Jul-17 | SANTA BARBARA | GRAPES, WINE | Redacted4 | 17.0486 | 32.5 | A | G | 0.2623 |
| 2017 | 11-Aug-17 | SANTA BARBARA | GRAPES, WINE | Redacted4 | 17.0486 | 32.5 | A | G | 0.2623 |
| 2017 | 21-Jul-17 | SANTA BARBARA | GRAPES, WINE | Redacted4 | 1.5747 | 3 | A | G | 0.2625 |
| 2017 | 8-Aug-17 | SANTA BARBARA | GRAPES, WINE | Redacted4 | 1.5957 | 3 | A | G | 0.2660 |
| 2017 | 26-Jul-17 | FRESNO | GRAPES, WINE | Redacted4 | 14.697 | 25 | A | G | 0.2939 |
| 2017 | 10-Jun-17 | FRESNO | GRAPES, WINE | Redacted4 | 14.697 | 25 | A | G | 0.2939 |
| 2017 | 7-Jun-17 | FRESNO | GRAPES, WINE | Redacted4 | 65.8427 | 112 | A | G | 0.2939 |
| 2017 | 12-Jun-17 | FRESNO | GRAPES, WINE | Redacted4 | 10.5819 | 18 | A | G | 0.2939 |
| 2017 | 26-Jul-17 | FRESNO | GRAPES, WINE | Redacted4 | 10.5819 | 18 | A | G | 0.2939 |
| 2017 | 3-Jun-17 | FRESNO | GRAPES, WINE | Redacted4 | 49.55 | 84 | A | G | 0.2949 |
| 2017 | 2-Jun-17 | FRESNO | GRAPES, WINE | Redacted4 | 46.0227 | 78 | A | G | 0.2950 |
| 2017 | 11-Jun-17 | FRESNO | GRAPES, WINE | Redacted4 | 66.0947 | 112 | A | G | 0.2951 |
| 2017 | 27-Jul-17 | FRESNO | GRAPES, WINE | Redacted4 | 66.0947 | 112 | A | G | 0.2951 |
| 2017 | 17-Jun-17 | MADERA | GRAPES, WINE | Redacted4 | 55.2609 | 70 | A | G |  |
| 2017 | 10-Jun-17 | MADERA | GRAPES, WINE | Redacted4 | 101.0421 | 110 | A | G |  |
| 2017 | 9-Jun-17 | FRESNO | GRAPES, WINE | Redacted4 | 102.8792 | 85 | A | G |  |
| 2017 | 4-Nov-17 | VENTURA | KALE | Redacted4 | 0.7558 | 4 | A | G | 0.0945 |
| 2017 | 4-Nov-17 | VENTURA | KALE | Redacted4 | 1.2597 | 6.4 | A | G | 0.0984 |
| 2017 | 16-May-17 | VENTURA | KALE | Redacted4 | 1.5957 | 8 | A | G | 0.0997 |
| 2017 | 16-Jun-17 | SANTA BARBARA | KALE | Redacted4 | 0.2519 | 1 | A | G | 0.1260 |
| 2017 | 16-Jun-17 | SANTA BARBARA | KALE | Redacted4 | 0.2519 | 1 | A | G | 0.1260 |
| 2017 | 9-Sep-17 | SANTA BARBARA | KALE | Redacted4 | 2.3515 | 9 | A | G | 0.1306 |
| 2017 | 20-Oct-17 | SANTA BARBARA | KALE | Redacted4 | 2.3515 | 9 | A | G | 0.1306 |
| 2017 | 21-Jul-17 | SANTA BARBARA | KALE | Redacted4 | 0.2624 | 1 | A | G | 0.1312 |
| 2017 | 21-Jul-17 | SANTA BARBARA | KALE | Redacted4 | 0.2624 | 1 | A | G | 0.1312 |
| 2017 | 18-Jul-17 | SANTA BARBARA | KALE | Redacted4 | 0.6036 | 2.3 | A | G | 0.1312 |
| 2017 | 9-Sep-17 | SANTA BARBARA | KALE | Redacted4 | 1.8476 | 7 | A | G | 0.1320 |
| 2017 | 9-Sep-17 | SANTA BARBARA | KALE | Redacted4 | 1.3437 | 5 | A | G | 0.1344 |
| 2017 | 1-Oct-17 | SANTA BARBARA | KALE | Redacted4 | 0.168 | 0.5 | A | G | 0.1680 |
| 2017 | 16-Oct-17 | SANTA BARBARA | KALE | Redacted4 | 0.168 | 0.5 | A | G | 0.1680 |
| 2017 | 16-Oct-17 | SANTA BARBARA | KALE | Redacted4 | 0.168 | 0.5 | A | G | 0.1680 |
| 2017 | 18-Aug-17 | SANTA BARBARA | KALE | Redacted4 | 0.168 | 0.5 | A | G | 0.1680 |
| 2017 | 21-Sep-17 | SANTA BARBARA | KALE | Redacted4 | 0.168 | 0.5 | A | G | 0.1680 |
| 2017 | 2-Aug-17 | VENTURA | KALE | Redacted4 | 0.168 | 0.5 | A | G | 0.1680 |
| 2017 | 27-Jul-17 | VENTURA | KALE | Redacted4 | 0.168 | 0.5 | A | G | 0.1680 |
| 2017 | 21-Jul-17 | VENTURA | KALE | Redacted4 | 0.168 | 0.5 | A | G | 0.1680 |
| 2017 | 1-Sep-17 | SANTA BARBARA | KALE | Redacted4 | 2.5195 | 6.5 | A | G | 0.1938 |
| 2017 | 1-Sep-17 | SANTA BARBARA | KALE | Redacted4 | 2.9394 | 7.5 | A | G | 0.1960 |
| 2017 | 1-Sep-17 | SANTA BARBARA | KALE | Redacted4 | 3.5273 | 9 | A | G | 0.1960 |
| 2017 | 25-Sep-17 | SANTA BARBARA | KALE | Redacted4 | 3.5273 | 9 | A | G | 0.1960 |
| 2017 | 29-Sep-17 | SANTA BARBARA | KALE | Redacted4 | 0.1968 | 0.5 | A | G | 0.1968 |
| 2017 | 11-Aug-17 | SANTA BARBARA | KALE | Redacted4 | 0.1968 | 0.5 | A | G | 0.1968 |
| 2017 | 6-Oct-17 | SANTA BARBARA | KALE | Redacted4 | 2.362 | 6 | A | G | 0.1968 |
| 2017 | 22-Jun-17 | SANTA BARBARA | KALE | Redacted4 | 3.543 | 9 | A | G | 0.1968 |
| 2017 | 25-Jul-17 | SANTA BARBARA | KALE | Redacted4 | 3.543 | 9 | A | G | 0.1968 |
| 2017 | 3-Aug-17 | SANTA BARBARA | KALE | Redacted4 | 5.7476 | 14.6 | A | G | 0.1968 |
| 2017 | 29-Sep-17 | SANTA BARBARA | KALE | Redacted4 | 5.7476 | 14.6 | A | G | 0.1968 |
| 2017 | 11-Aug-17 | SANTA BARBARA | KALE | Redacted4 | 2.7557 | 7 | A | G | 0.1968 |
| 2017 | 22-Aug-17 | SANTA BARBARA | KALE | Redacted4 | 5.5114 | 14 | A | G | 0.1968 |
| 2017 | 11-Aug-17 | SANTA BARBARA | KALE | Redacted4 | 1.9684 | 5 | A | G | 0.1968 |
| 2017 | 3-Jul-17 | SANTA BARBARA | KALE | Redacted4 | 2.7714 | 7 | A | G | 0.1980 |
| 2017 | 18-Aug-17 | SANTA BARBARA | KALE | Redacted4 | 2.7714 | 7 | A | G | 0.1980 |
| 2017 | 14-Jul-17 | SANTA BARBARA | KALE | Redacted4 | 5.5429 | 14 | A | G | 0.1980 |
| 2017 | 22-Sep-17 | SANTA BARBARA | KALE | Redacted4 | 5.5429 | 14 | A | G | 0.1980 |
| 2017 | 16-Jun-17 | SANTA BARBARA | LETTUCE, HEAD (ALL OR UNSPEC) | Redacted4 | 0.8398 | 6.56 | A | G | 0.0640 |
| 2017 | 16-Jun-17 | SANTA BARBARA | LETTUCE, HEAD (ALL OR UNSPEC) | Redacted4 | 1.3437 | 10.4 | A | G | 0.0646 |
| 2017 | 20-Jun-17 | SANTA BARBARA | LETTUCE, HEAD (ALL OR UNSPEC) | Redacted4 | 2.1836 | 16.9 | A | G | 0.0646 |
| 2017 | 15-Jun-17 | SANTA BARBARA | LETTUCE, HEAD (ALL OR UNSPEC) | Redacted4 | 1.6797 | 12.92 | A | G | 0.0650 |
| 2017 | 14-Jun-17 | SANTA BARBARA | LETTUCE, HEAD (ALL OR UNSPEC) | Redacted4 | 0.7558 | 5.7 | A | G | 0.0663 |
| 2017 | 31-Aug-17 | SAN BENITO | LETTUCE, HEAD (ALL OR UNSPEC) | Redacted4 | 1.5957 | 12 | A | G | 0.0665 |
| 2017 | 27-Jun-17 | SANTA BARBARA | LETTUCE, HEAD (ALL OR UNSPEC) | Redacted4 | 0.168 | 1 | A | G | 0.0840 |
| 2017 | 27-Jun-17 | SANTA BARBARA | LETTUCE, HEAD (ALL OR UNSPEC) | Redacted4 | 0.168 | 1 | A | G | 0.0840 |
| 2017 | 12-Jul-17 | SAN LUIS OBISPO | LETTUCE, HEAD (ALL OR UNSPEC) | Redacted4 | 0.3359 | 1.8 | A | G | 0.0933 |
| 2017 | 25-May-17 | SANTA BARBARA | LETTUCE, HEAD (ALL OR UNSPEC) | Redacted4 | 0.8398 | 4.5 | A | G | 0.0933 |
| 2017 | 26-May-17 | SAN LUIS OBISPO | LETTUCE, HEAD (ALL OR UNSPEC) | Redacted4 | 0.7558 | 4 | A | G | 0.0945 |
| 2017 | 1-Jun-17 | SANTA BARBARA | LETTUCE, HEAD (ALL OR UNSPEC) | Redacted4 | 0.7558 | 4 | A | G | 0.0945 |
| 2017 | 17-May-17 | SAN LUIS OBISPO | LETTUCE, HEAD (ALL OR UNSPEC) | Redacted4 | 0.6719 | 3.5 | A | G | 0.0960 |
| 2017 | 4-Jun-17 | SANTA BARBARA | LETTUCE, HEAD (ALL OR UNSPEC) | Redacted4 | 0.9238 | 4.8 | A | G | 0.0962 |
| 2017 | 20-Jun-17 | SANTA BARBARA | LETTUCE, HEAD (ALL OR UNSPEC) | Redacted4 | 0.9238 | 4.8 | A | G | 0.0962 |
| 2017 | 11-Jun-17 | SAN LUIS OBISPO | LETTUCE, HEAD (ALL OR UNSPEC) | Redacted4 | 1.6797 | 8.7 | A | G | 0.0965 |
| 2017 | 17-May-17 | SAN LUIS OBISPO | LETTUCE, HEAD (ALL OR UNSPEC) | Redacted4 | 1.6797 | 8.7 | A | G | 0.0965 |
| 2017 | 27-Jun-17 | SANTA BARBARA | LETTUCE, HEAD (ALL OR UNSPEC) | Redacted4 | 1.0918 | 5.65 | A | G | 0.0966 |
| 2017 | 20-Jun-17 | SANTA BARBARA | LETTUCE, HEAD (ALL OR UNSPEC) | Redacted4 | 1.5117 | 7.8 | A | G | 0.0969 |
| 2017 | 2-Jun-17 | SANTA BARBARA | LETTUCE, HEAD (ALL OR UNSPEC) | Redacted4 | 2.4355 | 12.5 | A | G | 0.0974 |
| 2017 | 14-Jun-17 | SANTA BARBARA | LETTUCE, HEAD (ALL OR UNSPEC) | Redacted4 | 2.4355 | 12.5 | A | G | 0.0974 |
| 2017 | 3-Jul-17 | SANTA BARBARA | LETTUCE, HEAD (ALL OR UNSPEC) | Redacted4 | 2.6875 | 13.78 | A | G | 0.0975 |
| 2017 | 27-Jun-17 | SANTA BARBARA | LETTUCE, HEAD (ALL OR UNSPEC) | Redacted4 | 1.8476 | 9.46 | A | G | 0.0977 |
| 2017 | 31-May-17 | SANTA BARBARA | LETTUCE, HEAD (ALL OR UNSPEC) | Redacted4 | 1.4277 | 7.29 | A | G | 0.0979 |
| 2017 | 13-Jul-17 | SAN LUIS OBISPO | LETTUCE, HEAD (ALL OR UNSPEC) | Redacted4 | 2.3515 | 12 | A | G | 0.0980 |
| 2017 | 25-May-17 | SAN LUIS OBISPO | LETTUCE, HEAD (ALL OR UNSPEC) | Redacted4 | 1.5117 | 7.7 | A | G | 0.0982 |
| 2017 | 5-Aug-17 | SANTA BARBARA | LETTUCE, HEAD (ALL OR UNSPEC) | Redacted4 | 0.9238 | 4.7 | A | G | 0.0983 |
| 2017 | 2-Jun-17 | SANTA BARBARA | LETTUCE, HEAD (ALL OR UNSPEC) | Redacted4 | 1.0235 | 5.2 | A | G | 0.0984 |
| 2017 | 26-May-17 | SANTA BARBARA | LETTUCE, HEAD (ALL OR UNSPEC) | Redacted4 | 1.0235 | 5.2 | A | G | 0.0984 |
| 2017 | 11-Jul-17 | SANTA BARBARA | LETTUCE, HEAD (ALL OR UNSPEC) | Redacted4 | 1.2597 | 6.4 | A | G | 0.0984 |
| 2017 | 26-May-17 | SAN LUIS OBISPO | LETTUCE, HEAD (ALL OR UNSPEC) | Redacted4 | 0.6889 | 3.5 | A | G | 0.0984 |
| 2017 | 5-Jun-17 | SAN LUIS OBISPO | LETTUCE, HEAD (ALL OR UNSPEC) | Redacted4 | 0.6889 | 3.5 | A | G | 0.0984 |
| 2017 | 2-Jun-17 | SANTA BARBARA | LETTUCE, HEAD (ALL OR UNSPEC) | Redacted4 | 0.807 | 4.1 | A | G | 0.0984 |
| 2017 | 22-May-17 | SANTA BARBARA | LETTUCE, HEAD (ALL OR UNSPEC) | Redacted4 | 1.614 | 8.2 | A | G | 0.0984 |
| 2017 | 5-Jun-17 | SANTA BARBARA | LETTUCE, HEAD (ALL OR UNSPEC) | Redacted4 | 1.5156 | 7.7 | A | G | 0.0984 |
| 2017 | 15-Jun-17 | SANTA BARBARA | LETTUCE, HEAD (ALL OR UNSPEC) | Redacted4 | 1.5156 | 7.7 | A | G | 0.0984 |
| 2017 | 5-Jun-17 | SANTA BARBARA | LETTUCE, HEAD (ALL OR UNSPEC) | Redacted4 | 1.5156 | 7.7 | A | G | 0.0984 |
| 2017 | 25-May-17 | SANTA BARBARA | LETTUCE, HEAD (ALL OR UNSPEC) | Redacted4 | 1.5156 | 7.7 | A | G | 0.0984 |
| 2017 | 24-May-17 | SANTA BARBARA | LETTUCE, HEAD (ALL OR UNSPEC) | Redacted4 | 2.4604 | 12.5 | A | G | 0.0984 |
| 2017 | 2-Jun-17 | SAN LUIS OBISPO | LETTUCE, HEAD (ALL OR UNSPEC) | Redacted4 | 1.2991 | 6.6 | A | G | 0.0984 |
| 2017 | 25-May-17 | SAN LUIS OBISPO | LETTUCE, HEAD (ALL OR UNSPEC) | Redacted4 | 1.2991 | 6.6 | A | G | 0.0984 |
| 2017 | 30-May-17 | SANTA BARBARA | LETTUCE, HEAD (ALL OR UNSPEC) | Redacted4 | 3.4249 | 17.4 | A | G | 0.0984 |
| 2017 | 28-Jun-17 | SANTA BARBARA | LETTUCE, HEAD (ALL OR UNSPEC) | Redacted4 | 2.9466 | 14.97 | A | G | 0.0984 |
| 2017 | 20-Jun-17 | SANTA BARBARA | LETTUCE, HEAD (ALL OR UNSPEC) | Redacted4 | 3.4446 | 17.5 | A | G | 0.0984 |
| 2017 | 8-Jun-17 | SAN LUIS OBISPO | LETTUCE, HEAD (ALL OR UNSPEC) | Redacted4 | 1.555 | 7.9 | A | G | 0.0984 |
| 2017 | 30-Jun-17 | SANTA BARBARA | LETTUCE, HEAD (ALL OR UNSPEC) | Redacted4 | 3.3344 | 16.94 | A | G | 0.0984 |
| 2017 | 29-Jun-17 | SANTA BARBARA | LETTUCE, HEAD (ALL OR UNSPEC) | Redacted4 | 2.5195 | 12.8 | A | G | 0.0984 |
| 2017 | 25-May-17 | SANTA BARBARA | LETTUCE, HEAD (ALL OR UNSPEC) | Redacted4 | 3.6021 | 18.3 | A | G | 0.0984 |
| 2017 | 1-Jun-17 | SANTA BARBARA | LETTUCE, HEAD (ALL OR UNSPEC) | Redacted4 | 3.1297 | 15.9 | A | G | 0.0984 |
| 2017 | 9-Jun-17 | SANTA BARBARA | LETTUCE, HEAD (ALL OR UNSPEC) | Redacted4 | 3.4348 | 17.45 | A | G | 0.0984 |
| 2017 | 9-Jun-17 | SANTA BARBARA | LETTUCE, HEAD (ALL OR UNSPEC) | Redacted4 | 2.1219 | 10.78 | A | G | 0.0984 |
| 2017 | 24-May-17 | SANTA BARBARA | LETTUCE, HEAD (ALL OR UNSPEC) | Redacted4 | 2.1219 | 10.78 | A | G | 0.0984 |
| 2017 | 5-Jun-17 | SANTA BARBARA | LETTUCE, HEAD (ALL OR UNSPEC) | Redacted4 | 2.1219 | 10.78 | A | G | 0.0984 |
| 2017 | 30-May-17 | SAN LUIS OBISPO | LETTUCE, HEAD (ALL OR UNSPEC) | Redacted4 | 1.7125 | 8.7 | A | G | 0.0984 |
| 2017 | 7-Jul-17 | SAN LUIS OBISPO | LETTUCE, HEAD (ALL OR UNSPEC) | Redacted4 | 1.5944 | 8.1 | A | G | 0.0984 |
| 2017 | 27-Jun-17 | SAN LUIS OBISPO | LETTUCE, HEAD (ALL OR UNSPEC) | Redacted4 | 1.5944 | 8.1 | A | G | 0.0984 |
| 2017 | 2-Aug-17 | SAN LUIS OBISPO | LETTUCE, HEAD (ALL OR UNSPEC) | Redacted4 | 1.5944 | 8.1 | A | G | 0.0984 |
| 2017 | 26-May-17 | SAN LUIS OBISPO | LETTUCE, HEAD (ALL OR UNSPEC) | Redacted4 | 1.4763 | 7.5 | A | G | 0.0984 |
| 2017 | 7-Jul-17 | SAN LUIS OBISPO | LETTUCE, HEAD (ALL OR UNSPEC) | Redacted4 | 0.4921 | 2.5 | A | G | 0.0984 |
| 2017 | 24-May-17 | SAN LUIS OBISPO | LETTUCE, HEAD (ALL OR UNSPEC) | Redacted4 | 1.2401 | 6.3 | A | G | 0.0984 |
| 2017 | 2-Jun-17 | SAN LUIS OBISPO | LETTUCE, HEAD (ALL OR UNSPEC) | Redacted4 | 1.2401 | 6.3 | A | G | 0.0984 |
| 2017 | 9-Aug-17 | SAN LUIS OBISPO | LETTUCE, HEAD (ALL OR UNSPEC) | Redacted4 | 0.748 | 3.8 | A | G | 0.0984 |
| 2017 | 29-Jun-17 | SANTA BARBARA | LETTUCE, HEAD (ALL OR UNSPEC) | Redacted4 | 0.3937 | 2 | A | G | 0.0984 |
| 2017 | 26-May-17 | SANTA BARBARA | LETTUCE, HEAD (ALL OR UNSPEC) | Redacted4 | 0.3937 | 2 | A | G | 0.0984 |
| 2017 | 2-Jun-17 | SANTA BARBARA | LETTUCE, HEAD (ALL OR UNSPEC) | Redacted4 | 3.4433 | 17.46 | A | G | 0.0986 |
| 2017 | 5-Aug-17 | SANTA BARBARA | LETTUCE, HEAD (ALL OR UNSPEC) | Redacted4 | 2.9394 | 14.9 | A | G | 0.0986 |
| 2017 | 11-Jul-17 | SANTA BARBARA | LETTUCE, HEAD (ALL OR UNSPEC) | Redacted4 | 3.3593 | 17.01 | A | G | 0.0987 |
| 2017 | 3-Jul-17 | SANTA BARBARA | LETTUCE, HEAD (ALL OR UNSPEC) | Redacted4 | 3.4433 | 17.4 | A | G | 0.0989 |
| 2017 | 12-Jul-17 | SAN LUIS OBISPO | LETTUCE, HEAD (ALL OR UNSPEC) | Redacted4 | 1.4277 | 7.2 | A | G | 0.0991 |
| 2017 | 5-Jun-17 | SANTA BARBARA | LETTUCE, HEAD (ALL OR UNSPEC) | Redacted4 | 1.5957 | 8 | A | G | 0.0997 |
| 2017 | 29-Jun-17 | SANTA BARBARA | LETTUCE, HEAD (ALL OR UNSPEC) | Redacted4 | 0.9238 | 4.63 | A | G | 0.0998 |
| 2017 | 20-Jun-17 | SANTA BARBARA | LETTUCE, HEAD (ALL OR UNSPEC) | Redacted4 | 0.9238 | 4.63 | A | G | 0.0998 |
| 2017 | 4-Jun-17 | SANTA BARBARA | LETTUCE, HEAD (ALL OR UNSPEC) | Redacted4 | 0.9238 | 4.63 | A | G | 0.0998 |
| 2017 | 19-Jun-17 | SAN LUIS OBISPO | LETTUCE, HEAD (ALL OR UNSPEC) | Redacted4 | 1.5117 | 7.5 | A | G | 0.1008 |
| 2017 | 3-Jul-17 | SANTA BARBARA | LETTUCE, HEAD (ALL OR UNSPEC) | Redacted4 | 1.0078 | 5 | A | G | 0.1008 |
| 2017 | 1-Jun-17 | SANTA BARBARA | LETTUCE, HEAD (ALL OR UNSPEC) | Redacted4 | 1.1758 | 5.82 | A | G | 0.1010 |
| 2017 | 25-May-17 | SAN LUIS OBISPO | LETTUCE, HEAD (ALL OR UNSPEC) | Redacted4 | 0.8398 | 4.1 | A | G | 0.1024 |
| 2017 | 25-May-17 | SAN LUIS OBISPO | LETTUCE, HEAD (ALL OR UNSPEC) | Redacted4 | 0.6719 | 3.2 | A | G | 0.1050 |
| 2017 | 25-Aug-17 | SAN LUIS OBISPO | LETTUCE, HEAD (ALL OR UNSPEC) | Redacted4 | 0.4199 | 1.7 | A | G | 0.1235 |
| 2017 | 11-Jul-17 | SAN LUIS OBISPO | LETTUCE, HEAD (ALL OR UNSPEC) | Redacted4 | 0.6719 | 2.7 | A | G | 0.1244 |
| 2017 | 6-Sep-17 | SANTA BARBARA | LETTUCE, HEAD (ALL OR UNSPEC) | Redacted4 | 0.6719 | 2.7 | A | G | 0.1244 |
| 2017 | 4-Sep-17 | SAN LUIS OBISPO | LETTUCE, HEAD (ALL OR UNSPEC) | Redacted4 | 0.5039 | 2 | A | G | 0.1260 |
| 2017 | 26-Jul-17 | SANTA BARBARA | LETTUCE, HEAD (ALL OR UNSPEC) | Redacted4 | 0.5039 | 2 | A | G | 0.1260 |
| 2017 | 10-Jul-17 | SANTA BARBARA | LETTUCE, HEAD (ALL OR UNSPEC) | Redacted4 | 0.5039 | 2 | A | G | 0.1260 |
| 2017 | 10-Aug-17 | SANTA BARBARA | LETTUCE, HEAD (ALL OR UNSPEC) | Redacted4 | 0.5879 | 2.32 | A | G | 0.1267 |
| 2017 | 12-Jul-17 | SANTA BARBARA | LETTUCE, HEAD (ALL OR UNSPEC) | Redacted4 | 1.1758 | 4.63 | A | G | 0.1270 |
| 2017 | 30-Aug-17 | SAN LUIS OBISPO | LETTUCE, HEAD (ALL OR UNSPEC) | Redacted4 | 1.9316 | 7.5 | A | G | 0.1288 |
| 2017 | 29-Aug-17 | SANTA BARBARA | LETTUCE, HEAD (ALL OR UNSPEC) | Redacted4 | 1.5117 | 5.86 | A | G | 0.1290 |
| 2017 | 30-Aug-17 | SAN LUIS OBISPO | LETTUCE, HEAD (ALL OR UNSPEC) | Redacted4 | 1.3437 | 5.2 | A | G | 0.1292 |
| 2017 | 21-Jun-17 | SANTA BARBARA | LETTUCE, HEAD (ALL OR UNSPEC) | Redacted4 | 1.3437 | 5.2 | A | G | 0.1292 |
| 2017 | 1-Sep-17 | SANTA BARBARA | LETTUCE, HEAD (ALL OR UNSPEC) | Redacted4 | 1.3437 | 5.2 | A | G | 0.1292 |
| 2017 | 29-Jun-17 | SANTA BARBARA | LETTUCE, HEAD (ALL OR UNSPEC) | Redacted4 | 1.3437 | 5.2 | A | G | 0.1292 |
| 2017 | 12-Jul-17 | SAN LUIS OBISPO | LETTUCE, HEAD (ALL OR UNSPEC) | Redacted4 | 2.0156 | 7.8 | A | G | 0.1292 |
| 2017 | 15-Sep-17 | SAN LUIS OBISPO | LETTUCE, HEAD (ALL OR UNSPEC) | Redacted4 | 2.0156 | 7.8 | A | G | 0.1292 |
| 2017 | 5-Sep-17 | SAN LUIS OBISPO | LETTUCE, HEAD (ALL OR UNSPEC) | Redacted4 | 1.0078 | 3.9 | A | G | 0.1292 |
| 2017 | 4-Sep-17 | SANTA BARBARA | LETTUCE, HEAD (ALL OR UNSPEC) | Redacted4 | 2.0156 | 7.8 | A | G | 0.1292 |
| 2017 | 1-Jun-17 | SANTA BARBARA | LETTUCE, HEAD (ALL OR UNSPEC) | Redacted4 | 2.0996 | 8.1 | A | G | 0.1296 |
| 2017 | 6-Jul-17 | SANTA BARBARA | LETTUCE, HEAD (ALL OR UNSPEC) | Redacted4 | 3.3593 | 12.95 | A | G | 0.1297 |
| 2017 | 12-Jul-17 | SAN LUIS OBISPO | LETTUCE, HEAD (ALL OR UNSPEC) | Redacted4 | 1.4277 | 5.5 | A | G | 0.1298 |
| 2017 | 11-Jul-17 | SANTA BARBARA | LETTUCE, HEAD (ALL OR UNSPEC) | Redacted4 | 1.4277 | 5.5 | A | G | 0.1298 |
| 2017 | 7-Aug-17 | SANTA BARBARA | LETTUCE, HEAD (ALL OR UNSPEC) | Redacted4 | 1.4277 | 5.5 | A | G | 0.1298 |
| 2017 | 24-Jul-17 | SANTA BARBARA | LETTUCE, HEAD (ALL OR UNSPEC) | Redacted4 | 1.4277 | 5.5 | A | G | 0.1298 |
| 2017 | 30-Jun-17 | SANTA BARBARA | LETTUCE, HEAD (ALL OR UNSPEC) | Redacted4 | 1.5117 | 5.82 | A | G | 0.1299 |
| 2017 | 18-Jul-17 | SANTA BARBARA | LETTUCE, HEAD (ALL OR UNSPEC) | Redacted4 | 3.3593 | 12.92 | A | G | 0.1300 |
| 2017 | 22-Sep-17 | SANTA BARBARA | LETTUCE, HEAD (ALL OR UNSPEC) | Redacted4 | 4.0312 | 15.5 | A | G | 0.1300 |
| 2017 | 26-Jul-17 | SANTA BARBARA | LETTUCE, HEAD (ALL OR UNSPEC) | Redacted4 | 4.0312 | 15.5 | A | G | 0.1300 |
| 2017 | 13-Sep-17 | SANTA BARBARA | LETTUCE, HEAD (ALL OR UNSPEC) | Redacted4 | 4.0312 | 15.5 | A | G | 0.1300 |
| 2017 | 13-Jul-17 | SANTA BARBARA | LETTUCE, HEAD (ALL OR UNSPEC) | Redacted4 | 4.0312 | 15.5 | A | G | 0.1300 |
| 2017 | 25-Aug-17 | SAN LUIS OBISPO | LETTUCE, HEAD (ALL OR UNSPEC) | Redacted4 | 2.9394 | 11.3 | A | G | 0.1301 |
| 2017 | 25-Jul-17 | SANTA BARBARA | LETTUCE, HEAD (ALL OR UNSPEC) | Redacted4 | 4.7031 | 18.07 | A | G | 0.1301 |
| 2017 | 28-Jul-17 | SANTA BARBARA | LETTUCE, HEAD (ALL OR UNSPEC) | Redacted4 | 4.4511 | 17.1 | A | G | 0.1301 |
| 2017 | 4-Aug-17 | SANTA BARBARA | LETTUCE, HEAD (ALL OR UNSPEC) | Redacted4 | 4.4511 | 17.1 | A | G | 0.1301 |
| 2017 | 19-Jun-17 | SANTA BARBARA | LETTUCE, HEAD (ALL OR UNSPEC) | Redacted4 | 4.5351 | 17.4 | A | G | 0.1303 |
| 2017 | 25-Aug-17 | SAN LUIS OBISPO | LETTUCE, HEAD (ALL OR UNSPEC) | Redacted4 | 1.9316 | 7.4 | A | G | 0.1305 |
| 2017 | 15-Aug-17 | SAN LUIS OBISPO | LETTUCE, HEAD (ALL OR UNSPEC) | Redacted4 | 1.9316 | 7.4 | A | G | 0.1305 |
| 2017 | 1-Jun-17 | SANTA BARBARA | LETTUCE, HEAD (ALL OR UNSPEC) | Redacted4 | 1.9316 | 7.4 | A | G | 0.1305 |
| 2017 | 8-Sep-17 | SAN LUIS OBISPO | LETTUCE, HEAD (ALL OR UNSPEC) | Redacted4 | 2.3515 | 9 | A | G | 0.1306 |
| 2017 | 1-Sep-17 | SAN LUIS OBISPO | LETTUCE, HEAD (ALL OR UNSPEC) | Redacted4 | 2.3515 | 9 | A | G | 0.1306 |
| 2017 | 11-Oct-17 | KERN | LETTUCE, HEAD (ALL OR UNSPEC) | Redacted4 | 4.7031 | 18 | A | A | 0.1306 |
| 2017 | 17-Oct-17 | KERN | LETTUCE, HEAD (ALL OR UNSPEC) | Redacted4 | 4.7031 | 18 | A | A | 0.1306 |
| 2017 | 4-Oct-17 | KERN | LETTUCE, HEAD (ALL OR UNSPEC) | Redacted4 | 4.7031 | 18 | A | A | 0.1306 |
| 2017 | 7-Aug-17 | SANTA BARBARA | LETTUCE, HEAD (ALL OR UNSPEC) | Redacted4 | 4.4511 | 17.01 | A | G | 0.1308 |
| 2017 | 1-Sep-17 | SAN LUIS OBISPO | LETTUCE, HEAD (ALL OR UNSPEC) | Redacted4 | 2.0156 | 7.7 | A | G | 0.1309 |
| 2017 | 8-Sep-17 | SAN LUIS OBISPO | LETTUCE, HEAD (ALL OR UNSPEC) | Redacted4 | 2.0156 | 7.7 | A | G | 0.1309 |
| 2017 | 15-Sep-17 | SAN LUIS OBISPO | LETTUCE, HEAD (ALL OR UNSPEC) | Redacted4 | 2.0156 | 7.7 | A | G | 0.1309 |
| 2017 | 10-Aug-17 | SANTA BARBARA | LETTUCE, HEAD (ALL OR UNSPEC) | Redacted4 | 4.4511 | 17 | A | G | 0.1309 |
| 2017 | 25-Aug-17 | SANTA BARBARA | LETTUCE, HEAD (ALL OR UNSPEC) | Redacted4 | 4.4511 | 17 | A | G | 0.1309 |
| 2017 | 6-Sep-17 | SANTA BARBARA | LETTUCE, HEAD (ALL OR UNSPEC) | Redacted4 | 4.4511 | 17 | A | G | 0.1309 |
| 2017 | 10-Oct-17 | KERN | LETTUCE, HEAD (ALL OR UNSPEC) | Redacted4 | 10.7498 | 41 | A | A | 0.1311 |
| 2017 | 10-Jul-17 | SANTA BARBARA | LETTUCE, HEAD (ALL OR UNSPEC) | Redacted4 | 0.2624 | 1 | A | G | 0.1312 |
| 2017 | 11-Jul-17 | SAN LUIS OBISPO | LETTUCE, HEAD (ALL OR UNSPEC) | Redacted4 | 0.4199 | 1.6 | A | G | 0.1312 |
| 2017 | 20-Jul-17 | SAN LUIS OBISPO | LETTUCE, HEAD (ALL OR UNSPEC) | Redacted4 | 0.4199 | 1.6 | A | G | 0.1312 |
| 2017 | 30-Jun-17 | SANTA BARBARA | LETTUCE, HEAD (ALL OR UNSPEC) | Redacted4 | 1.2597 | 4.8 | A | G | 0.1312 |
| 2017 | 22-Jun-17 | SANTA BARBARA | LETTUCE, HEAD (ALL OR UNSPEC) | Redacted4 | 1.2597 | 4.8 | A | G | 0.1312 |
| 2017 | 12-Jul-17 | SANTA BARBARA | LETTUCE, HEAD (ALL OR UNSPEC) | Redacted4 | 1.2597 | 4.8 | A | G | 0.1312 |
| 2017 | 8-Jul-17 | SANTA BARBARA | LETTUCE, HEAD (ALL OR UNSPEC) | Redacted4 | 1.5274 | 5.82 | A | G | 0.1312 |
| 2017 | 14-Jul-17 | SAN LUIS OBISPO | LETTUCE, HEAD (ALL OR UNSPEC) | Redacted4 | 0.6561 | 2.5 | A | G | 0.1312 |
| 2017 | 7-Sep-17 | SAN LUIS OBISPO | LETTUCE, HEAD (ALL OR UNSPEC) | Redacted4 | 0.6561 | 2.5 | A | G | 0.1312 |
| 2017 | 22-Aug-17 | SAN LUIS OBISPO | LETTUCE, HEAD (ALL OR UNSPEC) | Redacted4 | 0.6561 | 2.5 | A | G | 0.1312 |
| 2017 | 27-Jun-17 | SAN LUIS OBISPO | LETTUCE, HEAD (ALL OR UNSPEC) | Redacted4 | 0.6561 | 2.5 | A | G | 0.1312 |
| 2017 | 15-Jul-17 | SANTA BARBARA | LETTUCE, HEAD (ALL OR UNSPEC) | Redacted4 | 1.3122 | 5 | A | G | 0.1312 |
| 2017 | 21-Aug-17 | SAN LUIS OBISPO | LETTUCE, HEAD (ALL OR UNSPEC) | Redacted4 | 2.0208 | 7.7 | A | G | 0.1312 |
| 2017 | 22-Aug-17 | SAN LUIS OBISPO | LETTUCE, HEAD (ALL OR UNSPEC) | Redacted4 | 2.0208 | 7.7 | A | G | 0.1312 |
| 2017 | 8-Jul-17 | SANTA BARBARA | LETTUCE, HEAD (ALL OR UNSPEC) | Redacted4 | 1.9132 | 7.29 | A | G | 0.1312 |
| 2017 | 10-Jul-17 | SANTA BARBARA | LETTUCE, HEAD (ALL OR UNSPEC) | Redacted4 | 2.4827 | 9.46 | A | G | 0.1312 |
| 2017 | 6-Sep-17 | SAN LUIS OBISPO | LETTUCE, HEAD (ALL OR UNSPEC) | Redacted4 | 1.3647 | 5.2 | A | G | 0.1312 |
| 2017 | 6-Jun-17 | SANTA BARBARA | LETTUCE, HEAD (ALL OR UNSPEC) | Redacted4 | 1.3647 | 5.2 | A | G | 0.1312 |
| 2017 | 13-Sep-17 | SANTA BARBARA | LETTUCE, HEAD (ALL OR UNSPEC) | Redacted4 | 1.3647 | 5.2 | A | G | 0.1312 |
| 2017 | 27-Jun-17 | SAN LUIS OBISPO | LETTUCE, HEAD (ALL OR UNSPEC) | Redacted4 | 2.0733 | 7.9 | A | G | 0.1312 |
| 2017 | 25-Aug-17 | SAN LUIS OBISPO | LETTUCE, HEAD (ALL OR UNSPEC) | Redacted4 | 2.0733 | 7.9 | A | G | 0.1312 |
| 2017 | 22-Aug-17 | SAN LUIS OBISPO | LETTUCE, HEAD (ALL OR UNSPEC) | Redacted4 | 1.3857 | 5.28 | A | G | 0.1312 |
| 2017 | 8-Sep-17 | SAN LUIS OBISPO | LETTUCE, HEAD (ALL OR UNSPEC) | Redacted4 | 3.4905 | 13.3 | A | G | 0.1312 |
| 2017 | 25-Aug-17 | SAN LUIS OBISPO | LETTUCE, HEAD (ALL OR UNSPEC) | Redacted4 | 3.4905 | 13.3 | A | G | 0.1312 |
| 2017 | 29-Jun-17 | SANTA BARBARA | LETTUCE, HEAD (ALL OR UNSPEC) | Redacted4 | 3.4905 | 13.3 | A | G | 0.1312 |
| 2017 | 4-Aug-17 | SANTA BARBARA | LETTUCE, HEAD (ALL OR UNSPEC) | Redacted4 | 3.4905 | 13.3 | A | G | 0.1312 |
| 2017 | 22-Aug-17 | SANTA BARBARA | LETTUCE, HEAD (ALL OR UNSPEC) | Redacted4 | 3.8448 | 14.65 | A | G | 0.1312 |
| 2017 | 11-Aug-17 | SAN LUIS OBISPO | LETTUCE, HEAD (ALL OR UNSPEC) | Redacted4 | 2.1258 | 8.1 | A | G | 0.1312 |
| 2017 | 20-Jul-17 | SAN LUIS OBISPO | LETTUCE, HEAD (ALL OR UNSPEC) | Redacted4 | 1.8896 | 7.2 | A | G | 0.1312 |
| 2017 | 30-Jun-17 | SAN LUIS OBISPO | LETTUCE, HEAD (ALL OR UNSPEC) | Redacted4 | 0.7086 | 2.7 | A | G | 0.1312 |
| 2017 | 19-Jul-17 | SAN LUIS OBISPO | LETTUCE, HEAD (ALL OR UNSPEC) | Redacted4 | 0.7086 | 2.7 | A | G | 0.1312 |
| 2017 | 18-Aug-17 | SAN LUIS OBISPO | LETTUCE, HEAD (ALL OR UNSPEC) | Redacted4 | 2.1258 | 8.1 | A | G | 0.1312 |
| 2017 | 31-Jul-17 | SAN LUIS OBISPO | LETTUCE, HEAD (ALL OR UNSPEC) | Redacted4 | 0.4724 | 1.8 | A | G | 0.1312 |
| 2017 | 4-Aug-17 | SAN LUIS OBISPO | LETTUCE, HEAD (ALL OR UNSPEC) | Redacted4 | 2.8344 | 10.8 | A | G | 0.1312 |
| 2017 | 12-Sep-17 | SAN LUIS OBISPO | LETTUCE, HEAD (ALL OR UNSPEC) | Redacted4 | 1.6534 | 6.3 | A | G | 0.1312 |
| 2017 | 31-Jul-17 | SAN LUIS OBISPO | LETTUCE, HEAD (ALL OR UNSPEC) | Redacted4 | 1.8896 | 7.2 | A | G | 0.1312 |
| 2017 | 26-Jul-17 | SAN LUIS OBISPO | LETTUCE, HEAD (ALL OR UNSPEC) | Redacted4 | 2.8344 | 10.8 | A | G | 0.1312 |
| 2017 | 20-Jul-17 | SAN LUIS OBISPO | LETTUCE, HEAD (ALL OR UNSPEC) | Redacted4 | 0.4724 | 1.8 | A | G | 0.1312 |
| 2017 | 14-Jul-17 | SAN LUIS OBISPO | LETTUCE, HEAD (ALL OR UNSPEC) | Redacted4 | 2.1258 | 8.1 | A | G | 0.1312 |
| 2017 | 23-Aug-17 | SANTA BARBARA | LETTUCE, HEAD (ALL OR UNSPEC) | Redacted4 | 0.7086 | 2.7 | A | G | 0.1312 |
| 2017 | 22-Sep-17 | SANTA BARBARA | LETTUCE, HEAD (ALL OR UNSPEC) | Redacted4 | 4.0154 | 15.3 | A | G | 0.1312 |
| 2017 | 8-Aug-17 | SANTA BARBARA | LETTUCE, HEAD (ALL OR UNSPEC) | Redacted4 | 0.7086 | 2.7 | A | G | 0.1312 |
| 2017 | 13-Sep-17 | SANTA BARBARA | LETTUCE, HEAD (ALL OR UNSPEC) | Redacted4 | 2.1258 | 8.1 | A | G | 0.1312 |
| 2017 | 12-Sep-17 | SAN LUIS OBISPO | LETTUCE, HEAD (ALL OR UNSPEC) | Redacted4 | 3.543 | 13.5 | A | G | 0.1312 |
| 2017 | 25-Aug-17 | SANTA BARBARA | LETTUCE, HEAD (ALL OR UNSPEC) | Redacted4 | 3.543 | 13.5 | A | G | 0.1312 |
| 2017 | 10-Jul-17 | SANTA BARBARA | LETTUCE, HEAD (ALL OR UNSPEC) | Redacted4 | 3.9288 | 14.97 | A | G | 0.1312 |
| 2017 | 13-Jul-17 | SANTA BARBARA | LETTUCE, HEAD (ALL OR UNSPEC) | Redacted4 | 3.3593 | 12.8 | A | G | 0.1312 |
| 2017 | 7-Sep-17 | SAN LUIS OBISPO | LETTUCE, HEAD (ALL OR UNSPEC) | Redacted4 | 2.6507 | 10.1 | A | G | 0.1312 |
| 2017 | 21-Aug-17 | SAN LUIS OBISPO | LETTUCE, HEAD (ALL OR UNSPEC) | Redacted4 | 2.1783 | 8.3 | A | G | 0.1312 |
| 2017 | 26-Jul-17 | SAN LUIS OBISPO | LETTUCE, HEAD (ALL OR UNSPEC) | Redacted4 | 1.9421 | 7.4 | A | G | 0.1312 |
| 2017 | 4-Aug-17 | SAN LUIS OBISPO | LETTUCE, HEAD (ALL OR UNSPEC) | Redacted4 | 1.9421 | 7.4 | A | G | 0.1312 |
| 2017 | 13-Sep-17 | SANTA BARBARA | LETTUCE, HEAD (ALL OR UNSPEC) | Redacted4 | 1.9421 | 7.4 | A | G | 0.1312 |
| 2017 | 5-Aug-17 | SANTA BARBARA | LETTUCE, HEAD (ALL OR UNSPEC) | Redacted4 | 4.7424 | 18.07 | A | G | 0.1312 |
| 2017 | 22-Jun-17 | SAN LUIS OBISPO | LETTUCE, HEAD (ALL OR UNSPEC) | Redacted4 | 1.7059 | 6.5 | A | G | 0.1312 |
| 2017 | 16-Sep-17 | SANTA BARBARA | LETTUCE, HEAD (ALL OR UNSPEC) | Redacted4 | 4.4196 | 16.84 | A | G | 0.1312 |
| 2017 | 2-Aug-17 | SANTA BARBARA | LETTUCE, HEAD (ALL OR UNSPEC) | Redacted4 | 4.4301 | 16.88 | A | G | 0.1312 |
| 2017 | 10-Aug-17 | SANTA BARBARA | LETTUCE, HEAD (ALL OR UNSPEC) | Redacted4 | 4.4301 | 16.88 | A | G | 0.1312 |
| 2017 | 23-Aug-17 | SANTA BARBARA | LETTUCE, HEAD (ALL OR UNSPEC) | Redacted4 | 4.4301 | 16.88 | A | G | 0.1312 |
| 2017 | 27-Aug-17 | SANTA BARBARA | LETTUCE, HEAD (ALL OR UNSPEC) | Redacted4 | 4.934 | 18.8 | A | G | 0.1312 |
| 2017 | 7-Sep-17 | SANTA BARBARA | LETTUCE, HEAD (ALL OR UNSPEC) | Redacted4 | 4.4616 | 17 | A | G | 0.1312 |
| 2017 | 21-Aug-17 | SAN LUIS OBISPO | LETTUCE, HEAD (ALL OR UNSPEC) | Redacted4 | 0.9973 | 3.8 | A | G | 0.1312 |
| 2017 | 11-Aug-17 | SAN LUIS OBISPO | LETTUCE, HEAD (ALL OR UNSPEC) | Redacted4 | 1.7584 | 6.7 | A | G | 0.1312 |
| 2017 | 24-Jul-17 | SAN LUIS OBISPO | LETTUCE, HEAD (ALL OR UNSPEC) | Redacted4 | 1.7584 | 6.7 | A | G | 0.1312 |
| 2017 | 14-Aug-17 | SANTA BARBARA | LETTUCE, HEAD (ALL OR UNSPEC) | Redacted4 | 3.2806 | 12.5 | A | G | 0.1312 |
| 2017 | 13-Sep-17 | SANTA BARBARA | LETTUCE, HEAD (ALL OR UNSPEC) | Redacted4 | 2.0471 | 7.8 | A | G | 0.1312 |
| 2017 | 21-Sep-17 | SANTA BARBARA | LETTUCE, HEAD (ALL OR UNSPEC) | Redacted4 | 2.0471 | 7.8 | A | G | 0.1312 |
| 2017 | 15-Sep-17 | SANTA BARBARA | LETTUCE, HEAD (ALL OR UNSPEC) | Redacted4 | 4.6296 | 17.64 | A | G | 0.1312 |
| 2017 | 3-Jul-17 | SAN LUIS OBISPO | LETTUCE, HEAD (ALL OR UNSPEC) | Redacted4 | 1.8109 | 6.9 | A | G | 0.1312 |
| 2017 | 14-Jul-17 | SANTA BARBARA | LETTUCE, HEAD (ALL OR UNSPEC) | Redacted4 | 4.4354 | 16.9 | A | G | 0.1312 |
| 2017 | 13-Sep-17 | SANTA BARBARA | LETTUCE, HEAD (ALL OR UNSPEC) | Redacted4 | 2.6245 | 10 | A | G | 0.1312 |
| 2017 | 20-Aug-17 | SAN LUIS OBISPO | LETTUCE, HEAD (ALL OR UNSPEC) | Redacted4 | 0.5249 | 2 | A | G | 0.1312 |
| 2017 | 21-Jul-17 | SAN LUIS OBISPO | LETTUCE, HEAD (ALL OR UNSPEC) | Redacted4 | 3.1494 | 12 | A | G | 0.1312 |
| 2017 | 18-Aug-17 | SAN LUIS OBISPO | LETTUCE, HEAD (ALL OR UNSPEC) | Redacted4 | 1.5747 | 6 | A | G | 0.1312 |
| 2017 | 19-Jul-17 | SAN LUIS OBISPO | LETTUCE, HEAD (ALL OR UNSPEC) | Redacted4 | 3.1494 | 12 | A | G | 0.1312 |
| 2017 | 3-Jul-17 | SAN LUIS OBISPO | LETTUCE, HEAD (ALL OR UNSPEC) | Redacted4 | 3.1494 | 12 | A | G | 0.1312 |
| 2017 | 10-Nov-17 | SAN LUIS OBISPO | LETTUCE, HEAD (ALL OR UNSPEC) | Redacted4 | 2.0996 | 8 | A | G | 0.1312 |
| 2017 | 21-Sep-17 | SANTA BARBARA | LETTUCE, HEAD (ALL OR UNSPEC) | Redacted4 | 1.0498 | 4 | A | G | 0.1312 |
| 2017 | 6-Jun-17 | SANTA BARBARA | LETTUCE, HEAD (ALL OR UNSPEC) | Redacted4 | 0.5249 | 2 | A | G | 0.1312 |
| 2017 | 13-Sep-17 | SANTA BARBARA | LETTUCE, HEAD (ALL OR UNSPEC) | Redacted4 | 1.0498 | 4 | A | G | 0.1312 |
| 2017 | 14-Jul-17 | SANTA BARBARA | LETTUCE, HEAD (ALL OR UNSPEC) | Redacted4 | 3.4171 | 13.02 | A | G | 0.1312 |
| 2017 | 22-Aug-17 | SANTA BARBARA | LETTUCE, HEAD (ALL OR UNSPEC) | Redacted4 | 2.0025 | 7.63 | A | G | 0.1312 |
| 2017 | 21-Aug-17 | SAN LUIS OBISPO | LETTUCE, HEAD (ALL OR UNSPEC) | Redacted4 | 2.1521 | 8.2 | A | G | 0.1312 |
| 2017 | 11-Aug-17 | SAN LUIS OBISPO | LETTUCE, HEAD (ALL OR UNSPEC) | Redacted4 | 2.1521 | 8.2 | A | G | 0.1312 |
| 2017 | 8-Sep-17 | SAN LUIS OBISPO | LETTUCE, HEAD (ALL OR UNSPEC) | Redacted4 | 2.9657 | 11.3 | A | G | 0.1312 |
| 2017 | 3-Aug-17 | SAN LUIS OBISPO | LETTUCE, HEAD (ALL OR UNSPEC) | Redacted4 | 1.9159 | 7.3 | A | G | 0.1312 |
| 2017 | 15-Aug-17 | SAN LUIS OBISPO | LETTUCE, HEAD (ALL OR UNSPEC) | Redacted4 | 1.9159 | 7.3 | A | G | 0.1312 |
| 2017 | 31-Jul-17 | SANTA BARBARA | LETTUCE, HEAD (ALL OR UNSPEC) | Redacted4 | 1.6797 | 6.4 | A | G | 0.1312 |
| 2017 | 7-Aug-17 | SANTA BARBARA | LETTUCE, HEAD (ALL OR UNSPEC) | Redacted4 | 1.6797 | 6.4 | A | G | 0.1312 |
| 2017 | 21-Aug-17 | SAN LUIS OBISPO | LETTUCE, HEAD (ALL OR UNSPEC) | Redacted4 | 1.9684 | 7.5 | A | G | 0.1312 |
| 2017 | 7-Sep-17 | SAN LUIS OBISPO | LETTUCE, HEAD (ALL OR UNSPEC) | Redacted4 | 1.9684 | 7.5 | A | G | 0.1312 |
| 2017 | 6-Jun-17 | SAN LUIS OBISPO | LETTUCE, HEAD (ALL OR UNSPEC) | Redacted4 | 1.9684 | 7.5 | A | G | 0.1312 |
| 2017 | 11-Aug-17 | SAN LUIS OBISPO | LETTUCE, HEAD (ALL OR UNSPEC) | Redacted4 | 1.9684 | 7.5 | A | G | 0.1312 |
| 2017 | 10-Jul-17 | SANTA BARBARA | LETTUCE, HEAD (ALL OR UNSPEC) | Redacted4 | 1.7217 | 6.56 | A | G | 0.1312 |
| 2017 | 27-Jul-17 | SAN LUIS OBISPO | LETTUCE, HEAD (ALL OR UNSPEC) | Redacted4 | 1.4435 | 5.5 | A | G | 0.1312 |
| 2017 | 4-Aug-17 | SAN LUIS OBISPO | LETTUCE, HEAD (ALL OR UNSPEC) | Redacted4 | 1.1548 | 4.4 | A | G | 0.1312 |
| 2017 | 26-Jul-17 | SAN LUIS OBISPO | LETTUCE, HEAD (ALL OR UNSPEC) | Redacted4 | 1.1548 | 4.4 | A | G | 0.1312 |
| 2017 | 20-Jul-17 | SAN LUIS OBISPO | LETTUCE, HEAD (ALL OR UNSPEC) | Redacted4 | 1.4435 | 5.5 | A | G | 0.1312 |
| 2017 | 30-Jun-17 | SANTA BARBARA | LETTUCE, HEAD (ALL OR UNSPEC) | Redacted4 | 1.4435 | 5.5 | A | G | 0.1312 |
| 2017 | 22-Aug-17 | SANTA BARBARA | LETTUCE, HEAD (ALL OR UNSPEC) | Redacted4 | 0.6089 | 2.32 | A | G | 0.1312 |
| 2017 | 26-Jul-17 | SANTA BARBARA | LETTUCE, HEAD (ALL OR UNSPEC) | Redacted4 | 4.4511 | 16.94 | A | G | 0.1314 |
| 2017 | 27-Jul-17 | SANTA BARBARA | LETTUCE, HEAD (ALL OR UNSPEC) | Redacted4 | 2.6035 | 9.9 | A | G | 0.1315 |
| 2017 | 11-Nov-17 | KERN | LETTUCE, HEAD (ALL OR UNSPEC) | Redacted4 | 3.9472 | 15 | A | A | 0.1316 |
| 2017 | 22-Sep-17 | SANTA BARBARA | LETTUCE, HEAD (ALL OR UNSPEC) | Redacted4 | 0.7558 | 2.87 | A | G | 0.1317 |
| 2017 | 3-Jul-17 | SANTA BARBARA | LETTUCE, HEAD (ALL OR UNSPEC) | Redacted4 | 4.4511 | 16.9 | A | G | 0.1317 |
| 2017 | 11-Jul-17 | SANTA BARBARA | LETTUCE, HEAD (ALL OR UNSPEC) | Redacted4 | 4.4511 | 16.9 | A | G | 0.1317 |
| 2017 | 23-Sep-17 | SANTA BARBARA | LETTUCE, HEAD (ALL OR UNSPEC) | Redacted4 | 1.3437 | 5.1 | A | G | 0.1317 |
| 2017 | 1-Sep-17 | SANTA BARBARA | LETTUCE, HEAD (ALL OR UNSPEC) | Redacted4 | 4.4511 | 16.89 | A | G | 0.1318 |
| 2017 | 12-Sep-17 | SANTA BARBARA | LETTUCE, HEAD (ALL OR UNSPEC) | Redacted4 | 4.4511 | 16.89 | A | G | 0.1318 |
| 2017 | 29-Aug-17 | SANTA BARBARA | LETTUCE, HEAD (ALL OR UNSPEC) | Redacted4 | 4.955 | 18.8 | A | G | 0.1318 |
| 2017 | 6-Sep-17 | SANTA BARBARA | LETTUCE, HEAD (ALL OR UNSPEC) | Redacted4 | 4.4511 | 16.88 | A | G | 0.1318 |
| 2017 | 11-Aug-17 | SANTA BARBARA | LETTUCE, HEAD (ALL OR UNSPEC) | Redacted4 | 3.8632 | 14.65 | A | G | 0.1318 |
| 2017 | 18-Sep-17 | SAN LUIS OBISPO | LETTUCE, HEAD (ALL OR UNSPEC) | Redacted4 | 1.8476 | 7 | A | G | 0.1320 |
| 2017 | 6-Jul-17 | SANTA BARBARA | LETTUCE, HEAD (ALL OR UNSPEC) | Redacted4 | 4.6191 | 17.46 | A | G | 0.1323 |
| 2017 | 12-Jul-17 | SANTA BARBARA | LETTUCE, HEAD (ALL OR UNSPEC) | Redacted4 | 4.6191 | 17.46 | A | G | 0.1323 |
| 2017 | 25-Aug-17 | SAN LUIS OBISPO | LETTUCE, HEAD (ALL OR UNSPEC) | Redacted4 | 1.9316 | 7.3 | A | G | 0.1323 |
| 2017 | 6-Jul-17 | SANTA BARBARA | LETTUCE, HEAD (ALL OR UNSPEC) | Redacted4 | 4.6191 | 17.45 | A | G | 0.1324 |
| 2017 | 18-Jul-17 | SANTA BARBARA | LETTUCE, HEAD (ALL OR UNSPEC) | Redacted4 | 4.6191 | 17.45 | A | G | 0.1324 |
| 2017 | 29-Aug-17 | SANTA BARBARA | LETTUCE, HEAD (ALL OR UNSPEC) | Redacted4 | 3.9472 | 14.9 | A | G | 0.1325 |
| 2017 | 31-Jul-17 | SANTA BARBARA | LETTUCE, HEAD (ALL OR UNSPEC) | Redacted4 | 2.7714 | 10.46 | A | G | 0.1325 |
| 2017 | 14-Jul-17 | SANTA BARBARA | LETTUCE, HEAD (ALL OR UNSPEC) | Redacted4 | 2.7714 | 10.46 | A | G | 0.1325 |
| 2017 | 21-Jul-17 | SANTA BARBARA | LETTUCE, HEAD (ALL OR UNSPEC) | Redacted4 | 2.7714 | 10.46 | A | G | 0.1325 |
| 2017 | 7-Aug-17 | SANTA BARBARA | LETTUCE, HEAD (ALL OR UNSPEC) | Redacted4 | 2.7714 | 10.46 | A | G | 0.1325 |
| 2017 | 30-Jun-17 | SANTA BARBARA | LETTUCE, HEAD (ALL OR UNSPEC) | Redacted4 | 1.9316 | 7.29 | A | G | 0.1325 |
| 2017 | 24-Jul-17 | SANTA BARBARA | LETTUCE, HEAD (ALL OR UNSPEC) | Redacted4 | 3.5273 | 13.3 | A | G | 0.1326 |
| 2017 | 17-Jul-17 | SANTA BARBARA | LETTUCE, HEAD (ALL OR UNSPEC) | Redacted4 | 1.5117 | 5.7 | A | G | 0.1326 |
| 2017 | 10-Jul-17 | SANTA BARBARA | LETTUCE, HEAD (ALL OR UNSPEC) | Redacted4 | 3.5273 | 13.3 | A | G | 0.1326 |
| 2017 | 6-Jul-17 | SANTA BARBARA | LETTUCE, HEAD (ALL OR UNSPEC) | Redacted4 | 1.5117 | 5.7 | A | G | 0.1326 |
| 2017 | 8-Sep-17 | SAN LUIS OBISPO | LETTUCE, HEAD (ALL OR UNSPEC) | Redacted4 | 2.0156 | 7.6 | A | G | 0.1326 |
| 2017 | 21-Aug-17 | SAN LUIS OBISPO | LETTUCE, HEAD (ALL OR UNSPEC) | Redacted4 | 2.0156 | 7.6 | A | G | 0.1326 |
| 2017 | 19-Jun-17 | SAN LUIS OBISPO | LETTUCE, HEAD (ALL OR UNSPEC) | Redacted4 | 2.0996 | 7.9 | A | G | 0.1329 |
| 2017 | 5-Sep-17 | SAN LUIS OBISPO | LETTUCE, HEAD (ALL OR UNSPEC) | Redacted4 | 2.0996 | 7.9 | A | G | 0.1329 |
| 2017 | 15-Sep-17 | SAN LUIS OBISPO | LETTUCE, HEAD (ALL OR UNSPEC) | Redacted4 | 2.0996 | 7.9 | A | G | 0.1329 |
| 2017 | 1-Sep-17 | SAN LUIS OBISPO | LETTUCE, HEAD (ALL OR UNSPEC) | Redacted4 | 1.5957 | 6 | A | G | 0.1330 |
| 2017 | 25-Aug-17 | SAN LUIS OBISPO | LETTUCE, HEAD (ALL OR UNSPEC) | Redacted4 | 1.5957 | 6 | A | G | 0.1330 |
| 2017 | 25-Aug-17 | SAN LUIS OBISPO | LETTUCE, HEAD (ALL OR UNSPEC) | Redacted4 | 2.6875 | 10.1 | A | G | 0.1330 |
| 2017 | 1-Aug-17 | SAN LUIS OBISPO | LETTUCE, HEAD (ALL OR UNSPEC) | Redacted4 | 2.1836 | 8.2 | A | G | 0.1331 |
| 2017 | 21-Jun-17 | SANTA BARBARA | LETTUCE, HEAD (ALL OR UNSPEC) | Redacted4 | 1.0918 | 4.1 | A | G | 0.1331 |
| 2017 | 30-Jun-17 | SANTA BARBARA | LETTUCE, HEAD (ALL OR UNSPEC) | Redacted4 | 2.7714 | 10.4 | A | G | 0.1332 |
| 2017 | 22-Jun-17 | SANTA BARBARA | LETTUCE, HEAD (ALL OR UNSPEC) | Redacted4 | 2.7714 | 10.4 | A | G | 0.1332 |
| 2017 | 12-Jul-17 | SANTA BARBARA | LETTUCE, HEAD (ALL OR UNSPEC) | Redacted4 | 2.7714 | 10.4 | A | G | 0.1332 |
| 2017 | 7-Sep-17 | SANTA BARBARA | LETTUCE, HEAD (ALL OR UNSPEC) | Redacted4 | 0.6719 | 2.52 | A | G | 0.1333 |
| 2017 | 30-Aug-17 | SANTA BARBARA | LETTUCE, HEAD (ALL OR UNSPEC) | Redacted4 | 2.2675 | 8.5 | A | G | 0.1334 |
| 2017 | 11-Jul-17 | SAN LUIS OBISPO | LETTUCE, HEAD (ALL OR UNSPEC) | Redacted4 | 1.1758 | 4.4 | A | G | 0.1336 |
| 2017 | 29-Aug-17 | SANTA BARBARA | LETTUCE, HEAD (ALL OR UNSPEC) | Redacted4 | 1.2597 | 4.7 | A | G | 0.1340 |
| 2017 | 5-Aug-17 | SAN LUIS OBISPO | LETTUCE, HEAD (ALL OR UNSPEC) | Redacted4 | 1.9316 | 7.2 | A | G | 0.1341 |
| 2017 | 30-Aug-17 | SAN LUIS OBISPO | LETTUCE, HEAD (ALL OR UNSPEC) | Redacted4 | 0.6719 | 2.5 | A | G | 0.1344 |
| 2017 | 22-Jul-17 | SANTA BARBARA | LETTUCE, HEAD (ALL OR UNSPEC) | Redacted4 | 1.7636 | 6.56 | A | G | 0.1344 |
| 2017 | 15-Sep-17 | SAN LUIS OBISPO | LETTUCE, HEAD (ALL OR UNSPEC) | Redacted4 | 1.0918 | 4 | A | G | 0.1365 |
| 2017 | 5-Aug-17 | SAN LUIS OBISPO | LETTUCE, HEAD (ALL OR UNSPEC) | Redacted4 | 1.0918 | 4 | A | G | 0.1365 |
| 2017 | 5-Sep-17 | SAN LUIS OBISPO | LETTUCE, HEAD (ALL OR UNSPEC) | Redacted4 | 1.0918 | 4 | A | G | 0.1365 |
| 2017 | 30-Jun-17 | SANTA BARBARA | LETTUCE, HEAD (ALL OR UNSPEC) | Redacted4 | 1.0918 | 4 | A | G | 0.1365 |
| 2017 | 1-Sep-17 | SANTA BARBARA | LETTUCE, HEAD (ALL OR UNSPEC) | Redacted4 | 1.0918 | 4 | A | G | 0.1365 |
| 2017 | 9-Aug-17 | SAN LUIS OBISPO | LETTUCE, HEAD (ALL OR UNSPEC) | Redacted4 | 1.9316 | 6 | A | G | 0.1610 |
| 2017 | 9-Aug-17 | SAN LUIS OBISPO | LETTUCE, HEAD (ALL OR UNSPEC) | Redacted4 | 1.2597 | 3.9 | A | G | 0.1615 |
| 2017 | 9-Aug-17 | SAN LUIS OBISPO | LETTUCE, HEAD (ALL OR UNSPEC) | Redacted4 | 1.4277 | 4.4 | A | G | 0.1622 |
| 2017 | 9-Aug-17 | SANTA BARBARA | LETTUCE, HEAD (ALL OR UNSPEC) | Redacted4 | 5.5429 | 17.01 | A | G | 0.1629 |
| 2017 | 15-Aug-17 | SAN LUIS OBISPO | LETTUCE, HEAD (ALL OR UNSPEC) | Redacted4 | 3.6953 | 11.3 | A | G | 0.1635 |
| 2017 | 3-Aug-17 | SANTA BARBARA | LETTUCE, HEAD (ALL OR UNSPEC) | Redacted4 | 5.5429 | 16.94 | A | G | 0.1636 |
| 2017 | 16-Aug-17 | SANTA BARBARA | LETTUCE, HEAD (ALL OR UNSPEC) | Redacted4 | 1.9224 | 5.86 | A | G | 0.1640 |
| 2017 | 17-Jul-17 | SAN LUIS OBISPO | LETTUCE, HEAD (ALL OR UNSPEC) | Redacted4 | 3.543 | 10.8 | A | G | 0.1640 |
| 2017 | 7-Aug-17 | SANTA BARBARA | LETTUCE, HEAD (ALL OR UNSPEC) | Redacted4 | 5.0849 | 15.5 | A | G | 0.1640 |
| 2017 | 17-Aug-17 | SAN LUIS OBISPO | LETTUCE, HEAD (ALL OR UNSPEC) | Redacted4 | 0.5577 | 1.7 | A | G | 0.1640 |
| 2017 | 2-Aug-17 | SAN LUIS OBISPO | LETTUCE, HEAD (ALL OR UNSPEC) | Redacted4 | 2.198 | 6.7 | A | G | 0.1640 |
| 2017 | 9-Aug-17 | SANTA BARBARA | LETTUCE, HEAD (ALL OR UNSPEC) | Redacted4 | 2.0996 | 6.4 | A | G | 0.1640 |
| 2017 | 9-Aug-17 | SAN LUIS OBISPO | LETTUCE, HEAD (ALL OR UNSPEC) | Redacted4 | 0.6719 | 2 | A | G | 0.1680 |
| 2017 | 3-Aug-17 | SANTA BARBARA | LETTUCE, HEAD (ALL OR UNSPEC) | Redacted4 | 0.6719 | 2 | A | G | 0.1680 |
| 2017 | 8-Aug-17 | SAN LUIS OBISPO | LETTUCE, HEAD (ALL OR UNSPEC) | Redacted4 | 0.5879 | 1.7 | A | G | 0.1729 |
| 2017 | 25-Nov-17 | SANTA CLARA | LETTUCE, LEAF (ALL OR UNSPEC) | Redacted4 | 14.949 | 76 | A | G | 0.0983 |
| 2017 | 24-Oct-17 | KERN | LETTUCE, LEAF (ALL OR UNSPEC) | Redacted4 | 4.955 | 19 | A | A | 0.1304 |
| 2017 | 10-Oct-17 | KERN | LETTUCE, LEAF (ALL OR UNSPEC) | Redacted4 | 1.8476 | 7 | A | A | 0.1320 |
| 2017 | 28-Nov-17 | VENTURA | LETTUCE, LEAF (ALL OR UNSPEC) | Redacted4 | 2.6035 | 8 | A | G | 0.1627 |
| 2017 | 11-Dec-17 | VENTURA | MUSTARD GREENS, (LEAFY VEGETABLE) | Redacted4 | 0.5039 | 3.6 | A | G | 0.0700 |
| 2017 | 4-Nov-17 | VENTURA | MUSTARD GREENS, (LEAFY VEGETABLE) | Redacted4 | 0.6719 | 3.2 | A | G | 0.1050 |
| 2017 | 15-Jun-17 | KINGS | ONION (DRY, SPANISH, WHITE, YELLOW, RED, ETC.) | Redacted4 | 0.0472 | 40 | A | A | 0.0006 |
| 2017 | 15-Jun-17 | KINGS | ONION (DRY, SPANISH, WHITE, YELLOW, RED, ETC.) | Redacted4 | 0.1837 | 155.6 | A | A | 0.0006 |
| 2017 | 15-Jun-17 | KINGS | ONION (DRY, SPANISH, WHITE, YELLOW, RED, ETC.) | Redacted4 | 0.1857 | 157 | A | A | 0.0006 |
| 2017 | 11-Jul-17 | KINGS | ONION (DRY, SPANISH, WHITE, YELLOW, RED, ETC.) | Redacted4 | 0.2041 | 155.6 | A | A | 0.0007 |
| 2017 | 11-Jul-17 | KINGS | ONION (DRY, SPANISH, WHITE, YELLOW, RED, ETC.) | Redacted4 | 0.206 | 157 | A | A | 0.0007 |
| 2017 | 12-Jun-17 | KINGS | ONION (DRY, SPANISH, WHITE, YELLOW, RED, ETC.) | Redacted4 | 0.2165 | 157 | A | A | 0.0007 |
| 2017 | 12-Jun-17 | KINGS | ONION (DRY, SPANISH, WHITE, YELLOW, RED, ETC.) | Redacted4 | 0.2139 | 155 | A | A | 0.0007 |
| 2017 | 12-Jun-17 | FRESNO | ONION (DRY, SPANISH, WHITE, YELLOW, RED, ETC.) | Redacted4 | 0.2264 | 150 | A | A | 0.0008 |
| 2017 | 9-Jul-17 | FRESNO | ONION (DRY, SPANISH, WHITE, YELLOW, RED, ETC.) | Redacted4 | 0.2264 | 150 | A | A | 0.0008 |
| 2017 | 12-Jun-17 | FRESNO | ONION (DRY, SPANISH, WHITE, YELLOW, RED, ETC.) | Redacted4 | 0.1601 | 106 | A | A | 0.0008 |
| 2017 | 9-Jul-17 | FRESNO | ONION (DRY, SPANISH, WHITE, YELLOW, RED, ETC.) | Redacted4 | 0.1601 | 106 | A | A | 0.0008 |
| 2017 | 9-Jul-17 | FRESNO | ONION (DRY, SPANISH, WHITE, YELLOW, RED, ETC.) | Redacted4 | 0.0335 | 22 | A | A | 0.0008 |
| 2017 | 12-Jun-17 | FRESNO | ONION (DRY, SPANISH, WHITE, YELLOW, RED, ETC.) | Redacted4 | 0.0335 | 22 | A | A | 0.0008 |
| 2017 | 26-Jul-17 | FRESNO | ONION (DRY, SPANISH, WHITE, YELLOW, RED, ETC.) | Redacted4 | 0.0374 | 22 | A | A | 0.0009 |
| 2017 | 26-Jul-17 | FRESNO | ONION (DRY, SPANISH, WHITE, YELLOW, RED, ETC.) | Redacted4 | 0.2047 | 120 | A | A | 0.0009 |
| 2017 | 26-Jul-17 | FRESNO | ONION (DRY, SPANISH, WHITE, YELLOW, RED, ETC.) | Redacted4 | 0.1811 | 106 | A | A | 0.0009 |
| 2017 | 27-Jul-17 | KINGS | ONION (DRY, SPANISH, WHITE, YELLOW, RED, ETC.) | Redacted4 | 0.1378 | 75 | A | A | 0.0009 |
| 2017 | 27-Jul-17 | KINGS | ONION (DRY, SPANISH, WHITE, YELLOW, RED, ETC.) | Redacted4 | 0.2861 | 155.6 | A | A | 0.0009 |
| 2017 | 16-Jun-17 | KINGS | ONION (DRY, SPANISH, WHITE, YELLOW, RED, ETC.) | Redacted4 | 0.1837 | 80 | A | A | 0.0011 |
| 2017 | 16-Jun-17 | KINGS | ONION (DRY, SPANISH, WHITE, YELLOW, RED, ETC.) | Redacted4 | 0.2415 | 105 | A | A | 0.0012 |
| 2017 | 28-May-17 | FRESNO | ONION (DRY, SPANISH, WHITE, YELLOW, RED, ETC.) | Redacted4 | 0.0774 | 32 | A | A | 0.0012 |
| 2017 | 28-May-17 | FRESNO | ONION (DRY, SPANISH, WHITE, YELLOW, RED, ETC.) | Redacted4 | 0.1089 | 45 | A | A | 0.0012 |
| 2017 | 28-May-17 | FRESNO | ONION (DRY, SPANISH, WHITE, YELLOW, RED, ETC.) | Redacted4 | 0.1089 | 45 | A | A | 0.0012 |
| 2017 | 28-May-17 | FRESNO | ONION (DRY, SPANISH, WHITE, YELLOW, RED, ETC.) | Redacted4 | 0.1089 | 45 | A | A | 0.0012 |
| 2017 | 24-May-17 | KINGS | ONION (DRY, SPANISH, WHITE, YELLOW, RED, ETC.) | Redacted4 | 0.3668 | 151 | A | A | 0.0012 |
| 2017 | 27-May-17 | KINGS | ONION (DRY, SPANISH, WHITE, YELLOW, RED, ETC.) | Redacted4 | 0.0486 | 20 | A | A | 0.0012 |
| 2017 | 28-May-17 | FRESNO | ONION (DRY, SPANISH, WHITE, YELLOW, RED, ETC.) | Redacted4 | 0.0171 | 7 | A | A | 0.0012 |
| 2017 | 16-Jun-17 | FRESNO | ONION (DRY, SPANISH, WHITE, YELLOW, RED, ETC.) | Redacted4 | 0.4186 | 168 | A | A | 0.0012 |
| 2017 | 12-Jul-17 | FRESNO | ONION (DRY, SPANISH, WHITE, YELLOW, RED, ETC.) | Redacted4 | 0.4186 | 168 | A | A | 0.0012 |
| 2017 | 15-Jun-17 | KINGS | ONION (DRY, SPANISH, WHITE, YELLOW, RED, ETC.) | Redacted4 | 0.4186 | 152 | A | A | 0.0014 |
| 2017 | 27-Jul-17 | FRESNO | ONION (DRY, SPANISH, WHITE, YELLOW, RED, ETC.) | Redacted4 | 0.4232 | 150 | A | A | 0.0014 |
| 2017 | 16-Jun-17 | KINGS | ONION (DRY, SPANISH, WHITE, YELLOW, RED, ETC.) | Redacted4 | 0.4232 | 150 | A | A | 0.0014 |
| 2017 | 16-Jun-17 | FRESNO | ONION (DRY, SPANISH, WHITE, YELLOW, RED, ETC.) | Redacted4 | 0.4245 | 147 | A | A | 0.0014 |
| 2017 | 23-May-17 | KINGS | ONION (DRY, SPANISH, WHITE, YELLOW, RED, ETC.) | Redacted4 | 0.458 | 155 | A | A | 0.0015 |
| 2017 | 23-May-17 | FRESNO | ONION (DRY, SPANISH, WHITE, YELLOW, RED, ETC.) | Redacted4 | 0.9212 | 120 | A | A | 0.0038 |
| 2017 | 28-May-17 | KINGS | ONION (DRY, SPANISH, WHITE, YELLOW, RED, ETC.) | Redacted4 | 0.4193 | 45 | A | A | 0.0047 |
| 2017 | 28-May-17 | KINGS | ONION (DRY, SPANISH, WHITE, YELLOW, RED, ETC.) | Redacted4 | 0.4193 | 45 | A | A | 0.0047 |
| 2017 | 14-Jul-17 | FRESNO | ONION (DRY, SPANISH, WHITE, YELLOW, RED, ETC.) | Redacted4 | 0.4199 | 40 | A | A | 0.0052 |
| 2017 | 22-Jun-17 | FRESNO | ONION (DRY, SPANISH, WHITE, YELLOW, RED, ETC.) | Redacted4 | 0.4199 | 40 | A | A | 0.0052 |
| 2017 | 3-Jun-17 | KERN | ONION (DRY, SPANISH, WHITE, YELLOW, RED, ETC.) | Redacted4 | 8.3983 | 40 | A | G | 0.1050 |
| 2017 | 17-Jun-17 | KERN | ONION (DRY, SPANISH, WHITE, YELLOW, RED, ETC.) | Redacted4 | 9.4901 | 40 | A | A | 0.1186 |
| 2017 | 13-Jun-17 | SAN BENITO | ONION (DRY, SPANISH, WHITE, YELLOW, RED, ETC.) | Redacted4 | 3.3593 | 13 | A | G | 0.1292 |
| 2017 | 26-May-17 | SAN BENITO | ONION (DRY, SPANISH, WHITE, YELLOW, RED, ETC.) | Redacted4 | 3.3593 | 13 | A | G | 0.1292 |
| 2017 | 5-May-17 | SAN BENITO | ONION (DRY, SPANISH, WHITE, YELLOW, RED, ETC.) | Redacted4 | 3.3593 | 13 | A | G | 0.1292 |
| 2017 | 5-May-17 | SAN BENITO | ONION (DRY, SPANISH, WHITE, YELLOW, RED, ETC.) | Redacted4 | 3.1074 | 12 | A | G | 0.1295 |
| 2017 | 13-Jun-17 | SAN BENITO | ONION (DRY, SPANISH, WHITE, YELLOW, RED, ETC.) | Redacted4 | 3.1074 | 12 | A | G | 0.1295 |
| 2017 | 26-May-17 | SAN BENITO | ONION (DRY, SPANISH, WHITE, YELLOW, RED, ETC.) | Redacted4 | 3.1074 | 12 | A | G | 0.1295 |
| 2017 | 13-Jun-17 | SAN BENITO | ONION (DRY, SPANISH, WHITE, YELLOW, RED, ETC.) | Redacted4 | 2.9394 | 11.3 | A | G | 0.1301 |
| 2017 | 5-May-17 | SAN BENITO | ONION (DRY, SPANISH, WHITE, YELLOW, RED, ETC.) | Redacted4 | 2.9394 | 11.3 | A | G | 0.1301 |
| 2017 | 26-May-17 | SAN BENITO | ONION (DRY, SPANISH, WHITE, YELLOW, RED, ETC.) | Redacted4 | 2.9394 | 11.3 | A | G | 0.1301 |
| 2017 | 13-Jun-17 | SAN BENITO | ONION (DRY, SPANISH, WHITE, YELLOW, RED, ETC.) | Redacted4 | 2.6035 | 10 | A | G | 0.1302 |
| 2017 | 5-May-17 | SAN BENITO | ONION (DRY, SPANISH, WHITE, YELLOW, RED, ETC.) | Redacted4 | 2.6035 | 10 | A | G | 0.1302 |
| 2017 | 26-May-17 | SAN BENITO | ONION (DRY, SPANISH, WHITE, YELLOW, RED, ETC.) | Redacted4 | 2.6035 | 10 | A | G | 0.1302 |
| 2017 | 25-Jun-17 | MONTEREY | ONION (DRY, SPANISH, WHITE, YELLOW, RED, ETC.) | Redacted4 | 6.1308 | 18.8 | A | G | 0.1631 |
| 2017 | 25-Jun-17 | MONTEREY | ONION (DRY, SPANISH, WHITE, YELLOW, RED, ETC.) | Redacted4 | 5.8788 | 18 | A | G | 0.1633 |
| 2017 | 21-Jul-17 | SANTA CLARA | ONION (DRY, SPANISH, WHITE, YELLOW, RED, ETC.) | Redacted4 | 9.1542 | 28 | A | G | 0.1635 |
| 2017 | 24-Jun-17 | MONTEREY | ONION (DRY, SPANISH, WHITE, YELLOW, RED, ETC.) | Redacted4 | 7.1386 | 21.7 | A | G | 0.1645 |
| 2017 | 19-May-17 | KERN | ONION (DRY, SPANISH, WHITE, YELLOW, RED, ETC.) | Redacted4 | 8.2303 | 25 | A | A | 0.1646 |
| 2017 | 15-Jun-17 | KERN | ONION (DRY, SPANISH, WHITE, YELLOW, RED, ETC.) | Redacted4 | 8.3143 | 24 | A | A | 0.1732 |
| 2017 | 22-Jun-17 | KERN | ONION (DRY, SPANISH, WHITE, YELLOW, RED, ETC.) | Redacted4 | 8.3983 | 22 | A | A | 0.1909 |
| 2017 | 26-Jun-17 | MONTEREY | ONION (DRY, SPANISH, WHITE, YELLOW, RED, ETC.) | Redacted4 | 10.3929 | 26.4 | A | G | 0.1968 |
| 2017 | 25-Jun-17 | MONTEREY | ONION (DRY, SPANISH, WHITE, YELLOW, RED, ETC.) | Redacted4 | 10.4139 | 26.4 | A | G | 0.1972 |
| 2017 | 5-Jul-17 | KERN | ONION (DRY, SPANISH, WHITE, YELLOW, RED, ETC.) | Redacted4 | 8.3143 | 19 | A | A | 0.2188 |
| 2017 | 2-Jul-17 | MONTEREY | ONION (DRY, SPANISH, WHITE, YELLOW, RED, ETC.) | Redacted4 | 8.2303 | 18 | A | G | 0.2286 |
| 2017 | 11-Aug-17 | MONTEREY | ONION (DRY, SPANISH, WHITE, YELLOW, RED, ETC.) | Redacted4 | 8.2303 | 18 | A | G | 0.2286 |
| 2017 | 30-Jun-17 | MONTEREY | ONION (DRY, SPANISH, WHITE, YELLOW, RED, ETC.) | Redacted4 | 8.7342 | 19.1 | A | G | 0.2286 |
| 2017 | 19-Jun-17 | MONTEREY | ONION (DRY, SPANISH, WHITE, YELLOW, RED, ETC.) | Redacted4 | 8.7342 | 19.1 | A | G | 0.2286 |
| 2017 | 7-Jul-17 | MONTEREY | ONION (DRY, SPANISH, WHITE, YELLOW, RED, ETC.) | Redacted4 | 12.0936 | 26.4 | A | G | 0.2290 |
| 2017 | 1-Jul-17 | MONTEREY | ONION (DRY, SPANISH, WHITE, YELLOW, RED, ETC.) | Redacted4 | 8.6503 | 18.8 | A | G | 0.2301 |
| 2017 | 9-Aug-17 | MONTEREY | ONION (DRY, SPANISH, WHITE, YELLOW, RED, ETC.) | Redacted4 | 8.6503 | 18.8 | A | G | 0.2301 |
| 2017 | 2-Jul-17 | MONTEREY | ONION (DRY, SPANISH, WHITE, YELLOW, RED, ETC.) | Redacted4 | 9.994 | 21.7 | A | G | 0.2303 |
| 2017 | 3-Jul-17 | SAN BENITO | ONION (DRY, SPANISH, WHITE, YELLOW, RED, ETC.) | Redacted4 | 6.6347 | 11.3 | A | G | 0.2936 |
| 2017 | 21-Jun-17 | SAN BENITO | ONION (DRY, SPANISH, WHITE, YELLOW, RED, ETC.) | Redacted4 | 6.6347 | 11.3 | A | G | 0.2936 |
| 2017 | 21-Jun-17 | SAN BENITO | ONION (DRY, SPANISH, WHITE, YELLOW, RED, ETC.) | Redacted4 | 5.8788 | 10 | A | G | 0.2939 |
| 2017 | 6-Jul-17 | SAN BENITO | ONION (DRY, SPANISH, WHITE, YELLOW, RED, ETC.) | Redacted4 | 7.0546 | 12 | A | G | 0.2939 |
| 2017 | 21-Jun-17 | SAN BENITO | ONION (DRY, SPANISH, WHITE, YELLOW, RED, ETC.) | Redacted4 | 7.6425 | 13 | A | G | 0.2939 |
| 2017 | 18-Sep-17 | FRESNO | ORANGE (ALL OR UNSPEC) | Redacted4 | 4.4511 | 17.2 | A | G | 0.1294 |
| 2017 | 19-Sep-17 | FRESNO | ORANGE (ALL OR UNSPEC) | Redacted4 | 8.7342 | 33.6 | A | G | 0.1300 |
| 2017 | 20-Sep-17 | FRESNO | ORANGE (ALL OR UNSPEC) | Redacted4 | 8.2303 | 31.5 | A | G | 0.1306 |
| 2017 | 21-Sep-17 | FRESNO | ORANGE (ALL OR UNSPEC) | Redacted4 | 13.1014 | 49.9 | A | G | 0.1313 |
| 2017 | 19-Jul-17 | SAN BENITO | PEPPERS (CHILI TYPE) (FLAVORING AND SPICE CROP) | Redacted4 | 7.4745 | 38 | A | G | 0.0983 |
| 2017 | 17-May-17 | SANTA CLARA | PEPPERS (FRUITING VEGETABLE), (BELL,CHILI, ETC.) | Redacted4 | 0.5039 | 8 | A | G | 0.0315 |
| 2017 | 6-Jun-17 | SANTA CLARA | PEPPERS (FRUITING VEGETABLE), (BELL,CHILI, ETC.) | Redacted4 | 1.1758 | 18 | A | G | 0.0327 |
| 2017 | 18-May-17 | SANTA CLARA | PEPPERS (FRUITING VEGETABLE), (BELL,CHILI, ETC.) | Redacted4 | 3.2753 | 50 | A | G | 0.0328 |
| 2017 | 15-Jun-17 | SANTA CLARA | PEPPERS (FRUITING VEGETABLE), (BELL,CHILI, ETC.) | Redacted4 | 3.8632 | 39 | A | G | 0.0495 |
| 2017 | 24-May-17 | SANTA CLARA | PEPPERS (FRUITING VEGETABLE), (BELL,CHILI, ETC.) | Redacted4 | 2.6035 | 20 | A | G | 0.0651 |
| 2017 | 2-Jun-17 | SANTA CLARA | PEPPERS (FRUITING VEGETABLE), (BELL,CHILI, ETC.) | Redacted4 | 3.7792 | 29 | A | G | 0.0652 |
| 2017 | 17-Sep-17 | SANTA CLARA | PEPPERS (FRUITING VEGETABLE), (BELL,CHILI, ETC.) | Redacted4 | 2.3515 | 18 | A | G | 0.0653 |
| 2017 | 9-Sep-17 | SANTA CLARA | PEPPERS (FRUITING VEGETABLE), (BELL,CHILI, ETC.) | Redacted4 | 2.3515 | 18 | A | G | 0.0653 |
| 2017 | 6-Sep-17 | SANTA CLARA | PEPPERS (FRUITING VEGETABLE), (BELL,CHILI, ETC.) | Redacted4 | 2.3515 | 18 | A | G | 0.0653 |
| 2017 | 21-Jun-17 | SANTA CLARA | PEPPERS (FRUITING VEGETABLE), (BELL,CHILI, ETC.) | Redacted4 | 2.3515 | 18 | A | G | 0.0653 |
| 2017 | 5-Aug-17 | SAN BENITO | PEPPERS (FRUITING VEGETABLE), (BELL,CHILI, ETC.) | Redacted4 | 3.5273 | 27 | A | G | 0.0653 |
| 2017 | 5-Jun-17 | SANTA CLARA | PEPPERS (FRUITING VEGETABLE), (BELL,CHILI, ETC.) | Redacted4 | 5.123 | 39 | A | G | 0.0657 |
| 2017 | 7-Sep-17 | SANTA CLARA | PEPPERS (FRUITING VEGETABLE), (BELL,CHILI, ETC.) | Redacted4 | 3.0234 | 23 | A | G | 0.0657 |
| 2017 | 10-Sep-17 | SANTA CLARA | PEPPERS (FRUITING VEGETABLE), (BELL,CHILI, ETC.) | Redacted4 | 5.7948 | 44 | A | G | 0.0659 |
| 2017 | 12-Aug-17 | SANTA CLARA | PEPPERS (FRUITING VEGETABLE), (BELL,CHILI, ETC.) | Redacted4 | 1.8476 | 14 | A | G | 0.0660 |
| 2017 | 15-Aug-17 | SAN BENITO | PEPPERS (FRUITING VEGETABLE), (BELL,CHILI, ETC.) | Redacted4 | 4.955 | 30 | A | G | 0.0826 |
| 2017 | 11-Jul-17 | SAN BENITO | PEPPERS (FRUITING VEGETABLE), (BELL,CHILI, ETC.) | Redacted4 | 2.0996 | 11 | A | G | 0.0954 |
| 2017 | 30-Sep-17 | SAN BENITO | PEPPERS (FRUITING VEGETABLE), (BELL,CHILI, ETC.) | Redacted4 | 2.7714 | 14.5 | A | G | 0.0956 |
| 2017 | 29-Jul-17 | SANTA CLARA | PEPPERS (FRUITING VEGETABLE), (BELL,CHILI, ETC.) | Redacted4 | 1.9316 | 10 | A | G | 0.0966 |
| 2017 | 13-Oct-17 | SANTA CLARA | PEPPERS (FRUITING VEGETABLE), (BELL,CHILI, ETC.) | Redacted4 | 1.9316 | 10 | A | G | 0.0966 |
| 2017 | 11-Jul-17 | SAN BENITO | PEPPERS (FRUITING VEGETABLE), (BELL,CHILI, ETC.) | Redacted4 | 4.4511 | 23 | A | G | 0.0968 |
| 2017 | 14-Oct-17 | SAN BENITO | PEPPERS (FRUITING VEGETABLE), (BELL,CHILI, ETC.) | Redacted4 | 2.5195 | 13 | A | G | 0.0969 |
| 2017 | 30-Sep-17 | SAN BENITO | PEPPERS (FRUITING VEGETABLE), (BELL,CHILI, ETC.) | Redacted4 | 2.5195 | 13 | A | G | 0.0969 |
| 2017 | 30-Sep-17 | SAN BENITO | PEPPERS (FRUITING VEGETABLE), (BELL,CHILI, ETC.) | Redacted4 | 2.4355 | 12.5 | A | G | 0.0974 |
| 2017 | 22-Jul-17 | SAN BENITO | PEPPERS (FRUITING VEGETABLE), (BELL,CHILI, ETC.) | Redacted4 | 2.4355 | 12.5 | A | G | 0.0974 |
| 2017 | 11-Jul-17 | SAN BENITO | PEPPERS (FRUITING VEGETABLE), (BELL,CHILI, ETC.) | Redacted4 | 4.871 | 25 | A | G | 0.0974 |
| 2017 | 14-Oct-17 | SAN BENITO | PEPPERS (FRUITING VEGETABLE), (BELL,CHILI, ETC.) | Redacted4 | 4.7031 | 24.1 | A | G | 0.0976 |
| 2017 | 19-Oct-17 | SAN BENITO | PEPPERS (FRUITING VEGETABLE), (BELL,CHILI, ETC.) | Redacted4 | 4.7031 | 24.1 | A | G | 0.0976 |
| 2017 | 14-Jul-17 | SAN BENITO | PEPPERS (FRUITING VEGETABLE), (BELL,CHILI, ETC.) | Redacted4 | 4.7031 | 24.1 | A | G | 0.0976 |
| 2017 | 24-Aug-17 | SANTA CLARA | PEPPERS (FRUITING VEGETABLE), (BELL,CHILI, ETC.) | Redacted4 | 3.6113 | 18.49 | A | G | 0.0977 |
| 2017 | 1-Aug-17 | SANTA CLARA | PEPPERS (FRUITING VEGETABLE), (BELL,CHILI, ETC.) | Redacted4 | 3.6113 | 18.49 | A | G | 0.0977 |
| 2017 | 11-Jul-17 | SANTA CLARA | PEPPERS (FRUITING VEGETABLE), (BELL,CHILI, ETC.) | Redacted4 | 3.6113 | 18.49 | A | G | 0.0977 |
| 2017 | 15-Aug-17 | SANTA CLARA | PEPPERS (FRUITING VEGETABLE), (BELL,CHILI, ETC.) | Redacted4 | 3.6113 | 18.49 | A | G | 0.0977 |
| 2017 | 15-Jul-17 | SAN BENITO | PEPPERS (FRUITING VEGETABLE), (BELL,CHILI, ETC.) | Redacted4 | 4.871 | 24.9 | A | G | 0.0978 |
| 2017 | 14-Oct-17 | SAN BENITO | PEPPERS (FRUITING VEGETABLE), (BELL,CHILI, ETC.) | Redacted4 | 4.871 | 24.9 | A | G | 0.0978 |
| 2017 | 19-Oct-17 | SAN BENITO | PEPPERS (FRUITING VEGETABLE), (BELL,CHILI, ETC.) | Redacted4 | 4.871 | 24.9 | A | G | 0.0978 |
| 2017 | 7-Sep-17 | SANTA CLARA | PEPPERS (FRUITING VEGETABLE), (BELL,CHILI, ETC.) | Redacted4 | 1.7636 | 9 | A | G | 0.0980 |
| 2017 | 4-Aug-17 | SANTA CLARA | PEPPERS (FRUITING VEGETABLE), (BELL,CHILI, ETC.) | Redacted4 | 1.7636 | 9 | A | G | 0.0980 |
| 2017 | 18-Jul-17 | SANTA CLARA | PEPPERS (FRUITING VEGETABLE), (BELL,CHILI, ETC.) | Redacted4 | 1.7636 | 9 | A | G | 0.0980 |
| 2017 | 19-Jul-17 | SAN BENITO | PEPPERS (FRUITING VEGETABLE), (BELL,CHILI, ETC.) | Redacted4 | 5.2909 | 27 | A | G | 0.0980 |
| 2017 | 27-Jul-17 | SANTA CLARA | PEPPERS (FRUITING VEGETABLE), (BELL,CHILI, ETC.) | Redacted4 | 5.2909 | 27 | A | G | 0.0980 |
| 2017 | 21-Jul-17 | SAN BENITO | PEPPERS (FRUITING VEGETABLE), (BELL,CHILI, ETC.) | Redacted4 | 5.8788 | 30 | A | G | 0.0980 |
| 2017 | 29-Jul-17 | SAN BENITO | PEPPERS (FRUITING VEGETABLE), (BELL,CHILI, ETC.) | Redacted4 | 2.9394 | 15 | A | G | 0.0980 |
| 2017 | 21-Jul-17 | SAN BENITO | PEPPERS (FRUITING VEGETABLE), (BELL,CHILI, ETC.) | Redacted4 | 2.9394 | 15 | A | G | 0.0980 |
| 2017 | 19-Jul-17 | SAN BENITO | PEPPERS (FRUITING VEGETABLE), (BELL,CHILI, ETC.) | Redacted4 | 2.9394 | 15 | A | G | 0.0980 |
| 2017 | 6-Oct-17 | SAN BENITO | PEPPERS (FRUITING VEGETABLE), (BELL,CHILI, ETC.) | Redacted4 | 2.9394 | 15 | A | G | 0.0980 |
| 2017 | 28-Jul-17 | SAN BENITO | PEPPERS (FRUITING VEGETABLE), (BELL,CHILI, ETC.) | Redacted4 | 6.4667 | 33 | A | G | 0.0980 |
| 2017 | 20-Jul-17 | SAN BENITO | PEPPERS (FRUITING VEGETABLE), (BELL,CHILI, ETC.) | Redacted4 | 3.5273 | 18 | A | G | 0.0980 |
| 2017 | 5-Aug-17 | SANTA CLARA | PEPPERS (FRUITING VEGETABLE), (BELL,CHILI, ETC.) | Redacted4 | 7.0546 | 36 | A | G | 0.0980 |
| 2017 | 20-Jul-17 | SANTA CLARA | PEPPERS (FRUITING VEGETABLE), (BELL,CHILI, ETC.) | Redacted4 | 3.5273 | 18 | A | G | 0.0980 |
| 2017 | 29-Jun-17 | SANTA CLARA | PEPPERS (FRUITING VEGETABLE), (BELL,CHILI, ETC.) | Redacted4 | 3.5273 | 18 | A | G | 0.0980 |
| 2017 | 5-Jul-17 | SANTA CLARA | PEPPERS (FRUITING VEGETABLE), (BELL,CHILI, ETC.) | Redacted4 | 7.6425 | 39 | A | G | 0.0980 |
| 2017 | 10-Sep-17 | SAN BENITO | PEPPERS (FRUITING VEGETABLE), (BELL,CHILI, ETC.) | Redacted4 | 4.1152 | 21 | A | G | 0.0980 |
| 2017 | 19-Jul-17 | SAN BENITO | PEPPERS (FRUITING VEGETABLE), (BELL,CHILI, ETC.) | Redacted4 | 4.1152 | 21 | A | G | 0.0980 |
| 2017 | 7-Aug-17 | SANTA CLARA | PEPPERS (FRUITING VEGETABLE), (BELL,CHILI, ETC.) | Redacted4 | 4.1152 | 21 | A | G | 0.0980 |
| 2017 | 28-Jul-17 | SAN BENITO | PEPPERS (FRUITING VEGETABLE), (BELL,CHILI, ETC.) | Redacted4 | 1.1758 | 6 | A | G | 0.0980 |
| 2017 | 9-Jul-17 | SAN BENITO | PEPPERS (FRUITING VEGETABLE), (BELL,CHILI, ETC.) | Redacted4 | 6.1308 | 31.26 | A | G | 0.0981 |
| 2017 | 20-Jul-17 | SAN BENITO | PEPPERS (FRUITING VEGETABLE), (BELL,CHILI, ETC.) | Redacted4 | 4.871 | 24.8 | A | G | 0.0982 |
| 2017 | 11-Jul-17 | SAN BENITO | PEPPERS (FRUITING VEGETABLE), (BELL,CHILI, ETC.) | Redacted4 | 9.826 | 50 | A | G | 0.0983 |
| 2017 | 12-Jun-17 | SANTA CLARA | PEPPERS (FRUITING VEGETABLE), (BELL,CHILI, ETC.) | Redacted4 | 9.826 | 50 | A | G | 0.0983 |
| 2017 | 15-Jul-17 | SANTA CLARA | PEPPERS (FRUITING VEGETABLE), (BELL,CHILI, ETC.) | Redacted4 | 9.826 | 50 | A | G | 0.0983 |
| 2017 | 27-Jun-17 | SANTA CLARA | PEPPERS (FRUITING VEGETABLE), (BELL,CHILI, ETC.) | Redacted4 | 9.826 | 50 | A | G | 0.0983 |
| 2017 | 27-Aug-17 | SANTA CLARA | PEPPERS (FRUITING VEGETABLE), (BELL,CHILI, ETC.) | Redacted4 | 9.826 | 50 | A | G | 0.0983 |
| 2017 | 19-Oct-17 | SAN BENITO | PEPPERS (FRUITING VEGETABLE), (BELL,CHILI, ETC.) | Redacted4 | 7.4745 | 38 | A | G | 0.0983 |
| 2017 | 7-Jun-17 | SANTA CLARA | PEPPERS (FRUITING VEGETABLE), (BELL,CHILI, ETC.) | Redacted4 | 7.4745 | 38 | A | G | 0.0983 |
| 2017 | 29-Sep-17 | SAN BENITO | PEPPERS (FRUITING VEGETABLE), (BELL,CHILI, ETC.) | Redacted4 | 6.8866 | 35 | A | G | 0.0984 |
| 2017 | 4-Aug-17 | SAN BENITO | PEPPERS (FRUITING VEGETABLE), (BELL,CHILI, ETC.) | Redacted4 | 12.0096 | 61 | A | G | 0.0984 |
| 2017 | 18-Jul-17 | SAN BENITO | PEPPERS (FRUITING VEGETABLE), (BELL,CHILI, ETC.) | Redacted4 | 12.0096 | 61 | A | G | 0.0984 |
| 2017 | 22-Jul-17 | SAN BENITO | PEPPERS (FRUITING VEGETABLE), (BELL,CHILI, ETC.) | Redacted4 | 2.8554 | 14.5 | A | G | 0.0985 |
| 2017 | 12-Aug-17 | SANTA CLARA | PEPPERS (FRUITING VEGETABLE), (BELL,CHILI, ETC.) | Redacted4 | 5.7108 | 29 | A | G | 0.0985 |
| 2017 | 16-Jun-17 | SANTA CLARA | PEPPERS (FRUITING VEGETABLE), (BELL,CHILI, ETC.) | Redacted4 | 5.7108 | 29 | A | G | 0.0985 |
| 2017 | 2-Jul-17 | SANTA CLARA | PEPPERS (FRUITING VEGETABLE), (BELL,CHILI, ETC.) | Redacted4 | 5.7108 | 29 | A | G | 0.0985 |
| 2017 | 31-Jul-17 | SANTA CLARA | PEPPERS (FRUITING VEGETABLE), (BELL,CHILI, ETC.) | Redacted4 | 5.7108 | 29 | A | G | 0.0985 |
| 2017 | 15-Jul-17 | SANTA CLARA | PEPPERS (FRUITING VEGETABLE), (BELL,CHILI, ETC.) | Redacted4 | 5.7108 | 29 | A | G | 0.0985 |
| 2017 | 27-Aug-17 | SANTA CLARA | PEPPERS (FRUITING VEGETABLE), (BELL,CHILI, ETC.) | Redacted4 | 5.7108 | 29 | A | G | 0.0985 |
| 2017 | 10-Aug-17 | SAN BENITO | PEPPERS (FRUITING VEGETABLE), (BELL,CHILI, ETC.) | Redacted4 | 4.5351 | 23 | A | G | 0.0986 |
| 2017 | 29-Jul-17 | SANTA CLARA | PEPPERS (FRUITING VEGETABLE), (BELL,CHILI, ETC.) | Redacted4 | 4.5351 | 23 | A | G | 0.0986 |
| 2017 | 8-Jul-17 | SAN BENITO | PEPPERS (FRUITING VEGETABLE), (BELL,CHILI, ETC.) | Redacted4 | 6.2147 | 31.49 | A | G | 0.0987 |
| 2017 | 8-Aug-17 | SANTA CLARA | PEPPERS (FRUITING VEGETABLE), (BELL,CHILI, ETC.) | Redacted4 | 3.9472 | 20 | A | G | 0.0987 |
| 2017 | 10-Jul-17 | SAN BENITO | PEPPERS (FRUITING VEGETABLE), (BELL,CHILI, ETC.) | Redacted4 | 4.6191 | 23.4 | A | G | 0.0987 |
| 2017 | 9-Sep-17 | SAN BENITO | PEPPERS (FRUITING VEGETABLE), (BELL,CHILI, ETC.) | Redacted4 | 4.6191 | 23.4 | A | G | 0.0987 |
| 2017 | 20-Jul-17 | SAN BENITO | PEPPERS (FRUITING VEGETABLE), (BELL,CHILI, ETC.) | Redacted4 | 3.3593 | 17 | A | G | 0.0988 |
| 2017 | 29-Jun-17 | SANTA CLARA | PEPPERS (FRUITING VEGETABLE), (BELL,CHILI, ETC.) | Redacted4 | 3.3593 | 17 | A | G | 0.0988 |
| 2017 | 15-Jun-17 | SANTA CLARA | PEPPERS (FRUITING VEGETABLE), (BELL,CHILI, ETC.) | Redacted4 | 3.3593 | 17 | A | G | 0.0988 |
| 2017 | 19-Jul-17 | SANTA CLARA | PEPPERS (FRUITING VEGETABLE), (BELL,CHILI, ETC.) | Redacted4 | 3.3593 | 17 | A | G | 0.0988 |
| 2017 | 10-Aug-17 | SANTA CLARA | PEPPERS (FRUITING VEGETABLE), (BELL,CHILI, ETC.) | Redacted4 | 3.3593 | 17 | A | G | 0.0988 |
| 2017 | 22-Sep-17 | SANTA CLARA | PEPPERS (FRUITING VEGETABLE), (BELL,CHILI, ETC.) | Redacted4 | 7.2225 | 36.5 | A | G | 0.0989 |
| 2017 | 7-Sep-17 | SANTA CLARA | PEPPERS (FRUITING VEGETABLE), (BELL,CHILI, ETC.) | Redacted4 | 7.2225 | 36.5 | A | G | 0.0989 |
| 2017 | 17-Jul-17 | SANTA CLARA | PEPPERS (FRUITING VEGETABLE), (BELL,CHILI, ETC.) | Redacted4 | 2.7714 | 14 | A | G | 0.0990 |
| 2017 | 6-Aug-17 | SANTA CLARA | PEPPERS (FRUITING VEGETABLE), (BELL,CHILI, ETC.) | Redacted4 | 2.7714 | 14 | A | G | 0.0990 |
| 2017 | 14-Oct-17 | SAN BENITO | PEPPERS (FRUITING VEGETABLE), (BELL,CHILI, ETC.) | Redacted4 | 4.955 | 25 | A | G | 0.0991 |
| 2017 | 30-Sep-17 | SAN BENITO | PEPPERS (FRUITING VEGETABLE), (BELL,CHILI, ETC.) | Redacted4 | 4.955 | 25 | A | G | 0.0991 |
| 2017 | 10-Sep-17 | SAN BENITO | PEPPERS (FRUITING VEGETABLE), (BELL,CHILI, ETC.) | Redacted4 | 3.2753 | 16.5 | A | G | 0.0993 |
| 2017 | 19-Jul-17 | SAN BENITO | PEPPERS (FRUITING VEGETABLE), (BELL,CHILI, ETC.) | Redacted4 | 3.2753 | 16.5 | A | G | 0.0993 |
| 2017 | 5-Aug-17 | SAN BENITO | PEPPERS (FRUITING VEGETABLE), (BELL,CHILI, ETC.) | Redacted4 | 2.1836 | 11 | A | G | 0.0993 |
| 2017 | 19-Jul-17 | SAN BENITO | PEPPERS (FRUITING VEGETABLE), (BELL,CHILI, ETC.) | Redacted4 | 3.1914 | 16 | A | G | 0.0997 |
| 2017 | 6-Oct-17 | SAN BENITO | PEPPERS (FRUITING VEGETABLE), (BELL,CHILI, ETC.) | Redacted4 | 3.1914 | 16 | A | G | 0.0997 |
| 2017 | 17-Aug-17 | SANTA CLARA | PEPPERS (FRUITING VEGETABLE), (BELL,CHILI, ETC.) | Redacted4 | 1.0078 | 5 | A | G | 0.1008 |
| 2017 | 20-Jul-17 | SANTA CLARA | PEPPERS (FRUITING VEGETABLE), (BELL,CHILI, ETC.) | Redacted4 | 1.0078 | 5 | A | G | 0.1008 |
| 2017 | 16-Jun-17 | SAN BENITO | PEPPERS (FRUITING VEGETABLE), (BELL,CHILI, ETC.) | Redacted4 | 0.5039 | 2.4 | A | G | 0.1050 |
| 2017 | 28-Jun-17 | SAN BENITO | PEPPERS (FRUITING VEGETABLE), (BELL,CHILI, ETC.) | Redacted4 | 0.5039 | 2.4 | A | G | 0.1050 |
| 2017 | 8-Sep-17 | SAN BENITO | PEPPERS (FRUITING VEGETABLE), (BELL,CHILI, ETC.) | Redacted4 | 0.5039 | 2.4 | A | G | 0.1050 |
| 2017 | 11-Aug-17 | SAN BENITO | PEPPERS (FRUITING VEGETABLE), (BELL,CHILI, ETC.) | Redacted4 | 0.3359 | 1.5 | A | G | 0.1120 |
| 2017 | 27-Jul-17 | SAN BENITO | PEPPERS (FRUITING VEGETABLE), (BELL,CHILI, ETC.) | Redacted4 | 0.3359 | 1.5 | A | G | 0.1120 |
| 2017 | 30-Sep-17 | SANTA CLARA | PEPPERS (FRUITING VEGETABLE), (BELL,CHILI, ETC.) | Redacted4 | 2.8554 | 11 | A | G | 0.1298 |
| 2017 | 22-Aug-17 | SANTA CLARA | PEPPERS (FRUITING VEGETABLE), (BELL,CHILI, ETC.) | Redacted4 | 4.0312 | 15.5 | A | G | 0.1300 |
| 2017 | 28-Oct-17 | SANTA CLARA | PEPPERS (FRUITING VEGETABLE), (BELL,CHILI, ETC.) | Redacted4 | 2.6035 | 10 | A | G | 0.1302 |
| 2017 | 16-Aug-17 | SANTA CLARA | PEPPERS (FRUITING VEGETABLE), (BELL,CHILI, ETC.) | Redacted4 | 2.6035 | 10 | A | G | 0.1302 |
| 2017 | 6-Sep-17 | SANTA CLARA | PEPPERS (FRUITING VEGETABLE), (BELL,CHILI, ETC.) | Redacted4 | 2.6035 | 10 | A | G | 0.1302 |
| 2017 | 17-Aug-17 | SANTA CLARA | PEPPERS (FRUITING VEGETABLE), (BELL,CHILI, ETC.) | Redacted4 | 7.0546 | 27 | A | G | 0.1306 |
| 2017 | 2-Sep-17 | SANTA CLARA | PEPPERS (FRUITING VEGETABLE), (BELL,CHILI, ETC.) | Redacted4 | 4.7031 | 18 | A | G | 0.1306 |
| 2017 | 8-Aug-17 | SANTA CLARA | PEPPERS (FRUITING VEGETABLE), (BELL,CHILI, ETC.) | Redacted4 | 4.7031 | 18 | A | G | 0.1306 |
| 2017 | 17-Aug-17 | SANTA CLARA | PEPPERS (FRUITING VEGETABLE), (BELL,CHILI, ETC.) | Redacted4 | 4.7031 | 18 | A | G | 0.1306 |
| 2017 | 25-Aug-17 | SANTA CLARA | PEPPERS (FRUITING VEGETABLE), (BELL,CHILI, ETC.) | Redacted4 | 4.7031 | 18 | A | G | 0.1306 |
| 2017 | 30-Sep-17 | SANTA CLARA | PEPPERS (FRUITING VEGETABLE), (BELL,CHILI, ETC.) | Redacted4 | 4.7031 | 18 | A | G | 0.1306 |
| 2017 | 12-Aug-17 | SAN BENITO | PEPPERS (FRUITING VEGETABLE), (BELL,CHILI, ETC.) | Redacted4 | 8.3143 | 31.8 | A | G | 0.1307 |
| 2017 | 23-Aug-17 | SAN BENITO | PEPPERS (FRUITING VEGETABLE), (BELL,CHILI, ETC.) | Redacted4 | 8.3143 | 31.8 | A | G | 0.1307 |
| 2017 | 15-Aug-17 | SANTA CLARA | PEPPERS (FRUITING VEGETABLE), (BELL,CHILI, ETC.) | Redacted4 | 6.5507 | 25 | A | G | 0.1310 |
| 2017 | 14-Aug-17 | SANTA CLARA | PEPPERS (FRUITING VEGETABLE), (BELL,CHILI, ETC.) | Redacted4 | 6.5507 | 25 | A | G | 0.1310 |
| 2017 | 11-Aug-17 | SAN BENITO | PEPPERS (FRUITING VEGETABLE), (BELL,CHILI, ETC.) | Redacted4 | 8.6503 | 33 | A | G | 0.1311 |
| 2017 | 1-Sep-17 | SANTA CLARA | PEPPERS (FRUITING VEGETABLE), (BELL,CHILI, ETC.) | Redacted4 | 8.6503 | 33 | A | G | 0.1311 |
| 2017 | 16-Aug-17 | SANTA CLARA | PEPPERS (FRUITING VEGETABLE), (BELL,CHILI, ETC.) | Redacted4 | 10.2459 | 39 | A | G | 0.1314 |
| 2017 | 31-Aug-17 | SANTA CLARA | PEPPERS (FRUITING VEGETABLE), (BELL,CHILI, ETC.) | Redacted4 | 10.2459 | 39 | A | G | 0.1314 |
| 2017 | 16-Jul-17 | SANTA CLARA | PEPPERS (FRUITING VEGETABLE), (BELL,CHILI, ETC.) | Redacted4 | 10.2459 | 39 | A | G | 0.1314 |
| 2017 | 10-Sep-17 | SANTA CLARA | PEPPERS (FRUITING VEGETABLE), (BELL,CHILI, ETC.) | Redacted4 | 10.2459 | 39 | A | G | 0.1314 |
| 2017 | 30-Sep-17 | SANTA CLARA | PEPPERS (FRUITING VEGETABLE), (BELL,CHILI, ETC.) | Redacted4 | 10.2459 | 39 | A | G | 0.1314 |
| 2017 | 28-Jul-17 | SANTA CLARA | PEPPERS (FRUITING VEGETABLE), (BELL,CHILI, ETC.) | Redacted4 | 10.2459 | 39 | A | G | 0.1314 |
| 2017 | 21-Sep-17 | SANTA CLARA | PEPPERS (FRUITING VEGETABLE), (BELL,CHILI, ETC.) | Redacted4 | 9.994 | 38 | A | G | 0.1315 |
| 2017 | 11-Aug-17 | SAN BENITO | PEPPERS (FRUITING VEGETABLE), (BELL,CHILI, ETC.) | Redacted4 | 3.9472 | 15 | A | G | 0.1316 |
| 2017 | 3-Sep-17 | SANTA CLARA | PEPPERS (FRUITING VEGETABLE), (BELL,CHILI, ETC.) | Redacted4 | 11.5897 | 44 | A | G | 0.1317 |
| 2017 | 2-Sep-17 | SANTA CLARA | PEPPERS (FRUITING VEGETABLE), (BELL,CHILI, ETC.) | Redacted4 | 4.871 | 18.49 | A | G | 0.1317 |
| 2017 | 1-Oct-17 | SANTA CLARA | PEPPERS (FRUITING VEGETABLE), (BELL,CHILI, ETC.) | Redacted4 | 4.871 | 18.49 | A | G | 0.1317 |
| 2017 | 10-Sep-17 | SANTA CLARA | PEPPERS (FRUITING VEGETABLE), (BELL,CHILI, ETC.) | Redacted4 | 7.6425 | 29 | A | G | 0.1318 |
| 2017 | 18-Aug-17 | SANTA CLARA | PEPPERS (FRUITING VEGETABLE), (BELL,CHILI, ETC.) | Redacted4 | 1.8476 | 7 | A | G | 0.1320 |
| 2017 | 8-Sep-17 | SANTA CLARA | PEPPERS (FRUITING VEGETABLE), (BELL,CHILI, ETC.) | Redacted4 | 1.8476 | 7 | A | G | 0.1320 |
| 2017 | 25-Sep-17 | SANTA CLARA | PEPPERS (FRUITING VEGETABLE), (BELL,CHILI, ETC.) | Redacted4 | 5.5429 | 21 | A | G | 0.1320 |
| 2017 | 11-Aug-17 | SAN BENITO | PEPPERS (FRUITING VEGETABLE), (BELL,CHILI, ETC.) | Redacted4 | 1.5957 | 6 | A | G | 0.1330 |
| 2017 | 7-Sep-17 | SANTA CLARA | PEPPERS (FRUITING VEGETABLE), (BELL,CHILI, ETC.) | Redacted4 | 1.0918 | 4 | A | G | 0.1365 |
| 2017 | 3-Oct-17 | SAN BENITO | PEPPERS (FRUITING VEGETABLE), (BELL,CHILI, ETC.) | Redacted4 | 0.6719 | 2.4 | A | G | 0.1400 |
| 2017 | 18-Jul-17 | SANTA CLARA | PEPPERS (FRUITING VEGETABLE), (BELL,CHILI, ETC.) | Redacted4 | 14.949 | 38 | A | G | 0.1967 |
| 2017 | 30-Aug-17 | SANTA CLARA | PEPPERS (FRUITING VEGETABLE), (BELL,CHILI, ETC.) | Redacted4 | 2.7714 | 7 | A | G | 0.1980 |
| 2017 | 31-Jul-17 | SAN BENITO | PEPPERS (FRUITING VEGETABLE), (BELL,CHILI, ETC.) | Redacted4 | 14.781 | 25 | A | G | 0.2956 |
| 2017 | 7-Aug-17 | MADERA | PISTACHIO (PISTACHE NUT) | Redacted4 | 87.0148 | 388 | A | A | 0.1121 |
| 2017 | 28-Aug-17 | MADERA | PISTACHIO (PISTACHE NUT) | Redacted4 | 2.2675 | 10 | A | A | 0.1134 |
| 2017 | 20-Sep-17 | FRESNO | PISTACHIO (PISTACHE NUT) | Redacted4 | 3.4433 | 15.14 | A | A | 0.1137 |
| 2017 | 4-May-17 | FRESNO | PISTACHIO (PISTACHE NUT) | Redacted4 | 13.6053 | 59.5 | A | G | 0.1143 |
| 2017 | 10-Aug-17 | MADERA | PISTACHIO (PISTACHE NUT) | Redacted4 | 9.1542 | 40 | A | A | 0.1144 |
| 2017 | 4-May-17 | FRESNO | PISTACHIO (PISTACHE NUT) | Redacted4 | 19.2321 | 84 | A | G | 0.1145 |
| 2017 | 9-Aug-17 | FRESNO | PISTACHIO (PISTACHE NUT) | Redacted4 | 27.7144 | 121 | A | G | 0.1145 |
| 2017 | 4-May-17 | FRESNO | PISTACHIO (PISTACHE NUT) | Redacted4 | 27.7144 | 121 | A | G | 0.1145 |
| 2017 | 4-May-17 | FRESNO | PISTACHIO (PISTACHE NUT) | Redacted4 | 35.5248 | 155 | A | G | 0.1146 |
| 2017 | 9-Aug-17 | FRESNO | PISTACHIO (PISTACHE NUT) | Redacted4 | 35.5248 | 155 | A | G | 0.1146 |
| 2017 | 7-Aug-17 | MADERA | PISTACHIO (PISTACHE NUT) | Redacted4 | 22.9274 | 100 | A | A | 0.1146 |
| 2017 | 4-May-17 | FRESNO | PISTACHIO (PISTACHE NUT) | Redacted4 | 26.8746 | 117.2 | A | G | 0.1147 |
| 2017 | 4-May-17 | FRESNO | PISTACHIO (PISTACHE NUT) | Redacted4 | 16.1247 | 70.3 | A | G | 0.1147 |
| 2017 | 9-Aug-17 | FRESNO | PISTACHIO (PISTACHE NUT) | Redacted4 | 16.1247 | 70.3 | A | G | 0.1147 |
| 2017 | 29-Jul-17 | FRESNO | PISTACHIO (PISTACHE NUT) | Redacted4 | 6.8866 | 30 | A | A | 0.1148 |
| 2017 | 18-Sep-17 | MADERA | PISTACHIO (PISTACHE NUT) | Redacted4 | 17.2165 | 75 | A | A | 0.1148 |
| 2017 | 16-Sep-17 | MADERA | PISTACHIO (PISTACHE NUT) | Redacted4 | 17.2165 | 75 | A | A | 0.1148 |
| 2017 | 18-Sep-17 | MADERA | PISTACHIO (PISTACHE NUT) | Redacted4 | 17.2165 | 75 | A | A | 0.1148 |
| 2017 | 7-Aug-17 | MADERA | PISTACHIO (PISTACHE NUT) | Redacted4 | 27.5464 | 120 | A | A | 0.1148 |
| 2017 | 10-Aug-17 | MADERA | PISTACHIO (PISTACHE NUT) | Redacted4 | 17.2165 | 75 | A | A | 0.1148 |
| 2017 | 7-Aug-17 | MADERA | PISTACHIO (PISTACHE NUT) | Redacted4 | 17.2165 | 75 | A | A | 0.1148 |
| 2017 | 9-Aug-17 | FRESNO | PISTACHIO (PISTACHE NUT) | Redacted4 | 51.6496 | 225 | A | G | 0.1148 |
| 2017 | 4-May-17 | FRESNO | PISTACHIO (PISTACHE NUT) | Redacted4 | 51.6496 | 225 | A | G | 0.1148 |
| 2017 | 7-Aug-17 | MADERA | PISTACHIO (PISTACHE NUT) | Redacted4 | 74.157 | 323 | A | A | 0.1148 |
| 2017 | 20-Sep-17 | FRESNO | PISTACHIO (PISTACHE NUT) | Redacted4 | 101.9554 | 443.8 | A | A | 0.1149 |
| 2017 | 29-Jul-17 | FRESNO | PISTACHIO (PISTACHE NUT) | Redacted4 | 28.7222 | 125 | A | A | 0.1149 |
| 2017 | 7-Aug-17 | MADERA | PISTACHIO (PISTACHE NUT) | Redacted4 | 61.8115 | 269 | A | A | 0.1149 |
| 2017 | 7-Aug-17 | MADERA | PISTACHIO (PISTACHE NUT) | Redacted4 | 36.7846 | 160 | A | A | 0.1150 |
| 2017 | 7-Aug-17 | MADERA | PISTACHIO (PISTACHE NUT) | Redacted4 | 18.3923 | 80 | A | A | 0.1150 |
| 2017 | 7-Aug-17 | MADERA | PISTACHIO (PISTACHE NUT) | Redacted4 | 18.3923 | 80 | A | A | 0.1150 |
| 2017 | 7-Aug-17 | MADERA | PISTACHIO (PISTACHE NUT) | Redacted4 | 18.3923 | 80 | A | A | 0.1150 |
| 2017 | 16-Sep-17 | MADERA | PISTACHIO (PISTACHE NUT) | Redacted4 | 18.3923 | 80 | A | A | 0.1150 |
| 2017 | 20-Sep-17 | FRESNO | PISTACHIO (PISTACHE NUT) | Redacted4 | 19.736 | 85.8 | A | A | 0.1150 |
| 2017 | 10-Aug-17 | MADERA | PISTACHIO (PISTACHE NUT) | Redacted4 | 12.4295 | 54 | A | A | 0.1151 |
| 2017 | 28-Aug-17 | MADERA | PISTACHIO (PISTACHE NUT) | Redacted4 | 1.1758 | 5 | A | A | 0.1176 |
| 2017 | 4-Apr-17 | FRESNO | PISTACHIO (PISTACHE NUT) | Redacted4 | 28.2183 | 117.2 | A | G | 0.1204 |
| 2017 | 4-Apr-17 | FRESNO | PISTACHIO (PISTACHE NUT) | Redacted4 | 20.2399 | 84 | A | G | 0.1205 |
| 2017 | 4-Apr-17 | FRESNO | PISTACHIO (PISTACHE NUT) | Redacted4 | 37.3725 | 155 | A | G | 0.1206 |
| 2017 | 4-Apr-17 | FRESNO | PISTACHIO (PISTACHE NUT) | Redacted4 | 16.9646 | 70.3 | A | G | 0.1207 |
| 2017 | 4-Apr-17 | FRESNO | PISTACHIO (PISTACHE NUT) | Redacted4 | 14.3611 | 59.5 | A | G | 0.1207 |
| 2017 | 17-Jun-17 | FRESNO | PISTACHIO (PISTACHE NUT) | Redacted4 | 41.9915 | 160 | A | A | 0.1312 |
| 2017 | 18-Aug-17 | FRESNO | PISTACHIO (PISTACHE NUT) | Redacted4 | 50.8098 | 193.6 | A | G | 0.1312 |
| 2017 | 28-Jul-17 | FRESNO | PISTACHIO (PISTACHE NUT) | Redacted4 | 50.8098 | 193.6 | A | G | 0.1312 |
| 2017 | 27-Jul-17 | FRESNO | PISTACHIO (PISTACHE NUT) | Redacted4 | 7.9239 | 30 | A | G | 0.1321 |
| 2017 | 27-Jul-17 | FRESNO | PISTACHIO (PISTACHE NUT) | Redacted4 | 21.1309 | 80 | A | G | 0.1321 |
| 2017 | 27-Jul-17 | FRESNO | PISTACHIO (PISTACHE NUT) | Redacted4 | 11.8862 | 45 | A | G | 0.1321 |
| 2017 | 17-Aug-17 | FRESNO | PISTACHIO (PISTACHE NUT) | Redacted4 | 8 | 30 | A | G | 0.1333 |
| 2017 | 17-Aug-17 | FRESNO | PISTACHIO (PISTACHE NUT) | Redacted4 | 12.0004 | 45 | A | G | 0.1333 |
| 2017 | 17-Aug-17 | FRESNO | PISTACHIO (PISTACHE NUT) | Redacted4 | 21.3343 | 80 | A | G | 0.1333 |
| 2017 | 7-Aug-17 | KERN | PISTACHIO (PISTACHE NUT) | Redacted4 | 1.7636 | 6.6 | A | G | 0.1336 |
| 2017 | 4-Aug-17 | KERN | PISTACHIO (PISTACHE NUT) | Redacted4 | 20.1559 | 75 | A | G | 0.1344 |
| 2017 | 5-Aug-17 | KERN | PISTACHIO (PISTACHE NUT) | Redacted4 | 41.6556 | 155 | A | G | 0.1344 |
| 2017 | 5-Jun-17 | FRESNO | PISTACHIO (PISTACHE NUT) | Redacted4 | 30.2339 | 84 | A | G | 0.1800 |
| 2017 | 6-Jun-17 | FRESNO | PISTACHIO (PISTACHE NUT) | Redacted4 | 21.4157 | 59.5 | A | G | 0.1800 |
| 2017 | 5-Jun-17 | FRESNO | PISTACHIO (PISTACHE NUT) | Redacted4 | 43.5872 | 121 | A | G | 0.1801 |
| 2017 | 6-Jun-17 | FRESNO | PISTACHIO (PISTACHE NUT) | Redacted4 | 42.2435 | 117.2 | A | G | 0.1802 |
| 2017 | 5-Jun-17 | FRESNO | PISTACHIO (PISTACHE NUT) | Redacted4 | 81.1276 | 225 | A | G | 0.1803 |
| 2017 | 5-Jun-17 | FRESNO | PISTACHIO (PISTACHE NUT) | Redacted4 | 25.3629 | 70.3 | A | G | 0.1804 |
| 2017 | 5-Jun-17 | FRESNO | PISTACHIO (PISTACHE NUT) | Redacted4 | 55.9327 | 155 | A | G | 0.1804 |
| 2017 | 3-Jun-17 | FRESNO | PLUM (INCLUDES WILD PLUMS FOR HUMAN CONSUMPTION) | Redacted4 | 9.1542 | 40 | A | G | 0.1144 |
| 2017 | 23-Jun-17 | KERN | POMEGRANATE (MISCELLANEOUS FRUIT) | Redacted4 | 5.4589 | 12 | A | G | 0.2275 |
| 2017 | 23-Oct-17 | KERN | POMEGRANATE (MISCELLANEOUS FRUIT) | Redacted4 | 5.4589 | 12 | A | G | 0.2275 |
| 2017 | 22-Oct-17 | KERN | POMEGRANATE (MISCELLANEOUS FRUIT) | Redacted4 | 37.8764 | 82.6 | A | G | 0.2293 |
| 2017 | 19-Sep-17 | KINGS | POMEGRANATE (MISCELLANEOUS FRUIT) | Redacted4 | 61.4756 | 134 | A | G | 0.2294 |
| 2017 | 21-Sep-17 | MADERA | POMEGRANATE (MISCELLANEOUS FRUIT) | Redacted4 | 48.9621 | 106.72 | A | G | 0.2294 |
| 2017 | 28-Jun-17 | MADERA | POMEGRANATE (MISCELLANEOUS FRUIT) | Redacted4 | 48.9621 | 106.72 | A | G | 0.2294 |
| 2017 | 22-Aug-17 | MADERA | POMEGRANATE (MISCELLANEOUS FRUIT) | Redacted4 | 48.9621 | 106.72 | A | G | 0.2294 |
| 2017 | 16-Jun-17 | KERN | POMEGRANATE (MISCELLANEOUS FRUIT) | Redacted4 | 18.8122 | 41 | A | G | 0.2294 |
| 2017 | 25-Aug-17 | KERN | POMEGRANATE (MISCELLANEOUS FRUIT) | Redacted4 | 18.8122 | 41 | A | G | 0.2294 |
| 2017 | 20-Sep-17 | KINGS | POMEGRANATE (MISCELLANEOUS FRUIT) | Redacted4 | 109.6819 | 239 | A | G | 0.2295 |
| 2017 | 16-Jun-17 | KERN | POMEGRANATE (MISCELLANEOUS FRUIT) | Redacted4 | 52.3214 | 114 | A | G | 0.2295 |
| 2017 | 19-Sep-17 | KERN | POMEGRANATE (MISCELLANEOUS FRUIT) | Redacted4 | 52.3214 | 114 | A | G | 0.2295 |
| 2017 | 31-Aug-17 | KERN | POMEGRANATE (MISCELLANEOUS FRUIT) | Redacted4 | 52.3214 | 114 | A | G | 0.2295 |
| 2017 | 19-Jun-17 | KERN | POMEGRANATE (MISCELLANEOUS FRUIT) | Redacted4 | 112.4533 | 245 | A | G | 0.2295 |
| 2017 | 27-Aug-17 | KERN | POMEGRANATE (MISCELLANEOUS FRUIT) | Redacted4 | 112.4533 | 245 | A | G | 0.2295 |
| 2017 | 25-Oct-17 | KERN | POMEGRANATE (MISCELLANEOUS FRUIT) | Redacted4 | 67.0185 | 146 | A | G | 0.2295 |
| 2017 | 19-Sep-17 | KINGS | POMEGRANATE (MISCELLANEOUS FRUIT) | Redacted4 | 67.0185 | 146 | A | G | 0.2295 |
| 2017 | 16-Jun-17 | KINGS | POMEGRANATE (MISCELLANEOUS FRUIT) | Redacted4 | 68.0263 | 148.18 | A | G | 0.2295 |
| 2017 | 10-Oct-17 | KINGS | POMEGRANATE (MISCELLANEOUS FRUIT) | Redacted4 | 68.0263 | 148.18 | A | G | 0.2295 |
| 2017 | 5-Sep-17 | KINGS | POMEGRANATE (MISCELLANEOUS FRUIT) | Redacted4 | 68.0263 | 148.18 | A | G | 0.2295 |
| 2017 | 31-Aug-17 | KERN | POMEGRANATE (MISCELLANEOUS FRUIT) | Redacted4 | 95.4887 | 208 | A | G | 0.2295 |
| 2017 | 22-Sep-17 | KERN | POMEGRANATE (MISCELLANEOUS FRUIT) | Redacted4 | 95.4887 | 208 | A | G | 0.2295 |
| 2017 | 22-Jun-17 | KERN | POMEGRANATE (MISCELLANEOUS FRUIT) | Redacted4 | 95.4887 | 208 | A | G | 0.2295 |
| 2017 | 27-Jun-17 | MADERA | POMEGRANATE (MISCELLANEOUS FRUIT) | Redacted4 | 112.7892 | 245.67 | A | G | 0.2296 |
| 2017 | 20-Sep-17 | MADERA | POMEGRANATE (MISCELLANEOUS FRUIT) | Redacted4 | 112.7892 | 245.67 | A | G | 0.2296 |
| 2017 | 21-Aug-17 | MADERA | POMEGRANATE (MISCELLANEOUS FRUIT) | Redacted4 | 112.7892 | 245.67 | A | G | 0.2296 |
| 2017 | 9-Oct-17 | KINGS | POMEGRANATE (MISCELLANEOUS FRUIT) | Redacted4 | 106.5745 | 232.13 | A | G | 0.2296 |
| 2017 | 19-Jun-17 | KERN | POMEGRANATE (MISCELLANEOUS FRUIT) | Redacted4 | 189.7177 | 413.22 | A | G | 0.2296 |
| 2017 | 21-Jun-17 | KINGS | POMEGRANATE (MISCELLANEOUS FRUIT) | Redacted4 | 65.0869 | 141.76 | A | G | 0.2296 |
| 2017 | 30-Aug-17 | KINGS | POMEGRANATE (MISCELLANEOUS FRUIT) | Redacted4 | 65.0869 | 141.76 | A | G | 0.2296 |
| 2017 | 21-Jun-17 | KINGS | POMEGRANATE (MISCELLANEOUS FRUIT) | Redacted4 | 59.712 | 130.05 | A | G | 0.2296 |
| 2017 | 30-Aug-17 | KINGS | POMEGRANATE (MISCELLANEOUS FRUIT) | Redacted4 | 59.712 | 130.05 | A | G | 0.2296 |
| 2017 | 19-Oct-17 | KERN | POMEGRANATE (MISCELLANEOUS FRUIT) | Redacted4 | 188.7099 | 411 | A | G | 0.2296 |
| 2017 | 31-Aug-17 | KERN | POMEGRANATE (MISCELLANEOUS FRUIT) | Redacted4 | 188.7099 | 411 | A | G | 0.2296 |
| 2017 | 31-Aug-17 | KINGS | POMEGRANATE (MISCELLANEOUS FRUIT) | Redacted4 | 106.3226 | 231.56 | A | G | 0.2296 |
| 2017 | 22-Jun-17 | KINGS | POMEGRANATE (MISCELLANEOUS FRUIT) | Redacted4 | 106.3226 | 231.56 | A | G | 0.2296 |
| 2017 | 21-Jun-17 | KERN | POMEGRANATE (MISCELLANEOUS FRUIT) | Redacted4 | 98.2602 | 214 | A | G | 0.2296 |
| 2017 | 19-Sep-17 | KERN | POMEGRANATE (MISCELLANEOUS FRUIT) | Redacted4 | 98.2602 | 214 | A | G | 0.2296 |
| 2017 | 26-Aug-17 | KERN | POMEGRANATE (MISCELLANEOUS FRUIT) | Redacted4 | 98.2602 | 214 | A | G | 0.2296 |
| 2017 | 20-Jun-17 | KERN | POMEGRANATE (MISCELLANEOUS FRUIT) | Redacted4 | 183.671 | 400 | A | G | 0.2296 |
| 2017 | 22-Aug-17 | MADERA | POMEGRANATE (MISCELLANEOUS FRUIT) | Redacted4 | 170.0657 | 370.37 | A | G | 0.2296 |
| 2017 | 22-Sep-17 | MADERA | POMEGRANATE (MISCELLANEOUS FRUIT) | Redacted4 | 170.0657 | 370.37 | A | G | 0.2296 |
| 2017 | 28-Jun-17 | MADERA | POMEGRANATE (MISCELLANEOUS FRUIT) | Redacted4 | 170.0657 | 370.37 | A | G | 0.2296 |
| 2017 | 29-Aug-17 | KERN | POMEGRANATE (MISCELLANEOUS FRUIT) | Redacted4 | 106.0706 | 231 | A | G | 0.2296 |
| 2017 | 28-Aug-17 | KERN | POMEGRANATE (MISCELLANEOUS FRUIT) | Redacted4 | 99.184 | 216 | A | G | 0.2296 |
| 2017 | 16-Jun-17 | KERN | POMEGRANATE (MISCELLANEOUS FRUIT) | Redacted4 | 99.184 | 216 | A | G | 0.2296 |
| 2017 | 21-Sep-17 | KERN | POMEGRANATE (MISCELLANEOUS FRUIT) | Redacted4 | 99.184 | 216 | A | G | 0.2296 |
| 2017 | 23-Jun-17 | KERN | POMEGRANATE (MISCELLANEOUS FRUIT) | Redacted4 | 226.8382 | 494 | A | G | 0.2296 |
| 2017 | 31-Aug-17 | KERN | POMEGRANATE (MISCELLANEOUS FRUIT) | Redacted4 | 191.4814 | 417 | A | G | 0.2296 |
| 2017 | 22-Jun-17 | KERN | POMEGRANATE (MISCELLANEOUS FRUIT) | Redacted4 | 191.4814 | 417 | A | G | 0.2296 |
| 2017 | 21-Sep-17 | KERN | POMEGRANATE (MISCELLANEOUS FRUIT) | Redacted4 | 191.4814 | 417 | A | G | 0.2296 |
| 2017 | 23-Jun-17 | KERN | POMEGRANATE (MISCELLANEOUS FRUIT) | Redacted4 | 68.5302 | 149.24 | A | G | 0.2296 |
| 2017 | 7-Oct-17 | KINGS | POMEGRANATE (MISCELLANEOUS FRUIT) | Redacted4 | 173.425 | 377.67 | A | G | 0.2296 |
| 2017 | 30-Aug-17 | KERN | POMEGRANATE (MISCELLANEOUS FRUIT) | Redacted4 | 292.513 | 637 | A | G | 0.2296 |
| 2017 | 18-Oct-17 | KERN | POMEGRANATE (MISCELLANEOUS FRUIT) | Redacted4 | 68.5302 | 149.23 | A | G | 0.2296 |
| 2017 | 20-Sep-17 | KERN | POMEGRANATE (MISCELLANEOUS FRUIT) | Redacted4 | 194.2528 | 423 | A | G | 0.2296 |
| 2017 | 17-Jun-17 | KERN | POMEGRANATE (MISCELLANEOUS FRUIT) | Redacted4 | 194.2528 | 423 | A | G | 0.2296 |
| 2017 | 27-Aug-17 | KERN | POMEGRANATE (MISCELLANEOUS FRUIT) | Redacted4 | 194.2528 | 423 | A | G | 0.2296 |
| 2017 | 29-Jun-17 | MADERA | POMEGRANATE (MISCELLANEOUS FRUIT) | Redacted4 | 183.8389 | 400.32 | A | G | 0.2296 |
| 2017 | 23-Aug-17 | MADERA | POMEGRANATE (MISCELLANEOUS FRUIT) | Redacted4 | 183.8389 | 400.32 | A | G | 0.2296 |
| 2017 | 23-Sep-17 | MADERA | POMEGRANATE (MISCELLANEOUS FRUIT) | Redacted4 | 183.8389 | 400.32 | A | G | 0.2296 |
| 2017 | 23-Sep-17 | MADERA | POMEGRANATE (MISCELLANEOUS FRUIT) | Redacted4 | 143.611 | 312.72 | A | G | 0.2296 |
| 2017 | 30-Jun-17 | MADERA | POMEGRANATE (MISCELLANEOUS FRUIT) | Redacted4 | 143.611 | 312.72 | A | G | 0.2296 |
| 2017 | 24-Aug-17 | MADERA | POMEGRANATE (MISCELLANEOUS FRUIT) | Redacted4 | 143.611 | 312.72 | A | G | 0.2296 |
| 2017 | 20-Oct-17 | KERN | POMEGRANATE (MISCELLANEOUS FRUIT) | Redacted4 | 195.1766 | 425 | A | G | 0.2296 |
| 2017 | 29-Aug-17 | KERN | POMEGRANATE (MISCELLANEOUS FRUIT) | Redacted4 | 195.1766 | 425 | A | G | 0.2296 |
| 2017 | 25-Oct-17 | KERN | POMEGRANATE (MISCELLANEOUS FRUIT) | Redacted4 | 159.8198 | 348 | A | G | 0.2296 |
| 2017 | 17-Jun-17 | KINGS | POMEGRANATE (MISCELLANEOUS FRUIT) | Redacted4 | 142.0993 | 309.4 | A | G | 0.2296 |
| 2017 | 6-Sep-17 | KINGS | POMEGRANATE (MISCELLANEOUS FRUIT) | Redacted4 | 142.0993 | 309.4 | A | G | 0.2296 |
| 2017 | 29-Aug-17 | KERN | POMEGRANATE (MISCELLANEOUS FRUIT) | Redacted4 | 29.3941 | 64 | A | G | 0.2296 |
| 2017 | 15-Jun-17 | KERN | POMEGRANATE (MISCELLANEOUS FRUIT) | Redacted4 | 2.7557 | 6 | A | G | 0.2296 |
| 2017 | 21-Jun-17 | KERN | POMEGRANATE (MISCELLANEOUS FRUIT) | Redacted4 | 283.9467 | 618.23 | A | G | 0.2296 |
| 2017 | 3-Sep-17 | KINGS | POMEGRANATE (MISCELLANEOUS FRUIT) | Redacted4 | 209.0338 | 455.12 | A | G | 0.2296 |
| 2017 | 20-Jun-17 | KINGS | POMEGRANATE (MISCELLANEOUS FRUIT) | Redacted4 | 209.0338 | 455.12 | A | G | 0.2296 |
| 2017 | 22-Oct-17 | KERN | POMEGRANATE (MISCELLANEOUS FRUIT) | Redacted4 | 281.0913 | 612 | A | G | 0.2296 |
| 2017 | 19-Jun-17 | KERN | POMEGRANATE (MISCELLANEOUS FRUIT) | Redacted4 | 281.0913 | 612 | A | G | 0.2296 |
| 2017 | 27-Aug-17 | KERN | POMEGRANATE (MISCELLANEOUS FRUIT) | Redacted4 | 281.0913 | 612 | A | G | 0.2296 |
| 2017 | 29-Aug-17 | KERN | POMEGRANATE (MISCELLANEOUS FRUIT) | Redacted4 | 289.8255 | 631 | A | G | 0.2297 |
| 2017 | 27-Oct-17 | KERN | POMEGRANATE (MISCELLANEOUS FRUIT) | Redacted4 | 289.8255 | 631 | A | G | 0.2297 |
| 2017 | 23-Jun-17 | KERN | POMEGRANATE (MISCELLANEOUS FRUIT) | Redacted4 | 289.8255 | 631 | A | G | 0.2297 |
| 2017 | 26-Aug-17 | KERN | POMEGRANATE (MISCELLANEOUS FRUIT) | Redacted4 | 45.0149 | 98 | A | G | 0.2297 |
| 2017 | 19-Jun-17 | KERN | POMEGRANATE (MISCELLANEOUS FRUIT) | Redacted4 | 45.0149 | 98 | A | G | 0.2297 |
| 2017 | 7-Sep-17 | KINGS | POMEGRANATE (MISCELLANEOUS FRUIT) | Redacted4 | 110.6057 | 240.79 | A | G | 0.2297 |
| 2017 | 17-Jun-17 | KINGS | POMEGRANATE (MISCELLANEOUS FRUIT) | Redacted4 | 110.6057 | 240.79 | A | G | 0.2297 |
| 2017 | 9-Oct-17 | KINGS | POMEGRANATE (MISCELLANEOUS FRUIT) | Redacted4 | 110.6057 | 240.79 | A | G | 0.2297 |
| 2017 | 19-Sep-17 | MADERA | POMEGRANATE (MISCELLANEOUS FRUIT) | Redacted4 | 196.6043 | 428 | A | G | 0.2297 |
| 2017 | 27-Aug-17 | KERN | POMEGRANATE (MISCELLANEOUS FRUIT) | Redacted4 | 226.9222 | 494 | A | G | 0.2297 |
| 2017 | 20-Jun-17 | KERN | POMEGRANATE (MISCELLANEOUS FRUIT) | Redacted4 | 102.7953 | 223.73 | A | G | 0.2297 |
| 2017 | 18-Jun-17 | KINGS | POMEGRANATE (MISCELLANEOUS FRUIT) | Redacted4 | 67.7743 | 147.49 | A | G | 0.2298 |
| 2017 | 31-Aug-17 | KINGS | POMEGRANATE (MISCELLANEOUS FRUIT) | Redacted4 | 67.7743 | 147.49 | A | G | 0.2298 |
| 2017 | 20-Jun-17 | KERN | POMEGRANATE (MISCELLANEOUS FRUIT) | Redacted4 | 28.3023 | 61.57 | A | G | 0.2298 |
| 2017 | 16-Sep-17 | KINGS | POMEGRANATE (MISCELLANEOUS FRUIT) | Redacted4 | 35.8608 | 78 | A | G | 0.2299 |
| 2017 | 16-Sep-17 | KINGS | POMEGRANATE (MISCELLANEOUS FRUIT) | Redacted4 | 35.8608 | 78 | A | G | 0.2299 |
| 2017 | 25-Aug-17 | KERN | POMEGRANATE (MISCELLANEOUS FRUIT) | Redacted4 | 5.5429 | 12 | A | G | 0.2310 |
| 2017 | 11-Aug-17 | SAN JOAQUIN | PUMPKIN | Redacted4 | 4.7031 | 47.78 | A | G | 0.0492 |
| 2017 | 10-Aug-17 | SANTA CLARA | PUMPKIN | Redacted4 | 2.6035 | 20 | A | A | 0.0651 |
| 2017 | 19-Jul-17 | SANTA CLARA | PUMPKIN | Redacted4 | 2.4355 | 15.1 | A | G | 0.0806 |
| 2017 | 12-Jul-17 | SANTA CLARA | PUMPKIN | Redacted4 | 2.4355 | 15.1 | A | G | 0.0806 |
| 2017 | 8-Jul-17 | SANTA CLARA | PUMPKIN | Redacted4 | 2.2675 | 14 | A | G | 0.0810 |
| 2017 | 19-Jul-17 | SANTA CLARA | PUMPKIN | Redacted4 | 2.2675 | 14 | A | G | 0.0810 |
| 2017 | 8-Jul-17 | SANTA CLARA | PUMPKIN | Redacted4 | 6.0468 | 37 | A | G | 0.0817 |
| 2017 | 1-Jul-17 | SANTA CLARA | PUMPKIN | Redacted4 | 9.4901 | 58 | A | G | 0.0818 |
| 2017 | 28-Jun-17 | SANTA CLARA | PUMPKIN | Redacted4 | 3.2753 | 20 | A | G | 0.0819 |
| 2017 | 9-Jul-17 | SANTA CLARA | PUMPKIN | Redacted4 | 3.2753 | 20 | A | G | 0.0819 |
| 2017 | 29-Jul-17 | SANTA CLARA | PUMPKIN | Redacted4 | 4.787 | 29.2 | A | G | 0.0820 |
| 2017 | 15-Jul-17 | SANTA CLARA | PUMPKIN | Redacted4 | 3.4433 | 21 | A | G | 0.0820 |
| 2017 | 11-Jul-17 | SANTA CLARA | PUMPKIN | Redacted4 | 5.8788 | 35.6 | A | G | 0.0826 |
| 2017 | 18-Jul-17 | SANTA CLARA | PUMPKIN | Redacted4 | 5.8788 | 35.6 | A | G | 0.0826 |
| 2017 | 20-Jul-17 | SANTA CLARA | PUMPKIN | Redacted4 | 1.3437 | 7 | A | G | 0.0960 |
| 2017 | 17-Jul-17 | VENTURA | PUMPKIN | Redacted4 | 0.168 | 0.75 | A | G | 0.1120 |
| 2017 | 23-Aug-17 | SANTA CLARA | PUMPKIN | Redacted4 | 11.0018 | 42 | A | G | 0.1310 |
| 2017 | 4-Sep-17 | SANTA CLARA | PUMPKIN | Redacted4 | 0.6719 | 2.5 | A | G | 0.1344 |
| 2017 | 17-Jul-17 | VENTURA | PUMPKIN | Redacted4 | 0.4199 | 1.5 | A | G | 0.1400 |
| 2017 | 24-Aug-17 | SANTA CLARA | PUMPKIN | Redacted4 | 0.6719 | 2.4 | A | G | 0.1400 |
| 2017 | 8-Jul-17 | VENTURA | RASPBERRY (ALL OR UNSPEC) | Redacted4 | 0.5577 | 4.25 | A | G | 0.0656 |
| 2017 | 8-Jul-17 | VENTURA | RASPBERRY (ALL OR UNSPEC) | Redacted4 | 1.5091 | 11.5 | A | G | 0.0656 |
| 2017 | 8-Mar-17 | VENTURA | RASPBERRY (ALL OR UNSPEC) | Redacted4 | 0.4396 | 2.69 | A | G | 0.0817 |
| 2017 | 27-May-17 | VENTURA | RASPBERRY (ALL OR UNSPEC) | Redacted4 | 5.5967 | 34.13 | A | G | 0.0820 |
| 2017 | 16-Mar-17 | VENTURA | RASPBERRY (ALL OR UNSPEC) | Redacted4 | 0.5577 | 3.4 | A | G | 0.0820 |
| 2017 | 18-Mar-17 | VENTURA | RASPBERRY (ALL OR UNSPEC) | Redacted4 | 0.5577 | 3.4 | A | G | 0.0820 |
| 2017 | 8-Mar-17 | VENTURA | RASPBERRY (ALL OR UNSPEC) | Redacted4 | 1.3057 | 7.96 | A | G | 0.0820 |
| 2017 | 8-Mar-17 | VENTURA | RASPBERRY (ALL OR UNSPEC) | Redacted4 | 1.8831 | 11.46 | A | G | 0.0822 |
| 2017 | 9-Mar-17 | VENTURA | RASPBERRY (ALL OR UNSPEC) | Redacted4 | 1.7125 | 10.42 | A | G | 0.0822 |
| 2017 | 9-Mar-17 | VENTURA | RASPBERRY (ALL OR UNSPEC) | Redacted4 | 1.66 | 10.1 | A | G | 0.0822 |
| 2017 | 11-Nov-17 | VENTURA | RASPBERRY (ALL OR UNSPEC) | Redacted4 | 0.4199 | 1.99 | A | G | 0.1055 |
| 2017 | 25-May-17 | VENTURA | RASPBERRY (ALL OR UNSPEC) | Redacted4 | 0.5039 | 2.36 | A | G | 0.1068 |
| 2017 | 24-Jul-17 | VENTURA | RASPBERRY (ALL OR UNSPEC) | Redacted4 | 0.168 | 0.78 | A | G | 0.1077 |
| 2017 | 24-Apr-17 | VENTURA | RASPBERRY (ALL OR UNSPEC) | Redacted4 | 1.1548 | 5.3 | A | G | 0.1089 |
| 2017 | 20-Apr-17 | VENTURA | RASPBERRY (ALL OR UNSPEC) | Redacted4 | 1.1548 | 5.3 | A | G | 0.1089 |
| 2017 | 8-Apr-17 | VENTURA | RASPBERRY (ALL OR UNSPEC) | Redacted4 | 3.2871 | 15.06 | A | G | 0.1091 |
| 2017 | 1-Aug-17 | VENTURA | RASPBERRY (ALL OR UNSPEC) | Redacted4 | 1.3122 | 6 | A | G | 0.1094 |
| 2017 | 14-Apr-17 | VENTURA | RASPBERRY (ALL OR UNSPEC) | Redacted4 | 2.5129 | 11.49 | A | G | 0.1094 |
| 2017 | 28-Dec-17 | VENTURA | RASPBERRY (ALL OR UNSPEC) | Redacted4 | 1.555 | 7.1 | A | G | 0.1095 |
| 2017 | 28-Dec-17 | VENTURA | RASPBERRY (ALL OR UNSPEC) | Redacted4 | 0.8661 | 3.95 | A | G | 0.1096 |
| 2017 | 1-Aug-17 | VENTURA | RASPBERRY (ALL OR UNSPEC) | Redacted4 | 0.7677 | 3.5 | A | G | 0.1097 |
| 2017 | 26-Jul-17 | VENTURA | RASPBERRY (ALL OR UNSPEC) | Redacted4 | 1.3437 | 6.03 | A | G | 0.1114 |
| 2017 | 7-Nov-17 | VENTURA | RASPBERRY (ALL OR UNSPEC) | Redacted4 | 1.3437 | 6.03 | A | G | 0.1114 |
| 2017 | 4-Oct-17 | VENTURA | RASPBERRY (ALL OR UNSPEC) | Redacted4 | 0.6719 | 2.99 | A | G | 0.1124 |
| 2017 | 26-Jul-17 | VENTURA | RASPBERRY (ALL OR UNSPEC) | Redacted4 | 1.0918 | 4.85 | A | G | 0.1126 |
| 2017 | 1-Nov-17 | VENTURA | RASPBERRY (ALL OR UNSPEC) | Redacted4 | 1.0918 | 4.85 | A | G | 0.1126 |
| 2017 | 4-Nov-17 | VENTURA | RASPBERRY (ALL OR UNSPEC) | Redacted4 | 1.4277 | 6.3 | A | G | 0.1133 |
| 2017 | 20-Oct-17 | VENTURA | RASPBERRY (ALL OR UNSPEC) | Redacted4 | 1.6797 | 7.37 | A | G | 0.1140 |
| 2017 | 6-Jul-17 | VENTURA | RASPBERRY (ALL OR UNSPEC) | Redacted4 | 1.5957 | 7 | A | G | 0.1140 |
| 2017 | 4-Aug-17 | VENTURA | RASPBERRY (ALL OR UNSPEC) | Redacted4 | 1.5957 | 7 | A | G | 0.1140 |
| 2017 | 31-May-17 | VENTURA | RASPBERRY (ALL OR UNSPEC) | Redacted4 | 0.3674 | 1.61 | A | G | 0.1141 |
| 2017 | 22-Jul-17 | VENTURA | RASPBERRY (ALL OR UNSPEC) | Redacted4 | 1.2597 | 5.52 | A | G | 0.1141 |
| 2017 | 19-May-17 | VENTURA | RASPBERRY (ALL OR UNSPEC) | Redacted4 | 1.0078 | 4.41 | A | G | 0.1143 |
| 2017 | 6-Jun-17 | VENTURA | RASPBERRY (ALL OR UNSPEC) | Redacted4 | 1.0078 | 4.41 | A | G | 0.1143 |
| 2017 | 22-Aug-17 | VENTURA | RASPBERRY (ALL OR UNSPEC) | Redacted4 | 0.6036 | 2.64 | A | G | 0.1143 |
| 2017 | 23-Dec-17 | VENTURA | RASPBERRY (ALL OR UNSPEC) | Redacted4 | 0.8726 | 3.81 | A | G | 0.1145 |
| 2017 | 17-Aug-17 | VENTURA | RASPBERRY (ALL OR UNSPEC) | Redacted4 | 0.6299 | 2.75 | A | G | 0.1145 |
| 2017 | 17-Apr-17 | VENTURA | RASPBERRY (ALL OR UNSPEC) | Redacted4 | 0.6299 | 2.75 | A | G | 0.1145 |
| 2017 | 12-Oct-17 | VENTURA | RASPBERRY (ALL OR UNSPEC) | Redacted4 | 1.391 | 6.07 | A | G | 0.1146 |
| 2017 | 27-Mar-17 | VENTURA | RASPBERRY (ALL OR UNSPEC) | Redacted4 | 0.9579 | 4.18 | A | G | 0.1146 |
| 2017 | 21-Apr-17 | VENTURA | RASPBERRY (ALL OR UNSPEC) | Redacted4 | 0.9579 | 4.18 | A | G | 0.1146 |
| 2017 | 4-Apr-17 | VENTURA | RASPBERRY (ALL OR UNSPEC) | Redacted4 | 0.9579 | 4.18 | A | G | 0.1146 |
| 2017 | 25-Apr-17 | VENTURA | RASPBERRY (ALL OR UNSPEC) | Redacted4 | 0.9579 | 4.18 | A | G | 0.1146 |
| 2017 | 24-Aug-17 | VENTURA | RASPBERRY (ALL OR UNSPEC) | Redacted4 | 1.5747 | 6.87 | A | G | 0.1146 |
| 2017 | 22-Aug-17 | VENTURA | RASPBERRY (ALL OR UNSPEC) | Redacted4 | 0.9054 | 3.95 | A | G | 0.1146 |
| 2017 | 20-Jul-17 | VENTURA | RASPBERRY (ALL OR UNSPEC) | Redacted4 | 1.0892 | 4.75 | A | G | 0.1147 |
| 2017 | 14-Apr-17 | VENTURA | RASPBERRY (ALL OR UNSPEC) | Redacted4 | 1.0892 | 4.75 | A | G | 0.1147 |
| 2017 | 12-Jul-17 | VENTURA | RASPBERRY (ALL OR UNSPEC) | Redacted4 | 1.0892 | 4.75 | A | G | 0.1147 |
| 2017 | 31-May-17 | VENTURA | RASPBERRY (ALL OR UNSPEC) | Redacted4 | 0.4724 | 2.06 | A | G | 0.1147 |
| 2017 | 20-Apr-17 | VENTURA | RASPBERRY (ALL OR UNSPEC) | Redacted4 | 0.4724 | 2.06 | A | G | 0.1147 |
| 2017 | 20-Jul-17 | VENTURA | RASPBERRY (ALL OR UNSPEC) | Redacted4 | 2.3095 | 10.07 | A | G | 0.1147 |
| 2017 | 26-May-17 | VENTURA | RASPBERRY (ALL OR UNSPEC) | Redacted4 | 1.8371 | 8.01 | A | G | 0.1147 |
| 2017 | 12-May-17 | VENTURA | RASPBERRY (ALL OR UNSPEC) | Redacted4 | 1.8371 | 8.01 | A | G | 0.1147 |
| 2017 | 18-May-17 | VENTURA | RASPBERRY (ALL OR UNSPEC) | Redacted4 | 1.8371 | 8.01 | A | G | 0.1147 |
| 2017 | 20-Jul-17 | VENTURA | RASPBERRY (ALL OR UNSPEC) | Redacted4 | 1.555 | 6.78 | A | G | 0.1147 |
| 2017 | 24-Aug-17 | VENTURA | RASPBERRY (ALL OR UNSPEC) | Redacted4 | 1.555 | 6.78 | A | G | 0.1147 |
| 2017 | 10-May-17 | VENTURA | RASPBERRY (ALL OR UNSPEC) | Redacted4 | 1.555 | 6.78 | A | G | 0.1147 |
| 2017 | 28-Dec-17 | VENTURA | RASPBERRY (ALL OR UNSPEC) | Redacted4 | 1.555 | 6.78 | A | G | 0.1147 |
| 2017 | 3-Aug-17 | VENTURA | RASPBERRY (ALL OR UNSPEC) | Redacted4 | 1.555 | 6.78 | A | G | 0.1147 |
| 2017 | 20-Jul-17 | VENTURA | RASPBERRY (ALL OR UNSPEC) | Redacted4 | 1.0826 | 4.72 | A | G | 0.1147 |
| 2017 | 17-Apr-17 | VENTURA | RASPBERRY (ALL OR UNSPEC) | Redacted4 | 0.9382 | 4.09 | A | G | 0.1147 |
| 2017 | 25-Mar-17 | VENTURA | RASPBERRY (ALL OR UNSPEC) | Redacted4 | 0.9382 | 4.09 | A | G | 0.1147 |
| 2017 | 21-Apr-17 | VENTURA | RASPBERRY (ALL OR UNSPEC) | Redacted4 | 0.9382 | 4.09 | A | G | 0.1147 |
| 2017 | 11-Apr-17 | VENTURA | RASPBERRY (ALL OR UNSPEC) | Redacted4 | 2.6245 | 11.44 | A | G | 0.1147 |
| 2017 | 18-Jul-17 | VENTURA | RASPBERRY (ALL OR UNSPEC) | Redacted4 | 1.7781 | 7.75 | A | G | 0.1147 |
| 2017 | 29-Jun-17 | VENTURA | RASPBERRY (ALL OR UNSPEC) | Redacted4 | 1.7781 | 7.75 | A | G | 0.1147 |
| 2017 | 1-Jun-17 | VENTURA | RASPBERRY (ALL OR UNSPEC) | Redacted4 | 3.4118 | 14.87 | A | G | 0.1147 |
| 2017 | 8-May-17 | VENTURA | RASPBERRY (ALL OR UNSPEC) | Redacted4 | 2.7032 | 11.78 | A | G | 0.1147 |
| 2017 | 24-Aug-17 | VENTURA | RASPBERRY (ALL OR UNSPEC) | Redacted4 | 2.3751 | 10.35 | A | G | 0.1147 |
| 2017 | 24-Jun-17 | VENTURA | RASPBERRY (ALL OR UNSPEC) | Redacted4 | 2.3751 | 10.35 | A | G | 0.1147 |
| 2017 | 17-May-17 | VENTURA | RASPBERRY (ALL OR UNSPEC) | Redacted4 | 2.3751 | 10.35 | A | G | 0.1147 |
| 2017 | 9-May-17 | VENTURA | RASPBERRY (ALL OR UNSPEC) | Redacted4 | 0.9776 | 4.26 | A | G | 0.1147 |
| 2017 | 3-May-17 | VENTURA | RASPBERRY (ALL OR UNSPEC) | Redacted4 | 0.9776 | 4.26 | A | G | 0.1147 |
| 2017 | 4-Apr-17 | VENTURA | RASPBERRY (ALL OR UNSPEC) | Redacted4 | 0.5118 | 2.23 | A | G | 0.1148 |
| 2017 | 26-Dec-17 | VENTURA | RASPBERRY (ALL OR UNSPEC) | Redacted4 | 3.5758 | 15.58 | A | G | 0.1148 |
| 2017 | 1-Aug-17 | VENTURA | RASPBERRY (ALL OR UNSPEC) | Redacted4 | 3.2871 | 14.32 | A | G | 0.1148 |
| 2017 | 16-Jun-17 | VENTURA | RASPBERRY (ALL OR UNSPEC) | Redacted4 | 3.2871 | 14.32 | A | G | 0.1148 |
| 2017 | 17-Jul-17 | VENTURA | RASPBERRY (ALL OR UNSPEC) | Redacted4 | 3.2871 | 14.32 | A | G | 0.1148 |
| 2017 | 22-Jul-17 | VENTURA | RASPBERRY (ALL OR UNSPEC) | Redacted4 | 3.5627 | 15.52 | A | G | 0.1148 |
| 2017 | 17-Jul-17 | VENTURA | RASPBERRY (ALL OR UNSPEC) | Redacted4 | 3.5627 | 15.52 | A | G | 0.1148 |
| 2017 | 3-Sep-17 | VENTURA | RASPBERRY (ALL OR UNSPEC) | Redacted4 | 3.5627 | 15.52 | A | G | 0.1148 |
| 2017 | 13-May-17 | VENTURA | RASPBERRY (ALL OR UNSPEC) | Redacted4 | 2.6376 | 11.49 | A | G | 0.1148 |
| 2017 | 30-Apr-17 | VENTURA | RASPBERRY (ALL OR UNSPEC) | Redacted4 | 2.0799 | 9.06 | A | G | 0.1148 |
| 2017 | 27-May-17 | VENTURA | RASPBERRY (ALL OR UNSPEC) | Redacted4 | 2.0799 | 9.06 | A | G | 0.1148 |
| 2017 | 31-May-17 | VENTURA | RASPBERRY (ALL OR UNSPEC) | Redacted4 | 2.631 | 11.46 | A | G | 0.1148 |
| 2017 | 6-May-17 | VENTURA | RASPBERRY (ALL OR UNSPEC) | Redacted4 | 2.631 | 11.46 | A | G | 0.1148 |
| 2017 | 16-May-17 | VENTURA | RASPBERRY (ALL OR UNSPEC) | Redacted4 | 2.631 | 11.46 | A | G | 0.1148 |
| 2017 | 3-May-17 | VENTURA | RASPBERRY (ALL OR UNSPEC) | Redacted4 | 3.4577 | 15.06 | A | G | 0.1148 |
| 2017 | 20-Jul-17 | VENTURA | RASPBERRY (ALL OR UNSPEC) | Redacted4 | 0.2296 | 1 | A | G | 0.1148 |
| 2017 | 4-Apr-17 | VENTURA | RASPBERRY (ALL OR UNSPEC) | Redacted4 | 0.2296 | 1 | A | G | 0.1148 |
| 2017 | 20-Jul-17 | VENTURA | RASPBERRY (ALL OR UNSPEC) | Redacted4 | 2.6245 | 11.43 | A | G | 0.1148 |
| 2017 | 14-Jun-17 | VENTURA | RASPBERRY (ALL OR UNSPEC) | Redacted4 | 1.3778 | 6 | A | G | 0.1148 |
| 2017 | 21-Jul-17 | VENTURA | RASPBERRY (ALL OR UNSPEC) | Redacted4 | 1.3778 | 6 | A | G | 0.1148 |
| 2017 | 13-Oct-17 | VENTURA | RASPBERRY (ALL OR UNSPEC) | Redacted4 | 1.3778 | 6 | A | G | 0.1148 |
| 2017 | 14-Apr-17 | VENTURA | RASPBERRY (ALL OR UNSPEC) | Redacted4 | 0.6889 | 3 | A | G | 0.1148 |
| 2017 | 15-Apr-17 | VENTURA | RASPBERRY (ALL OR UNSPEC) | Redacted4 | 1.1941 | 5.2 | A | G | 0.1148 |
| 2017 | 14-Apr-17 | VENTURA | RASPBERRY (ALL OR UNSPEC) | Redacted4 | 1.8371 | 8 | A | G | 0.1148 |
| 2017 | 14-Jun-17 | VENTURA | RASPBERRY (ALL OR UNSPEC) | Redacted4 | 0.8267 | 3.6 | A | G | 0.1148 |
| 2017 | 14-Apr-17 | VENTURA | RASPBERRY (ALL OR UNSPEC) | Redacted4 | 0.8267 | 3.6 | A | G | 0.1148 |
| 2017 | 17-May-17 | VENTURA | RASPBERRY (ALL OR UNSPEC) | Redacted4 | 0.8267 | 3.6 | A | G | 0.1148 |
| 2017 | 27-May-17 | VENTURA | RASPBERRY (ALL OR UNSPEC) | Redacted4 | 0.8267 | 3.6 | A | G | 0.1148 |
| 2017 | 22-Jun-17 | VENTURA | RASPBERRY (ALL OR UNSPEC) | Redacted4 | 3.4446 | 15 | A | G | 0.1148 |
| 2017 | 6-Aug-17 | VENTURA | RASPBERRY (ALL OR UNSPEC) | Redacted4 | 3.4446 | 15 | A | G | 0.1148 |
| 2017 | 18-Jul-17 | VENTURA | RASPBERRY (ALL OR UNSPEC) | Redacted4 | 2.2964 | 10 | A | G | 0.1148 |
| 2017 | 15-Aug-17 | VENTURA | RASPBERRY (ALL OR UNSPEC) | Redacted4 | 2.2964 | 10 | A | G | 0.1148 |
| 2017 | 18-Jul-17 | VENTURA | RASPBERRY (ALL OR UNSPEC) | Redacted4 | 1.1482 | 5 | A | G | 0.1148 |
| 2017 | 31-May-17 | VENTURA | RASPBERRY (ALL OR UNSPEC) | Redacted4 | 2.2964 | 10 | A | G | 0.1148 |
| 2017 | 29-Jun-17 | VENTURA | RASPBERRY (ALL OR UNSPEC) | Redacted4 | 2.2964 | 10 | A | G | 0.1148 |
| 2017 | 11-Jul-17 | VENTURA | RASPBERRY (ALL OR UNSPEC) | Redacted4 | 1.4697 | 6.4 | A | G | 0.1148 |
| 2017 | 18-Jul-17 | VENTURA | RASPBERRY (ALL OR UNSPEC) | Redacted4 | 3.215 | 14 | A | G | 0.1148 |
| 2017 | 12-May-17 | VENTURA | RASPBERRY (ALL OR UNSPEC) | Redacted4 | 1.286 | 5.6 | A | G | 0.1148 |
| 2017 | 7-Jun-17 | VENTURA | RASPBERRY (ALL OR UNSPEC) | Redacted4 | 2.0668 | 9 | A | G | 0.1148 |
| 2017 | 5-Jul-17 | VENTURA | RASPBERRY (ALL OR UNSPEC) | Redacted4 | 0.9186 | 4 | A | G | 0.1148 |
| 2017 | 17-Apr-17 | VENTURA | RASPBERRY (ALL OR UNSPEC) | Redacted4 | 0.9186 | 4 | A | G | 0.1148 |
| 2017 | 14-Jul-17 | VENTURA | RASPBERRY (ALL OR UNSPEC) | Redacted4 | 0.9186 | 4 | A | G | 0.1148 |
| 2017 | 27-May-17 | VENTURA | RASPBERRY (ALL OR UNSPEC) | Redacted4 | 0.9186 | 4 | A | G | 0.1148 |
| 2017 | 13-May-17 | VENTURA | RASPBERRY (ALL OR UNSPEC) | Redacted4 | 5.4064 | 23.54 | A | G | 0.1148 |
| 2017 | 25-May-17 | VENTURA | RASPBERRY (ALL OR UNSPEC) | Redacted4 | 5.4064 | 23.54 | A | G | 0.1148 |
| 2017 | 12-May-17 | VENTURA | RASPBERRY (ALL OR UNSPEC) | Redacted4 | 1.6928 | 7.37 | A | G | 0.1148 |
| 2017 | 4-May-17 | VENTURA | RASPBERRY (ALL OR UNSPEC) | Redacted4 | 3.4249 | 14.91 | A | G | 0.1149 |
| 2017 | 8-May-17 | VENTURA | RASPBERRY (ALL OR UNSPEC) | Redacted4 | 3.4249 | 14.91 | A | G | 0.1149 |
| 2017 | 9-May-17 | VENTURA | RASPBERRY (ALL OR UNSPEC) | Redacted4 | 3.4249 | 14.91 | A | G | 0.1149 |
| 2017 | 17-Jun-17 | VENTURA | RASPBERRY (ALL OR UNSPEC) | Redacted4 | 1.9618 | 8.54 | A | G | 0.1149 |
| 2017 | 15-Jul-17 | VENTURA | RASPBERRY (ALL OR UNSPEC) | Redacted4 | 1.9618 | 8.54 | A | G | 0.1149 |
| 2017 | 4-May-17 | VENTURA | RASPBERRY (ALL OR UNSPEC) | Redacted4 | 1.9618 | 8.54 | A | G | 0.1149 |
| 2017 | 22-Apr-17 | VENTURA | RASPBERRY (ALL OR UNSPEC) | Redacted4 | 0.912 | 3.97 | A | G | 0.1149 |
| 2017 | 12-Jun-17 | VENTURA | RASPBERRY (ALL OR UNSPEC) | Redacted4 | 0.912 | 3.97 | A | G | 0.1149 |
| 2017 | 30-Apr-17 | VENTURA | RASPBERRY (ALL OR UNSPEC) | Redacted4 | 0.912 | 3.97 | A | G | 0.1149 |
| 2017 | 30-May-17 | VENTURA | RASPBERRY (ALL OR UNSPEC) | Redacted4 | 0.912 | 3.97 | A | G | 0.1149 |
| 2017 | 8-Jul-17 | VENTURA | RASPBERRY (ALL OR UNSPEC) | Redacted4 | 7.8406 | 34.13 | A | G | 0.1149 |
| 2017 | 21-Apr-17 | VENTURA | RASPBERRY (ALL OR UNSPEC) | Redacted4 | 1.7322 | 7.54 | A | G | 0.1149 |
| 2017 | 25-Apr-17 | VENTURA | RASPBERRY (ALL OR UNSPEC) | Redacted4 | 1.7322 | 7.54 | A | G | 0.1149 |
| 2017 | 31-Aug-17 | VENTURA | RASPBERRY (ALL OR UNSPEC) | Redacted4 | 3.005 | 13.08 | A | G | 0.1149 |
| 2017 | 21-Jul-17 | VENTURA | RASPBERRY (ALL OR UNSPEC) | Redacted4 | 3.005 | 13.08 | A | G | 0.1149 |
| 2017 | 20-Jul-17 | VENTURA | RASPBERRY (ALL OR UNSPEC) | Redacted4 | 1.2729 | 5.54 | A | G | 0.1149 |
| 2017 | 14-Aug-17 | VENTURA | RASPBERRY (ALL OR UNSPEC) | Redacted4 | 1.2729 | 5.54 | A | G | 0.1149 |
| 2017 | 4-Apr-17 | VENTURA | RASPBERRY (ALL OR UNSPEC) | Redacted4 | 1.2729 | 5.54 | A | G | 0.1149 |
| 2017 | 30-Apr-17 | VENTURA | RASPBERRY (ALL OR UNSPEC) | Redacted4 | 1.7715 | 7.71 | A | G | 0.1149 |
| 2017 | 22-Apr-17 | VENTURA | RASPBERRY (ALL OR UNSPEC) | Redacted4 | 1.7715 | 7.71 | A | G | 0.1149 |
| 2017 | 11-May-17 | VENTURA | RASPBERRY (ALL OR UNSPEC) | Redacted4 | 1.7715 | 7.71 | A | G | 0.1149 |
| 2017 | 9-Dec-17 | VENTURA | RASPBERRY (ALL OR UNSPEC) | Redacted4 | 2.4932 | 10.85 | A | G | 0.1149 |
| 2017 | 20-Jul-17 | VENTURA | RASPBERRY (ALL OR UNSPEC) | Redacted4 | 3.3987 | 14.79 | A | G | 0.1149 |
| 2017 | 18-May-17 | VENTURA | RASPBERRY (ALL OR UNSPEC) | Redacted4 | 0.9238 | 4.02 | A | G | 0.1149 |
| 2017 | 6-May-17 | VENTURA | RASPBERRY (ALL OR UNSPEC) | Redacted4 | 2.3948 | 10.42 | A | G | 0.1149 |
| 2017 | 16-May-17 | VENTURA | RASPBERRY (ALL OR UNSPEC) | Redacted4 | 2.3948 | 10.42 | A | G | 0.1149 |
| 2017 | 7-Apr-17 | VENTURA | RASPBERRY (ALL OR UNSPEC) | Redacted4 | 1.2204 | 5.31 | A | G | 0.1149 |
| 2017 | 4-Apr-17 | VENTURA | RASPBERRY (ALL OR UNSPEC) | Redacted4 | 1.2204 | 5.31 | A | G | 0.1149 |
| 2017 | 1-Jun-17 | VENTURA | RASPBERRY (ALL OR UNSPEC) | Redacted4 | 2.841 | 12.36 | A | G | 0.1149 |
| 2017 | 18-May-17 | VENTURA | RASPBERRY (ALL OR UNSPEC) | Redacted4 | 2.7491 | 11.96 | A | G | 0.1149 |
| 2017 | 11-May-17 | VENTURA | RASPBERRY (ALL OR UNSPEC) | Redacted4 | 2.7491 | 11.96 | A | G | 0.1149 |
| 2017 | 11-Apr-17 | VENTURA | RASPBERRY (ALL OR UNSPEC) | Redacted4 | 2.7491 | 11.96 | A | G | 0.1149 |
| 2017 | 27-Jul-17 | VENTURA | RASPBERRY (ALL OR UNSPEC) | Redacted4 | 3.379 | 14.7 | A | G | 0.1149 |
| 2017 | 27-Jul-17 | VENTURA | RASPBERRY (ALL OR UNSPEC) | Redacted4 | 1.2138 | 5.28 | A | G | 0.1149 |
| 2017 | 19-Jul-17 | VENTURA | RASPBERRY (ALL OR UNSPEC) | Redacted4 | 1.2138 | 5.28 | A | G | 0.1149 |
| 2017 | 9-Dec-17 | VENTURA | RASPBERRY (ALL OR UNSPEC) | Redacted4 | 2.4276 | 10.56 | A | G | 0.1149 |
| 2017 | 14-Jun-17 | VENTURA | RASPBERRY (ALL OR UNSPEC) | Redacted4 | 1.2138 | 5.28 | A | G | 0.1149 |
| 2017 | 8-May-17 | VENTURA | RASPBERRY (ALL OR UNSPEC) | Redacted4 | 1.2138 | 5.28 | A | G | 0.1149 |
| 2017 | 22-Jun-17 | VENTURA | RASPBERRY (ALL OR UNSPEC) | Redacted4 | 1.391 | 6.05 | A | G | 0.1150 |
| 2017 | 3-May-17 | VENTURA | RASPBERRY (ALL OR UNSPEC) | Redacted4 | 1.391 | 6.05 | A | G | 0.1150 |
| 2017 | 17-Jun-17 | VENTURA | RASPBERRY (ALL OR UNSPEC) | Redacted4 | 1.391 | 6.05 | A | G | 0.1150 |
| 2017 | 18-May-17 | VENTURA | RASPBERRY (ALL OR UNSPEC) | Redacted4 | 2.6442 | 11.5 | A | G | 0.1150 |
| 2017 | 8-Jun-17 | VENTURA | RASPBERRY (ALL OR UNSPEC) | Redacted4 | 2.5064 | 10.9 | A | G | 0.1150 |
| 2017 | 24-Aug-17 | VENTURA | RASPBERRY (ALL OR UNSPEC) | Redacted4 | 1.6993 | 7.39 | A | G | 0.1150 |
| 2017 | 4-Apr-17 | VENTURA | RASPBERRY (ALL OR UNSPEC) | Redacted4 | 0.9842 | 4.28 | A | G | 0.1150 |
| 2017 | 26-Aug-17 | VENTURA | RASPBERRY (ALL OR UNSPEC) | Redacted4 | 2.2767 | 9.9 | A | G | 0.1150 |
| 2017 | 16-May-17 | VENTURA | RASPBERRY (ALL OR UNSPEC) | Redacted4 | 2.3227 | 10.1 | A | G | 0.1150 |
| 2017 | 6-May-17 | VENTURA | RASPBERRY (ALL OR UNSPEC) | Redacted4 | 2.3227 | 10.1 | A | G | 0.1150 |
| 2017 | 6-May-17 | VENTURA | RASPBERRY (ALL OR UNSPEC) | Redacted4 | 1.8306 | 7.96 | A | G | 0.1150 |
| 2017 | 14-Aug-17 | VENTURA | RASPBERRY (ALL OR UNSPEC) | Redacted4 | 1.5616 | 6.79 | A | G | 0.1150 |
| 2017 | 12-Oct-17 | VENTURA | RASPBERRY (ALL OR UNSPEC) | Redacted4 | 1.5616 | 6.79 | A | G | 0.1150 |
| 2017 | 6-Aug-17 | VENTURA | RASPBERRY (ALL OR UNSPEC) | Redacted4 | 2.1849 | 9.5 | A | G | 0.1150 |
| 2017 | 22-Apr-17 | VENTURA | RASPBERRY (ALL OR UNSPEC) | Redacted4 | 1.4238 | 6.19 | A | G | 0.1150 |
| 2017 | 5-Apr-17 | VENTURA | RASPBERRY (ALL OR UNSPEC) | Redacted4 | 1.555 | 6.76 | A | G | 0.1150 |
| 2017 | 10-Apr-17 | VENTURA | RASPBERRY (ALL OR UNSPEC) | Redacted4 | 1.7256 | 7.5 | A | G | 0.1150 |
| 2017 | 10-Apr-17 | VENTURA | RASPBERRY (ALL OR UNSPEC) | Redacted4 | 1.7256 | 7.5 | A | G | 0.1150 |
| 2017 | 20-Dec-17 | VENTURA | RASPBERRY (ALL OR UNSPEC) | Redacted4 | 1.5025 | 6.53 | A | G | 0.1150 |
| 2017 | 5-Jun-17 | VENTURA | RASPBERRY (ALL OR UNSPEC) | Redacted4 | 0.4855 | 2.11 | A | G | 0.1150 |
| 2017 | 14-Aug-17 | VENTURA | RASPBERRY (ALL OR UNSPEC) | Redacted4 | 0.4855 | 2.11 | A | G | 0.1150 |
| 2017 | 12-Oct-17 | VENTURA | RASPBERRY (ALL OR UNSPEC) | Redacted4 | 0.4855 | 2.11 | A | G | 0.1150 |
| 2017 | 2-Sep-17 | VENTURA | RASPBERRY (ALL OR UNSPEC) | Redacted4 | 1.3188 | 5.73 | A | G | 0.1151 |
| 2017 | 17-Jun-17 | VENTURA | RASPBERRY (ALL OR UNSPEC) | Redacted4 | 1.3188 | 5.73 | A | G | 0.1151 |
| 2017 | 22-Jul-17 | VENTURA | RASPBERRY (ALL OR UNSPEC) | Redacted4 | 1.3188 | 5.73 | A | G | 0.1151 |
| 2017 | 15-Jul-17 | VENTURA | RASPBERRY (ALL OR UNSPEC) | Redacted4 | 1.3188 | 5.73 | A | G | 0.1151 |
| 2017 | 1-Dec-17 | VENTURA | RASPBERRY (ALL OR UNSPEC) | Redacted4 | 1.3188 | 5.73 | A | G | 0.1151 |
| 2017 | 24-Jun-17 | VENTURA | RASPBERRY (ALL OR UNSPEC) | Redacted4 | 1.2204 | 5.3 | A | G | 0.1151 |
| 2017 | 13-May-17 | VENTURA | RASPBERRY (ALL OR UNSPEC) | Redacted4 | 1.2204 | 5.3 | A | G | 0.1151 |
| 2017 | 11-May-17 | VENTURA | RASPBERRY (ALL OR UNSPEC) | Redacted4 | 1.0432 | 4.53 | A | G | 0.1151 |
| 2017 | 27-Oct-17 | VENTURA | RASPBERRY (ALL OR UNSPEC) | Redacted4 | 2.6875 | 11.67 | A | G | 0.1151 |
| 2017 | 15-Nov-17 | VENTURA | RASPBERRY (ALL OR UNSPEC) | Redacted4 | 2.6875 | 11.67 | A | G | 0.1151 |
| 2017 | 22-Apr-17 | VENTURA | RASPBERRY (ALL OR UNSPEC) | Redacted4 | 0.7808 | 3.39 | A | G | 0.1152 |
| 2017 | 30-Apr-17 | VENTURA | RASPBERRY (ALL OR UNSPEC) | Redacted4 | 0.7808 | 3.39 | A | G | 0.1152 |
| 2017 | 24-May-17 | VENTURA | RASPBERRY (ALL OR UNSPEC) | Redacted4 | 1.0367 | 4.5 | A | G | 0.1152 |
| 2017 | 15-Dec-17 | VENTURA | RASPBERRY (ALL OR UNSPEC) | Redacted4 | 0.9907 | 4.3 | A | G | 0.1152 |
| 2017 | 3-May-17 | VENTURA | RASPBERRY (ALL OR UNSPEC) | Redacted4 | 0.7742 | 3.36 | A | G | 0.1152 |
| 2017 | 22-Jun-17 | VENTURA | RASPBERRY (ALL OR UNSPEC) | Redacted4 | 0.7742 | 3.36 | A | G | 0.1152 |
| 2017 | 17-Jun-17 | VENTURA | RASPBERRY (ALL OR UNSPEC) | Redacted4 | 0.7742 | 3.36 | A | G | 0.1152 |
| 2017 | 11-May-17 | VENTURA | RASPBERRY (ALL OR UNSPEC) | Redacted4 | 0.853 | 3.7 | A | G | 0.1153 |
| 2017 | 12-Jul-17 | VENTURA | RASPBERRY (ALL OR UNSPEC) | Redacted4 | 0.853 | 3.7 | A | G | 0.1153 |
| 2017 | 21-Jul-17 | VENTURA | RASPBERRY (ALL OR UNSPEC) | Redacted4 | 0.807 | 3.5 | A | G | 0.1153 |
| 2017 | 21-Jul-17 | VENTURA | RASPBERRY (ALL OR UNSPEC) | Redacted4 | 0.807 | 3.5 | A | G | 0.1153 |
| 2017 | 1-Aug-17 | VENTURA | RASPBERRY (ALL OR UNSPEC) | Redacted4 | 0.807 | 3.5 | A | G | 0.1153 |
| 2017 | 19-Dec-17 | VENTURA | RASPBERRY (ALL OR UNSPEC) | Redacted4 | 0.4658 | 2.02 | A | G | 0.1153 |
| 2017 | 14-Jun-17 | VENTURA | RASPBERRY (ALL OR UNSPEC) | Redacted4 | 0.5774 | 2.5 | A | G | 0.1155 |
| 2017 | 31-Jul-17 | VENTURA | RASPBERRY (ALL OR UNSPEC) | Redacted4 | 0.5774 | 2.5 | A | G | 0.1155 |
| 2017 | 8-May-17 | VENTURA | RASPBERRY (ALL OR UNSPEC) | Redacted4 | 0.5774 | 2.5 | A | G | 0.1155 |
| 2017 | 4-Apr-17 | VENTURA | RASPBERRY (ALL OR UNSPEC) | Redacted4 | 0.5774 | 2.5 | A | G | 0.1155 |
| 2017 | 22-Jul-17 | VENTURA | RASPBERRY (ALL OR UNSPEC) | Redacted4 | 2.5195 | 10.89 | A | G | 0.1157 |
| 2017 | 18-Nov-17 | VENTURA | RASPBERRY (ALL OR UNSPEC) | Redacted4 | 0.9238 | 3.98 | A | G | 0.1161 |
| 2017 | 26-Jul-17 | VENTURA | RASPBERRY (ALL OR UNSPEC) | Redacted4 | 0.9238 | 3.97 | A | G | 0.1163 |
| 2017 | 30-May-17 | VENTURA | RASPBERRY (ALL OR UNSPEC) | Redacted4 | 0.9238 | 3.97 | A | G | 0.1163 |
| 2017 | 18-May-17 | VENTURA | RASPBERRY (ALL OR UNSPEC) | Redacted4 | 1.0078 | 4.32 | A | G | 0.1166 |
| 2017 | 20-Jul-17 | VENTURA | RASPBERRY (ALL OR UNSPEC) | Redacted4 | 1.0078 | 4.32 | A | G | 0.1166 |
| 2017 | 11-Nov-17 | VENTURA | RASPBERRY (ALL OR UNSPEC) | Redacted4 | 1.0078 | 4.32 | A | G | 0.1166 |
| 2017 | 30-Jun-17 | VENTURA | RASPBERRY (ALL OR UNSPEC) | Redacted4 | 3.5627 | 15.25 | A | G | 0.1168 |
| 2017 | 18-May-17 | VENTURA | RASPBERRY (ALL OR UNSPEC) | Redacted4 | 0.0262 | 0.1 | A | G | 0.1310 |
| 2017 | 11-May-17 | VENTURA | RASPBERRY (ALL OR UNSPEC) | Redacted4 | 0.0262 | 0.1 | A | G | 0.1310 |
| 2017 | 24-Mar-17 | VENTURA | RASPBERRY (ALL OR UNSPEC) | Redacted4 | 7.3354 | 27.95 | A | G | 0.1312 |
| 2017 | 30-Jun-17 | MONTEREY | RASPBERRY (ALL OR UNSPEC) | Redacted4 | 9.3221 | 15 | A | G | 0.3107 |
| 2017 | 21-Jul-17 | MONTEREY | RASPBERRY (ALL OR UNSPEC) | Redacted4 | 9.3221 | 15 | A | G | 0.3107 |
| 2017 | 25-Jul-17 | MONTEREY | RASPBERRY (ALL OR UNSPEC) | Redacted4 | 12.4295 | 20 | A | G | 0.3107 |
| 2017 | 18-Jul-17 | MONTEREY | RASPBERRY (ALL OR UNSPEC) | Redacted4 | 18.0564 | 29 | A | G | 0.3113 |
| 2017 | 1-Aug-17 | MONTEREY | RASPBERRY (ALL OR UNSPEC) | Redacted4 | 25.5309 | 41 | A | G | 0.3114 |
| 2017 | 18-Sep-17 | MONTEREY | RASPBERRY (ALL OR UNSPEC) | Redacted4 | 24.943 | 40 | A | G | 0.3118 |
| 2017 | 4-Aug-17 | MONTEREY | RASPBERRY (ALL OR UNSPEC) | Redacted4 | 18.7282 | 30 | A | G | 0.3121 |
| 2017 | 15-Jun-17 | MONTEREY | RASPBERRY (ALL OR UNSPEC) | Redacted4 | 9.0702 | 14.5 | A | G | 0.3128 |
| 2017 | 11-Jul-17 | SANTA CLARA | SQUASH (ALL OR UNSPEC) | Redacted4 | 1.8476 | 7 | A | G | 0.1320 |
| 2017 | 29-Jul-17 | SANTA CLARA | SQUASH (ALL OR UNSPEC) | Redacted4 | 2.2675 | 7 | A | G | 0.1620 |
| 2017 | 25-Oct-17 | SANTA CLARA | SQUASH (SUMMER) | Redacted4 | 1.0918 | 4.25 | A | G | 0.1284 |
| 2017 | 16-Sep-17 | SANTA CLARA | SQUASH (SUMMER) | Redacted4 | 1.0918 | 4.25 | A | G | 0.1284 |
| 2017 | 9-May-17 | MONTEREY | STRAWBERRY (ALL OR UNSPEC) | Redacted4 | 0.6719 | 8 | A | G | 0.0420 |
| 2017 | 18-Dec-17 | VENTURA | STRAWBERRY (ALL OR UNSPEC) | Redacted4 | 0.2519 | 2 | A | G | 0.0630 |
| 2017 | 22-Aug-17 | VENTURA | STRAWBERRY (ALL OR UNSPEC) | Redacted4 | 0.3281 | 2.5 | A | G | 0.0656 |
| 2017 | 17-Jun-17 | SANTA CRUZ | STRAWBERRY (ALL OR UNSPEC) | Redacted4 | 3.4446 | 21 | A | G | 0.0820 |
| 2017 | 17-Jun-17 | SANTA CRUZ | STRAWBERRY (ALL OR UNSPEC) | Redacted4 | 3.9367 | 24 | A | G | 0.0820 |
| 2017 | 17-Jun-17 | SANTA CRUZ | STRAWBERRY (ALL OR UNSPEC) | Redacted4 | 4.9209 | 30 | A | G | 0.0820 |
| 2017 | 17-Jun-17 | SANTA CRUZ | STRAWBERRY (ALL OR UNSPEC) | Redacted4 | 1.9684 | 12 | A | G | 0.0820 |
| 2017 | 4-May-17 | SANTA CRUZ | STRAWBERRY (ALL OR UNSPEC) | Redacted4 | 0.3359 | 2 | A | G | 0.0840 |
| 2017 | 6-May-17 | SANTA CRUZ | STRAWBERRY (ALL OR UNSPEC) | Redacted4 | 1.0078 | 6 | A | G | 0.0840 |
| 2017 | 27-Apr-17 | SANTA CRUZ | STRAWBERRY (ALL OR UNSPEC) | Redacted4 | 0.5039 | 3 | A | G | 0.0840 |
| 2017 | 5-May-17 | SANTA CRUZ | STRAWBERRY (ALL OR UNSPEC) | Redacted4 | 0.5039 | 3 | A | G | 0.0840 |
| 2017 | 30-Aug-17 | VENTURA | STRAWBERRY (ALL OR UNSPEC) | Redacted4 | 0.168 | 1 | A | G | 0.0840 |
| 2017 | 18-Dec-17 | VENTURA | STRAWBERRY (ALL OR UNSPEC) | Redacted4 | 0.7558 | 4 | A | G | 0.0945 |
| 2017 | 28-Aug-17 | VENTURA | STRAWBERRY (ALL OR UNSPEC) | Redacted4 | 1.7715 | 9 | A | G | 0.0984 |
| 2017 | 20-May-17 | SANTA CRUZ | STRAWBERRY (ALL OR UNSPEC) | Redacted4 | 2.5589 | 13 | A | G | 0.0984 |
| 2017 | 20-May-17 | SANTA CRUZ | STRAWBERRY (ALL OR UNSPEC) | Redacted4 | 2.5589 | 13 | A | G | 0.0984 |
| 2017 | 20-May-17 | SANTA CRUZ | STRAWBERRY (ALL OR UNSPEC) | Redacted4 | 1.8306 | 9.3 | A | G | 0.0984 |
| 2017 | 29-Sep-17 | VENTURA | STRAWBERRY (ALL OR UNSPEC) | Redacted4 | 3.7792 | 19 | A | G | 0.0995 |
| 2017 | 1-May-17 | MONTEREY | STRAWBERRY (ALL OR UNSPEC) | Redacted4 | 1.6797 | 8 | A | G | 0.1050 |
| 2017 | 1-May-17 | MONTEREY | STRAWBERRY (ALL OR UNSPEC) | Redacted4 | 9.1542 | 40 | A | G | 0.1144 |
| 2017 | 12-May-17 | SANTA CRUZ | STRAWBERRY (ALL OR UNSPEC) | Redacted4 | 7.5585 | 33 | A | G | 0.1145 |
| 2017 | 24-Apr-17 | SANTA CRUZ | STRAWBERRY (ALL OR UNSPEC) | Redacted4 | 7.5585 | 33 | A | G | 0.1145 |
| 2017 | 20-Apr-17 | SANTA CRUZ | STRAWBERRY (ALL OR UNSPEC) | Redacted4 | 22.9274 | 100 | A | G | 0.1146 |
| 2017 | 21-Apr-17 | MONTEREY | STRAWBERRY (ALL OR UNSPEC) | Redacted4 | 3.4433 | 15 | A | G | 0.1148 |
| 2017 | 1-May-17 | MONTEREY | STRAWBERRY (ALL OR UNSPEC) | Redacted4 | 3.4433 | 15 | A | G | 0.1148 |
| 2017 | 22-Apr-17 | SANTA CRUZ | STRAWBERRY (ALL OR UNSPEC) | Redacted4 | 6.8866 | 30 | A | G | 0.1148 |
| 2017 | 26-Aug-17 | VENTURA | STRAWBERRY (ALL OR UNSPEC) | Redacted4 | 9.1856 | 40 | A | G | 0.1148 |
| 2017 | 28-Aug-17 | VENTURA | STRAWBERRY (ALL OR UNSPEC) | Redacted4 | 10.8784 | 47.36 | A | G | 0.1148 |
| 2017 | 23-Aug-17 | VENTURA | STRAWBERRY (ALL OR UNSPEC) | Redacted4 | 9.7762 | 42.56 | A | G | 0.1149 |
| 2017 | 1-May-17 | SANTA CRUZ | STRAWBERRY (ALL OR UNSPEC) | Redacted4 | 8.7342 | 38 | A | G | 0.1149 |
| 2017 | 10-May-17 | SANTA CRUZ | STRAWBERRY (ALL OR UNSPEC) | Redacted4 | 8.0624 | 35 | A | G | 0.1152 |
| 2017 | 22-Apr-17 | MONTEREY | STRAWBERRY (ALL OR UNSPEC) | Redacted4 | 1.8476 | 8 | A | G | 0.1155 |
| 2017 | 11-May-17 | SANTA CRUZ | STRAWBERRY (ALL OR UNSPEC) | Redacted4 | 2.7714 | 12 | A | G | 0.1155 |
| 2017 | 8-May-17 | SANTA CRUZ | STRAWBERRY (ALL OR UNSPEC) | Redacted4 | 7.3905 | 32 | A | G | 0.1155 |
| 2017 | 8-Apr-17 | MONTEREY | STRAWBERRY (ALL OR UNSPEC) | Redacted4 | 6.4667 | 28 | A | G | 0.1155 |
| 2017 | 15-Apr-17 | MONTEREY | STRAWBERRY (ALL OR UNSPEC) | Redacted4 | 6.4667 | 28 | A | G | 0.1155 |
| 2017 | 25-Apr-17 | MONTEREY | STRAWBERRY (ALL OR UNSPEC) | Redacted4 | 6.4667 | 28 | A | G | 0.1155 |
| 2017 | 20-Apr-17 | MONTEREY | STRAWBERRY (ALL OR UNSPEC) | Redacted4 | 4.6191 | 20 | A | G | 0.1155 |
| 2017 | 14-Apr-17 | MONTEREY | STRAWBERRY (ALL OR UNSPEC) | Redacted4 | 4.6191 | 20 | A | G | 0.1155 |
| 2017 | 20-Apr-17 | MONTEREY | STRAWBERRY (ALL OR UNSPEC) | Redacted4 | 4.6191 | 20 | A | G | 0.1155 |
| 2017 | 8-Apr-17 | MONTEREY | STRAWBERRY (ALL OR UNSPEC) | Redacted4 | 3.0234 | 13 | A | G | 0.1163 |
| 2017 | 26-Apr-17 | MONTEREY | STRAWBERRY (ALL OR UNSPEC) | Redacted4 | 3.0234 | 13 | A | G | 0.1163 |
| 2017 | 10-May-17 | MONTEREY | STRAWBERRY (ALL OR UNSPEC) | Redacted4 | 3.0234 | 13 | A | G | 0.1163 |
| 2017 | 15-Apr-17 | MONTEREY | STRAWBERRY (ALL OR UNSPEC) | Redacted4 | 3.0234 | 13 | A | G | 0.1163 |
| 2017 | 20-Apr-17 | SANTA CRUZ | STRAWBERRY (ALL OR UNSPEC) | Redacted4 | 1.1758 | 5 | A | G | 0.1176 |
| 2017 | 8-Dec-17 | VENTURA | STRAWBERRY (ALL OR UNSPEC) | Redacted4 | 0.5039 | 2 | A | G | 0.1260 |
| 2017 | 6-Oct-17 | VENTURA | STRAWBERRY (ALL OR UNSPEC) | Redacted4 | 0.5039 | 2 | A | G | 0.1260 |
| 2017 | 18-Nov-17 | VENTURA | STRAWBERRY (ALL OR UNSPEC) | Redacted4 | 0.5039 | 2 | A | G | 0.1260 |
| 2017 | 8-Dec-17 | VENTURA | STRAWBERRY (ALL OR UNSPEC) | Redacted4 | 0.5039 | 2 | A | G | 0.1260 |
| 2017 | 20-Apr-17 | VENTURA | STRAWBERRY (ALL OR UNSPEC) | Redacted4 | 1.9316 | 7.5 | A | G | 0.1288 |
| 2017 | 22-Dec-17 | VENTURA | STRAWBERRY (ALL OR UNSPEC) | Redacted4 | 3.1074 | 12 | A | G | 0.1295 |
| 2017 | 25-May-17 | VENTURA | STRAWBERRY (ALL OR UNSPEC) | Redacted4 | 5.2069 | 20 | A | G | 0.1302 |
| 2017 | 17-Nov-17 | VENTURA | STRAWBERRY (ALL OR UNSPEC) | Redacted4 | 7.8104 | 30 | A | G | 0.1302 |
| 2017 | 23-Dec-17 | VENTURA | STRAWBERRY (ALL OR UNSPEC) | Redacted4 | 4.955 | 19 | A | G | 0.1304 |
| 2017 | 30-Oct-17 | VENTURA | STRAWBERRY (ALL OR UNSPEC) | Redacted4 | 11.7576 | 45 | A | G | 0.1306 |
| 2017 | 10-Oct-17 | VENTURA | STRAWBERRY (ALL OR UNSPEC) | Redacted4 | 11.7576 | 45 | A | G | 0.1306 |
| 2017 | 28-Oct-17 | VENTURA | STRAWBERRY (ALL OR UNSPEC) | Redacted4 | 11.7576 | 45 | A | G | 0.1306 |
| 2017 | 23-Dec-17 | VENTURA | STRAWBERRY (ALL OR UNSPEC) | Redacted4 | 9.4061 | 36 | A | G | 0.1306 |
| 2017 | 16-Nov-17 | VENTURA | STRAWBERRY (ALL OR UNSPEC) | Redacted4 | 11.5057 | 44 | A | G | 0.1307 |
| 2017 | 7-Oct-17 | VENTURA | STRAWBERRY (ALL OR UNSPEC) | Redacted4 | 13.6053 | 52 | A | G | 0.1308 |
| 2017 | 28-Oct-17 | VENTURA | STRAWBERRY (ALL OR UNSPEC) | Redacted4 | 13.6053 | 52 | A | G | 0.1308 |
| 2017 | 27-May-17 | VENTURA | STRAWBERRY (ALL OR UNSPEC) | Redacted4 | 11.2537 | 43 | A | G | 0.1309 |
| 2017 | 16-Nov-17 | VENTURA | STRAWBERRY (ALL OR UNSPEC) | Redacted4 | 8.9022 | 34 | A | G | 0.1309 |
| 2017 | 19-Sep-17 | VENTURA | STRAWBERRY (ALL OR UNSPEC) | Redacted4 | 13.3533 | 51 | A | G | 0.1309 |
| 2017 | 24-Nov-17 | VENTURA | STRAWBERRY (ALL OR UNSPEC) | Redacted4 | 8.9022 | 34 | A | G | 0.1309 |
| 2017 | 23-Dec-17 | VENTURA | STRAWBERRY (ALL OR UNSPEC) | Redacted4 | 4.4511 | 17 | A | G | 0.1309 |
| 2017 | 13-Nov-17 | VENTURA | STRAWBERRY (ALL OR UNSPEC) | Redacted4 | 10.2459 | 39.13 | A | G | 0.1309 |
| 2017 | 24-Jun-17 | VENTURA | STRAWBERRY (ALL OR UNSPEC) | Redacted4 | 11.0018 | 42 | A | G | 0.1310 |
| 2017 | 21-Oct-17 | VENTURA | STRAWBERRY (ALL OR UNSPEC) | Redacted4 | 11.0018 | 42 | A | G | 0.1310 |
| 2017 | 10-Oct-17 | VENTURA | STRAWBERRY (ALL OR UNSPEC) | Redacted4 | 11.0018 | 42 | A | G | 0.1310 |
| 2017 | 18-Nov-17 | VENTURA | STRAWBERRY (ALL OR UNSPEC) | Redacted4 | 11.0018 | 42 | A | G | 0.1310 |
| 2017 | 7-Oct-17 | VENTURA | STRAWBERRY (ALL OR UNSPEC) | Redacted4 | 11.0018 | 42 | A | G | 0.1310 |
| 2017 | 9-Sep-17 | VENTURA | STRAWBERRY (ALL OR UNSPEC) | Redacted4 | 11.0018 | 42 | A | G | 0.1310 |
| 2017 | 6-Oct-17 | VENTURA | STRAWBERRY (ALL OR UNSPEC) | Redacted4 | 26.2027 | 100 | A | G | 0.1310 |
| 2017 | 23-May-17 | VENTURA | STRAWBERRY (ALL OR UNSPEC) | Redacted4 | 13.1014 | 50 | A | G | 0.1310 |
| 2017 | 4-Mar-17 | VENTURA | STRAWBERRY (ALL OR UNSPEC) | Redacted4 | 21.7516 | 83 | A | G | 0.1310 |
| 2017 | 16-Sep-17 | VENTURA | STRAWBERRY (ALL OR UNSPEC) | Redacted4 | 8.6503 | 33 | A | G | 0.1311 |
| 2017 | 9-Sep-17 | VENTURA | STRAWBERRY (ALL OR UNSPEC) | Redacted4 | 8.6503 | 33 | A | G | 0.1311 |
| 2017 | 30-Sep-17 | VENTURA | STRAWBERRY (ALL OR UNSPEC) | Redacted4 | 8.6503 | 33 | A | G | 0.1311 |
| 2017 | 15-Dec-17 | VENTURA | STRAWBERRY (ALL OR UNSPEC) | Redacted4 | 19.4001 | 74 | A | G | 0.1311 |
| 2017 | 28-May-17 | VENTURA | STRAWBERRY (ALL OR UNSPEC) | Redacted4 | 23.5992 | 90 | A | G | 0.1311 |
| 2017 | 15-Dec-17 | VENTURA | STRAWBERRY (ALL OR UNSPEC) | Redacted4 | 23.5992 | 90 | A | G | 0.1311 |
| 2017 | 18-Sep-17 | VENTURA | STRAWBERRY (ALL OR UNSPEC) | Redacted4 | 12.8494 | 49 | A | G | 0.1311 |
| 2017 | 27-May-17 | VENTURA | STRAWBERRY (ALL OR UNSPEC) | Redacted4 | 14.949 | 57 | A | G | 0.1311 |
| 2017 | 2-Dec-17 | VENTURA | STRAWBERRY (ALL OR UNSPEC) | Redacted4 | 3.7399 | 14.26 | A | G | 0.1311 |
| 2017 | 7-Sep-17 | SANTA CRUZ | STRAWBERRY (ALL OR UNSPEC) | Redacted4 | 17.0486 | 65 | A | G | 0.1311 |
| 2017 | 20-Dec-17 | VENTURA | STRAWBERRY (ALL OR UNSPEC) | Redacted4 | 8.1687 | 31.13 | A | G | 0.1312 |
| 2017 | 20-Dec-17 | VENTURA | STRAWBERRY (ALL OR UNSPEC) | Redacted4 | 19.6638 | 74.93 | A | G | 0.1312 |
| 2017 | 2-Dec-17 | VENTURA | STRAWBERRY (ALL OR UNSPEC) | Redacted4 | 19.6638 | 74.93 | A | G | 0.1312 |
| 2017 | 21-Jun-17 | SANTA CRUZ | STRAWBERRY (ALL OR UNSPEC) | Redacted4 | 3.4118 | 13 | A | G | 0.1312 |
| 2017 | 21-Jun-17 | SANTA CRUZ | STRAWBERRY (ALL OR UNSPEC) | Redacted4 | 3.4118 | 13 | A | G | 0.1312 |
| 2017 | 24-May-17 | VENTURA | STRAWBERRY (ALL OR UNSPEC) | Redacted4 | 31.4936 | 120 | A | G | 0.1312 |
| 2017 | 12-Dec-17 | VENTURA | STRAWBERRY (ALL OR UNSPEC) | Redacted4 | 16.7966 | 64 | A | G | 0.1312 |
| 2017 | 15-Dec-17 | VENTURA | STRAWBERRY (ALL OR UNSPEC) | Redacted4 | 33.5932 | 128 | A | G | 0.1312 |
| 2017 | 29-Jun-17 | VENTURA | STRAWBERRY (ALL OR UNSPEC) | Redacted4 | 8.3983 | 32 | A | G | 0.1312 |
| 2017 | 29-Jul-17 | SANTA CRUZ | STRAWBERRY (ALL OR UNSPEC) | Redacted4 | 22.8329 | 87 | A | G | 0.1312 |
| 2017 | 16-Dec-17 | VENTURA | STRAWBERRY (ALL OR UNSPEC) | Redacted4 | 29.3941 | 112 | A | G | 0.1312 |
| 2017 | 9-Sep-17 | MONTEREY | STRAWBERRY (ALL OR UNSPEC) | Redacted4 | 10.4979 | 40 | A | G | 0.1312 |
| 2017 | 5-Nov-17 | VENTURA | STRAWBERRY (ALL OR UNSPEC) | Redacted4 | 10.4979 | 40 | A | G | 0.1312 |
| 2017 | 2-Oct-17 | VENTURA | STRAWBERRY (ALL OR UNSPEC) | Redacted4 | 10.4979 | 40 | A | G | 0.1312 |
| 2017 | 17-Nov-17 | VENTURA | STRAWBERRY (ALL OR UNSPEC) | Redacted4 | 10.4979 | 40 | A | G | 0.1312 |
| 2017 | 24-Sep-17 | VENTURA | STRAWBERRY (ALL OR UNSPEC) | Redacted4 | 10.4979 | 40 | A | G | 0.1312 |
| 2017 | 19-Nov-17 | VENTURA | STRAWBERRY (ALL OR UNSPEC) | Redacted4 | 10.4979 | 40 | A | G | 0.1312 |
| 2017 | 28-Nov-17 | VENTURA | STRAWBERRY (ALL OR UNSPEC) | Redacted4 | 10.4979 | 40 | A | G | 0.1312 |
| 2017 | 8-Sep-17 | VENTURA | STRAWBERRY (ALL OR UNSPEC) | Redacted4 | 10.4979 | 40 | A | G | 0.1312 |
| 2017 | 27-Nov-17 | VENTURA | STRAWBERRY (ALL OR UNSPEC) | Redacted4 | 10.4979 | 40 | A | G | 0.1312 |
| 2017 | 23-Nov-17 | VENTURA | STRAWBERRY (ALL OR UNSPEC) | Redacted4 | 10.4979 | 40 | A | G | 0.1312 |
| 2017 | 8-Oct-17 | VENTURA | STRAWBERRY (ALL OR UNSPEC) | Redacted4 | 10.4979 | 40 | A | G | 0.1312 |
| 2017 | 13-Nov-17 | VENTURA | STRAWBERRY (ALL OR UNSPEC) | Redacted4 | 10.4979 | 40 | A | G | 0.1312 |
| 2017 | 18-Sep-17 | VENTURA | STRAWBERRY (ALL OR UNSPEC) | Redacted4 | 10.4979 | 40 | A | G | 0.1312 |
| 2017 | 19-Oct-17 | VENTURA | STRAWBERRY (ALL OR UNSPEC) | Redacted4 | 10.4979 | 40 | A | G | 0.1312 |
| 2017 | 30-Oct-17 | VENTURA | STRAWBERRY (ALL OR UNSPEC) | Redacted4 | 10.4979 | 40 | A | G | 0.1312 |
| 2017 | 11-Sep-17 | VENTURA | STRAWBERRY (ALL OR UNSPEC) | Redacted4 | 10.4979 | 40 | A | G | 0.1312 |
| 2017 | 8-Sep-17 | MONTEREY | STRAWBERRY (ALL OR UNSPEC) | Redacted4 | 2.0996 | 8 | A | G | 0.1312 |
| 2017 | 13-Dec-17 | VENTURA | STRAWBERRY (ALL OR UNSPEC) | Redacted4 | 2.0996 | 8 | A | G | 0.1312 |
| 2017 | 21-Jun-17 | SANTA CRUZ | STRAWBERRY (ALL OR UNSPEC) | Redacted4 | 2.4408 | 9.3 | A | G | 0.1312 |
| 2017 | 28-May-17 | VENTURA | STRAWBERRY (ALL OR UNSPEC) | Redacted4 | 22.8434 | 87 | A | G | 0.1313 |
| 2017 | 22-Dec-17 | VENTURA | STRAWBERRY (ALL OR UNSPEC) | Redacted4 | 22.8434 | 87 | A | G | 0.1313 |
| 2017 | 1-Dec-17 | VENTURA | STRAWBERRY (ALL OR UNSPEC) | Redacted4 | 20.7438 | 79 | A | G | 0.1313 |
| 2017 | 2-Dec-17 | VENTURA | STRAWBERRY (ALL OR UNSPEC) | Redacted4 | 36.7846 | 140 | A | G | 0.1314 |
| 2017 | 4-Mar-17 | VENTURA | STRAWBERRY (ALL OR UNSPEC) | Redacted4 | 18.3923 | 70 | A | G | 0.1314 |
| 2017 | 10-Sep-17 | VENTURA | STRAWBERRY (ALL OR UNSPEC) | Redacted4 | 26.2867 | 100 | A | G | 0.1314 |
| 2017 | 18-Nov-17 | VENTURA | STRAWBERRY (ALL OR UNSPEC) | Redacted4 | 26.2867 | 100 | A | G | 0.1314 |
| 2017 | 29-Nov-17 | VENTURA | STRAWBERRY (ALL OR UNSPEC) | Redacted4 | 26.2867 | 100 | A | G | 0.1314 |
| 2017 | 30-Nov-17 | VENTURA | STRAWBERRY (ALL OR UNSPEC) | Redacted4 | 16.0408 | 61 | A | G | 0.1315 |
| 2017 | 19-Dec-17 | VENTURA | STRAWBERRY (ALL OR UNSPEC) | Redacted4 | 16.0408 | 61 | A | G | 0.1315 |
| 2017 | 16-Nov-17 | VENTURA | STRAWBERRY (ALL OR UNSPEC) | Redacted4 | 16.0408 | 61 | A | G | 0.1315 |
| 2017 | 14-Apr-17 | VENTURA | STRAWBERRY (ALL OR UNSPEC) | Redacted4 | 11.8416 | 45 | A | G | 0.1316 |
| 2017 | 24-Nov-17 | VENTURA | STRAWBERRY (ALL OR UNSPEC) | Redacted4 | 11.8416 | 45 | A | G | 0.1316 |
| 2017 | 2-Oct-17 | VENTURA | STRAWBERRY (ALL OR UNSPEC) | Redacted4 | 7.8944 | 30 | A | G | 0.1316 |
| 2017 | 13-Dec-17 | VENTURA | STRAWBERRY (ALL OR UNSPEC) | Redacted4 | 5.7948 | 22 | A | G | 0.1317 |
| 2017 | 9-Dec-17 | VENTURA | STRAWBERRY (ALL OR UNSPEC) | Redacted4 | 11.5897 | 44 | A | G | 0.1317 |
| 2017 | 28-Nov-17 | VENTURA | STRAWBERRY (ALL OR UNSPEC) | Redacted4 | 9.4901 | 36 | A | G | 0.1318 |
| 2017 | 23-Sep-17 | SANTA CRUZ | STRAWBERRY (ALL OR UNSPEC) | Redacted4 | 1.8476 | 7 | A | G | 0.1320 |
| 2017 | 11-Dec-17 | VENTURA | STRAWBERRY (ALL OR UNSPEC) | Redacted4 | 5.2909 | 20 | A | G | 0.1323 |
| 2017 | 15-Nov-17 | VENTURA | STRAWBERRY (ALL OR UNSPEC) | Redacted4 | 5.2909 | 20 | A | G | 0.1323 |
| 2017 | 11-Dec-17 | VENTURA | STRAWBERRY (ALL OR UNSPEC) | Redacted4 | 5.2909 | 20 | A | G | 0.1323 |
| 2017 | 12-Dec-17 | VENTURA | STRAWBERRY (ALL OR UNSPEC) | Redacted4 | 3.4433 | 13 | A | G | 0.1324 |
| 2017 | 15-Dec-17 | VENTURA | STRAWBERRY (ALL OR UNSPEC) | Redacted4 | 3.1914 | 12 | A | G | 0.1330 |
| 2017 | 14-Apr-17 | VENTURA | STRAWBERRY (ALL OR UNSPEC) | Redacted4 | 1.3437 | 5 | A | G | 0.1344 |
| 2017 | 30-Dec-17 | VENTURA | STRAWBERRY (ALL OR UNSPEC) | Redacted4 | 1.0918 | 4 | A | G | 0.1365 |
| 2017 | 29-Dec-17 | VENTURA | STRAWBERRY (ALL OR UNSPEC) | Redacted4 | 1.0918 | 4 | A | G | 0.1365 |
| 2017 | 9-Dec-17 | VENTURA | STRAWBERRY (ALL OR UNSPEC) | Redacted4 | 1.0918 | 4 | A | G | 0.1365 |
| 2017 | 28-Oct-17 | VENTURA | STRAWBERRY (ALL OR UNSPEC) | Redacted4 | 1.0918 | 4 | A | G | 0.1365 |
| 2017 | 12-Dec-17 | VENTURA | STRAWBERRY (ALL OR UNSPEC) | Redacted4 | 1.3437 | 4.2 | A | G | 0.1600 |
| 2017 | 23-Dec-17 | VENTURA | STRAWBERRY (ALL OR UNSPEC) | Redacted4 | 4.0312 | 12.5 | A | G | 0.1612 |
| 2017 | 11-Dec-17 | VENTURA | STRAWBERRY (ALL OR UNSPEC) | Redacted4 | 4.787 | 14.7 | A | G | 0.1628 |
| 2017 | 11-Nov-17 | VENTURA | STRAWBERRY (ALL OR UNSPEC) | Redacted4 | 11.0858 | 34 | A | G | 0.1630 |
| 2017 | 3-Jun-17 | MONTEREY | STRAWBERRY (ALL OR UNSPEC) | Redacted4 | 6.0468 | 18.5 | A | G | 0.1634 |
| 2017 | 10-May-17 | MONTEREY | STRAWBERRY (ALL OR UNSPEC) | Redacted4 | 9.1542 | 28 | A | G | 0.1635 |
| 2017 | 15-Dec-17 | VENTURA | STRAWBERRY (ALL OR UNSPEC) | Redacted4 | 9.1542 | 28 | A | G | 0.1635 |
| 2017 | 16-Dec-17 | VENTURA | STRAWBERRY (ALL OR UNSPEC) | Redacted4 | 19.652 | 60 | A | G | 0.1638 |
| 2017 | 22-Aug-17 | VENTURA | STRAWBERRY (ALL OR UNSPEC) | Redacted4 | 9.826 | 30 | A | G | 0.1638 |
| 2017 | 20-Dec-17 | VENTURA | STRAWBERRY (ALL OR UNSPEC) | Redacted4 | 16.7126 | 51 | A | G | 0.1638 |
| 2017 | 22-Dec-17 | VENTURA | STRAWBERRY (ALL OR UNSPEC) | Redacted4 | 37.7084 | 115 | A | G | 0.1639 |
| 2017 | 2-Oct-17 | VENTURA | STRAWBERRY (ALL OR UNSPEC) | Redacted4 | 13.7732 | 42 | A | G | 0.1640 |
| 2017 | 25-Nov-17 | VENTURA | STRAWBERRY (ALL OR UNSPEC) | Redacted4 | 13.7732 | 42 | A | G | 0.1640 |
| 2017 | 21-Oct-17 | VENTURA | STRAWBERRY (ALL OR UNSPEC) | Redacted4 | 13.7732 | 42 | A | G | 0.1640 |
| 2017 | 28-Oct-17 | VENTURA | STRAWBERRY (ALL OR UNSPEC) | Redacted4 | 13.7732 | 42 | A | G | 0.1640 |
| 2017 | 10-Nov-17 | VENTURA | STRAWBERRY (ALL OR UNSPEC) | Redacted4 | 13.7732 | 42 | A | G | 0.1640 |
| 2017 | 1-Nov-17 | VENTURA | STRAWBERRY (ALL OR UNSPEC) | Redacted4 | 13.7732 | 42 | A | G | 0.1640 |
| 2017 | 14-Oct-17 | VENTURA | STRAWBERRY (ALL OR UNSPEC) | Redacted4 | 13.7732 | 42 | A | G | 0.1640 |
| 2017 | 22-Nov-17 | VENTURA | STRAWBERRY (ALL OR UNSPEC) | Redacted4 | 59.376 | 181 | A | G | 0.1640 |
| 2017 | 6-Jun-17 | VENTURA | STRAWBERRY (ALL OR UNSPEC) | Redacted4 | 49.2141 | 150 | A | G | 0.1640 |
| 2017 | 10-Jun-17 | VENTURA | STRAWBERRY (ALL OR UNSPEC) | Redacted4 | 49.2141 | 150 | A | G | 0.1640 |
| 2017 | 27-May-17 | VENTURA | STRAWBERRY (ALL OR UNSPEC) | Redacted4 | 49.2141 | 150 | A | G | 0.1640 |
| 2017 | 23-Sep-17 | VENTURA | STRAWBERRY (ALL OR UNSPEC) | Redacted4 | 45.9387 | 140 | A | G | 0.1641 |
| 2017 | 19-May-17 | SANTA CRUZ | STRAWBERRY (ALL OR UNSPEC) | Redacted4 | 38.0443 | 115.93 | A | G | 0.1641 |
| 2017 | 18-Nov-17 | VENTURA | STRAWBERRY (ALL OR UNSPEC) | Redacted4 | 7.2225 | 22 | A | G | 0.1641 |
| 2017 | 25-Sep-17 | VENTURA | STRAWBERRY (ALL OR UNSPEC) | Redacted4 | 10.8338 | 33 | A | G | 0.1641 |
| 2017 | 1-Nov-17 | VENTURA | STRAWBERRY (ALL OR UNSPEC) | Redacted4 | 32.8374 | 100 | A | G | 0.1642 |
| 2017 | 21-Oct-17 | VENTURA | STRAWBERRY (ALL OR UNSPEC) | Redacted4 | 32.8374 | 100 | A | G | 0.1642 |
| 2017 | 14-Oct-17 | VENTURA | STRAWBERRY (ALL OR UNSPEC) | Redacted4 | 11.1697 | 34 | A | G | 0.1643 |
| 2017 | 21-Oct-17 | VENTURA | STRAWBERRY (ALL OR UNSPEC) | Redacted4 | 11.1697 | 34 | A | G | 0.1643 |
| 2017 | 4-Nov-17 | VENTURA | STRAWBERRY (ALL OR UNSPEC) | Redacted4 | 11.1697 | 34 | A | G | 0.1643 |
| 2017 | 1-Nov-17 | VENTURA | STRAWBERRY (ALL OR UNSPEC) | Redacted4 | 11.1697 | 34 | A | G | 0.1643 |
| 2017 | 26-Jun-17 | SANTA CRUZ | STRAWBERRY (ALL OR UNSPEC) | Redacted4 | 19.3161 | 58.77 | A | G | 0.1643 |
| 2017 | 19-May-17 | SANTA CRUZ | STRAWBERRY (ALL OR UNSPEC) | Redacted4 | 19.3161 | 58.77 | A | G | 0.1643 |
| 2017 | 27-May-17 | VENTURA | STRAWBERRY (ALL OR UNSPEC) | Redacted4 | 4.2831 | 13 | A | G | 0.1647 |
| 2017 | 1-Jun-17 | VENTURA | STRAWBERRY (ALL OR UNSPEC) | Redacted4 | 4.2831 | 13 | A | G | 0.1647 |
| 2017 | 9-Jun-17 | VENTURA | STRAWBERRY (ALL OR UNSPEC) | Redacted4 | 4.955 | 15 | A | G | 0.1652 |
| 2017 | 15-Dec-17 | VENTURA | STRAWBERRY (ALL OR UNSPEC) | Redacted4 | 4.955 | 15 | A | G | 0.1652 |
| 2017 | 22-Dec-17 | VENTURA | STRAWBERRY (ALL OR UNSPEC) | Redacted4 | 0.6719 | 2 | A | G | 0.1680 |
| 2017 | 12-Jul-17 | MONTEREY | STRAWBERRY (ALL OR UNSPEC) | Redacted4 | 3.8632 | 10 | A | G | 0.1932 |
| 2017 | 29-Jun-17 | MONTEREY | STRAWBERRY (ALL OR UNSPEC) | Redacted4 | 3.8632 | 10 | A | G | 0.1932 |
| 2017 | 8-Jul-17 | MONTEREY | STRAWBERRY (ALL OR UNSPEC) | Redacted4 | 3.8632 | 10 | A | G | 0.1932 |
| 2017 | 8-Jul-17 | MONTEREY | STRAWBERRY (ALL OR UNSPEC) | Redacted4 | 3.1074 | 8 | A | G | 0.1942 |
| 2017 | 12-Jul-17 | MONTEREY | STRAWBERRY (ALL OR UNSPEC) | Redacted4 | 3.1074 | 8 | A | G | 0.1942 |
| 2017 | 9-Sep-17 | MONTEREY | STRAWBERRY (ALL OR UNSPEC) | Redacted4 | 4.4511 | 11.4 | A | G | 0.1952 |
| 2017 | 25-Aug-17 | MONTEREY | STRAWBERRY (ALL OR UNSPEC) | Redacted4 | 4.4511 | 11.4 | A | G | 0.1952 |
| 2017 | 9-Oct-17 | MONTEREY | STRAWBERRY (ALL OR UNSPEC) | Redacted4 | 4.4511 | 11.4 | A | G | 0.1952 |
| 2017 | 12-Jul-17 | MONTEREY | STRAWBERRY (ALL OR UNSPEC) | Redacted4 | 7.8104 | 20 | A | G | 0.1953 |
| 2017 | 6-Jul-17 | MONTEREY | STRAWBERRY (ALL OR UNSPEC) | Redacted4 | 7.8104 | 20 | A | G | 0.1953 |
| 2017 | 6-Jul-17 | MONTEREY | STRAWBERRY (ALL OR UNSPEC) | Redacted4 | 7.8104 | 20 | A | G | 0.1953 |
| 2017 | 26-Aug-17 | MONTEREY | STRAWBERRY (ALL OR UNSPEC) | Redacted4 | 6.3827 | 16.3 | A | G | 0.1958 |
| 2017 | 23-Sep-17 | MONTEREY | STRAWBERRY (ALL OR UNSPEC) | Redacted4 | 6.3827 | 16.3 | A | G | 0.1958 |
| 2017 | 12-Jul-17 | MONTEREY | STRAWBERRY (ALL OR UNSPEC) | Redacted4 | 8.2303 | 21 | A | G | 0.1960 |
| 2017 | 30-Jun-17 | MONTEREY | STRAWBERRY (ALL OR UNSPEC) | Redacted4 | 8.2303 | 21 | A | G | 0.1960 |
| 2017 | 30-Jan-17 | MONTEREY | STRAWBERRY (ALL OR UNSPEC) | Redacted4 | 5.8788 | 15 | A | G | 0.1960 |
| 2017 | 15-Jul-17 | MONTEREY | STRAWBERRY (ALL OR UNSPEC) | Redacted4 | 11.7576 | 30 | A | G | 0.1960 |
| 2017 | 29-Jun-17 | MONTEREY | STRAWBERRY (ALL OR UNSPEC) | Redacted4 | 14.1092 | 36 | A | G | 0.1960 |
| 2017 | 21-Sep-17 | MONTEREY | STRAWBERRY (ALL OR UNSPEC) | Redacted4 | 4.1152 | 10.5 | A | G | 0.1960 |
| 2017 | 7-Sep-17 | MONTEREY | STRAWBERRY (ALL OR UNSPEC) | Redacted4 | 4.1152 | 10.5 | A | G | 0.1960 |
| 2017 | 30-Jan-17 | MONTEREY | STRAWBERRY (ALL OR UNSPEC) | Redacted4 | 4.7031 | 12 | A | G | 0.1960 |
| 2017 | 6-Jul-17 | MONTEREY | STRAWBERRY (ALL OR UNSPEC) | Redacted4 | 4.7031 | 12 | A | G | 0.1960 |
| 2017 | 12-Jul-17 | MONTEREY | STRAWBERRY (ALL OR UNSPEC) | Redacted4 | 13.3533 | 34 | A | G | 0.1964 |
| 2017 | 30-Jun-17 | MONTEREY | STRAWBERRY (ALL OR UNSPEC) | Redacted4 | 13.3533 | 34 | A | G | 0.1964 |
| 2017 | 7-Sep-17 | MONTEREY | STRAWBERRY (ALL OR UNSPEC) | Redacted4 | 3.5273 | 8.98 | A | G | 0.1964 |
| 2017 | 6-Jul-17 | MONTEREY | STRAWBERRY (ALL OR UNSPEC) | Redacted4 | 12.1775 | 31 | A | G | 0.1964 |
| 2017 | 5-Jul-17 | MONTEREY | STRAWBERRY (ALL OR UNSPEC) | Redacted4 | 12.1775 | 31 | A | G | 0.1964 |
| 2017 | 6-Jul-17 | MONTEREY | STRAWBERRY (ALL OR UNSPEC) | Redacted4 | 12.1775 | 31 | A | G | 0.1964 |
| 2017 | 17-Jul-17 | MONTEREY | STRAWBERRY (ALL OR UNSPEC) | Redacted4 | 11.0018 | 28 | A | G | 0.1965 |
| 2017 | 23-Jul-17 | MONTEREY | STRAWBERRY (ALL OR UNSPEC) | Redacted4 | 20.8278 | 53 | A | G | 0.1965 |
| 2017 | 23-Jul-17 | MONTEREY | STRAWBERRY (ALL OR UNSPEC) | Redacted4 | 20.8278 | 53 | A | G | 0.1965 |
| 2017 | 18-Jul-17 | MONTEREY | STRAWBERRY (ALL OR UNSPEC) | Redacted4 | 20.8278 | 53 | A | G | 0.1965 |
| 2017 | 8-Jul-17 | MONTEREY | STRAWBERRY (ALL OR UNSPEC) | Redacted4 | 20.8278 | 53 | A | G | 0.1965 |
| 2017 | 1-Jul-17 | SANTA CRUZ | STRAWBERRY (ALL OR UNSPEC) | Redacted4 | 23.0953 | 58.77 | A | G | 0.1965 |
| 2017 | 14-Oct-17 | VENTURA | STRAWBERRY (ALL OR UNSPEC) | Redacted4 | 18.4763 | 47 | A | G | 0.1966 |
| 2017 | 6-Jul-17 | MONTEREY | STRAWBERRY (ALL OR UNSPEC) | Redacted4 | 17.3005 | 44 | A | G | 0.1966 |
| 2017 | 2-Aug-17 | MONTEREY | STRAWBERRY (ALL OR UNSPEC) | Redacted4 | 17.3005 | 44 | A | G | 0.1966 |
| 2017 | 25-Aug-17 | MONTEREY | STRAWBERRY (ALL OR UNSPEC) | Redacted4 | 8.6503 | 22 | A | G | 0.1966 |
| 2017 | 9-Sep-17 | MONTEREY | STRAWBERRY (ALL OR UNSPEC) | Redacted4 | 8.6503 | 22 | A | G | 0.1966 |
| 2017 | 9-Oct-17 | MONTEREY | STRAWBERRY (ALL OR UNSPEC) | Redacted4 | 8.6503 | 22 | A | G | 0.1966 |
| 2017 | 1-Jul-17 | MONTEREY | STRAWBERRY (ALL OR UNSPEC) | Redacted4 | 16.1247 | 41 | A | G | 0.1966 |
| 2017 | 11-Sep-17 | MONTEREY | STRAWBERRY (ALL OR UNSPEC) | Redacted4 | 8.0624 | 20.5 | A | G | 0.1966 |
| 2017 | 26-Sep-17 | MONTEREY | STRAWBERRY (ALL OR UNSPEC) | Redacted4 | 8.0624 | 20.5 | A | G | 0.1966 |
| 2017 | 25-Aug-17 | MONTEREY | STRAWBERRY (ALL OR UNSPEC) | Redacted4 | 7.4745 | 19 | A | G | 0.1967 |
| 2017 | 23-Sep-17 | MONTEREY | STRAWBERRY (ALL OR UNSPEC) | Redacted4 | 7.4745 | 19 | A | G | 0.1967 |
| 2017 | 12-Sep-17 | MONTEREY | STRAWBERRY (ALL OR UNSPEC) | Redacted4 | 7.4745 | 19 | A | G | 0.1967 |
| 2017 | 26-Aug-17 | MONTEREY | STRAWBERRY (ALL OR UNSPEC) | Redacted4 | 7.4745 | 19 | A | G | 0.1967 |
| 2017 | 28-Nov-17 | VENTURA | STRAWBERRY (ALL OR UNSPEC) | Redacted4 | 7.4745 | 19 | A | G | 0.1967 |
| 2017 | 16-Dec-17 | VENTURA | STRAWBERRY (ALL OR UNSPEC) | Redacted4 | 7.4745 | 19 | A | G | 0.1967 |
| 2017 | 7-Oct-17 | VENTURA | STRAWBERRY (ALL OR UNSPEC) | Redacted4 | 13.7732 | 35 | A | G | 0.1968 |
| 2017 | 25-Jul-17 | MONTEREY | STRAWBERRY (ALL OR UNSPEC) | Redacted4 | 32.6694 | 83 | A | G | 0.1968 |
| 2017 | 18-Jul-17 | MONTEREY | STRAWBERRY (ALL OR UNSPEC) | Redacted4 | 32.6694 | 83 | A | G | 0.1968 |
| 2017 | 25-Jul-17 | MONTEREY | STRAWBERRY (ALL OR UNSPEC) | Redacted4 | 32.6694 | 83 | A | G | 0.1968 |
| 2017 | 28-Jun-17 | MONTEREY | STRAWBERRY (ALL OR UNSPEC) | Redacted4 | 32.6694 | 83 | A | G | 0.1968 |
| 2017 | 15-Jul-17 | MONTEREY | STRAWBERRY (ALL OR UNSPEC) | Redacted4 | 32.6694 | 83 | A | G | 0.1968 |
| 2017 | 18-Jul-17 | MONTEREY | STRAWBERRY (ALL OR UNSPEC) | Redacted4 | 32.6694 | 83 | A | G | 0.1968 |
| 2017 | 8-Jul-17 | MONTEREY | STRAWBERRY (ALL OR UNSPEC) | Redacted4 | 32.6694 | 83 | A | G | 0.1968 |
| 2017 | 28-Oct-17 | VENTURA | STRAWBERRY (ALL OR UNSPEC) | Redacted4 | 54.337 | 138 | A | G | 0.1969 |
| 2017 | 16-Nov-17 | VENTURA | STRAWBERRY (ALL OR UNSPEC) | Redacted4 | 54.337 | 138 | A | G | 0.1969 |
| 2017 | 4-Sep-17 | VENTURA | STRAWBERRY (ALL OR UNSPEC) | Redacted4 | 59.8799 | 152 | A | G | 0.1970 |
| 2017 | 16-Oct-17 | VENTURA | STRAWBERRY (ALL OR UNSPEC) | Redacted4 | 35.8608 | 91 | A | G | 0.1970 |
| 2017 | 15-Dec-17 | VENTURA | STRAWBERRY (ALL OR UNSPEC) | Redacted4 | 24.4391 | 62 | A | G | 0.1971 |
| 2017 | 20-Dec-17 | VENTURA | STRAWBERRY (ALL OR UNSPEC) | Redacted4 | 28.9742 | 73.5 | A | G | 0.1971 |
| 2017 | 10-Jul-17 | MONTEREY | STRAWBERRY (ALL OR UNSPEC) | Redacted4 | 14.1931 | 36 | A | G | 0.1971 |
| 2017 | 17-Jul-17 | MONTEREY | STRAWBERRY (ALL OR UNSPEC) | Redacted4 | 14.1931 | 36 | A | G | 0.1971 |
| 2017 | 9-Jul-17 | MONTEREY | STRAWBERRY (ALL OR UNSPEC) | Redacted4 | 14.1931 | 36 | A | G | 0.1971 |
| 2017 | 9-Jul-17 | MONTEREY | STRAWBERRY (ALL OR UNSPEC) | Redacted4 | 14.1931 | 36 | A | G | 0.1971 |
| 2017 | 10-Jul-17 | MONTEREY | STRAWBERRY (ALL OR UNSPEC) | Redacted4 | 14.1931 | 36 | A | G | 0.1971 |
| 2017 | 29-Jun-17 | MONTEREY | STRAWBERRY (ALL OR UNSPEC) | Redacted4 | 18.1403 | 46 | A | G | 0.1972 |
| 2017 | 24-Aug-17 | MONTEREY | STRAWBERRY (ALL OR UNSPEC) | Redacted4 | 9.0702 | 23 | A | G | 0.1972 |
| 2017 | 29-Sep-17 | MONTEREY | STRAWBERRY (ALL OR UNSPEC) | Redacted4 | 9.0702 | 23 | A | G | 0.1972 |
| 2017 | 7-Oct-17 | VENTURA | STRAWBERRY (ALL OR UNSPEC) | Redacted4 | 22.0875 | 56 | A | G | 0.1972 |
| 2017 | 5-Sep-17 | VENTURA | STRAWBERRY (ALL OR UNSPEC) | Redacted4 | 13.0174 | 33 | A | G | 0.1972 |
| 2017 | 18-Oct-17 | VENTURA | STRAWBERRY (ALL OR UNSPEC) | Redacted4 | 13.0174 | 33 | A | G | 0.1972 |
| 2017 | 16-Nov-17 | VENTURA | STRAWBERRY (ALL OR UNSPEC) | Redacted4 | 13.0174 | 33 | A | G | 0.1972 |
| 2017 | 28-Oct-17 | VENTURA | STRAWBERRY (ALL OR UNSPEC) | Redacted4 | 13.0174 | 33 | A | G | 0.1972 |
| 2017 | 13-Jul-17 | MONTEREY | STRAWBERRY (ALL OR UNSPEC) | Redacted4 | 7.8944 | 20 | A | G | 0.1974 |
| 2017 | 18-Jul-17 | MONTEREY | STRAWBERRY (ALL OR UNSPEC) | Redacted4 | 7.8944 | 20 | A | G | 0.1974 |
| 2017 | 2-Aug-17 | MONTEREY | STRAWBERRY (ALL OR UNSPEC) | Redacted4 | 7.8944 | 20 | A | G | 0.1974 |
| 2017 | 12-Jul-17 | MONTEREY | STRAWBERRY (ALL OR UNSPEC) | Redacted4 | 3.9472 | 10 | A | G | 0.1974 |
| 2017 | 3-Jul-17 | MONTEREY | STRAWBERRY (ALL OR UNSPEC) | Redacted4 | 3.9472 | 10 | A | G | 0.1974 |
| 2017 | 12-Jul-17 | MONTEREY | STRAWBERRY (ALL OR UNSPEC) | Redacted4 | 7.8944 | 20 | A | G | 0.1974 |
| 2017 | 25-Jul-17 | MONTEREY | STRAWBERRY (ALL OR UNSPEC) | Redacted4 | 7.8944 | 20 | A | G | 0.1974 |
| 2017 | 6-Jul-17 | MONTEREY | STRAWBERRY (ALL OR UNSPEC) | Redacted4 | 15.7888 | 40 | A | G | 0.1974 |
| 2017 | 12-Jul-17 | MONTEREY | STRAWBERRY (ALL OR UNSPEC) | Redacted4 | 7.8944 | 20 | A | G | 0.1974 |
| 2017 | 19-Jul-17 | MONTEREY | STRAWBERRY (ALL OR UNSPEC) | Redacted4 | 7.8944 | 20 | A | G | 0.1974 |
| 2017 | 3-Jul-17 | MONTEREY | STRAWBERRY (ALL OR UNSPEC) | Redacted4 | 3.9472 | 10 | A | G | 0.1974 |
| 2017 | 30-Nov-17 | VENTURA | STRAWBERRY (ALL OR UNSPEC) | Redacted4 | 3.3593 | 8.5 | A | G | 0.1976 |
| 2017 | 22-Sep-17 | MONTEREY | STRAWBERRY (ALL OR UNSPEC) | Redacted4 | 9.4901 | 24 | A | G | 0.1977 |
| 2017 | 8-Sep-17 | MONTEREY | STRAWBERRY (ALL OR UNSPEC) | Redacted4 | 9.4901 | 24 | A | G | 0.1977 |
| 2017 | 25-Aug-17 | MONTEREY | STRAWBERRY (ALL OR UNSPEC) | Redacted4 | 8.1464 | 20.6 | A | G | 0.1977 |
| 2017 | 29-Sep-17 | MONTEREY | STRAWBERRY (ALL OR UNSPEC) | Redacted4 | 8.1464 | 20.6 | A | G | 0.1977 |
| 2017 | 11-Jul-17 | MONTEREY | STRAWBERRY (ALL OR UNSPEC) | Redacted4 | 2.7714 | 7 | A | G | 0.1980 |
| 2017 | 1-Jul-17 | MONTEREY | STRAWBERRY (ALL OR UNSPEC) | Redacted4 | 2.7714 | 7 | A | G | 0.1980 |
| 2017 | 22-Jul-17 | MONTEREY | STRAWBERRY (ALL OR UNSPEC) | Redacted4 | 5.5429 | 14 | A | G | 0.1980 |
| 2017 | 20-Jul-17 | MONTEREY | STRAWBERRY (ALL OR UNSPEC) | Redacted4 | 3.1914 | 8 | A | G | 0.1995 |
| 2017 | 20-Jul-17 | MONTEREY | STRAWBERRY (ALL OR UNSPEC) | Redacted4 | 3.1914 | 8 | A | G | 0.1995 |
| 2017 | 13-Jul-17 | MONTEREY | STRAWBERRY (ALL OR UNSPEC) | Redacted4 | 3.1914 | 8 | A | G | 0.1995 |
| 2017 | 17-Oct-17 | MONTEREY | STRAWBERRY (ALL OR UNSPEC) | Redacted4 | 8.2303 | 18 | A | G | 0.2286 |
| 2017 | 2-Oct-17 | MONTEREY | STRAWBERRY (ALL OR UNSPEC) | Redacted4 | 8.2303 | 18 | A | G | 0.2286 |
| 2017 | 12-Oct-17 | MONTEREY | STRAWBERRY (ALL OR UNSPEC) | Redacted4 | 8.2303 | 18 | A | G | 0.2286 |
| 2017 | 31-Aug-17 | MONTEREY | STRAWBERRY (ALL OR UNSPEC) | Redacted4 | 9.1542 | 20 | A | G | 0.2289 |
| 2017 | 16-Sep-17 | MONTEREY | STRAWBERRY (ALL OR UNSPEC) | Redacted4 | 9.1542 | 20 | A | G | 0.2289 |
| 2017 | 30-Sep-17 | MONTEREY | STRAWBERRY (ALL OR UNSPEC) | Redacted4 | 9.1542 | 20 | A | G | 0.2289 |
| 2017 | 3-Nov-17 | MONTEREY | STRAWBERRY (ALL OR UNSPEC) | Redacted4 | 9.1542 | 20 | A | G | 0.2289 |
| 2017 | 14-Aug-17 | MONTEREY | STRAWBERRY (ALL OR UNSPEC) | Redacted4 | 9.1542 | 20 | A | G | 0.2289 |
| 2017 | 16-Sep-17 | MONTEREY | STRAWBERRY (ALL OR UNSPEC) | Redacted4 | 9.1542 | 20 | A | G | 0.2289 |
| 2017 | 19-Sep-17 | MONTEREY | STRAWBERRY (ALL OR UNSPEC) | Redacted4 | 9.1542 | 20 | A | G | 0.2289 |
| 2017 | 30-Sep-17 | MONTEREY | STRAWBERRY (ALL OR UNSPEC) | Redacted4 | 9.1542 | 20 | A | G | 0.2289 |
| 2017 | 12-Oct-17 | MONTEREY | STRAWBERRY (ALL OR UNSPEC) | Redacted4 | 9.1542 | 20 | A | G | 0.2289 |
| 2017 | 4-Sep-17 | MONTEREY | STRAWBERRY (ALL OR UNSPEC) | Redacted4 | 9.1542 | 20 | A | G | 0.2289 |
| 2017 | 23-Sep-17 | MONTEREY | STRAWBERRY (ALL OR UNSPEC) | Redacted4 | 9.1542 | 20 | A | G | 0.2289 |
| 2017 | 29-Sep-17 | MONTEREY | STRAWBERRY (ALL OR UNSPEC) | Redacted4 | 9.1542 | 20 | A | G | 0.2289 |
| 2017 | 5-Nov-17 | MONTEREY | STRAWBERRY (ALL OR UNSPEC) | Redacted4 | 9.1542 | 20 | A | G | 0.2289 |
| 2017 | 17-Nov-17 | MONTEREY | STRAWBERRY (ALL OR UNSPEC) | Redacted4 | 9.1542 | 20 | A | G | 0.2289 |
| 2017 | 20-Sep-17 | MONTEREY | STRAWBERRY (ALL OR UNSPEC) | Redacted4 | 9.1542 | 20 | A | G | 0.2289 |
| 2017 | 4-Oct-17 | MONTEREY | STRAWBERRY (ALL OR UNSPEC) | Redacted4 | 9.1542 | 20 | A | G | 0.2289 |
| 2017 | 14-Oct-17 | MONTEREY | STRAWBERRY (ALL OR UNSPEC) | Redacted4 | 9.1542 | 20 | A | G | 0.2289 |
| 2017 | 10-Oct-17 | MONTEREY | STRAWBERRY (ALL OR UNSPEC) | Redacted4 | 9.1542 | 20 | A | G | 0.2289 |
| 2017 | 26-Aug-17 | MONTEREY | STRAWBERRY (ALL OR UNSPEC) | Redacted4 | 9.1542 | 20 | A | G | 0.2289 |
| 2017 | 11-Sep-17 | MONTEREY | STRAWBERRY (ALL OR UNSPEC) | Redacted4 | 9.1542 | 20 | A | G | 0.2289 |
| 2017 | 26-Sep-17 | MONTEREY | STRAWBERRY (ALL OR UNSPEC) | Redacted4 | 9.1542 | 20 | A | G | 0.2289 |
| 2017 | 9-Nov-17 | MONTEREY | STRAWBERRY (ALL OR UNSPEC) | Redacted4 | 9.1542 | 20 | A | G | 0.2289 |
| 2017 | 20-Sep-17 | MONTEREY | STRAWBERRY (ALL OR UNSPEC) | Redacted4 | 22.0036 | 48 | A | G | 0.2292 |
| 2017 | 7-Nov-17 | MONTEREY | STRAWBERRY (ALL OR UNSPEC) | Redacted4 | 17.8884 | 39 | A | G | 0.2293 |
| 2017 | 27-Sep-17 | MONTEREY | STRAWBERRY (ALL OR UNSPEC) | Redacted4 | 12.8494 | 28 | A | G | 0.2295 |
| 2017 | 29-Nov-17 | MONTEREY | STRAWBERRY (ALL OR UNSPEC) | Redacted4 | 25.6988 | 56 | A | G | 0.2295 |
| 2017 | 14-Sep-17 | MONTEREY | STRAWBERRY (ALL OR UNSPEC) | Redacted4 | 25.6988 | 56 | A | G | 0.2295 |
| 2017 | 26-Oct-17 | MONTEREY | STRAWBERRY (ALL OR UNSPEC) | Redacted4 | 25.6988 | 56 | A | G | 0.2295 |
| 2017 | 9-Nov-17 | MONTEREY | STRAWBERRY (ALL OR UNSPEC) | Redacted4 | 12.8494 | 28 | A | G | 0.2295 |
| 2017 | 14-Oct-17 | MONTEREY | STRAWBERRY (ALL OR UNSPEC) | Redacted4 | 12.8494 | 28 | A | G | 0.2295 |
| 2017 | 10-Oct-17 | MONTEREY | STRAWBERRY (ALL OR UNSPEC) | Redacted4 | 12.8494 | 28 | A | G | 0.2295 |
| 2017 | 23-Nov-17 | MONTEREY | STRAWBERRY (ALL OR UNSPEC) | Redacted4 | 25.6988 | 56 | A | G | 0.2295 |
| 2017 | 28-Aug-17 | MONTEREY | STRAWBERRY (ALL OR UNSPEC) | Redacted4 | 12.8494 | 28 | A | G | 0.2295 |
| 2017 | 20-Sep-17 | MONTEREY | STRAWBERRY (ALL OR UNSPEC) | Redacted4 | 12.8494 | 28 | A | G | 0.2295 |
| 2017 | 6-Sep-17 | MONTEREY | STRAWBERRY (ALL OR UNSPEC) | Redacted4 | 46.3586 | 101 | A | G | 0.2295 |
| 2017 | 25-Nov-17 | MONTEREY | STRAWBERRY (ALL OR UNSPEC) | Redacted4 | 33.5092 | 73 | A | G | 0.2295 |
| 2017 | 15-Nov-17 | MONTEREY | STRAWBERRY (ALL OR UNSPEC) | Redacted4 | 13.7732 | 30 | A | G | 0.2296 |
| 2017 | 1-Sep-17 | MONTEREY | STRAWBERRY (ALL OR UNSPEC) | Redacted4 | 13.7732 | 30 | A | G | 0.2296 |
| 2017 | 24-Oct-17 | MONTEREY | STRAWBERRY (ALL OR UNSPEC) | Redacted4 | 13.7732 | 30 | A | G | 0.2296 |
| 2017 | 11-Aug-17 | MONTEREY | STRAWBERRY (ALL OR UNSPEC) | Redacted4 | 13.7732 | 30 | A | G | 0.2296 |
| 2017 | 26-Sep-17 | MONTEREY | STRAWBERRY (ALL OR UNSPEC) | Redacted4 | 13.7732 | 30 | A | G | 0.2296 |
| 2017 | 14-Sep-17 | MONTEREY | STRAWBERRY (ALL OR UNSPEC) | Redacted4 | 13.7732 | 30 | A | G | 0.2296 |
| 2017 | 14-Oct-17 | MONTEREY | STRAWBERRY (ALL OR UNSPEC) | Redacted4 | 13.7732 | 30 | A | G | 0.2296 |
| 2017 | 21-Sep-17 | MONTEREY | STRAWBERRY (ALL OR UNSPEC) | Redacted4 | 13.7732 | 30 | A | G | 0.2296 |
| 2017 | 13-Nov-17 | MONTEREY | STRAWBERRY (ALL OR UNSPEC) | Redacted4 | 51.9015 | 113 | A | G | 0.2297 |
| 2017 | 4-Nov-17 | MONTEREY | STRAWBERRY (ALL OR UNSPEC) | Redacted4 | 51.9015 | 113 | A | G | 0.2297 |
| 2017 | 27-Sep-17 | MONTEREY | STRAWBERRY (ALL OR UNSPEC) | Redacted4 | 38.1283 | 83 | A | G | 0.2297 |
| 2017 | 3-Oct-17 | MONTEREY | STRAWBERRY (ALL OR UNSPEC) | Redacted4 | 38.1283 | 83 | A | G | 0.2297 |
| 2017 | 11-Nov-17 | MONTEREY | STRAWBERRY (ALL OR UNSPEC) | Redacted4 | 38.1283 | 83 | A | G | 0.2297 |
| 2017 | 30-Aug-17 | MONTEREY | STRAWBERRY (ALL OR UNSPEC) | Redacted4 | 38.1283 | 83 | A | G | 0.2297 |
| 2017 | 13-Oct-17 | MONTEREY | STRAWBERRY (ALL OR UNSPEC) | Redacted4 | 38.1283 | 83 | A | G | 0.2297 |
| 2017 | 28-Nov-17 | MONTEREY | STRAWBERRY (ALL OR UNSPEC) | Redacted4 | 38.1283 | 83 | A | G | 0.2297 |
| 2017 | 3-Oct-17 | MONTEREY | STRAWBERRY (ALL OR UNSPEC) | Redacted4 | 38.1283 | 83 | A | G | 0.2297 |
| 2017 | 4-Oct-17 | MONTEREY | STRAWBERRY (ALL OR UNSPEC) | Redacted4 | 38.1283 | 83 | A | G | 0.2297 |
| 2017 | 6-Nov-17 | MONTEREY | STRAWBERRY (ALL OR UNSPEC) | Redacted4 | 38.1283 | 83 | A | G | 0.2297 |
| 2017 | 22-Sep-17 | MONTEREY | STRAWBERRY (ALL OR UNSPEC) | Redacted4 | 38.1283 | 83 | A | G | 0.2297 |
| 2017 | 25-Sep-17 | MONTEREY | STRAWBERRY (ALL OR UNSPEC) | Redacted4 | 38.1283 | 83 | A | G | 0.2297 |
| 2017 | 4-Oct-17 | MONTEREY | STRAWBERRY (ALL OR UNSPEC) | Redacted4 | 38.1283 | 83 | A | G | 0.2297 |
| 2017 | 6-Oct-17 | MONTEREY | STRAWBERRY (ALL OR UNSPEC) | Redacted4 | 15.6208 | 34 | A | G | 0.2297 |
| 2017 | 1-Nov-17 | MONTEREY | STRAWBERRY (ALL OR UNSPEC) | Redacted4 | 15.6208 | 34 | A | G | 0.2297 |
| 2017 | 29-Aug-17 | MONTEREY | STRAWBERRY (ALL OR UNSPEC) | Redacted4 | 24.3551 | 53 | A | G | 0.2298 |
| 2017 | 9-Sep-17 | MONTEREY | STRAWBERRY (ALL OR UNSPEC) | Redacted4 | 24.3551 | 53 | A | G | 0.2298 |
| 2017 | 15-Aug-17 | MONTEREY | STRAWBERRY (ALL OR UNSPEC) | Redacted4 | 24.3551 | 53 | A | G | 0.2298 |
| 2017 | 3-Oct-17 | MONTEREY | STRAWBERRY (ALL OR UNSPEC) | Redacted4 | 16.5447 | 36 | A | G | 0.2298 |
| 2017 | 16-Sep-17 | MONTEREY | STRAWBERRY (ALL OR UNSPEC) | Redacted4 | 16.5447 | 36 | A | G | 0.2298 |
| 2017 | 31-Aug-17 | MONTEREY | STRAWBERRY (ALL OR UNSPEC) | Redacted4 | 16.5447 | 36 | A | G | 0.2298 |
| 2017 | 30-Sep-17 | MONTEREY | STRAWBERRY (ALL OR UNSPEC) | Redacted4 | 16.5447 | 36 | A | G | 0.2298 |
| 2017 | 4-Nov-17 | MONTEREY | STRAWBERRY (ALL OR UNSPEC) | Redacted4 | 16.5447 | 36 | A | G | 0.2298 |
| 2017 | 9-Oct-17 | MONTEREY | STRAWBERRY (ALL OR UNSPEC) | Redacted4 | 16.5447 | 36 | A | G | 0.2298 |
| 2017 | 15-Nov-17 | MONTEREY | STRAWBERRY (ALL OR UNSPEC) | Redacted4 | 16.5447 | 36 | A | G | 0.2298 |
| 2017 | 21-Sep-17 | MONTEREY | STRAWBERRY (ALL OR UNSPEC) | Redacted4 | 16.5447 | 36 | A | G | 0.2298 |
| 2017 | 30-Sep-17 | MONTEREY | STRAWBERRY (ALL OR UNSPEC) | Redacted4 | 16.5447 | 36 | A | G | 0.2298 |
| 2017 | 11-Oct-17 | MONTEREY | STRAWBERRY (ALL OR UNSPEC) | Redacted4 | 25.2789 | 55 | A | G | 0.2298 |
| 2017 | 17-Oct-17 | MONTEREY | STRAWBERRY (ALL OR UNSPEC) | Redacted4 | 25.2789 | 55 | A | G | 0.2298 |
| 2017 | 26-Oct-17 | MONTEREY | STRAWBERRY (ALL OR UNSPEC) | Redacted4 | 18.3923 | 40 | A | G | 0.2299 |
| 2017 | 26-Aug-17 | MONTEREY | STRAWBERRY (ALL OR UNSPEC) | Redacted4 | 18.3923 | 40 | A | G | 0.2299 |
| 2017 | 18-Oct-17 | MONTEREY | STRAWBERRY (ALL OR UNSPEC) | Redacted4 | 18.3923 | 40 | A | G | 0.2299 |
| 2017 | 23-Nov-17 | MONTEREY | STRAWBERRY (ALL OR UNSPEC) | Redacted4 | 19.3161 | 42 | A | G | 0.2300 |
| 2017 | 11-Oct-17 | MONTEREY | STRAWBERRY (ALL OR UNSPEC) | Redacted4 | 19.3161 | 42 | A | G | 0.2300 |
| 2017 | 2-Oct-17 | MONTEREY | STRAWBERRY (ALL OR UNSPEC) | Redacted4 | 9.6581 | 21 | A | G | 0.2300 |
| 2017 | 30-Aug-17 | MONTEREY | STRAWBERRY (ALL OR UNSPEC) | Redacted4 | 20.2399 | 44 | A | G | 0.2300 |
| 2017 | 7-Sep-17 | MONTEREY | STRAWBERRY (ALL OR UNSPEC) | Redacted4 | 20.2399 | 44 | A | G | 0.2300 |
| 2017 | 23-Sep-17 | MONTEREY | STRAWBERRY (ALL OR UNSPEC) | Redacted4 | 20.2399 | 44 | A | G | 0.2300 |
| 2017 | 11-Nov-17 | MONTEREY | STRAWBERRY (ALL OR UNSPEC) | Redacted4 | 20.2399 | 44 | A | G | 0.2300 |
| 2017 | 25-Nov-17 | MONTEREY | STRAWBERRY (ALL OR UNSPEC) | Redacted4 | 20.2399 | 44 | A | G | 0.2300 |
| 2017 | 13-Oct-17 | MONTEREY | STRAWBERRY (ALL OR UNSPEC) | Redacted4 | 22.0875 | 48 | A | G | 0.2301 |
| 2017 | 7-Nov-17 | MONTEREY | STRAWBERRY (ALL OR UNSPEC) | Redacted4 | 22.0875 | 48 | A | G | 0.2301 |
| 2017 | 15-Nov-17 | MONTEREY | STRAWBERRY (ALL OR UNSPEC) | Redacted4 | 22.0875 | 48 | A | G | 0.2301 |
| 2017 | 27-Sep-17 | MONTEREY | STRAWBERRY (ALL OR UNSPEC) | Redacted4 | 22.0875 | 48 | A | G | 0.2301 |
| 2017 | 28-Aug-17 | MONTEREY | STRAWBERRY (ALL OR UNSPEC) | Redacted4 | 22.0875 | 48 | A | G | 0.2301 |
| 2017 | 12-Oct-17 | MONTEREY | STRAWBERRY (ALL OR UNSPEC) | Redacted4 | 22.0875 | 48 | A | G | 0.2301 |
| 2017 | 4-Sep-17 | MONTEREY | STRAWBERRY (ALL OR UNSPEC) | Redacted4 | 22.0875 | 48 | A | G | 0.2301 |
| 2017 | 27-Oct-17 | MONTEREY | STRAWBERRY (ALL OR UNSPEC) | Redacted4 | 22.0875 | 48 | A | G | 0.2301 |
| 2017 | 2-Nov-17 | MONTEREY | STRAWBERRY (ALL OR UNSPEC) | Redacted4 | 22.0875 | 48 | A | G | 0.2301 |
| 2017 | 5-Oct-17 | MONTEREY | STRAWBERRY (ALL OR UNSPEC) | Redacted4 | 22.0875 | 48 | A | G | 0.2301 |
| 2017 | 24-Nov-17 | MONTEREY | STRAWBERRY (ALL OR UNSPEC) | Redacted4 | 22.0875 | 48 | A | G | 0.2301 |
| 2017 | 24-Sep-17 | MONTEREY | STRAWBERRY (ALL OR UNSPEC) | Redacted4 | 22.0875 | 48 | A | G | 0.2301 |
| 2017 | 19-Sep-17 | MONTEREY | STRAWBERRY (ALL OR UNSPEC) | Redacted4 | 22.0875 | 48 | A | G | 0.2301 |
| 2017 | 8-Nov-17 | MONTEREY | STRAWBERRY (ALL OR UNSPEC) | Redacted4 | 22.0875 | 48 | A | G | 0.2301 |
| 2017 | 7-Nov-17 | MONTEREY | STRAWBERRY (ALL OR UNSPEC) | Redacted4 | 22.0875 | 48 | A | G | 0.2301 |
| 2017 | 17-Nov-17 | MONTEREY | STRAWBERRY (ALL OR UNSPEC) | Redacted4 | 22.0875 | 48 | A | G | 0.2301 |
| 2017 | 11-Oct-17 | MONTEREY | STRAWBERRY (ALL OR UNSPEC) | Redacted4 | 14.2771 | 31 | A | G | 0.2303 |
| 2017 | 11-Oct-17 | MONTEREY | STRAWBERRY (ALL OR UNSPEC) | Redacted4 | 14.2771 | 31 | A | G | 0.2303 |
| 2017 | 10-Nov-17 | MONTEREY | STRAWBERRY (ALL OR UNSPEC) | Redacted4 | 14.2771 | 31 | A | G | 0.2303 |
| 2017 | 15-Oct-17 | MONTEREY | STRAWBERRY (ALL OR UNSPEC) | Redacted4 | 14.2771 | 31 | A | G | 0.2303 |
| 2017 | 22-Sep-17 | MONTEREY | STRAWBERRY (ALL OR UNSPEC) | Redacted4 | 14.2771 | 31 | A | G | 0.2303 |
| 2017 | 11-Nov-17 | MONTEREY | STRAWBERRY (ALL OR UNSPEC) | Redacted4 | 14.2771 | 31 | A | G | 0.2303 |
| 2017 | 17-Oct-17 | MONTEREY | STRAWBERRY (ALL OR UNSPEC) | Redacted4 | 14.2771 | 31 | A | G | 0.2303 |
| 2017 | 6-Sep-17 | MONTEREY | STRAWBERRY (ALL OR UNSPEC) | Redacted4 | 14.2771 | 31 | A | G | 0.2303 |
| 2017 | 22-Aug-17 | MONTEREY | STRAWBERRY (ALL OR UNSPEC) | Redacted4 | 14.2771 | 31 | A | G | 0.2303 |
| 2017 | 21-Oct-17 | MONTEREY | STRAWBERRY (ALL OR UNSPEC) | Redacted4 | 5.5429 | 12 | A | G | 0.2310 |
| 2017 | 8-Nov-17 | MONTEREY | STRAWBERRY (ALL OR UNSPEC) | Redacted4 | 5.5429 | 12 | A | G | 0.2310 |
| 2017 | 3-Oct-17 | MONTEREY | STRAWBERRY (ALL OR UNSPEC) | Redacted4 | 5.5429 | 12 | A | G | 0.2310 |
| 2017 | 28-Oct-17 | MONTEREY | STRAWBERRY (ALL OR UNSPEC) | Redacted4 | 5.5429 | 12 | A | G | 0.2310 |
| 2017 | 30-Sep-17 | MONTEREY | STRAWBERRY (ALL OR UNSPEC) | Redacted4 | 4.6191 | 10 | A | G | 0.2310 |
| 2017 | 30-Sep-17 | MONTEREY | STRAWBERRY (ALL OR UNSPEC) | Redacted4 | 4.6191 | 10 | A | G | 0.2310 |
| 2017 | 1-Nov-17 | MONTEREY | STRAWBERRY (ALL OR UNSPEC) | Redacted4 | 4.6191 | 10 | A | G | 0.2310 |
| 2017 | 25-Oct-17 | MONTEREY | STRAWBERRY (ALL OR UNSPEC) | Redacted4 | 4.6191 | 10 | A | G | 0.2310 |
| 2017 | 9-Nov-17 | MONTEREY | STRAWBERRY (ALL OR UNSPEC) | Redacted4 | 4.6191 | 10 | A | G | 0.2310 |
| 2017 | 25-Sep-17 | MONTEREY | STRAWBERRY (ALL OR UNSPEC) | Redacted4 | 4.6191 | 10 | A | G | 0.2310 |
| 2017 | 9-Oct-17 | MONTEREY | STRAWBERRY (ALL OR UNSPEC) | Redacted4 | 4.6191 | 10 | A | G | 0.2310 |
| 2017 | 22-Nov-17 | MONTEREY | STRAWBERRY (ALL OR UNSPEC) | Redacted4 | 4.6191 | 10 | A | G | 0.2310 |
| 2017 | 14-Oct-17 | MONTEREY | STRAWBERRY (ALL OR UNSPEC) | Redacted4 | 4.6191 | 10 | A | G | 0.2310 |
| 2017 | 20-Sep-17 | MONTEREY | STRAWBERRY (ALL OR UNSPEC) | Redacted4 | 3.6953 | 8 | A | G | 0.2310 |
| 2017 | 30-Sep-17 | MONTEREY | STRAWBERRY (ALL OR UNSPEC) | Redacted4 | 3.6953 | 8 | A | G | 0.2310 |
| 2017 | 13-Sep-17 | MONTEREY | STRAWBERRY (ALL OR UNSPEC) | Redacted4 | 3.6953 | 8 | A | G | 0.2310 |
| 2017 | 16-Oct-17 | MONTEREY | STRAWBERRY (ALL OR UNSPEC) | Redacted4 | 3.6953 | 8 | A | G | 0.2310 |
| 2017 | 16-Sep-17 | MONTEREY | STRAWBERRY (ALL OR UNSPEC) | Redacted4 | 3.6953 | 8 | A | G | 0.2310 |
| 2017 | 19-Sep-17 | MONTEREY | STRAWBERRY (ALL OR UNSPEC) | Redacted4 | 3.6953 | 8 | A | G | 0.2310 |
| 2017 | 25-Nov-17 | MONTEREY | STRAWBERRY (ALL OR UNSPEC) | Redacted4 | 3.6953 | 8 | A | G | 0.2310 |
| 2017 | 3-Oct-17 | MONTEREY | STRAWBERRY (ALL OR UNSPEC) | Redacted4 | 3.6953 | 8 | A | G | 0.2310 |
| 2017 | 25-Aug-17 | MONTEREY | STRAWBERRY (ALL OR UNSPEC) | Redacted4 | 3.6953 | 8 | A | G | 0.2310 |
| 2017 | 28-Sep-17 | MONTEREY | STRAWBERRY (ALL OR UNSPEC) | Redacted4 | 3.6953 | 8 | A | G | 0.2310 |
| 2017 | 21-Apr-17 | SANTA CRUZ | STRAWBERRY (ALL OR UNSPEC) | Redacted4 | 0.5039 | 1 | A | G | 0.2520 |
| 2017 | 4-Jul-17 | SANTA BARBARA | STRAWBERRY (ALL OR UNSPEC) | Redacted4 | 11.2537 | 21.59 | A | G | 0.2606 |
| 2017 | 8-Jun-17 | VENTURA | STRAWBERRY (ALL OR UNSPEC) | Redacted4 | 7.3065 | 14 | A | G | 0.2609 |
| 2017 | 3-Jun-17 | VENTURA | STRAWBERRY (ALL OR UNSPEC) | Redacted4 | 7.3065 | 14 | A | G | 0.2609 |
| 2017 | 21-Nov-17 | VENTURA | STRAWBERRY (ALL OR UNSPEC) | Redacted4 | 12.0096 | 23 | A | G | 0.2611 |
| 2017 | 13-Jul-17 | VENTURA | STRAWBERRY (ALL OR UNSPEC) | Redacted4 | 3.5273 | 6.75 | A | G | 0.2613 |
| 2017 | 1-Dec-17 | VENTURA | STRAWBERRY (ALL OR UNSPEC) | Redacted4 | 11.5057 | 22 | A | G | 0.2615 |
| 2017 | 20-May-17 | VENTURA | STRAWBERRY (ALL OR UNSPEC) | Redacted4 | 15.7048 | 30 | A | G | 0.2617 |
| 2017 | 21-Jun-17 | VENTURA | STRAWBERRY (ALL OR UNSPEC) | Redacted4 | 15.7048 | 30 | A | G | 0.2617 |
| 2017 | 9-Jun-17 | VENTURA | STRAWBERRY (ALL OR UNSPEC) | Redacted4 | 15.7048 | 30 | A | G | 0.2617 |
| 2017 | 3-Jun-17 | VENTURA | STRAWBERRY (ALL OR UNSPEC) | Redacted4 | 15.7048 | 30 | A | G | 0.2617 |
| 2017 | 2-Aug-17 | SANTA BARBARA | STRAWBERRY (ALL OR UNSPEC) | Redacted4 | 21.9196 | 41.86 | A | G | 0.2618 |
| 2017 | 13-Jun-17 | VENTURA | STRAWBERRY (ALL OR UNSPEC) | Redacted4 | 17.8044 | 34 | A | G | 0.2618 |
| 2017 | 2-Jun-17 | VENTURA | STRAWBERRY (ALL OR UNSPEC) | Redacted4 | 17.8044 | 34 | A | G | 0.2618 |
| 2017 | 18-May-17 | VENTURA | STRAWBERRY (ALL OR UNSPEC) | Redacted4 | 17.8044 | 34 | A | G | 0.2618 |
| 2017 | 26-Jun-17 | VENTURA | STRAWBERRY (ALL OR UNSPEC) | Redacted4 | 17.8044 | 34 | A | G | 0.2618 |
| 2017 | 21-Nov-17 | VENTURA | STRAWBERRY (ALL OR UNSPEC) | Redacted4 | 11.0018 | 21 | A | G | 0.2619 |
| 2017 | 30-Nov-17 | VENTURA | STRAWBERRY (ALL OR UNSPEC) | Redacted4 | 11.0018 | 21 | A | G | 0.2619 |
| 2017 | 25-Jul-17 | SANTA BARBARA | STRAWBERRY (ALL OR UNSPEC) | Redacted4 | 38.4642 | 73.41 | A | G | 0.2620 |
| 2017 | 4-Aug-17 | SANTA BARBARA | STRAWBERRY (ALL OR UNSPEC) | Redacted4 | 16.5447 | 31.55 | A | G | 0.2622 |
| 2017 | 13-Jul-17 | MONTEREY | STRAWBERRY (ALL OR UNSPEC) | Redacted4 | 12.8494 | 24.5 | A | G | 0.2622 |
| 2017 | 5-Aug-17 | SANTA BARBARA | STRAWBERRY (ALL OR UNSPEC) | Redacted4 | 19.3161 | 36.83 | A | G | 0.2622 |
| 2017 | 3-Jun-17 | MONTEREY | STRAWBERRY (ALL OR UNSPEC) | Redacted4 | 65.5908 | 125 | A | G | 0.2624 |
| 2017 | 3-Aug-17 | MONTEREY | STRAWBERRY (ALL OR UNSPEC) | Redacted4 | 31.4936 | 60 | A | G | 0.2624 |
| 2017 | 16-Jun-17 | VENTURA | STRAWBERRY (ALL OR UNSPEC) | Redacted4 | 33.5932 | 64 | A | G | 0.2624 |
| 2017 | 12-May-17 | VENTURA | STRAWBERRY (ALL OR UNSPEC) | Redacted4 | 33.5932 | 64 | A | G | 0.2624 |
| 2017 | 27-Jul-17 | MONTEREY | STRAWBERRY (ALL OR UNSPEC) | Redacted4 | 35.6928 | 68 | A | G | 0.2624 |
| 2017 | 17-Jun-17 | VENTURA | STRAWBERRY (ALL OR UNSPEC) | Redacted4 | 10.4979 | 20 | A | G | 0.2624 |
| 2017 | 31-May-17 | VENTURA | STRAWBERRY (ALL OR UNSPEC) | Redacted4 | 10.4979 | 20 | A | G | 0.2624 |
| 2017 | 10-Jun-17 | VENTURA | STRAWBERRY (ALL OR UNSPEC) | Redacted4 | 10.4979 | 20 | A | G | 0.2624 |
| 2017 | 3-Aug-17 | MONTEREY | STRAWBERRY (ALL OR UNSPEC) | Redacted4 | 24.691 | 47 | A | G | 0.2627 |
| 2017 | 3-Jun-17 | MONTEREY | STRAWBERRY (ALL OR UNSPEC) | Redacted4 | 36.7846 | 70 | A | G | 0.2627 |
| 2017 | 17-May-17 | VENTURA | STRAWBERRY (ALL OR UNSPEC) | Redacted4 | 18.3923 | 35 | A | G | 0.2627 |
| 2017 | 15-May-17 | VENTURA | STRAWBERRY (ALL OR UNSPEC) | Redacted4 | 18.3923 | 35 | A | G | 0.2627 |
| 2017 | 2-Jun-17 | VENTURA | STRAWBERRY (ALL OR UNSPEC) | Redacted4 | 18.3923 | 35 | A | G | 0.2627 |
| 2017 | 17-Nov-17 | VENTURA | STRAWBERRY (ALL OR UNSPEC) | Redacted4 | 18.1403 | 34.5 | A | G | 0.2629 |
| 2017 | 30-Nov-17 | VENTURA | STRAWBERRY (ALL OR UNSPEC) | Redacted4 | 18.1403 | 34.5 | A | G | 0.2629 |
| 2017 | 2-Jun-17 | MONTEREY | STRAWBERRY (ALL OR UNSPEC) | Redacted4 | 8.4823 | 16.13 | A | G | 0.2629 |
| 2017 | 11-May-17 | VENTURA | STRAWBERRY (ALL OR UNSPEC) | Redacted4 | 17.8884 | 34 | A | G | 0.2631 |
| 2017 | 18-May-17 | VENTURA | STRAWBERRY (ALL OR UNSPEC) | Redacted4 | 17.8884 | 34 | A | G | 0.2631 |
| 2017 | 18-Jul-17 | VENTURA | STRAWBERRY (ALL OR UNSPEC) | Redacted4 | 1.3437 | 2.5 | A | G | 0.2687 |
| 2017 | 14-Jul-17 | SANTA BARBARA | STRAWBERRY (ALL OR UNSPEC) | Redacted4 | 3.6113 | 6.18 | A | G | 0.2922 |
| 2017 | 28-Jun-17 | SANTA BARBARA | STRAWBERRY (ALL OR UNSPEC) | Redacted4 | 6.7186 | 11.47 | A | G | 0.2929 |
| 2017 | 10-Jul-17 | SANTA BARBARA | STRAWBERRY (ALL OR UNSPEC) | Redacted4 | 10.4979 | 17.91 | A | G | 0.2931 |
| 2017 | 29-Jun-17 | SANTA BARBARA | STRAWBERRY (ALL OR UNSPEC) | Redacted4 | 10.4979 | 17.91 | A | G | 0.2931 |
| 2017 | 20-Jun-17 | SANTA BARBARA | STRAWBERRY (ALL OR UNSPEC) | Redacted4 | 10.4979 | 17.91 | A | G | 0.2931 |
| 2017 | 1-Jun-17 | SANTA BARBARA | STRAWBERRY (ALL OR UNSPEC) | Redacted4 | 10.4979 | 17.91 | A | G | 0.2931 |
| 2017 | 8-Jun-17 | SANTA BARBARA | STRAWBERRY (ALL OR UNSPEC) | Redacted4 | 10.4979 | 17.91 | A | G | 0.2931 |
| 2017 | 25-May-17 | SANTA BARBARA | STRAWBERRY (ALL OR UNSPEC) | Redacted4 | 10.4979 | 17.91 | A | G | 0.2931 |
| 2017 | 30-Sep-17 | SANTA BARBARA | STRAWBERRY (ALL OR UNSPEC) | Redacted4 | 5.2909 | 9.02 | A | G | 0.2933 |
| 2017 | 1-Sep-17 | SANTA BARBARA | STRAWBERRY (ALL OR UNSPEC) | Redacted4 | 13.8572 | 23.6 | A | G | 0.2936 |
| 2017 | 26-May-17 | SANTA BARBARA | STRAWBERRY (ALL OR UNSPEC) | Redacted4 | 12.5975 | 21.45 | A | G | 0.2936 |
| 2017 | 10-Jul-17 | SANTA BARBARA | STRAWBERRY (ALL OR UNSPEC) | Redacted4 | 12.6814 | 21.59 | A | G | 0.2937 |
| 2017 | 2-Jun-17 | SANTA BARBARA | STRAWBERRY (ALL OR UNSPEC) | Redacted4 | 12.6814 | 21.59 | A | G | 0.2937 |
| 2017 | 15-Sep-17 | SANTA BARBARA | STRAWBERRY (ALL OR UNSPEC) | Redacted4 | 8.5663 | 14.58 | A | G | 0.2938 |
| 2017 | 29-Sep-17 | SANTA BARBARA | STRAWBERRY (ALL OR UNSPEC) | Redacted4 | 8.5663 | 14.58 | A | G | 0.2938 |
| 2017 | 20-Sep-17 | SANTA BARBARA | STRAWBERRY (ALL OR UNSPEC) | Redacted4 | 8.5663 | 14.58 | A | G | 0.2938 |
| 2017 | 5-Oct-17 | SANTA BARBARA | STRAWBERRY (ALL OR UNSPEC) | Redacted4 | 8.5663 | 14.58 | A | G | 0.2938 |
| 2017 | 20-Jun-17 | SANTA BARBARA | STRAWBERRY (ALL OR UNSPEC) | Redacted4 | 12.5135 | 21.29 | A | G | 0.2939 |
| 2017 | 27-Apr-17 | VENTURA | STRAWBERRY (ALL OR UNSPEC) | Redacted4 | 11.7576 | 20 | A | G | 0.2939 |
| 2017 | 12-May-17 | SANTA BARBARA | STRAWBERRY (ALL OR UNSPEC) | Redacted4 | 12.9334 | 22 | A | G | 0.2939 |
| 2017 | 5-May-17 | VENTURA | STRAWBERRY (ALL OR UNSPEC) | Redacted4 | 14.1092 | 24 | A | G | 0.2939 |
| 2017 | 20-May-17 | SANTA BARBARA | STRAWBERRY (ALL OR UNSPEC) | Redacted4 | 11.3377 | 19.28 | A | G | 0.2940 |
| 2017 | 18-May-17 | SANTA BARBARA | STRAWBERRY (ALL OR UNSPEC) | Redacted4 | 11.7576 | 19.99 | A | G | 0.2941 |
| 2017 | 22-Jun-17 | SANTA BARBARA | STRAWBERRY (ALL OR UNSPEC) | Redacted4 | 18.5603 | 31.55 | A | G | 0.2941 |
| 2017 | 31-May-17 | SANTA BARBARA | STRAWBERRY (ALL OR UNSPEC) | Redacted4 | 18.5603 | 31.55 | A | G | 0.2941 |
| 2017 | 9-May-17 | SANTA BARBARA | STRAWBERRY (ALL OR UNSPEC) | Redacted4 | 18.5603 | 31.55 | A | G | 0.2941 |
| 2017 | 24-May-17 | SANTA BARBARA | STRAWBERRY (ALL OR UNSPEC) | Redacted4 | 18.5603 | 31.55 | A | G | 0.2941 |
| 2017 | 28-Jun-17 | SANTA BARBARA | STRAWBERRY (ALL OR UNSPEC) | Redacted4 | 18.5603 | 31.55 | A | G | 0.2941 |
| 2017 | 16-Jul-17 | SANTA BARBARA | STRAWBERRY (ALL OR UNSPEC) | Redacted4 | 18.5603 | 31.55 | A | G | 0.2941 |
| 2017 | 13-Jul-17 | SANTA BARBARA | STRAWBERRY (ALL OR UNSPEC) | Redacted4 | 23.7672 | 40.37 | A | G | 0.2944 |
| 2017 | 11-Nov-17 | SANTA BARBARA | STRAWBERRY (ALL OR UNSPEC) | Redacted4 | 24.775 | 42.08 | A | G | 0.2944 |
| 2017 | 19-May-17 | SANTA BARBARA | STRAWBERRY (ALL OR UNSPEC) | Redacted4 | 12.4295 | 21.11 | A | G | 0.2944 |
| 2017 | 3-Aug-17 | SANTA BARBARA | STRAWBERRY (ALL OR UNSPEC) | Redacted4 | 23.0114 | 39.08 | A | G | 0.2944 |
| 2017 | 29-Jun-17 | SANTA BARBARA | STRAWBERRY (ALL OR UNSPEC) | Redacted4 | 5.9628 | 10.12 | A | G | 0.2946 |
| 2017 | 10-May-17 | SANTA BARBARA | STRAWBERRY (ALL OR UNSPEC) | Redacted4 | 12.3455 | 20.95 | A | G | 0.2946 |
| 2017 | 16-Sep-17 | SANTA BARBARA | STRAWBERRY (ALL OR UNSPEC) | Redacted4 | 23.4313 | 39.76 | A | G | 0.2947 |
| 2017 | 6-Oct-17 | SANTA BARBARA | STRAWBERRY (ALL OR UNSPEC) | Redacted4 | 23.4313 | 39.76 | A | G | 0.2947 |
| 2017 | 13-Oct-17 | SANTA BARBARA | STRAWBERRY (ALL OR UNSPEC) | Redacted4 | 23.4313 | 39.76 | A | G | 0.2947 |
| 2017 | 10-Nov-17 | SANTA BARBARA | STRAWBERRY (ALL OR UNSPEC) | Redacted4 | 23.4313 | 39.76 | A | G | 0.2947 |
| 2017 | 22-Sep-17 | SANTA BARBARA | STRAWBERRY (ALL OR UNSPEC) | Redacted4 | 23.4313 | 39.76 | A | G | 0.2947 |
| 2017 | 1-Nov-17 | SANTA BARBARA | STRAWBERRY (ALL OR UNSPEC) | Redacted4 | 23.4313 | 39.76 | A | G | 0.2947 |
| 2017 | 29-Sep-17 | SANTA BARBARA | STRAWBERRY (ALL OR UNSPEC) | Redacted4 | 22.1715 | 37.62 | A | G | 0.2947 |
| 2017 | 27-Nov-17 | SANTA BARBARA | STRAWBERRY (ALL OR UNSPEC) | Redacted4 | 22.1715 | 37.62 | A | G | 0.2947 |
| 2017 | 16-Nov-17 | SANTA BARBARA | STRAWBERRY (ALL OR UNSPEC) | Redacted4 | 22.1715 | 37.62 | A | G | 0.2947 |
| 2017 | 13-Sep-17 | SANTA BARBARA | STRAWBERRY (ALL OR UNSPEC) | Redacted4 | 22.1715 | 37.62 | A | G | 0.2947 |
| 2017 | 23-Sep-17 | SANTA BARBARA | STRAWBERRY (ALL OR UNSPEC) | Redacted4 | 22.1715 | 37.62 | A | G | 0.2947 |
| 2017 | 7-Oct-17 | SANTA BARBARA | STRAWBERRY (ALL OR UNSPEC) | Redacted4 | 22.1715 | 37.62 | A | G | 0.2947 |
| 2017 | 9-Jun-17 | SANTA BARBARA | STRAWBERRY (ALL OR UNSPEC) | Redacted4 | 40.3119 | 68.38 | A | G | 0.2948 |
| 2017 | 26-May-17 | SANTA BARBARA | STRAWBERRY (ALL OR UNSPEC) | Redacted4 | 12.5975 | 21.36 | A | G | 0.2949 |
| 2017 | 5-Aug-17 | SANTA BARBARA | STRAWBERRY (ALL OR UNSPEC) | Redacted4 | 12.5975 | 21.36 | A | G | 0.2949 |
| 2017 | 31-May-17 | SANTA BARBARA | STRAWBERRY (ALL OR UNSPEC) | Redacted4 | 12.5975 | 21.36 | A | G | 0.2949 |
| 2017 | 21-Jun-17 | SANTA BARBARA | STRAWBERRY (ALL OR UNSPEC) | Redacted4 | 12.5975 | 21.36 | A | G | 0.2949 |
| 2017 | 12-Jul-17 | SANTA BARBARA | STRAWBERRY (ALL OR UNSPEC) | Redacted4 | 12.5975 | 21.36 | A | G | 0.2949 |
| 2017 | 12-Jun-17 | SANTA BARBARA | STRAWBERRY (ALL OR UNSPEC) | Redacted4 | 12.5975 | 21.36 | A | G | 0.2949 |
| 2017 | 26-Apr-17 | VENTURA | STRAWBERRY (ALL OR UNSPEC) | Redacted4 | 25.9508 | 44 | A | G | 0.2949 |
| 2017 | 30-May-17 | SANTA BARBARA | STRAWBERRY (ALL OR UNSPEC) | Redacted4 | 24.691 | 41.86 | A | G | 0.2949 |
| 2017 | 2-May-17 | SANTA BARBARA | STRAWBERRY (ALL OR UNSPEC) | Redacted4 | 24.691 | 41.86 | A | G | 0.2949 |
| 2017 | 6-Jun-17 | SANTA BARBARA | STRAWBERRY (ALL OR UNSPEC) | Redacted4 | 24.691 | 41.86 | A | G | 0.2949 |
| 2017 | 24-Jun-17 | SANTA BARBARA | STRAWBERRY (ALL OR UNSPEC) | Redacted4 | 24.691 | 41.86 | A | G | 0.2949 |
| 2017 | 6-Jul-17 | SANTA BARBARA | STRAWBERRY (ALL OR UNSPEC) | Redacted4 | 24.691 | 41.86 | A | G | 0.2949 |
| 2017 | 26-May-17 | SANTA BARBARA | STRAWBERRY (ALL OR UNSPEC) | Redacted4 | 24.691 | 41.86 | A | G | 0.2949 |
| 2017 | 3-Aug-17 | SANTA BARBARA | STRAWBERRY (ALL OR UNSPEC) | Redacted4 | 23.5992 | 40 | A | G | 0.2950 |
| 2017 | 4-May-17 | VENTURA | STRAWBERRY (ALL OR UNSPEC) | Redacted4 | 23.5992 | 40 | A | G | 0.2950 |
| 2017 | 31-Aug-17 | SANTA BARBARA | STRAWBERRY (ALL OR UNSPEC) | Redacted4 | 18.1403 | 30.74 | A | G | 0.2951 |
| 2017 | 26-Aug-17 | SANTA BARBARA | STRAWBERRY (ALL OR UNSPEC) | Redacted4 | 18.1403 | 30.74 | A | G | 0.2951 |
| 2017 | 28-Sep-17 | SANTA BARBARA | STRAWBERRY (ALL OR UNSPEC) | Redacted4 | 18.1403 | 30.74 | A | G | 0.2951 |
| 2017 | 5-May-17 | VENTURA | STRAWBERRY (ALL OR UNSPEC) | Redacted4 | 20.6598 | 35 | A | G | 0.2951 |
| 2017 | 9-May-17 | SANTA BARBARA | STRAWBERRY (ALL OR UNSPEC) | Redacted4 | 43.3353 | 73.41 | A | G | 0.2952 |
| 2017 | 2-May-17 | VENTURA | STRAWBERRY (ALL OR UNSPEC) | Redacted4 | 20.072 | 34 | A | G | 0.2952 |
| 2017 | 22-Nov-17 | SANTA BARBARA | STRAWBERRY (ALL OR UNSPEC) | Redacted4 | 32.0815 | 54.34 | A | G | 0.2952 |
| 2017 | 15-Nov-17 | SANTA BARBARA | STRAWBERRY (ALL OR UNSPEC) | Redacted4 | 32.0815 | 54.34 | A | G | 0.2952 |
| 2017 | 17-Aug-17 | SANTA BARBARA | STRAWBERRY (ALL OR UNSPEC) | Redacted4 | 32.0815 | 54.34 | A | G | 0.2952 |
| 2017 | 14-Aug-17 | SANTA BARBARA | STRAWBERRY (ALL OR UNSPEC) | Redacted4 | 32.0815 | 54.34 | A | G | 0.2952 |
| 2017 | 14-Oct-17 | SANTA BARBARA | STRAWBERRY (ALL OR UNSPEC) | Redacted4 | 30.8218 | 52.2 | A | G | 0.2952 |
| 2017 | 2-Nov-17 | SANTA BARBARA | STRAWBERRY (ALL OR UNSPEC) | Redacted4 | 30.8218 | 52.2 | A | G | 0.2952 |
| 2017 | 7-Sep-17 | SANTA BARBARA | STRAWBERRY (ALL OR UNSPEC) | Redacted4 | 30.8218 | 52.2 | A | G | 0.2952 |
| 2017 | 12-Jul-17 | SANTA BARBARA | STRAWBERRY (ALL OR UNSPEC) | Redacted4 | 18.9802 | 32.14 | A | G | 0.2953 |
| 2017 | 23-Jun-17 | SANTA BARBARA | STRAWBERRY (ALL OR UNSPEC) | Redacted4 | 21.7516 | 36.83 | A | G | 0.2953 |
| 2017 | 10-May-17 | SANTA BARBARA | STRAWBERRY (ALL OR UNSPEC) | Redacted4 | 21.7516 | 36.83 | A | G | 0.2953 |
| 2017 | 25-May-17 | SANTA BARBARA | STRAWBERRY (ALL OR UNSPEC) | Redacted4 | 21.7516 | 36.83 | A | G | 0.2953 |
| 2017 | 1-Jun-17 | SANTA BARBARA | STRAWBERRY (ALL OR UNSPEC) | Redacted4 | 21.7516 | 36.83 | A | G | 0.2953 |
| 2017 | 17-May-17 | SANTA BARBARA | STRAWBERRY (ALL OR UNSPEC) | Redacted4 | 21.7516 | 36.83 | A | G | 0.2953 |
| 2017 | 30-Jun-17 | SANTA BARBARA | STRAWBERRY (ALL OR UNSPEC) | Redacted4 | 21.7516 | 36.83 | A | G | 0.2953 |
| 2017 | 25-Aug-17 | SANTA BARBARA | STRAWBERRY (ALL OR UNSPEC) | Redacted4 | 13.9412 | 23.6 | A | G | 0.2954 |
| 2017 | 8-Sep-17 | SANTA BARBARA | STRAWBERRY (ALL OR UNSPEC) | Redacted4 | 23.5153 | 39.76 | A | G | 0.2957 |
| 2017 | 24-Nov-17 | VENTURA | STRAWBERRY (ALL OR UNSPEC) | Redacted4 | 20.4079 | 34.5 | A | G | 0.2958 |
| 2017 | 3-Sep-17 | SANTA BARBARA | STRAWBERRY (ALL OR UNSPEC) | Redacted4 | 22.2555 | 37.62 | A | G | 0.2958 |
| 2017 | 11-Aug-17 | SANTA BARBARA | STRAWBERRY (ALL OR UNSPEC) | Redacted4 | 22.2555 | 37.62 | A | G | 0.2958 |
| 2017 | 24-Aug-17 | SANTA BARBARA | STRAWBERRY (ALL OR UNSPEC) | Redacted4 | 22.2555 | 37.62 | A | G | 0.2958 |
| 2017 | 18-Aug-17 | SANTA BARBARA | STRAWBERRY (ALL OR UNSPEC) | Redacted4 | 22.2555 | 37.62 | A | G | 0.2958 |
| 2017 | 18-Jul-17 | MONTEREY | STRAWBERRY (ALL OR UNSPEC) | Redacted4 | 31.4097 | 53 | A | G | 0.2963 |
| 2017 | 9-Jun-17 | MONTEREY | STRAWBERRY (ALL OR UNSPEC) | Redacted4 | 44.847 | 72 | A | G | 0.3114 |
| 2017 | 10-Jun-17 | MONTEREY | STRAWBERRY (ALL OR UNSPEC) | Redacted4 | 76.0047 | 122 | A | G | 0.3115 |
| 2017 | 12-May-17 | MONTEREY | STRAWBERRY (ALL OR UNSPEC) | Redacted4 | 75.4168 | 121 | A | G | 0.3116 |
| 2017 | 13-May-17 | MONTEREY | STRAWBERRY (ALL OR UNSPEC) | Redacted4 | 43.6712 | 70 | A | G | 0.3119 |
| 2017 | 12-May-17 | MONTEREY | STRAWBERRY (ALL OR UNSPEC) | Redacted4 | 10.078 | 16.13 | A | G | 0.3124 |
| 2017 | 9-Jun-17 | MONTEREY | STRAWBERRY (ALL OR UNSPEC) | Redacted4 | 10.078 | 16.13 | A | G | 0.3124 |
| 2017 | 30-May-17 | FRESNO | TANGERINE (MANDARIN, SATSUMA, MURCOTT, ETC.) | Redacted4 | 2.7819 | 8.5 | A | G | 0.1636 |
| 2017 | 30-May-17 | FRESNO | TANGERINE (MANDARIN, SATSUMA, MURCOTT, ETC.) | Redacted4 | 2.7819 | 8.5 | A | G | 0.1636 |
| 2017 | 7-Jul-17 | FRESNO | TANGERINE (MANDARIN, SATSUMA, MURCOTT, ETC.) | Redacted4 | 4.913 | 15 | A | G | 0.1638 |
| 2017 | 1-Jun-17 | FRESNO | TANGERINE (MANDARIN, SATSUMA, MURCOTT, ETC.) | Redacted4 | 4.913 | 15 | A | G | 0.1638 |
| 2017 | 7-Jul-17 | FRESNO | TANGERINE (MANDARIN, SATSUMA, MURCOTT, ETC.) | Redacted4 | 2.8449 | 8.68 | A | G | 0.1639 |
| 2017 | 1-Jun-17 | FRESNO | TANGERINE (MANDARIN, SATSUMA, MURCOTT, ETC.) | Redacted4 | 18.6967 | 57 | A | G | 0.1640 |
| 2017 | 7-Jul-17 | FRESNO | TANGERINE (MANDARIN, SATSUMA, MURCOTT, ETC.) | Redacted4 | 18.6967 | 57 | A | G | 0.1640 |
| 2017 | 1-Jun-17 | TULARE | TANGERINE (MANDARIN, SATSUMA, MURCOTT, ETC.) | Redacted4 | 15.7783 | 48.1 | A | G | 0.1640 |
| 2017 | 8-Jul-17 | TULARE | TANGERINE (MANDARIN, SATSUMA, MURCOTT, ETC.) | Redacted4 | 15.7783 | 48.1 | A | G | 0.1640 |
| 2017 | 8-Jul-17 | TULARE | TANGERINE (MANDARIN, SATSUMA, MURCOTT, ETC.) | Redacted4 | 20.6703 | 63.01 | A | G | 0.1640 |
| 2017 | 30-May-17 | FRESNO | TANGERINE (MANDARIN, SATSUMA, MURCOTT, ETC.) | Redacted4 | 1.3122 | 4 | A | G | 0.1640 |
| 2017 | 30-May-17 | FRESNO | TANGERINE (MANDARIN, SATSUMA, MURCOTT, ETC.) | Redacted4 | 1.3122 | 4 | A | G | 0.1640 |
| 2017 | 31-May-17 | TULARE | TANGERINE (MANDARIN, SATSUMA, MURCOTT, ETC.) | Redacted4 | 13.1224 | 40 | A | G | 0.1640 |
| 2017 | 1-Jun-17 | FRESNO | TANGERINE (MANDARIN, SATSUMA, MURCOTT, ETC.) | Redacted4 | 3.2858 | 10 | A | G | 0.1643 |
| 2017 | 7-Jul-17 | FRESNO | TANGERINE (MANDARIN, SATSUMA, MURCOTT, ETC.) | Redacted4 | 3.2858 | 10 | A | G | 0.1643 |
| 2017 | 31-May-17 | SAN DIEGO | TOMATO | Redacted4 | 5.4851 | 41.8 | A | G | 0.0656 |
| 2017 | 5-Sep-17 | SAN BENITO | TOMATO | Redacted4 | 1.2597 | 6.5 | A | G | 0.0969 |
| 2017 | 11-Aug-17 | SAN BENITO | TOMATO | Redacted4 | 1.2597 | 6.5 | A | G | 0.0969 |
| 2017 | 22-Jun-17 | FRESNO | TOMATO | Redacted4 | 7.8104 | 40 | A | G | 0.0976 |
| 2017 | 22-Jun-17 | FRESNO | TOMATO | Redacted4 | 7.8104 | 40 | A | G | 0.0976 |
| 2017 | 22-Jun-17 | FRESNO | TOMATO | Redacted4 | 7.8104 | 40 | A | G | 0.0976 |
| 2017 | 21-Jul-17 | SANTA CLARA | TOMATO | Redacted4 | 0.5879 | 3 | A | G | 0.0980 |
| 2017 | 20-Jul-17 | SANTA CLARA | TOMATO | Redacted4 | 0.5039 | 2.5 | A | G | 0.1008 |
| 2017 | 26-Jul-17 | SAN BENITO | TOMATO | Redacted4 | 1.0918 | 5.35 | A | G | 0.1020 |
| 2017 | 28-Jun-17 | SAN BENITO | TOMATO | Redacted4 | 1.0918 | 5.35 | A | G | 0.1020 |
| 2017 | 16-Jun-17 | SAN BENITO | TOMATO | Redacted4 | 1.0918 | 5.35 | A | G | 0.1020 |
| 2017 | 19-Jul-17 | FRESNO | TOMATO | Redacted4 | 12.5975 | 50 | A | A | 0.1260 |
| 2017 | 19-Jul-17 | FRESNO | TOMATO | Redacted4 | 12.5975 | 50 | A | A | 0.1260 |
| 2017 | 14-Sep-17 | SAN BENITO | TOMATO | Redacted4 | 9.1542 | 35 | A | G | 0.1308 |
| 2017 | 12-Aug-17 | FRESNO | TOMATO | Redacted4 | 13.3533 | 51 | A | A | 0.1309 |
| 2017 | 12-Aug-17 | FRESNO | TOMATO | Redacted4 | 13.3533 | 51 | A | A | 0.1309 |
| 2017 | 12-Aug-17 | FRESNO | TOMATO | Redacted4 | 13.3533 | 51 | A | A | 0.1309 |
| 2017 | 2-Jul-17 | FRESNO | TOMATO | Redacted4 | 13.1014 | 50 | A | A | 0.1310 |
| 2017 | 4-Aug-17 | FRESNO | TOMATO | Redacted4 | 13.1014 | 50 | A | A | 0.1310 |
| 2017 | 2-Jul-17 | FRESNO | TOMATO | Redacted4 | 13.1014 | 50 | A | A | 0.1310 |
| 2017 | 2-Jul-17 | FRESNO | TOMATO | Redacted4 | 13.1014 | 50 | A | A | 0.1310 |
| 2017 | 4-Aug-17 | FRESNO | TOMATO | Redacted4 | 13.1014 | 50 | A | A | 0.1310 |
| 2017 | 4-Aug-17 | FRESNO | TOMATO | Redacted4 | 13.1014 | 50 | A | A | 0.1310 |
| 2017 | 2-Jul-17 | FRESNO | TOMATO | Redacted4 | 12.6814 | 48.34 | A | A | 0.1312 |
| 2017 | 2-Jul-17 | FRESNO | TOMATO | Redacted4 | 12.6814 | 48.33 | A | A | 0.1312 |
| 2017 | 2-Jul-17 | FRESNO | TOMATO | Redacted4 | 12.6814 | 48.33 | A | A | 0.1312 |
| 2017 | 15-Sep-17 | SAN BENITO | TOMATO | Redacted4 | 20.9958 | 80 | A | G | 0.1312 |
| 2017 | 1-Sep-17 | FRESNO | TOMATO | Redacted4 | 8.1464 | 25 | A | A | 0.1629 |
| 2017 | 12-Jul-17 | FRESNO | TOMATO | Redacted4 | 15.7888 | 48.34 | A | A | 0.1633 |
| 2017 | 30-Jul-17 | FRESNO | TOMATO | Redacted4 | 15.7888 | 48.34 | A | A | 0.1633 |
| 2017 | 26-Jul-17 | FRESNO | TOMATO | Redacted4 | 15.7888 | 48.34 | A | A | 0.1633 |
| 2017 | 30-Jul-17 | FRESNO | TOMATO | Redacted4 | 15.7888 | 48.33 | A | A | 0.1633 |
| 2017 | 26-Jul-17 | FRESNO | TOMATO | Redacted4 | 15.7888 | 48.33 | A | A | 0.1633 |
| 2017 | 30-Jul-17 | FRESNO | TOMATO | Redacted4 | 15.7888 | 48.33 | A | A | 0.1633 |
| 2017 | 12-Jul-17 | FRESNO | TOMATO | Redacted4 | 15.7888 | 48.33 | A | A | 0.1633 |
| 2017 | 12-Jul-17 | FRESNO | TOMATO | Redacted4 | 15.7888 | 48.33 | A | A | 0.1633 |
| 2017 | 26-Jul-17 | FRESNO | TOMATO | Redacted4 | 15.7888 | 48.33 | A | A | 0.1633 |
| 2017 | 25-Jun-17 | FRESNO | TOMATO | Redacted4 | 16.8806 | 51.67 | A | A | 0.1634 |
| 2017 | 25-Jun-17 | FRESNO | TOMATO | Redacted4 | 16.8806 | 51.67 | A | A | 0.1634 |
| 2017 | 25-Jun-17 | FRESNO | TOMATO | Redacted4 | 16.8806 | 51.66 | A | A | 0.1634 |
| 2017 | 12-Aug-17 | FRESNO | TOMATO | Redacted4 | 16.1247 | 49.34 | A | A | 0.1634 |
| 2017 | 30-Aug-17 | FRESNO | TOMATO | Redacted4 | 16.1247 | 49.34 | A | A | 0.1634 |
| 2017 | 26-Jul-17 | FRESNO | TOMATO | Redacted4 | 16.1247 | 49.34 | A | A | 0.1634 |
| 2017 | 2-Aug-17 | FRESNO | TOMATO | Redacted4 | 16.1247 | 49.34 | A | A | 0.1634 |
| 2017 | 12-Jul-17 | FRESNO | TOMATO | Redacted4 | 16.1247 | 49.34 | A | A | 0.1634 |
| 2017 | 12-Jul-17 | FRESNO | TOMATO | Redacted4 | 16.1247 | 49.33 | A | A | 0.1634 |
| 2017 | 12-Aug-17 | FRESNO | TOMATO | Redacted4 | 16.1247 | 49.33 | A | A | 0.1634 |
| 2017 | 12-Aug-17 | FRESNO | TOMATO | Redacted4 | 16.1247 | 49.33 | A | A | 0.1634 |
| 2017 | 30-Aug-17 | FRESNO | TOMATO | Redacted4 | 16.1247 | 49.33 | A | A | 0.1634 |
| 2017 | 30-Aug-17 | FRESNO | TOMATO | Redacted4 | 16.1247 | 49.33 | A | A | 0.1634 |
| 2017 | 2-Aug-17 | FRESNO | TOMATO | Redacted4 | 16.1247 | 49.33 | A | A | 0.1634 |
| 2017 | 2-Aug-17 | FRESNO | TOMATO | Redacted4 | 16.1247 | 49.33 | A | A | 0.1634 |
| 2017 | 26-Jul-17 | FRESNO | TOMATO | Redacted4 | 16.1247 | 49.33 | A | A | 0.1634 |
| 2017 | 26-Jul-17 | FRESNO | TOMATO | Redacted4 | 16.1247 | 49.33 | A | A | 0.1634 |
| 2017 | 12-Jul-17 | FRESNO | TOMATO | Redacted4 | 16.1247 | 49.33 | A | A | 0.1634 |
| 2017 | 25-Jun-17 | FRESNO | TOMATO | Redacted4 | 19.3161 | 59 | A | A | 0.1637 |
| 2017 | 25-Jun-17 | FRESNO | TOMATO | Redacted4 | 19.3161 | 59 | A | A | 0.1637 |
| 2017 | 13-Sep-17 | FRESNO | TOMATO | Redacted4 | 17.4685 | 53.34 | A | A | 0.1637 |
| 2017 | 20-Sep-17 | FRESNO | TOMATO | Redacted4 | 17.4685 | 53.34 | A | A | 0.1637 |
| 2017 | 28-Sep-17 | FRESNO | TOMATO | Redacted4 | 17.4685 | 53.34 | A | A | 0.1637 |
| 2017 | 28-Aug-17 | FRESNO | TOMATO | Redacted4 | 17.4685 | 53.34 | A | A | 0.1637 |
| 2017 | 6-Sep-17 | FRESNO | TOMATO | Redacted4 | 17.4685 | 53.34 | A | A | 0.1637 |
| 2017 | 5-Sep-17 | FRESNO | TOMATO | Redacted4 | 17.4685 | 53.34 | A | A | 0.1637 |
| 2017 | 13-Sep-17 | FRESNO | TOMATO | Redacted4 | 17.4685 | 53.34 | A | A | 0.1637 |
| 2017 | 25-Aug-17 | FRESNO | TOMATO | Redacted4 | 26.2027 | 80 | A | A | 0.1638 |
| 2017 | 1-Sep-17 | FRESNO | TOMATO | Redacted4 | 26.2027 | 80 | A | A | 0.1638 |
| 2017 | 25-Aug-17 | FRESNO | TOMATO | Redacted4 | 16.3767 | 50 | A | A | 0.1638 |
| 2017 | 1-Sep-17 | FRESNO | TOMATO | Redacted4 | 16.3767 | 50 | A | A | 0.1638 |
| 2017 | 11-Jul-17 | FRESNO | TOMATO | Redacted4 | 16.3767 | 50 | A | A | 0.1638 |
| 2017 | 11-Jul-17 | FRESNO | TOMATO | Redacted4 | 16.3767 | 50 | A | A | 0.1638 |
| 2017 | 12-Jul-17 | FRESNO | TOMATO | Redacted4 | 16.3767 | 50 | A | A | 0.1638 |
| 2017 | 12-Jul-17 | FRESNO | TOMATO | Redacted4 | 16.3767 | 50 | A | A | 0.1638 |
| 2017 | 12-Aug-17 | FRESNO | TOMATO | Redacted4 | 16.3767 | 50 | A | A | 0.1638 |
| 2017 | 12-Aug-17 | FRESNO | TOMATO | Redacted4 | 16.3767 | 50 | A | A | 0.1638 |
| 2017 | 12-Aug-17 | FRESNO | TOMATO | Redacted4 | 16.3767 | 50 | A | A | 0.1638 |
| 2017 | 25-Aug-17 | FRESNO | TOMATO | Redacted4 | 16.3767 | 50 | A | A | 0.1638 |
| 2017 | 1-Aug-17 | FRESNO | TOMATO | Redacted4 | 16.3767 | 50 | A | A | 0.1638 |
| 2017 | 12-Jul-17 | FRESNO | TOMATO | Redacted4 | 16.3767 | 50 | A | A | 0.1638 |
| 2017 | 11-Jul-17 | FRESNO | TOMATO | Redacted4 | 16.3767 | 50 | A | A | 0.1638 |
| 2017 | 1-Aug-17 | FRESNO | TOMATO | Redacted4 | 16.3767 | 50 | A | A | 0.1638 |
| 2017 | 25-Aug-17 | FRESNO | TOMATO | Redacted4 | 16.3767 | 50 | A | A | 0.1638 |
| 2017 | 30-Jul-17 | FRESNO | TOMATO | Redacted4 | 16.3767 | 50 | A | A | 0.1638 |
| 2017 | 1-Aug-17 | FRESNO | TOMATO | Redacted4 | 16.3767 | 50 | A | A | 0.1638 |
| 2017 | 5-Jul-17 | FRESNO | TOMATO | Redacted4 | 6.5507 | 20 | A | G | 0.1638 |
| 2017 | 6-Jul-17 | FRESNO | TOMATO | Redacted4 | 13.1014 | 40 | A | A | 0.1638 |
| 2017 | 6-Jul-17 | FRESNO | TOMATO | Redacted4 | 13.1014 | 40 | A | A | 0.1638 |
| 2017 | 28-Aug-17 | FRESNO | TOMATO | Redacted4 | 17.4685 | 53.33 | A | A | 0.1638 |
| 2017 | 20-Sep-17 | FRESNO | TOMATO | Redacted4 | 17.4685 | 53.33 | A | A | 0.1638 |
| 2017 | 13-Sep-17 | FRESNO | TOMATO | Redacted4 | 17.4685 | 53.33 | A | A | 0.1638 |
| 2017 | 28-Sep-17 | FRESNO | TOMATO | Redacted4 | 17.4685 | 53.33 | A | A | 0.1638 |
| 2017 | 13-Sep-17 | FRESNO | TOMATO | Redacted4 | 17.4685 | 53.33 | A | A | 0.1638 |
| 2017 | 13-Sep-17 | FRESNO | TOMATO | Redacted4 | 17.4685 | 53.33 | A | A | 0.1638 |
| 2017 | 6-Sep-17 | FRESNO | TOMATO | Redacted4 | 17.4685 | 53.33 | A | A | 0.1638 |
| 2017 | 28-Sep-17 | FRESNO | TOMATO | Redacted4 | 17.4685 | 53.33 | A | A | 0.1638 |
| 2017 | 5-Sep-17 | FRESNO | TOMATO | Redacted4 | 17.4685 | 53.33 | A | A | 0.1638 |
| 2017 | 6-Oct-17 | FRESNO | TOMATO | Redacted4 | 17.4685 | 53.33 | A | A | 0.1638 |
| 2017 | 6-Oct-17 | FRESNO | TOMATO | Redacted4 | 17.4685 | 53.33 | A | A | 0.1638 |
| 2017 | 28-Aug-17 | FRESNO | TOMATO | Redacted4 | 17.4685 | 53.33 | A | A | 0.1638 |
| 2017 | 6-Sep-17 | FRESNO | TOMATO | Redacted4 | 17.4685 | 53.33 | A | A | 0.1638 |
| 2017 | 5-Sep-17 | FRESNO | TOMATO | Redacted4 | 17.4685 | 53.33 | A | A | 0.1638 |
| 2017 | 13-Sep-17 | FRESNO | TOMATO | Redacted4 | 17.4685 | 53.33 | A | A | 0.1638 |
| 2017 | 20-Sep-17 | FRESNO | TOMATO | Redacted4 | 17.4685 | 53.33 | A | A | 0.1638 |
| 2017 | 28-Sep-17 | FRESNO | TOMATO | Redacted4 | 17.4685 | 53.33 | A | A | 0.1638 |
| 2017 | 5-Sep-17 | FRESNO | TOMATO | Redacted4 | 16.7126 | 51 | A | A | 0.1638 |
| 2017 | 22-Sep-17 | FRESNO | TOMATO | Redacted4 | 16.7126 | 51 | A | A | 0.1638 |
| 2017 | 5-Sep-17 | FRESNO | TOMATO | Redacted4 | 16.7126 | 51 | A | A | 0.1638 |
| 2017 | 28-Aug-17 | FRESNO | TOMATO | Redacted4 | 16.7126 | 51 | A | A | 0.1638 |
| 2017 | 5-Sep-17 | FRESNO | TOMATO | Redacted4 | 16.7126 | 51 | A | A | 0.1638 |
| 2017 | 20-Aug-17 | FRESNO | TOMATO | Redacted4 | 16.7126 | 51 | A | A | 0.1638 |
| 2017 | 28-Aug-17 | FRESNO | TOMATO | Redacted4 | 16.7126 | 51 | A | A | 0.1638 |
| 2017 | 28-Aug-17 | FRESNO | TOMATO | Redacted4 | 16.7126 | 51 | A | A | 0.1638 |
| 2017 | 20-Aug-17 | FRESNO | TOMATO | Redacted4 | 16.7126 | 51 | A | A | 0.1638 |
| 2017 | 12-Sep-17 | FRESNO | TOMATO | Redacted4 | 16.7126 | 51 | A | A | 0.1638 |
| 2017 | 20-Aug-17 | FRESNO | TOMATO | Redacted4 | 16.7126 | 51 | A | A | 0.1638 |
| 2017 | 6-Sep-17 | FRESNO | TOMATO | Redacted4 | 26.2867 | 80 | A | A | 0.1643 |
| 2017 | 12-Aug-17 | FRESNO | TOMATO | Redacted4 | 26.2867 | 80 | A | A | 0.1643 |
| 2017 | 1-Aug-17 | FRESNO | TOMATO | Redacted4 | 26.2867 | 80 | A | A | 0.1643 |
| 2017 | 11-Aug-17 | SANTA CLARA | TOMATO | Redacted4 | 5.7948 | 14.8 | A | G | 0.1958 |
| 2017 | 11-Aug-17 | SANTA CLARA | TOMATO | Redacted4 | 5.7948 | 14.8 | A | G | 0.1958 |
| 2017 | 17-Jul-17 | SANTA CLARA | TOMATO | Redacted4 | 4.7031 | 12 | A | G | 0.1960 |
| 2017 | 21-Aug-17 | FRESNO | TOMATO | Redacted4 | 19.652 | 50 | A | A | 0.1965 |
| 2017 | 20-Aug-17 | FRESNO | TOMATO | Redacted4 | 19.652 | 50 | A | A | 0.1965 |
| 2017 | 20-Aug-17 | FRESNO | TOMATO | Redacted4 | 19.652 | 50 | A | A | 0.1965 |
| 2017 | 21-Aug-17 | FRESNO | TOMATO | Redacted4 | 19.652 | 50 | A | A | 0.1965 |
| 2017 | 21-Aug-17 | FRESNO | TOMATO | Redacted4 | 19.652 | 50 | A | A | 0.1965 |
| 2017 | 20-Aug-17 | FRESNO | TOMATO | Redacted4 | 19.652 | 50 | A | A | 0.1965 |
| 2017 | 18-Aug-17 | SAN BENITO | TOMATO | Redacted4 | 19.652 | 50 | A | G | 0.1965 |
| 2017 | 26-Aug-17 | SANTA CLARA | TOMATO | Redacted4 | 9.826 | 25 | A | G | 0.1965 |
| 2017 | 15-Aug-17 | SANTA CLARA | TOMATO | Redacted4 | 9.826 | 25 | A | G | 0.1965 |
| 2017 | 11-Aug-17 | SANTA CLARA | TOMATO | Redacted4 | 8.7342 | 22.2 | A | G | 0.1967 |
| 2017 | 1-Sep-17 | SAN BENITO | TOMATO | Redacted4 | 27.5464 | 70 | A | G | 0.1968 |
| 2017 | 21-Aug-17 | FRESNO | TOMATO | Redacted4 | 31.4936 | 80 | A | A | 0.1968 |
| 2017 | 20-Aug-17 | FRESNO | TOMATO | Redacted4 | 31.4936 | 80 | A | A | 0.1968 |
| 2017 | 5-Sep-17 | SAN BENITO | TOMATO | Redacted4 | 31.4936 | 80 | A | G | 0.1968 |
| 2017 | 29-Aug-17 | SAN BENITO | TOMATO | Redacted4 | 31.4936 | 80 | A | G | 0.1968 |
| 2017 | 26-Aug-17 | SANTA CLARA | TOMATO | Redacted4 | 5.7108 | 14.5 | A | G | 0.1969 |
| 2017 | 15-Aug-17 | SANTA CLARA | TOMATO | Redacted4 | 5.7108 | 14.5 | A | G | 0.1969 |
| 2017 | 24-Aug-17 | SAN BENITO | TOMATO | Redacted4 | 21.6676 | 55 | A | G | 0.1970 |
| 2017 | 24-Aug-17 | SAN BENITO | TOMATO | Redacted4 | 10.2459 | 26 | A | G | 0.1970 |
| 2017 | 1-Sep-17 | SAN BENITO | TOMATO | Redacted4 | 10.2459 | 26 | A | G | 0.1970 |
| 2017 | 17-Jul-17 | SANTA CLARA | TOMATO | Redacted4 | 10.2459 | 26 | A | G | 0.1970 |
| 2017 | 2-Sep-17 | SAN BENITO | TOMATO | Redacted4 | 29.562 | 75 | A | G | 0.1971 |
| 2017 | 23-Aug-17 | SAN BENITO | TOMATO | Redacted4 | 7.8944 | 20 | A | G | 0.1974 |
| 2017 | 30-Aug-17 | SAN BENITO | TOMATO | Redacted4 | 7.8944 | 20 | A | G | 0.1974 |
| 2017 | 5-Sep-17 | SAN BENITO | TOMATO | Redacted4 | 15.7888 | 40 | A | G | 0.1974 |
| 2017 | 26-Aug-17 | SANTA CLARA | TOMATO | Redacted4 | 11.8416 | 30 | A | G | 0.1974 |
| 2017 | 15-Aug-17 | SANTA CLARA | TOMATO | Redacted4 | 11.8416 | 30 | A | G | 0.1974 |
| 2017 | 11-Aug-17 | SANTA CLARA | TOMATO | Redacted4 | 8.8182 | 22.3 | A | G | 0.1977 |
| 2017 | 15-Aug-17 | SANTA CLARA | TOMATO | Redacted4 | 5.5429 | 14 | A | G | 0.1980 |
| 2017 | 26-Aug-17 | SANTA CLARA | TOMATO | Redacted4 | 5.5429 | 14 | A | G | 0.1980 |
| 2017 | 11-Aug-17 | SANTA CLARA | TOMATO | Redacted4 | 2.9394 | 7.4 | A | G | 0.1986 |
| 2017 | 11-Aug-17 | SANTA CLARA | TOMATO | Redacted4 | 2.9394 | 7.4 | A | G | 0.1986 |
| 2017 | 22-Jul-17 | SANTA CLARA | TOMATO | Redacted4 | 69.286 | 33 | A | G |  |
| 2017 | 8-Jul-17 | KINGS | TOMATOES, FOR PROCESSING/CANNING | Redacted4 | 0.4186 | 152 | A | A | 0.0014 |
| 2017 | 16-Jul-17 | KINGS | TOMATOES, FOR PROCESSING/CANNING | Redacted4 | 0.4186 | 152 | A | A | 0.0014 |
| 2017 | 22-Jun-17 | KINGS | TOMATOES, FOR PROCESSING/CANNING | Redacted4 | 0.4206 | 149 | A | A | 0.0014 |
| 2017 | 12-Sep-17 | FRESNO | TOMATOES, FOR PROCESSING/CANNING | Redacted4 | 6.3486 | 63 | A | G | 0.0504 |
| 2017 | 12-Sep-17 | FRESNO | TOMATOES, FOR PROCESSING/CANNING | Redacted4 | 7.3564 | 73 | A | G | 0.0504 |
| 2017 | 12-Sep-17 | FRESNO | TOMATOES, FOR PROCESSING/CANNING | Redacted4 | 5.2404 | 52 | A | G | 0.0504 |
| 2017 | 12-Sep-17 | FRESNO | TOMATOES, FOR PROCESSING/CANNING | Redacted4 | 6.2482 | 62 | A | G | 0.0504 |
| 2017 | 8-Jul-17 | FRESNO | TOMATOES, FOR PROCESSING/CANNING | Redacted4 | 5.0521 | 44 | A | G | 0.0574 |
| 2017 | 15-Jul-17 | FRESNO | TOMATOES, FOR PROCESSING/CANNING | Redacted4 | 6.6924 | 58 | A | G | 0.0577 |
| 2017 | 7-Jul-17 | FRESNO | TOMATOES, FOR PROCESSING/CANNING | Redacted4 | 11.5601 | 100 | A | G | 0.0578 |
| 2017 | 7-Jul-17 | FRESNO | TOMATOES, FOR PROCESSING/CANNING | Redacted4 | 12.7162 | 110 | A | G | 0.0578 |
| 2017 | 16-Jul-17 | FRESNO | TOMATOES, FOR PROCESSING/CANNING | Redacted4 | 9.8418 | 85 | A | G | 0.0579 |
| 2017 | 17-Jul-17 | FRESNO | TOMATOES, FOR PROCESSING/CANNING | Redacted4 | 8.7264 | 75 | A | G | 0.0582 |
| 2017 | 6-Aug-17 | FRESNO | TOMATOES, FOR PROCESSING/CANNING | Redacted4 | 7.2173 | 62 | A | G | 0.0582 |
| 2017 | 5-Aug-17 | FRESNO | TOMATOES, FOR PROCESSING/CANNING | Redacted4 | 8.5144 | 73 | A | G | 0.0583 |
| 2017 | 5-Aug-17 | FRESNO | TOMATOES, FOR PROCESSING/CANNING | Redacted4 | 8.5144 | 73 | A | G | 0.0583 |
| 2017 | 5-Aug-17 | FRESNO | TOMATOES, FOR PROCESSING/CANNING | Redacted4 | 8.1647 | 70 | A | G | 0.0583 |
| 2017 | 7-Aug-17 | FRESNO | TOMATOES, FOR PROCESSING/CANNING | Redacted4 | 6.1347 | 52 | A | G | 0.0590 |
| 2017 | 7-Aug-17 | FRESNO | TOMATOES, FOR PROCESSING/CANNING | Redacted4 | 7.3151 | 62 | A | G | 0.0590 |
| 2017 | 31-May-17 | KERN | TOMATOES, FOR PROCESSING/CANNING | Redacted4 | 8.2303 | 45 | A | A | 0.0914 |
| 2017 | 19-Jul-17 | FRESNO | TOMATOES, FOR PROCESSING/CANNING | Redacted4 | 19.0274 | 100 | A | G | 0.0951 |
| 2017 | 19-Jul-17 | FRESNO | TOMATOES, FOR PROCESSING/CANNING | Redacted4 | 20.9302 | 110 | A | G | 0.0951 |
| 2017 | 17-Jul-17 | FRESNO | TOMATOES, FOR PROCESSING/CANNING | Redacted4 | 8.3983 | 44 | A | G | 0.0954 |
| 2017 | 21-Aug-17 | FRESNO | TOMATOES, FOR PROCESSING/CANNING | Redacted4 | 10.0005 | 52 | A | G | 0.0962 |
| 2017 | 21-Aug-17 | FRESNO | TOMATOES, FOR PROCESSING/CANNING | Redacted4 | 14.0396 | 73 | A | G | 0.0962 |
| 2017 | 21-Aug-17 | FRESNO | TOMATOES, FOR PROCESSING/CANNING | Redacted4 | 14.0396 | 73 | A | G | 0.0962 |
| 2017 | 21-Aug-17 | FRESNO | TOMATOES, FOR PROCESSING/CANNING | Redacted4 | 12.1165 | 63 | A | G | 0.0962 |
| 2017 | 21-Aug-17 | FRESNO | TOMATOES, FOR PROCESSING/CANNING | Redacted4 | 13.4629 | 70 | A | G | 0.0962 |
| 2017 | 21-Aug-17 | FRESNO | TOMATOES, FOR PROCESSING/CANNING | Redacted4 | 11.9243 | 62 | A | G | 0.0962 |
| 2017 | 9-Aug-17 | FRESNO | TOMATOES, FOR PROCESSING/CANNING | Redacted4 | 16.4029 | 85 | A | G | 0.0965 |
| 2017 | 5-Sep-17 | FRESNO | TOMATOES, FOR PROCESSING/CANNING | Redacted4 | 4.871 | 25 | A | G | 0.0974 |
| 2017 | 8-Aug-17 | FRESNO | TOMATOES, FOR PROCESSING/CANNING | Redacted4 | 14.6314 | 75 | A | G | 0.0975 |
| 2017 | 28-Aug-17 | FRESNO | TOMATOES, FOR PROCESSING/CANNING | Redacted4 | 2.3515 | 12 | A | A | 0.0980 |
| 2017 | 5-Sep-17 | FRESNO | TOMATOES, FOR PROCESSING/CANNING | Redacted4 | 11.7576 | 60 | A | G | 0.0980 |
| 2017 | 5-Sep-17 | FRESNO | TOMATOES, FOR PROCESSING/CANNING | Redacted4 | 6.4667 | 33 | A | G | 0.0980 |
| 2017 | 5-Sep-17 | FRESNO | TOMATOES, FOR PROCESSING/CANNING | Redacted4 | 15.7048 | 80 | A | G | 0.0982 |
| 2017 | 30-Jun-17 | FRESNO | TOMATOES, FOR PROCESSING/CANNING | Redacted4 | 7.4745 | 38 | A | A | 0.0983 |
| 2017 | 12-Jun-17 | FRESNO | TOMATOES, FOR PROCESSING/CANNING | Redacted4 | 14.949 | 76 | A | A | 0.0983 |
| 2017 | 8-Jul-17 | FRESNO | TOMATOES, FOR PROCESSING/CANNING | Redacted4 | 29.562 | 150 | A | A | 0.0985 |
| 2017 | 28-Aug-17 | FRESNO | TOMATOES, FOR PROCESSING/CANNING | Redacted4 | 5.5429 | 28 | A | G | 0.0990 |
| 2017 | 30-Jun-17 | FRESNO | TOMATOES, FOR PROCESSING/CANNING | Redacted4 | 22.9274 | 100 | A | A | 0.1146 |
| 2017 | 12-Jun-17 | FRESNO | TOMATOES, FOR PROCESSING/CANNING | Redacted4 | 12.5975 | 48 | A | A | 0.1312 |
| 2017 | 14-Jun-17 | FRESNO | TOMATOES, FOR PROCESSING/CANNING | Redacted4 | 39.3881 | 150 | A | A | 0.1313 |
| 2017 | 12-Jun-17 | FRESNO | TOMATOES, FOR PROCESSING/CANNING | Redacted4 | 30.4859 | 116 | A | A | 0.1314 |
| 2017 | 12-Jun-17 | FRESNO | TOMATOES, FOR PROCESSING/CANNING | Redacted4 | 14.1931 | 54 | A | A | 0.1314 |
| 2017 | 12-Jun-17 | FRESNO | TOMATOES, FOR PROCESSING/CANNING | Redacted4 | 22.0875 | 84 | A | A | 0.1315 |
| 2017 | 12-Jun-17 | FRESNO | TOMATOES, FOR PROCESSING/CANNING | Redacted4 | 9.994 | 38 | A | A | 0.1315 |
| 2017 | 12-Jun-17 | FRESNO | TOMATOES, FOR PROCESSING/CANNING | Redacted4 | 12.4295 | 38 | A | A | 0.1635 |
| 2017 | 14-Jul-17 | FRESNO | TOMATOES, FOR PROCESSING/CANNING | Redacted4 | 49.2141 | 150 | A | A | 0.1640 |
| 2017 | 22-Jun-17 | KERN | TOMATOES, FOR PROCESSING/CANNING | Redacted4 | 8.3983 | 22 | A | A | 0.1909 |
| 2017 | 11-Dec-17 | VENTURA | TURNIP (TURNIP GREENS) | Redacted4 | 0.2519 | 1.8 | A | G | 0.0700 |
| 2017 | 13-Jul-17 | STANISLAUS | WALNUT (ENGLISH WALNUT, PERSIAN WALNUT) | Redacted4 | 6.8866 | 19 | A | A | 0.1812 |
| 2017 | 10-Jun-17 | MADERA | WALNUT (ENGLISH WALNUT, PERSIAN WALNUT) | Redacted4 | 183.7129 | 200 | A | G |  |
| 2017 | 2-Sep-17 | SAN JOAQUIN | WATERMELONS | Redacted4 | 16.6286 | 130 | A | G | 0.0640 |
| 2017 | 3-Aug-17 | STANISLAUS | WATERMELONS | Redacted4 | 5.5429 | 34 | A | A | 0.0815 |
| 2017 | 3-Aug-17 | STANISLAUS | WATERMELONS | Redacted4 | 4.955 | 30 | A | A | 0.0826 |
| 2017 | 2-Jul-17 | STANISLAUS | WATERMELONS | Redacted4 | 8.3983 | 46 | A | G | 0.0913 |
| 2017 | 28-Jul-17 | SANTA CLARA | WATERMELONS | Redacted4 | 1.3437 | 7 | A | G | 0.0960 |
| 2017 | 11-Sep-17 | SAN JOAQUIN | WATERMELONS | Redacted4 | 32.1655 | 166 | A | G | 0.0969 |
| 2017 | 22-Jun-17 | SAN JOAQUIN | WATERMELONS | Redacted4 | 8.3143 | 41 | A | G | 0.1014 |
| 2017 | 8-Jun-17 | SAN JOAQUIN | WATERMELONS | Redacted4 | 8.3143 | 41 | A | G | 0.1014 |
| 2017 | 11-Sep-17 | SAN JOAQUIN | WATERMELONS | Redacted4 | 8.3143 | 41 | A | G | 0.1014 |
| 2017 | 7-Jul-17 | SAN JOAQUIN | WATERMELONS | Redacted4 | 3.9472 | 18 | A | G | 0.1096 |
| 2017 | 8-Jun-17 | SAN JOAQUIN | WATERMELONS | Redacted4 | 3.9472 | 18 | A | G | 0.1096 |
| 2017 | 22-Jun-17 | SAN JOAQUIN | WATERMELONS | Redacted4 | 3.9472 | 18 | A | G | 0.1096 |
| 2017 | 2-Aug-17 | SAN JOAQUIN | WATERMELONS | Redacted4 | 6.7186 | 29 | A | G | 0.1158 |
| 2017 | 15-Aug-17 | SAN JOAQUIN | WATERMELONS | Redacted4 | 6.7186 | 29 | A | G | 0.1158 |
| 2017 | 8-Jun-17 | SAN JOAQUIN | WATERMELONS | Redacted4 | 29.3101 | 126 | A | G | 0.1163 |
| 2017 | 19-Aug-17 | SAN JOAQUIN | WATERMELONS | Redacted4 | 29.3101 | 126 | A | G | 0.1163 |
| 2017 | 22-Jul-17 | SAN JOAQUIN | WATERMELONS | Redacted4 | 29.3101 | 126 | A | G | 0.1163 |
| 2017 | 22-Jun-17 | SAN JOAQUIN | WATERMELONS | Redacted4 | 29.3101 | 126 | A | G | 0.1163 |
| 2017 | 31-Jul-17 | SAN JOAQUIN | WATERMELONS | Redacted4 | 6.2987 | 27 | A | G | 0.1166 |
| 2017 | 29-Jul-17 | SAN JOAQUIN | WATERMELONS | Redacted4 | 33.5092 | 140 | A | G | 0.1197 |
| 2017 | 1-Jul-17 | SAN JOAQUIN | WATERMELONS | Redacted4 | 33.5092 | 140 | A | G | 0.1197 |
| 2017 | 15-Jul-17 | SAN JOAQUIN | WATERMELONS | Redacted4 | 33.5092 | 140 | A | G | 0.1197 |
| 2017 | 12-Aug-17 | SAN JOAQUIN | WATERMELONS | Redacted4 | 33.5092 | 140 | A | G | 0.1197 |
| 2017 | 15-Aug-17 | SAN JOAQUIN | WATERMELONS | Redacted4 | 16.7126 | 69 | A | G | 0.1211 |
| 2017 | 20-Jul-17 | SAN JOAQUIN | WATERMELONS | Redacted4 | 16.7126 | 69 | A | G | 0.1211 |
| 2017 | 6-Jul-17 | SAN JOAQUIN | WATERMELONS | Redacted4 | 16.7126 | 69 | A | G | 0.1211 |
| 2017 | 7-Jun-17 | SAN JOAQUIN | WATERMELONS | Redacted4 | 16.7126 | 69 | A | G | 0.1211 |
| 2017 | 21-Jun-17 | SAN JOAQUIN | WATERMELONS | Redacted4 | 16.7126 | 69 | A | G | 0.1211 |
| 2017 | 11-Jul-17 | SAN JOAQUIN | WATERMELONS | Redacted4 | 40.3119 | 166 | A | G | 0.1214 |
| 2017 | 2-Aug-17 | SAN JOAQUIN | WATERMELONS | Redacted4 | 10.078 | 41 | A | G | 0.1229 |
| 2017 | 7-Aug-17 | SAN JOAQUIN | WATERMELONS | Redacted4 | 40.8158 | 166 | A | G | 0.1229 |
| 2017 | 21-Aug-17 | SAN JOAQUIN | WATERMELONS | Redacted4 | 40.8158 | 166 | A | G | 0.1229 |
| 2017 | 4-Sep-17 | SAN JOAQUIN | WATERMELONS | Redacted4 | 40.8158 | 166 | A | G | 0.1229 |
| 2017 | 24-Jul-17 | SAN JOAQUIN | WATERMELONS | Redacted4 | 40.8158 | 166 | A | G | 0.1229 |
| 2017 | 22-Aug-17 | SAN JOAQUIN | WATERMELONS | Redacted4 | 8.3983 | 34 | A | G | 0.1235 |
| 2017 | 15-Jun-17 | SAN JOAQUIN | WATERMELONS | Redacted4 | 8.3983 | 34 | A | G | 0.1235 |
| 2017 | 11-Jul-17 | SAN JOAQUIN | WATERMELONS | Redacted4 | 8.3983 | 34 | A | G | 0.1235 |
| 2017 | 5-Sep-17 | SAN JOAQUIN | WATERMELONS | Redacted4 | 8.3983 | 34 | A | G | 0.1235 |
| 2017 | 29-Jun-17 | SAN JOAQUIN | WATERMELONS | Redacted4 | 8.3983 | 34 | A | G | 0.1235 |
| 2017 | 24-Jul-17 | SAN JOAQUIN | WATERMELONS | Redacted4 | 8.3983 | 34 | A | G | 0.1235 |
| 2017 | 7-Aug-17 | SAN JOAQUIN | WATERMELONS | Redacted4 | 8.3983 | 34 | A | G | 0.1235 |
| 2017 | 15-Sep-17 | STANISLAUS | WATERMELONS | Redacted4 | 8.3983 | 34 | A | A | 0.1235 |
| 2017 | 19-Aug-17 | SAN JOAQUIN | WATERMELONS | Redacted4 | 20.9118 | 84 | A | G | 0.1245 |
| 2017 | 22-Jul-17 | SAN JOAQUIN | WATERMELONS | Redacted4 | 20.9118 | 84 | A | G | 0.1245 |
| 2017 | 25-May-17 | SAN JOAQUIN | WATERMELONS | Redacted4 | 31.4097 | 126 | A | G | 0.1246 |
| 2017 | 5-Jul-17 | SAN JOAQUIN | WATERMELONS | Redacted4 | 10.2459 | 41 | A | G | 0.1250 |
| 2017 | 19-Jul-17 | SAN JOAQUIN | WATERMELONS | Redacted4 | 10.4979 | 42 | A | G | 0.1250 |
| 2017 | 5-Jul-17 | SAN JOAQUIN | WATERMELONS | Redacted4 | 10.4979 | 42 | A | G | 0.1250 |
| 2017 | 31-Jul-17 | SAN JOAQUIN | WATERMELONS | Redacted4 | 10.4979 | 42 | A | G | 0.1250 |
| 2017 | 2-Jun-17 | STANISLAUS | WATERMELONS | Redacted4 | 6.8026 | 27 | A | G | 0.1260 |
| 2017 | 27-Jun-17 | STANISLAUS | WATERMELONS | Redacted4 | 6.8026 | 27 | A | G | 0.1260 |
| 2017 | 26-Jul-17 | STANISLAUS | WATERMELONS | Redacted4 | 6.8026 | 27 | A | G | 0.1260 |
| 2017 | 15-Jun-17 | STANISLAUS | WATERMELONS | Redacted4 | 6.8026 | 27 | A | G | 0.1260 |
| 2017 | 12-Jul-17 | STANISLAUS | WATERMELONS | Redacted4 | 6.8026 | 27 | A | G | 0.1260 |
| 2017 | 26-Jul-17 | STANISLAUS | WATERMELONS | Redacted4 | 18.3923 | 73 | A | G | 0.1260 |
| 2017 | 27-Jun-17 | STANISLAUS | WATERMELONS | Redacted4 | 18.3923 | 73 | A | G | 0.1260 |
| 2017 | 15-Jun-17 | STANISLAUS | WATERMELONS | Redacted4 | 18.3923 | 73 | A | G | 0.1260 |
| 2017 | 12-Jul-17 | STANISLAUS | WATERMELONS | Redacted4 | 18.3923 | 73 | A | G | 0.1260 |
| 2017 | 15-Jun-17 | SAN JOAQUIN | WATERMELONS | Redacted4 | 33.2573 | 130 | A | G | 0.1279 |
| 2017 | 11-Jul-17 | SAN JOAQUIN | WATERMELONS | Redacted4 | 33.2573 | 130 | A | G | 0.1279 |
| 2017 | 19-Jul-17 | SAN JOAQUIN | WATERMELONS | Redacted4 | 10.4979 | 41 | A | G | 0.1280 |
| 2017 | 25-May-17 | SAN JOAQUIN | WATERMELONS | Redacted4 | 10.4979 | 41 | A | G | 0.1280 |
| 2017 | 19-Jul-17 | SAN JOAQUIN | WATERMELONS | Redacted4 | 7.4745 | 29 | A | G | 0.1289 |
| 2017 | 22-Aug-17 | SAN JOAQUIN | WATERMELONS | Redacted4 | 33.6772 | 130 | A | G | 0.1295 |
| 2017 | 5-Sep-17 | SAN JOAQUIN | WATERMELONS | Redacted4 | 33.6772 | 130 | A | G | 0.1295 |
| 2017 | 29-Jun-17 | SAN JOAQUIN | WATERMELONS | Redacted4 | 33.6772 | 130 | A | G | 0.1295 |
| 2017 | 24-Jul-17 | SAN JOAQUIN | WATERMELONS | Redacted4 | 33.6772 | 130 | A | G | 0.1295 |
| 2017 | 7-Aug-17 | SAN JOAQUIN | WATERMELONS | Redacted4 | 33.6772 | 130 | A | G | 0.1295 |
| 2017 | 20-Aug-17 | STANISLAUS | WATERMELONS | Redacted4 | 8.9022 | 34 | A | A | 0.1309 |
| 2017 | 12-Aug-17 | SANTA CLARA | WATERMELONS | Redacted4 | 2.0996 | 8 | A | G | 0.1312 |
| 2017 | 20-Aug-17 | STANISLAUS | WATERMELONS | Redacted4 | 7.8944 | 30 | A | A | 0.1316 |
| 2017 | 20-Jul-17 | SAN JOAQUIN | WATERMELONS | Redacted4 | 4.787 | 18 | A | G | 0.1330 |
| 2017 | 5-Jul-17 | SAN JOAQUIN | WATERMELONS | Redacted4 | 33.5092 | 126 | A | G | 0.1330 |
| 2017 | 16-Aug-17 | SAN JOAQUIN | WATERMELONS | Redacted4 | 11.3377 | 42 | A | G | 0.1350 |
| 2017 | 30-Aug-17 | SAN JOAQUIN | WATERMELONS | Redacted4 | 11.1697 | 41 | A | G | 0.1362 |
| 2017 | 5-Aug-17 | SAN JOAQUIN | WATERMELONS | Redacted4 | 23.1793 | 84 | A | G | 0.1380 |
| 2017 | 15-Sep-17 | STANISLAUS | WATERMELONS | Redacted4 | 8.3983 | 30 | A | A | 0.1400 |
| 2017 | 5-Aug-17 | SAN JOAQUIN | WATERMELONS | Redacted4 | 35.5248 | 126 | A | G | 0.1410 |
| 2017 | 16-Aug-17 | SAN JOAQUIN | WATERMELONS | Redacted4 | 5.123 | 18 | A | G | 0.1423 |
| 2017 | 9-Sep-17 | SAN JOAQUIN | WATERMELONS | Redacted4 | 8.3983 | 29 | A | G | 0.1448 |
| 2017 | 26-Aug-17 | SAN JOAQUIN | WATERMELONS | Redacted4 | 8.3983 | 29 | A | G | 0.1448 |
| 2017 | 25-Sep-17 | SAN JOAQUIN | WATERMELONS | Redacted4 | 8.3983 | 29 | A | G | 0.1448 |
| 2017 | 19-Sep-17 | SAN JOAQUIN | WATERMELONS | Redacted4 | 48.4582 | 166 | A | G | 0.1460 |
| 2017 | 3-Aug-17 | SAN JOAQUIN | WATERMELONS | Redacted4 | 20.1559 | 69 | A | G | 0.1461 |
| 2017 | 3-Aug-17 | SAN JOAQUIN | WATERMELONS | Redacted4 | 5.2909 | 18 | A | G | 0.1470 |
| 2017 | 30-Aug-17 | SAN JOAQUIN | WATERMELONS | Redacted4 | 5.2909 | 18 | A | G | 0.1470 |
| 2017 | 14-Sep-17 | SAN JOAQUIN | WATERMELONS | Redacted4 | 12.5135 | 42 | A | G | 0.1490 |
| 2017 | 26-Aug-17 | SAN JOAQUIN | WATERMELONS | Redacted4 | 41.8236 | 140 | A | G | 0.1494 |
| 2017 | 9-Sep-17 | SAN JOAQUIN | WATERMELONS | Redacted4 | 41.8236 | 140 | A | G | 0.1494 |
| 2017 | 2-Sep-17 | SAN JOAQUIN | WATERMELONS | Redacted4 | 25.1109 | 84 | A | G | 0.1495 |
| 2017 | 12-Sep-17 | SAN JOAQUIN | WATERMELONS | Redacted4 | 12.5135 | 41 | A | G | 0.1526 |
| 2017 | 9-Aug-17 | SAN JOAQUIN | WATERMELONS | Redacted4 | 21.0797 | 69 | A | G | 0.1528 |
| 2017 | 16-Aug-17 | SAN JOAQUIN | WATERMELONS | Redacted4 | 8.3983 | 27 | A | G | 0.1555 |
| 2017 | 30-Aug-17 | SAN JOAQUIN | WATERMELONS | Redacted4 | 8.3983 | 27 | A | G | 0.1555 |
| 2017 | 14-Sep-17 | SAN JOAQUIN | WATERMELONS | Redacted4 | 8.3983 | 27 | A | G | 0.1555 |
| 2017 | 9-Aug-17 | STANISLAUS | WATERMELONS | Redacted4 | 24.5231 | 73 | A | G | 0.1680 |
| 2017 | 9-Aug-17 | STANISLAUS | WATERMELONS | Redacted4 | 9.0702 | 27 | A | G | 0.1680 |
| 2017 | 22-Sep-17 | SAN BENITO | WATERMELONS | Redacted4 | 0.168 | 0.5 | A | G | 0.1680 |
| 2017 | 22-Sep-17 | SAN BENITO | WATERMELONS | Redacted4 | 0.084 | 0.25 | A | G | 0.1680 |
| 2017 | 25-Jul-17 | STANISLAUS | WATERMELONS | Redacted4 | 33.1733 | 92 | A | A | 0.1803 |

| **Crop** | **90^th^ Percentile Values Application Rate (lb/A)** | | | |
| --- | --- | --- | --- | --- |
|  | **Redacted 1** | **Redacted 2** | **Redacted3** | **Redacted4** |
| Alfalfa | 0.358 |  |  |  |
| Almond | 0.302 | 0.179 |  | 0.180 |
| Artichoke |  | 0.307 |  | 0.266 |
| Avocado |  |  |  | 0.131 |
| Beans |  |  |  | 0.133 |
| Blackberry |  | 0.362 |  | 0.312 |
| Blueberry |  |  |  | 0.115 |
| Bok Choy |  |  |  | 0.136 |
| Broccoli |  |  |  | 0.166 |
| Brussel Sprouts |  |  |  | 0.264 |
| Cabbage |  |  |  | 0.235 |
| Carrots |  | 0.060 |  | 0.131 |
| Cauliflower |  |  |  | 0.164 |
| Celery |  | 0.270 |  |  |
| Cherry |  |  |  | 0.175 |
| Cilantro |  | 0.142 |  |  |
| Citrus Fruits |  |  |  | 0.134 |
| Corn |  |  |  | 0.164 |
| Endive |  | 0.231 |  |  |
| Garlic |  | 0.228 |  |  |
| Grapes | 0.189 |  |  | 0.132 |
| Grapes Wine |  | 0.360 |  | 0.197 |
| Kale |  |  |  | 0.197 |
| Leek |  | 0.227 |  |  |
| Lettuce |  | 0.271 |  | 0.133 |
| Mustard Greens |  |  |  | 0.101 |
| Onion |  |  |  | 0.230 |
| Orange | 0.075 |  |  | 0.131 |
| Peppers |  |  |  | 0.131 |
| Pistachio | 0.071 |  |  | 0.180 |
| Plum |  |  |  | 0.114 |
| Pomegranate |  |  |  | 0.230 |
| Pumpkin |  |  |  | 0.135 |
| Raspberry |  | 0.360 |  | 0.115 |
| Squash |  |  |  | 0.153 |
| Strawberry | 0.355 | 0.269 |  | 0.295 |
| Tangerine | 0.075 |  |  | 0.164 |
| Tomatoes |  |  |  | 0.197 |
| Turnip |  |  |  | 0.070 |
| Walnut |  | 0.192 |  | 0.181 |
| Watermelon |  |  |  | 0.150 |
| Wheat | 0.236 |  |  |  |
| **Overall Peak** | **0.358** | **0.360** | **0.328** | **0.312** |
|  |  |  |  |  |

“Redacted 1 – 4” indicates the product names that have been redacted from this report.

**CDPR PUR Database Queries with Post-Processing for Trisiloxane-Acetoxy**

| **Crop** | **90^th^ Percentile Values Application Rate (lb/A)** | | | | |
| --- | --- | --- | --- | --- | --- |
|  | **Redacted1** | **Redacted2** | **Redacted3** | **Redacted4** | **Redacted5** |
| Alfalfa |  | 0.0268 | 0.0390 |  | 0.2418 |
| Almond |  | 0.4197 | 0.2681 |  | 0.4857 |
| Apple |  |  | 0.4020 |  |  |
| Apricot |  | 0.2327 | 0.2709 | 0.1884 |  |
| Arugula |  |  | 0.1341 |  |  |
| Artichoke |  |  | 0.1341 |  |  |
| Asparagus |  |  | 0.0549 |  |  |
| Avocado |  | 0.2012 |  |  |  |
| Barley |  |  |  |  | 0.2434 |
| Beans |  |  | 0.2150 |  |  |
| Beets |  |  | 0.1924 |  |  |
| Blueberry |  |  | 0.2684 |  |  |
| Bok Choy |  |  | 0.1274 |  |  |
| Broccoli |  |  | 0.1341 |  | 0.2429 |
| Brussel Sprouts |  |  | 0.1501 |  |  |
| Cabbage |  | 0.1610 | 0.1341 |  | 0.2427 |
| Cantaloupe |  |  |  |  | 0.2430 |
| Carrots |  |  | 0.0670 |  |  |
| Cauliflower |  |  | 0.1341 |  | 0.2953 |
| Celery |  |  | 0.2011 |  | 0.1214 |
| Cherry |  | 0.2754 | 0.2758 | 0.1887 | 0.5149 |
| Chestnut |  |  |  |  | 0.2429 |
| Chinese Cabbage |  |  |  |  | 0.2461 |
| Cilantro |  |  | 0.2161 |  |  |
| Citrus Fruit |  | 0.5438 |  |  |  |
| Corn |  | 0.0570 | 0.0402 |  | 0.2652 |
| Cotton |  | 0.0311 | 0.0431 |  |  |
| Cucumber |  |  | 0.1860 |  |  |
| Eggplant |  | 0.0687 |  |  | 0.2459 |
| Endive |  | 0.2025 | 0.0715 |  |  |
| Flavoring/Seasonings |  |  | 0.1114 |  |  |
| Garlic |  | 0.0537 |  |  |  |
| Gourds |  |  | 0.0469 |  |  |
| Grapes |  | 0.5322 | 0.5362 |  | 0.4857 |
| Grapes, Wine | 0.1267 | 0.3545 | 0.2696 | 0.2603 | 0.6094 |
| Kale |  |  | 0.1341 |  |  |
| Lemon |  | 0.6117 | 0.5080 |  |  |
| Lettuce |  | 0.1495 | 0.2681 |  | 0.4857 |
| Nectarine |  | 0.4293 |  | 0.6039 |  |
| Melon |  |  |  |  | 0.2440 |
| Mustard |  |  | 0.1341 |  |  |
| Oats |  |  | 0.0265 |  |  |
| Olives |  |  | 0.3318 |  |  |
| Onion |  | 0.0830 | 0.2011 |  | 0.1830 |
| Orange |  | 0.6101 | 0.6033 |  |  |
| Parsley |  |  | 0.1339 |  |  |
| Peach |  | 0.4282 | 0.2148 | 0.4998 |  |
| Pear |  | 0.2232 | 0.2011 |  |  |
| Peas |  |  | 0.1341 |  | 0.0243 |
| Pecans |  |  | 0.2978 |  |  |
| Peppers |  | 0.0443 | 0.1609 |  | 0.2452 |
| Pistachio |  | 0.5365 | 0.2145 |  | 0.4899 |
| Plum |  | 0.4293 |  |  |  |
| Pluot |  | 0.2135 |  |  |  |
| Pomegranate |  | 0.2670 |  |  |  |
| Potato |  | 0.0807 | 0.0276 |  |  |
| Prune |  | 0.2467 | 0.2683 | 0.0905 | 0.4934 |
| Pumpkin |  |  | 0.0771 |  | 0.2443 |
| Radicchio |  |  | 0.2681 |  |  |
| Rice |  |  |  |  | 0.2914 |
| Safflower |  |  |  |  | 0.2413 |
| Sorghum |  |  | 0.0393 |  |  |
| Spinach |  |  | 0.1341 |  | 0.0121 |
| Squash |  |  | 0.3606 |  | 0.2429 |
| Strawberry |  | 0.4025 | 0.4022 |  |  |
| Sunflower |  |  | 0.4075 |  | 0.4204 |
| Swiss Chard |  |  | 0.1341 |  |  |
| Tangelo |  | 0.6143 | 0.5028 |  |  |
| Tangerines |  | 0.5778 | 0.5027 |  | 0.2429 |
| Tomatoes | 0.0188 | 0.0537 | 0.0541 |  | 0.3891 |
| Walnut |  | 0.5116 | 0.2681 |  | 0.5868 |
| Watermelons |  |  | 0.0804 |  | 0.1956 |
| Wheat |  | 0.0016 |  |  |  |
| **Overall Peak** | **0.1267** | **0.6143** | **0.6033** | **0.6039** | **0.6094** |
